# Enantioselective Lithiation of *N*‑Benzyl Ureas with a Chiral Lithium Amide: Dicyclopropylmethyl (Dcpm) as an Organolithium-Resistant Nitrogen Protecting Group

**DOI:** 10.1021/acs.orglett.6c02182

**Published:** 2026-06-18

**Authors:** Maria Schwarz, Jonathan Clayden

**Affiliations:** School of Chemistry, 1980 University of Bristol, Cantock’s Close, Bristol BS8 1TS, U.K.

## Abstract

Treatment of *N*-benzyl urea derivatives with a chiral lithium amide base leads to enantioselective deprotonation, generating benzyllithiums that can be trapped by reactive electrophiles, giving products in good enantiomeric excess. Sterically hindered nitrogen substituents were necessary for good enantioselectivity, and using the dicyclopropylmethyl (Dcpm) group as a new organolithium-resistant, acid-labile protecting group both facilitated the enantioselective deprotonation and enabled the synthesis of carbamate-protected primary chiral benzylamines after deprotection and solvolysis.

## Graphical abstract

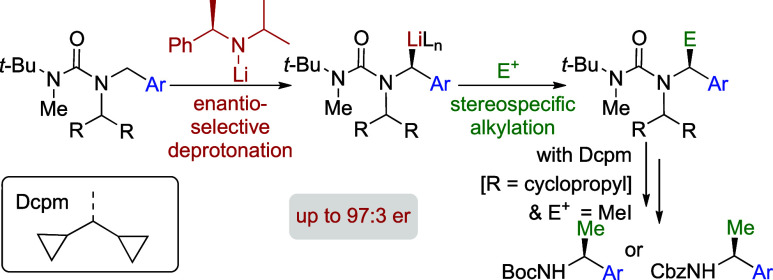

Chiral amines are key structural motifs in natural products, drugs, and agrochemicals, and enantiopure α-alkyl benzylamines in particular are found as building blocks in many pharmacologically active molecules (Scheme 1a).^1^ Transition metal-catalyzed reactions are the most widely used methods in the synthesis of chiral benzylamines (Scheme 1b).^2^ Asymmetric hydrogenation of imines and enamines^3^ is the most established process, but strategies such as enantioselective C­(sp^3^)–H amination^4^ and asymmetric addition to imines^5^ and methods entailing enantioselective lithiation^6,7^ have also been explored. More recently, biocatalytic methods are becoming more important.^8^

**Scheme 1:**
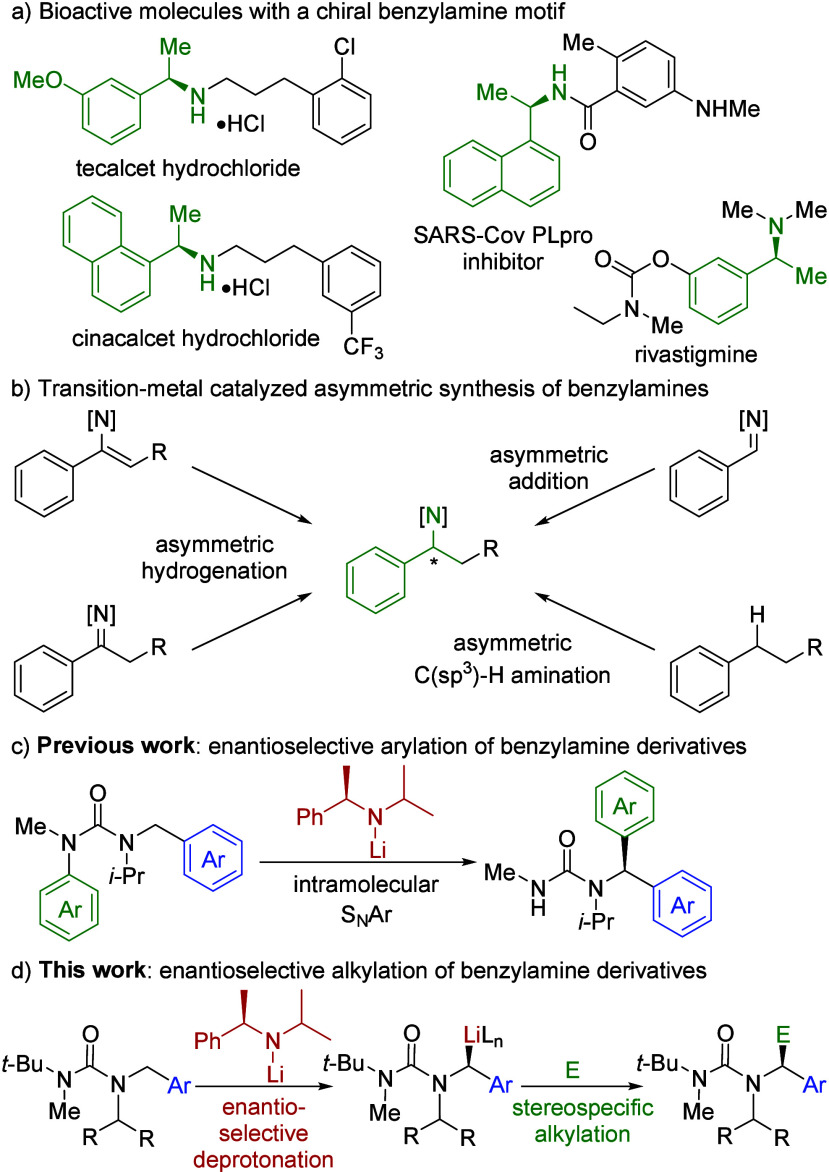
Chiral Benzylamines and Their Synthesis

We previously reported a transition metal free approach to the enantioselective α-arylation of benzylamines via a stereospecific intramolecular nucleophilic aromatic substitution (S_N_Ar) of chiral organolithium intermediates that can be generated by asymmetric benzylic deprotonation of *N*′-aryl-*N*-benzyl ureas (Scheme 1c) using a chiral lithium amide base.^7^ Configurational stability of the benzyllithium intermediate on the time scale of the aryl migration reaction guarantees its stereospecificity. In this work, we show that a benzyllithium intermediate generated by enantioselective lithiation of an appropriately substituted *N*-benzyl urea using a chiral lithium amide base can be trapped by an electrophile to undergo a stereospecific substitution (Scheme 1d). A careful choice of the urea substituents is required to ensure good enantioselectivity in the deprotonation. By using the dicyclopropylmethyl (Dcpm) group^9^ as a new protecting group^10^ on the urea nitrogen bonded to the benzyl group, the synthesis of carbamate-protected primary chiral benzylamines is possible.

Enantioselective lithiation–substitution reactions at benzylic positions of benzylamines have been reported, but all of them require at least stoichiometric amounts of enantiopure sparteine or a sparteine surrogate in combination with strong lithium bases like *s*-BuLi.^6^ The high cost and supply issues associated with those chiral ligands^11^ make the use of chiral lithium amide bases^12^ much more desirable. Urea derivatives **1** (Table 1) were therefore treated with chiral lithium amide **3** in THF at −78 °C for 5 min, followed by the addition of iodomethane. Urea **1a** with an ethyl substituent on the urea nitrogen bonded to the benzyl group formed the desired product, but in racemic form (entry 1). Pleasingly, with *i*Pr derivative **1b**, product **2b** was formed in 75% yield and 81:19 er (entry 2). Further increasing the steric bulk in **1c** to a *t*Bu group led to no conversion of the starting material (entry 3). The reaction was also sensitive to the substitution on the more remote (N′) urea nitrogen. An *N′*-*t*Bu-*N′*-Me urea was essential for high er values, as one can see in the decreased enantioselectivity for **1d** and **1e** (entries 4 and 5, respectively). Reducing the amount of base to 1.1 equiv resulted in a low yield (entry 6), which could be improved by a longer reaction time before the addition of iodomethane (entry 7), but this led to a decrease in the er. A further decrease in the er was noted when the electrophile was added after 2 h (entry 8), which suggests that the benzyllithium intermediate racemizes on a time scale of hours at this temperature and that short reaction times are required in order to maintain its configurational integrity. Less reactive electrophiles consequently resulted in products with low er values (for example, EtI gave a product in 77% yield but only 67:33 er). A change to lithium amide base **5** reduced enantioselectivity (entry 10), and though bulkier naphthyl base **4** gave a slightly improved er, the yield decreased significantly (entry 9).

**Table 1:**
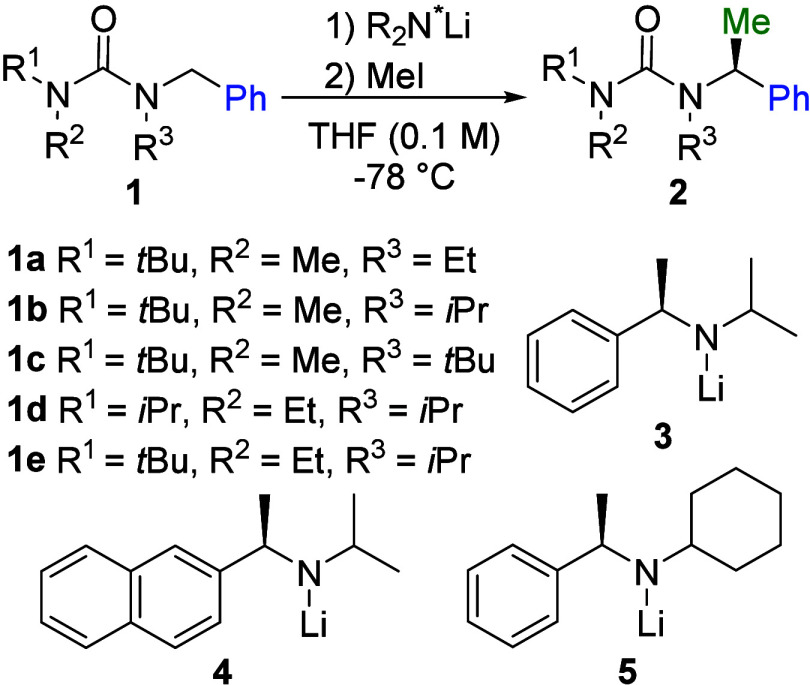
Optimization of the Asymmetric Lithiation–Substitution Reaction^a^

entry	base (equiv)	SM	time^b^	yield (%)^c^	er^d^
1^e^	**3** (2.2)	**1a**	5	73	50:50
2	**3** (2.2)	**1b**	5	75	81:19
3	**3** (2.2)	**1c**	5	0	–
4	**3** (2.2)	**1d**	5	77	54:46
5	**3** (2.2)	**1e**	5	80	69:31
6	**3** (1.1)	**1b**	5	43	80:20
7	**3** (1.1)	**1b**	30	68	68:32
8	**3** (1.1)	**1b**	120	61	50:50
9	**4** (2.2)	**1b**	5	61	83:17
10	**5** (2.2)	**1b**	5	84	77:23

a Reactions were performed using 0.25 mmol of **1** and 5 equiv of MeI.

b Reaction time in minutes before the addition of MeI.

c Isolated yield.

d The enantiomeric ratio was determined by analytical HPLC on a chiral stationary phase.

e With 3 equiv; MeI was used.

With *N*′-*tert*-butyl-*N*-benzyl-*N*-isopropyl-*N*′-methylurea providing the best selectivity, a series of substrates carrying variously substituted benzyl groups were explored (Scheme 2). A variety of substituents with different steric and electronic properties were tolerated. Importantly, halogen substituents, which are not compatible with stronger organolithium bases such as the *s*-BuLi–sparteine complex, were untouched by the chiral lithium amide.

**Scheme 2:**
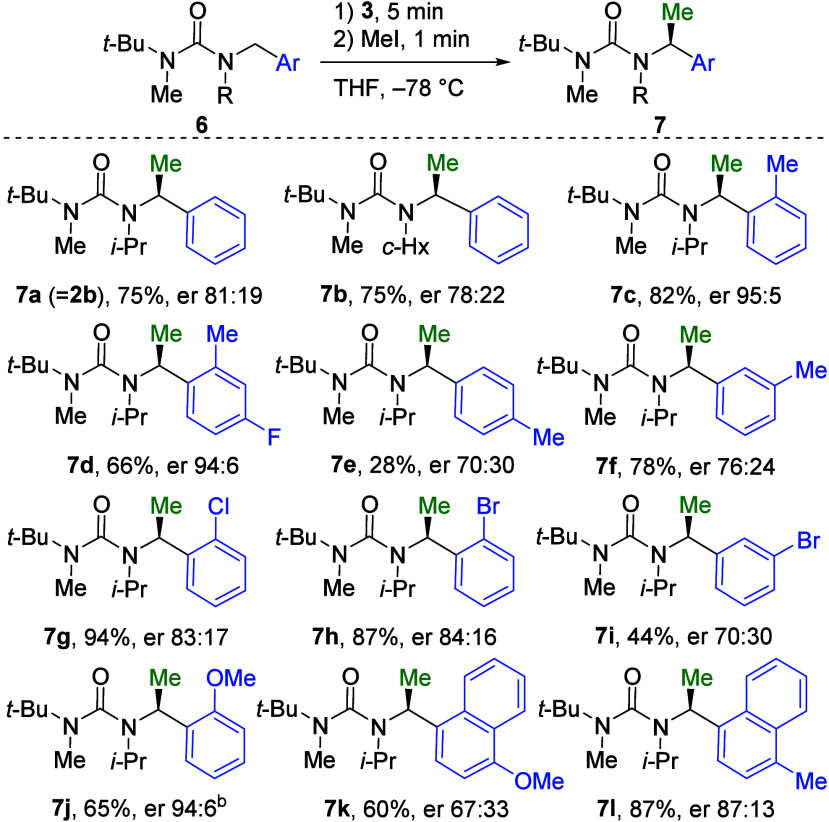
Variation of the Urea Substituents^a^

a Reactions were performed on a 0.25 mmol scale with **3** (2.2 equiv) and MeI (5 equiv) in THF (0.1 M).
b On a 0.5 mmol scale.

Alkylated products **7** formed upon treatment of a range of *N*-benzyl urea derivatives **6** with chiral lithium amide **3** at −78 °C for 5 min followed by iodomethane are summarized in Scheme 2. A cyclohexyl group (**7b**) was tolerated as an alternative substituent on the nitrogen atom adjacent to the reactive center, resulting in a yield and an er similar to those of **7a** (=**2b**). Generally, an improvement in enantioselectivity was observed for *ortho* substituents on the benzyl substituent (**7c**, **7d**, **7g**, **7h**, **7j**, and **7l**) likely a result of the increase in steric hindrance around the reaction site during the deprotonation. *Meta* substituents (**7f** and **7i**) performed somewhat less well. Electron-donating *para* substituents (**7e** and **7l**) led to a significant decrease in er and yield. Two *ortho* substituents were not tolerated (see the Supporting Information).

The absolute configuration of the products was established through the synthesis of an authentic sample of (*R*)-**7a** (see the Supporting Information), whose HPLC retention time was identical to that of the minor enantiomer on a chiral stationary phase. This confirms that the substitution occurs with retention of configuration after the preferential deprotonation of the pro-*S* proton by (*R*)-**3**,^7,13^ giving (*S*)-**7**.

Urea products **7** were transformed into *N*-isopropyl α-methylbenzylamines **12** retaining the original er, as illustrated in Scheme 3. However, primary benzylamine derivatives lacking the *i*Pr group provide more versatile and valuable building blocks for the synthesis of various bioactive molecules. We therefore sought a protecting group with steric demands similar to those of the *i*Pr group, but which can be easily removed. The dicyclopropylmethyl (Dcpm) substituent has a steric bulk akin to that of *i*Pr and was suggested as a protecting group in peptide chemistry but has not found any widespread application.^9^ Dcpm amides are expected to be stable to strong bases, but because of the exceptional ability of α-cyclopropyl substituents to stabilize carbocations, rates of solvolysis are very high and the Dcpm group can be removed under mildly acidic conditions.^14^

**Scheme 3:**
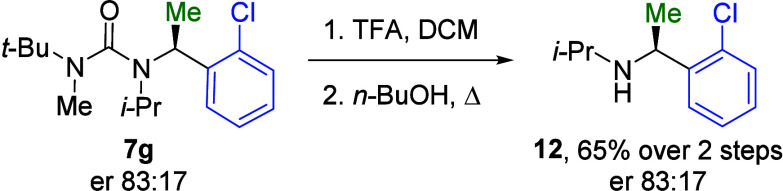
Conversion to *N*-Isopropylbenzylamine

Satisfyingly, Dcpm analogues **8** afforded products **9** with similar enantioselectivity (Scheme 4a), and in the case of **9b** and **9c**, the enantiomeric excess was even slightly larger than that of the corresponding *i*Pr derivatives. In order to achieve high yields, the amount of chiral lithium amide **3** was increased to 3 equiv and the reaction time before the addition of the electrophile was changed to 15–20 min (further details in the Supporting Information).

**Scheme 4:**
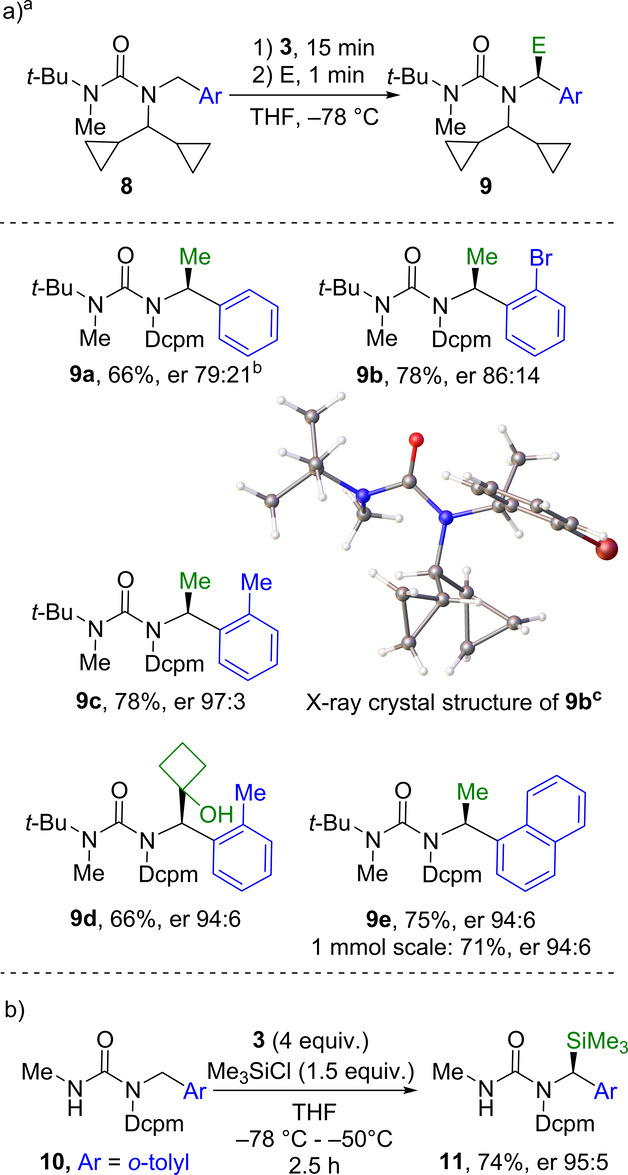
Reaction of Dcpm Derivatives

a Reactions were performed on a 0.25 mmol scale with **3** (3 equiv) and **E** (5 equiv) in THF (0.1 M). Dcpm = dicyclopropylmethyl.
b Reaction time of 20 min before the addition of MeI.
c Ellipsoid contour at the 50% probability level.

With a reactive electrophile such as cyclobutanone, urea **9d** was obtained with excellent er, but chlorotrimethylsilane under the same conditions afforded no desired product, possibly due to the increased steric bulk. Enantioselective silylation was however possible using trisubstituted urea **10** with an “unprotected” nitrogen atom. Treatment with an excess of **3** and Me_3_SiCl gave silane **11** in good yield and enantiomeric excess (Scheme 4b), presumably by way of an N-silylated intermediate.

The Dcpm group of products **9** could be easily removed under mildly acidic conditions (Scheme 5), and the hindered ureas were solvolyzed under neutral conditions^15^ to give carbamate-protected primary chiral benzylamines without loss of the enantiomeric excess.

**Scheme 5:**
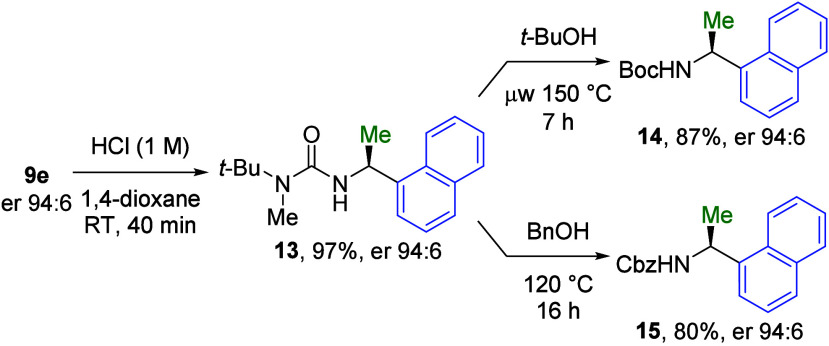
Conversion to Benzylamine Derivatives

In summary, readily available benzyl urea derivatives can be lithiated enantioselectively using a chiral lithium amide base, and the resulting organolithiums are configurationally stable enough to undergo an enantiospecific substitution with reactive electrophiles. Cleavage of the urea yields enantioenriched, pharmacologically relevant α-substituted benzylamine derivatives.

## Supplementary Material



## Data Availability

The data underlying this study are available in the published article and its Supporting Information.

## Notes

Full experimental details and characterization data for all new compounds, including copies of NMR and HPLC spectra (PDF)
