# Supplementary material for: Enantioselective Lithiation of N‑Benzyl Ureas with a Chiral Lithium Amide: Dicyclopropylmethyl (Dcpm) as an Organolithium-Resistant Nitrogen Protecting Group
Source: Org Lett. 2026 Jun 18;28(26):8425–9. doi: 10.1021/acs.orglett.6c02182 (PMC13339769; doi:10.1021/acs.orglett.6c02182)
Supplement: Supplementary file 1 [file ol6c02182_si_001.pdf]

Supporting Information to

Enantioselective lithiation of *N*-benzyl ureas with a chiral lithium amide: dicyclopropylmethyl (Dcpm) as an organolithium-resistant nitrogen protecting group

Maria Schwarz and Jonathan Clayden*

School of Chemistry, University of Bristol, Cantock's Close, Bristol BS8 1TS, U.K.

Table of Contents

1 Experimental 1

1.1 General information 1

1.2 General procedures 3

1.3 Additional optimisation 6

1.4 Experimental procedures 7

1.4.1 Starting materials 7

1.4.2 Enantioselective deprotonation-alkylation 19

1.4.2.1 Unsuccessful reactions 32

1.4.3 Conversion to benzylamine derivatives 33

2 Analytical data and spectra 38

2.1 ^{1}H and ^{13}C NMR spectra 38

2.1.1 Starting materials 38

2.1.2 Products from enantioselective deprotonation-alkylation 61

2.1.3 Products from conversion to benzylamine derivatives 84

2.2 X-ray crystallography data 89

2.3 HPLC spectra 91

3 References 119

1 Experimental

1.1 General information

All reactions were performed under a dry nitrogen atmosphere using standard Schlenk techniques in flame-dried glassware, unless otherwise stated. Air- and/or moisture-sensitive liquids and solutions were transferred via syringe into the reaction vessels through rubber septa. Reaction mixtures were stirred magnetically. Anhydrous DCM and THF were dried on an Anhydrous Engineering alumina column drying system before use. Petroleum ether refers to the petroleum ether fraction that boils in the range 40–60 °C. All reagents were purchased at the highest commercial quality, and used as received, with the following exceptions: diisopropylamine was distilled from KOH; Et_3N was stored over KOH. Reactions below 0 °C were cooled using a cryostat.

Chromatography: Flash chromatography was performed on an automated Biotage Isolera Spektra Four on pre-packed silica gel Biotage Sfär Duo columns, or on VWR silica gel (40–63 μm).

TLC: TLC was performed on aluminium backed silica plates (0.2 mm, 60 F254) which were developed using standard visualising agents: UV fluorescence (254 nm), potassium permanganate / Δ and Seebach stain/ Δ.

m.p.: Melting points, expressed in °C, were measured on a Stuart SMP30 melting point apparatus, and are uncorrected.

NMR: NMR spectra were recorded on a Bruker Avance (400, 500 or 600 MHz), Jeol ECZ (400 MHz) or Varian VNMR (400 or 500 MHz) spectrometer. Experiments were run at 298 K unless otherwise stated. ^1H chemical shifts are reported in parts per million (ppm), quoted to the nearest 0.01 ppm, relative to residual solvent: $CDCl_3$ (7.26 ppm). ^{13}C chemical shifts are reported in parts per million (ppm), quoted to the nearest 0.1 ppm, relative to residual solvent: $CDCl_3$ (77.2 ppm). ^{19}F chemical shifts are reported in parts per million (ppm), quoted to the nearest 0.1 ppm, relative to hexafluorobenzene (−164.9 ppm) as an internal standard. Spin-spin coupling constants (J) are reported in Hz, and multiplicities are reported according to the following convention: s = singlet, d = doublet, t = triplet, q = quartet, quint = quintet, sext = sextet, sept = septet, m = multiplet, br = broad, or some combination thereof. 2D NMR

experiments (COSY, HSQC and HMBC) were used, where necessary, to assign NMR spectra. Due to restricted C-N bond rotation, broadening of NMR signals is observed for many of the reported compounds.

IR: IR spectra were recorded from neat compounds on a Perkin Elmer Spectrum One FT-IR spectrometer (iD5 diamond ATR sampling accessory). Only strong and selected absorptions (v_{max} expressed in cm^{-1}) are reported.

HRMS: High resolution mass spectra were recorded by the technical staff at the University of Bristol on a Bruker Daltronics micrOTOF II (ESI), Thermo Scientific Q Exactive Plus Orbitrap LC-MS/MS System or Thermo Scientific Orbitrap Elite (ESI) mass spectrometer, with only molecular ions of interest ($[M+H]^+$) reported. The isotope of each element with the highest natural abundance was used to calculate *m*/*z* values, unless otherwise stated.

$[\alpha]_D^T$: Optical rotations, expressed in °/cm^2/g, were measured on a Bellingham and Stanley ADP220 polarimeter using a cell with a pathlength of 2.5 cm in the solvent specified, at temperature *T*, expressed in °C, at concentration *c*, expressed in g/100 mL.

HPLC: Enantiomeric ratios were determined by HPLC on an Agilent 1100 series or Agilent Technologies 1260 Infinity instrument with UV detection at 230 or 254 nm at 25 °C. Compounds were separated on a chiral Regis (*R,R*) Whelk-O® 1, a CHIRALCEL® OD-H or a CHIRALPAK® IA column with hexane:2-propanol (IPA) as the eluent or on a CHIRALPAK® IJ-3 column with MeCN:H_2O (0.5% Formic Acid) as the eluent.

Microwave: Microwave reactions were performed in a Biotage® Initiator$^+$ with fixed hold times.

1.2 General procedures

General Procedure 1: Urea starting material synthesis

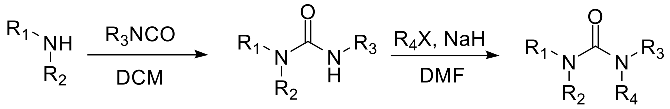

The secondary amine (1 equiv.) was added to a solution of the isocyanate (1 equiv.) in anhydrous DCM (1 M) at 0 °C under a nitrogen atmosphere and stirred at RT for 1 h. The solvent was removed under reduced pressure and the crude intermediate used in the next step without purification.

NaH (60% in mineral oil, 2 equiv.) was added to a solution of urea in anhydrous DMF (2 M) under a nitrogen atmosphere at 0 °C. The mixture was stirred for 30 minutes before the alkylhalide (2.5 equiv.) was added and stirred for a further 18 h at RT. Water was added, and the aqueous layer was extracted three times with ethyl acetate. The combined organic layers were consecutively washed with water and brine, dried over $MgSO_4$ and concentrated under reduced pressure to give the crude urea.

General Procedure 2: Urea starting material synthesis

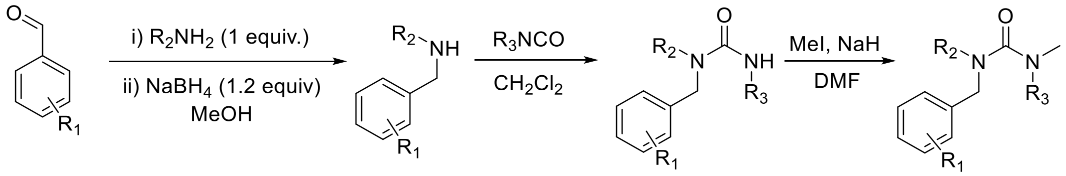

Alkylamine (1 equiv.) and benzaldehyde (1 equiv.) were dissolved in MeOH (0.4 M) and the resulting mixture was stirred for 4 h to overnight at RT. Then $NaBH_4$ (1.2 equiv.) was added portion-wise at 0 °C and the mixture allowed to warm to RT. After full conversion was observed by TLC (around 2 h), water was added to the solution, and the mixture was extracted with DCM three times. The combined organic layers were dried over $MgSO_4$ and then the solvent was removed under reduced pressure to give the secondary amine. The product was used in the next step without further purification.

The isocyanate (1 equiv.) was added to a solution of the secondary amine (1 equiv.) in anhydrous DCM (1 M) at 0 °C under a nitrogen atmosphere and stirred at RT for 1 h. The solvent was removed under reduced pressure and the crude intermediate used in the next step without purification.

NaH (60% in mineral oil, 2 equiv.) was added to a solution of urea in anhydrous DMF (2 M) under a nitrogen atmosphere at 0 °C. The mixture was stirred for 30 minutes before MeI (2.5 equiv.) was added and stirred for a further 18 h at RT. Water was added, and the aqueous layer was extracted three times with ethyl acetate. The combined organic layers were consecutively washed with water and brine, dried over $MgSO_4$ and concentrated under reduced pressure to give the crude urea.

General Procedure 3: *N*-Dicyclopropylmethyl urea starting material synthesis

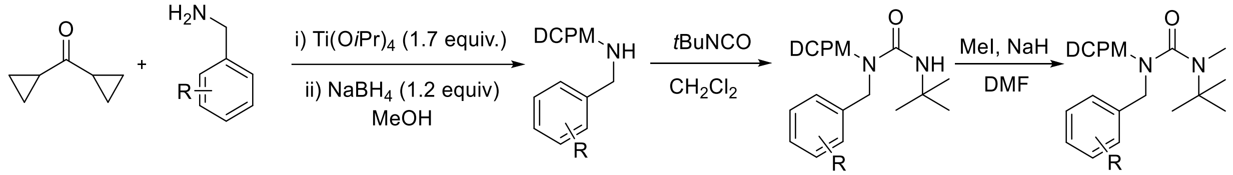

$Ti(OiPr)_4$ (1.7 equiv., 8.5 mmol, 2.53 mL) was added to the benzylamine (1 equiv.) and dicyclopropylketone (1.1 equiv., 5.5 mmol, 0.62 mL) under a nitrogen atmosphere. The mixture was stirred at RT for 18 h, before cooling to 0°C. Anhydrous MeOH (7.5 mL) and $NaBH_4$ (1.6 equiv., 8 mmol, 0.302 g) (portion-wise) were added and the mixture allowed to warm to RT. After 3 h, 1 M NaOH (aqu., 20 mL) and DCM (20 mL) were added, and the mixture was filtered through celite (washed with DCM). The layers were separated, and the aqueous layer was extracted with DCM (2 × 20 mL). The combined organic layers were washed with brine (20 mL), dried over $MgSO_4$ and then evaporated under reduced pressure to give the secondary amine. The product was used in the next step without further purification.

The secondary amine (1 equiv.) was dissolved in anhydrous DCM (5 mL) under a nitrogen atmosphere, tert-butylisocyanate (1 equiv., 5 mmol, 0.57 mL) added and the solution stirred at RT for 1 h. The solvent was removed under reduced pressure and the crude intermediate used in the next step without purification.

NaH (60% in mineral oil, 2 equiv., 10 mmol, 0.400 g) was slowly added to a solution of crude urea in anhydrous DMF (5 mL) under a nitrogen atmosphere at 0°C. The mixture was stirred at RT for 15 min before MeI (2.5 equiv., 12.5 mmol, 0.780 g) was added. The solution was stirred for a further 18 h at RT. Water (10 mL) was carefully added and the mixture was extracted with EtOAc (3 × 20 mL). The combined organic layers were washed with H_2O (50 mL) and brine (50 mL), dried over $MgSO_4$ and concentrated under reduced pressure.

General Procedure 4: Enantioselective deprotonation-alkylation of *N*-benzyl-*N*-isopropyl ureas

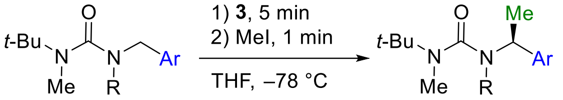

6 **7**

To a solution of (*R*)-*N*-(1-phenylethyl)-1-methylethylamine **3** (2.2 equiv.) in dry THF (0.275 M) at –78 ºC, was added n-butyl lithium (2.4 equiv., 2.5 M in hexanes) dropwise. The resulting mixture was allowed to warm to room temperature over 15 minutes, resulting in a clear yellow solution. This solution was then cooled back down to -78 °C and a solution of urea in dry THF (0.5 M) was added dropwise. The mixture was stirred at –78 ºC for 5 min. MeI (5 equiv.) was added quickly and the solution stirred for a further minute. MeOH was added to quench the reaction, the mixture allowed to warm to RT and concentrated under reduced pressure to yield the crude product.

General Procedure 5: Enantioselective deprotonation-alkylation **of** *N*-benzyl-*N*-dicyclopropylmethyl ureas

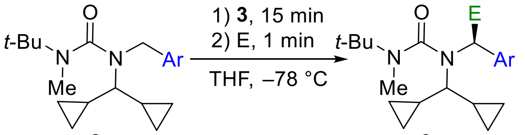

8 **9**

To a solution of (*R*)-*N*-(1-phenylethyl)-1-methylethylamine **3** (3 equiv.) in dry THF (0.375 M) at –78 ºC, was added n-butyl lithium (3.3 equiv., 2.5 M in hexanes) dropwise. The resulting mixture was allowed to warm to room temperature over 15 minutes, resulting in a clear yellow solution. This solution was then cooled back down to -78 °C and a solution of urea in dry THF (0.5 M) was added dropwise. The mixture was stirred at –78 ºC for 20 min unless otherwise stated. MeI (5 equiv.) was added quickly and the solution stirred for a further minute. MeOH was added to quench the reaction, the mixture allowed to warm to RT and concentrated under reduced pressure to yield the crude product.

1.3 Additional optimisation

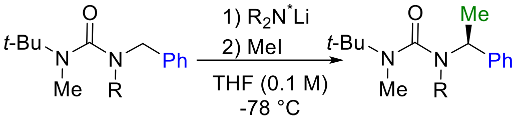

6a R = *i*Pr
8b R = DCPM

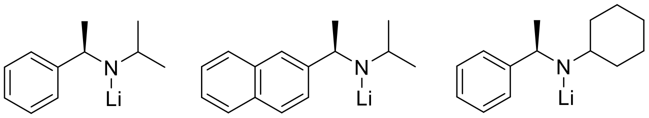

3 **4** **5**

SM	additive (equiv)	base (equiv)	temperature	time[b]	yield	er[d]
**6a**		**3** (2.2)	-78 ˚C	5	75%	81:19
**6a**		**3** (1.1)	-94 ˚C	10	no conv.	
**6a**		**3** (1.1)	-94 ˚C	120	46%	65:35
**8a**		**3** (2.2)	-78 ˚C	5	23%	85:15
**8a**		**3** (4.4)	-78 ˚C	5	43%[e]	88:12
**8a**		**3** (4.4)	-78 ˚C	30	69%	83:17
**8a**		**3** (3.3)	-78 ˚C	20	66%	79:21
**8a**		**4** (4.4)	-78 ˚C	5	trace	85:15
**8a**		**5** (4.4)	-78 ˚C	5	trace	85:15
**8a**	DMPU (2.2)	**3** (2.2)	-78 ˚C	5	32%	55:45
**8a**	DMPU (10)	**3** (2.2)	-78 ˚C	5	21%	50:50
**8a**	TMEDA (2.2)	**3** (2.2)	-78 ˚C	5	30%	84:16
**8a**	Me_2NEt (2.2)	**3** (2.2)	-78 ˚C	5	38%	85:15
**8a**	Me_2NEt (10)	**3** (2.2)	-78 ˚C	5	39%	86:14
**8a**	Me_2NEt (10)	**3** (2.2)	-78 ˚C	30	54%	70:30

[a]Reactions performed using 0.25 mmol of SM and 5 equiv. MeI. [b]Reaction time in min before addition of MeI. [c]Isolated yield. [d]Enantiomeric ratio determined by analytical HPLC on chiral stationary phase. [e] isolated product contained some impurities. Dcpm = dicyclopropylmethyl. DMPU = *N*,*N'*-Dimethylpropyleneurea. TMEDA = *N*,*N*,*N'*,*N'*-Tetramethylethylenediamine.

1.4 Experimental procedures

Chiral amines (R)-*N*-(1-phenylethyl)-1-methylethylamine[1] **3**, (R)-*N*-(1-naphthylethyl)-1-methylethylamine[1] **4** and (R)-*N*-(1-phenylethyl)cyclohexanamine[2] **5** were synthesised according to literature precedent.

Racemic samples of all products were synthesised for comparison on chiral HPLC according to General Procedure 4 or 5 using diisopropylamine instead of chiral (*R*)-*N*-(1-phenylethyl)-1-methylethylamine.

1.4.1 Starting materials

1-Benzyl-3-(*tert*-butyl)-1-ethyl-3-methylurea **1a**

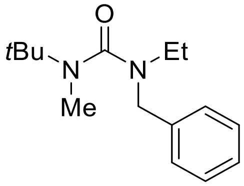

Synthesised according to general procedure 1, using *N*-methyl-*tert*-butylamine (0.60 mL, 5 mmol), ethyl isocyanate (0.40 mL, 5 mmol) and benzyl bromide (1.48 mL, 12.5 mmol). The crude urea was purified by silica gel flash column chromatography (petroleum ether + EtOAC 0-20%) to afford the title product as a colourless oil (1.165 g, 94% yield).
^{1}H NMR (400 MHz, $CDCl_3$) δ 7.37 – 7.27 (m, 2H), 7.27 – 7.11 (m, 3H), 4.43 (s, 2H), 3.17 (q, *J* = 7.1 Hz, 2H), 2.74 (s, 3H), 1.31 (s, 9H), 1.07 (t, *J* = 7.1 Hz, 3H). **^{13}C NMR** (101 MHz, $CDCl_3$) δ 165.4, 138.8, 128.6, 127.71, 127.1, 54.5, 50.2, 41.4, 34.6, 27.5, 12.8. **IR** ν_{max} = 2969, 2932, 1642, 1474, 1454, 1386, 1360, 1313 cm^{-1}. **HRMS** (ESI$^+$) *m/z* calcd for $C_{15}H_{25}N_2O$ $[M+H]^+$ 249.1961, found 249.1969.

1-Benzyl-3-(*tert*-butyl)-1-isopropyl-3-methylurea **1b = 6a**

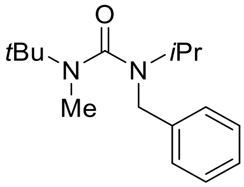

Synthesised according to general procedure 1, using *N*-benzylisopropylamine (2.51 mL, 15 mmol), *tert*-butyl isocyanate (1.71 mL, 15 mmol) and methyl iodide (2.33 mL, 37.5 mmol). The crude urea was purified by silica gel flash column chromatography (petroleum ether + EtOAC 0-20%) to afford the title product as a colourless oil (3.584 g, 91% yield).

^{1}H NMR (400 MHz, $CDCl_3$) δ 7.30 – 7.10 (m, 5H), 4.28 (s, 2H), 4.10 (sept, *J* = 6.8 Hz, 1H), 2.74 (s, 3H), 1.21 (s, 9H), 1.15 (d, *J* = 6.7 Hz, 6H). **^{13}C NMR** (101 MHz, $CDCl_3$) δ ^{13}C 165.3, 140.7, 128.3, 127.2, 126.6, 54.4, 49.9, 45.7, 34.3, 27.5, 21.0. **IR** ν_{max} = 2968, 1643, 1454, 1362, 1336 cm^{-1}. **HRMS** (ESI$^+$) *m*/*z* calcd for $C_{16}H_{27}N_2O$ [M+H]$^+$ 263.2118, found 263.2126.

1-Benzyl-1,3-di-*tert*-butyl-3-methylurea **1c**

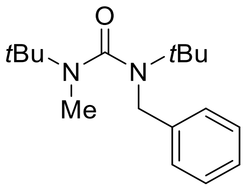

Synthesised according to general procedure 2, using *tert*-butylamine (0.43 mL, 5 mmol), benzaldehyde (0.51 mL, 5 mmol), *tert*-butyl isocyanate (0.57 mL, 5 mmol) and methyl iodide (0.78 mL, 12.5 mmol). The crude urea was purified by silica gel flash column chromatography (petroleum ether + EtOAC 0-20%) to afford the title product as a colourless solid (1.006 g, 73% yield).

m.p. = 67–69 °C. **^{1}H NMR** (400 MHz, $CDCl_3$) δ 7.30 – 7.25 (m, 4H), 7.25 – 7.18 (m, 1H), 4.29 (s, 2H), 2.77 (s, 3H), 1.30 (s, 9H), 1.18 (s, 9H). **^{13}C NMR** (101 MHz, $CDCl_3$) δ 164.8, 140.8, 128.3, 128.1, 126.9, 55.6, 54.8, 50.8, 34.4, 29.1, 27.5. **IR** ν_{max} = 2958, 2922, 1630, 1473, 1384, 1345 cm^{-1}. **HRMS** (ESI$^+$) *m*/*z* calcd for $C_{17}H_{29}N_2O$ [M+H]$^+$ 277.2274, found 277.2282.

1-Benzyl-3-ethyl-1,3-diisopropylurea **1d**

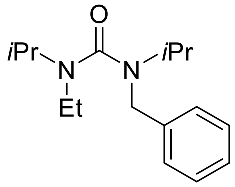

Synthesised according to general procedure 1, using *N*-ethyl-isopropylylamine (0.61 mL, 5 mmol), isopropyl isocyanate (0.49 mL, 5 mmol) and benzyl bromide (1.48 mL, 12.5 mmol). The crude urea was purified by silica gel flash column chromatography (petroleum ether + EtOAC 0-20%) to afford the title product as a colourless oil (0.729 g, 56% yield).

^{1}H NMR (400 MHz, $CDCl_3$) δ 7.32 – 7.25 (m, 4H), 7.24 – 7.16 (m, 1H), 4.24 (s, 2H), 3.97 (sept, *J* = 6.7 Hz, 1H), 3.86 (sept, *J* = 6.7 Hz, 1H), 3.07 (q, *J* = 7.0 Hz, 2H), 1.23 (d, *J* = 6.8 Hz, 6H), 1.10 (d, *J* = 6.7 Hz, 6H), 1.05 (t, *J* = 7.0 Hz, 3H). **^{13}C NMR** (101 MHz, $CDCl_3$) δ 165.2, 140.7, 128.2, 127.5, 126.5, 51.3, 50.3, 45.8, 36.0, 20.6, 20.5, 15.2. **IR** ν_{max} = 2968, 2932, 1642, 1402, 1367, 1318 cm^{-1}. **HRMS** (ESI$^+$) *m/z* calcd for $C_{16}H_{27}N_2O$ [M+H]$^+$ 263.2118, found 263.2121.

1-Benzyl-3-(*tert*-butyl)-3-ethyl-1-isopropylurea **1e**

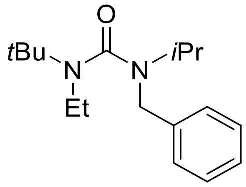

Synthesised according to general procedure 1, using *N*-ethyl-*tert*-butylylamine (0.68 mL, 5 mmol), isopropyl isocyanate (0.49 mL, 5 mmol) and benzyl bromide (1.48 mL, 12.5 mmol). The crude urea was purified by silica gel flash column chromatography (petroleum ether + EtOAC 0-20%) to afford the title product as a colourless oil (0.831 g, 60% yield).

^{1}H NMR (400 MHz, $CDCl_3$) δ 7.35 – 7.24 (m, 4H), 7.23 – 7.17 (m, 1H), 4.37 (s, 2H), 4.24 (sept, *J* = 6.8 Hz, 1H), 3.15 (q, *J* = 7.1 Hz, 2H), 1.25 (s, 9H), 1.17 (d, *J* = 6.8 Hz, 6H), 1.05 (t, *J* = 7.1 Hz, 3H). **^{13}C NMR** (101 MHz, $CDCl_3$) δ 255.1, 165.7, 140.4, 128.3, 127.8, 126.7, 55.1, 50.4, 46.5, 41.3, 28.9, 21.1, 16.2. **IR** ν_{max} = 2971, 1639, 1358, 1282, 1215 cm^{-1}. **HRMS** (ESI$^+$) *m/z* calcd for $C_{17}H_{29}N_2O$ [M+H]$^+$ 277.2274, found 277.2276.

1-Benzyl-3-(*tert*-butyl)-1-cyclohexyl-3-methylurea **6b**

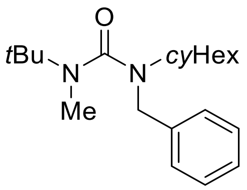

Synthesised according to general procedure 1, using *N*-methyl-*tert*-butylamine (0.60 mL, 5 mmol), cyclohexyl isocyanate (0.64 mL, 5 mmol) and benzyl bromide (1.48 mL, 12.5 mmol). The crude urea was purified by silica gel flash column chromatography (petroleum ether + EtOAC 0-20%) to afford the title product as a colourless solid (1.056 g, 70% yield).

m.p. = 46–48 °C. **^{1}H NMR** (400 MHz, $CDCl_3$) δ 7.31 – 7.22 (m, 4H), 7.22 – 7.16 (m, 1H), 4.32 (s, 2H), 3.70 (tt, *J* = 11.9, 3.4 Hz, 1H), 2.74 (s, 3H), 1.83 – 1.72 (m, 4H), 1.66 – 1.58 (m, 1H), 1.49 – 1.37 (m, 2H), 1.37 – 1.24 (m, 3H), 1.19 (s, 9H), 1.11 – 0.97 (m, 1H). **^{13}C NMR** (101 MHz, $CDCl_3$) 165.3, 140.9, 128.3, 127.2, 126.6, 58.4, 54.4, 46.9, 34.4, 31.8, 27.5, 26.4, 25.8. **IR** ν_{max} = 2928, 2852, 1626, 1450, 1358, 1321 cm^{-1}. **HRMS** (ESI$^+$) *m*/*z* calcd for $C_{19}H_{31}N_2O$ $[M+H]^+$ 303.2431, found 303.2442.

1-(*tert*-Butyl)-3-isopropyl-1-methyl-3-(2-methylbenzyl)urea **6c**

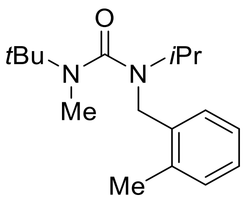

Synthesised according to general procedure 2, using isoproylamine (0.43 mL, 5 mmol), *o*-tolualdehyde (0.51 mL, 5 mmol), *tert*-butyl isocyanate (0.57 mL, 5 mmol) and methyl iodide (0.78 mL, 12.5 mmol). The crude urea was purified by silica gel flash column chromatography (petroleum ether + EtOAC 0-30%) to afford the title product as a colourless oil (0.616 g, 45% yield).

^{1}H NMR (400 MHz, $CDCl_3$) δ7.23 – 7.05 (m, 4H), 4.30 (s, 2H), 4.06 (sept, *J* = 6.8 Hz, 1H), 2.82 (s, 3H), 2.33 (s, 3H), 1.29 (s, 9H), 1.16 (d, *J* = 6.7 Hz, 6H). **^{13}C NMR** (101 MHz, $CDCl_3$) δ^{13}C NMR (101 MHz, $CDCl_3$) δ 165.5, 138.2, 135.2, 130.1, 126.4, 126.3, 125.9, 54.5, 50.3, 43.7, 34.5, 27.6, 20.7, 19.4. **IR** ν_{max} = 2968, 1644, 1458, 1361, 1334 cm^{-1}. **HRMS** (ESI$^+$) *m*/*z* calcd for $C_{17}H_{29}N_2O$ $[M+H]^+$ 277.2274, found 277.2273.

1-(*tert*-Butyl)-3-(4-fluoro-2-methylbenzyl)-3-isopropyl-1-methylurea **6d**

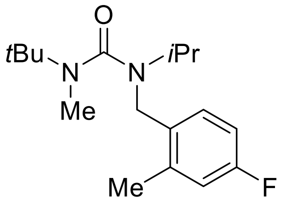

Synthesised according to general procedure 2, using isoproylamine (0.43 ml, 5 mmol), 4-fluoro-2-methylbenzaldehyde (0.60 ml, 5 mmol), *tert*-butyl isocyanate (0.57 ml, 5 mmol) and methyl iodide (0.78 ml, 12.5 mmol). The crude urea was purified by silica gel flash column chromatography (petroleum ether + EtOAC 0-15%) to afford the title product as a colourless oil (1.391 g, 94% yield).

^{1}H NMR (400 MHz, $CDCl_3$) δ 7.17 – 7.05 (m, 1H), 6.90 – 6.75 (m, 2H), 4.22 (s, 2H), 4.05 (sept, *J* = 6.8 Hz, 1H), 2.81 (s, 3H), 2.32 (s, 3H), 1.28 (s, 9H), 1.14 (d, *J* = 6.8 Hz, 6H). **^{13}C NMR** (101 MHz, $CDCl_3$) δ 165.4, 161.5 (d, *J* = 243.3 Hz), 137.5 (d, *J* = 7.6 Hz), 133.8 (d, *J* = 3.0 Hz), 128.0 (d, *J* = 8.3 Hz), 116.8 (d, *J* = 21.1 Hz), 112.4 (d, *J* = 20.9 Hz), 54.6, 50.4, 42.8, 34.5, 27.6, 20.7, 19.5 (d, *J* = 1.6 Hz). **^{19}F NMR** (377 MHz, $CDCl_3$) δ −121.3 (m, 1F). **IR** (film) ν_{max} = 2967, 1644, 1496, 1436, 1362, 1335 cm^{-1}. **HRMS** (ESI^+) *m/z* calcd for $C_{17}H_{28}FN_2O$ $[M+H]^+$ 295.2180, found 295.2167.

1-(*tert*-Butyl)-3-isopropyl-1-methyl-3-(4-methylbenzyl)urea **6e**

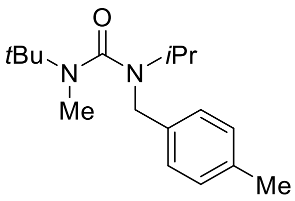

Synthesised according to general procedure 2, using isoproylamine (0.43 mL, 5 mmol), *p*-tolualdehyde (0.59 mL, 5 mmol), *tert*-butyl isocyanate (0.57 mL, 5 mmol) and methyl iodide (0.78 mL, 12.5 mmol). The crude urea was purified by silica gel flash column chromatography (petroleum ether + EtOAC 0-30%) to afford the title product as a colourless oil (1.249 g, 90% yield).

^{1}H NMR (400 MHz, $CDCl_3$) δ 7.15 (d, *J* = 8.1 Hz, 2H), 7.08 (d, *J* = 7.8 Hz, 2H), 4.25 (s, 2H), 4.09 (sept, *J* = 6.7 Hz, 1H), 2.74 (s, 3H), 2.31 (s, 3H), 1.23 (s, 9H), 1.16 (d, *J* = 6.7 Hz, 6H). **^{13}C NMR** (101 MHz, $CDCl_3$) δ 165.3, 137.6, 136.0, 129.0, 127.2, 54.4, 49.9, 45.5, 34.3, 27.5, 21.2, 21.0. **IR** ν_{max} = 2971, 1643, 1457, 1361, 1333 cm^{-1}. **HRMS** (ESI^+) *m/z* calcd for $C_{17}H_{29}N_2O$ $[M+H]^+$ 277.2274, found 277.2268.

1-(*tert*-Butyl)-3-isopropyl-1-methyl-3-(3-methylbenzyl)urea **6f**

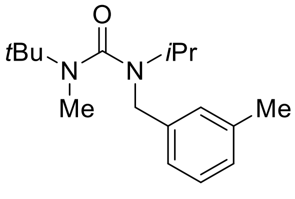

Synthesised according to general procedure 2, using isoproylamine (0.43 mL, 5 mmol), *m*-tolualdehyde (0.59 mL, 5 mmol), *tert*-butyl isocyanate (0.57 mL, 5 mmol) and methyl iodide (0.78 mL, 12.5 mmol). The crude urea was purified by silica gel flash column chromatography (petroleum ether + EtOAC 0-30%) to afford the title product as a colourless oil (1.296 g, 94% yield).

^{1}H NMR (400 MHz, $CDCl_3$) δ 7.16 (td, *J* = 7.3, 0.9 Hz, 1H), 7.10 – 6.94 (m, 3H), 4.26 (s, 2H), 4.11 (sept, *J* = 6.7 Hz, 1H), 2.75 (s, 3H), 2.31 (s, 3H), 1.22 (s, 9H), 1.16 (d, *J* = 6.8 Hz, 6H). **^{13}C NMR** (101 MHz, $CDCl_3$) δ 165.2, 140.6, 137.8, 128.2, 128.0, 127.3, 124.2, 54.4, 49.8, 45.8, 34.3, 27.5, 21.6, 21.0. **IR** ν_{max} = 2969, 1643, 1459, 1361, 1331 cm^{-1}. **HRMS** (ESI$^+$) *m*/*z* calcd for $C_{17}H_{29}N_2O$ [M+H]$^+$ 277.2274, found 277.2268.

1-(*tert*-Butyl)-3-(2-chlorobenzyl)-3-isopropyl-1-methylurea **6g**

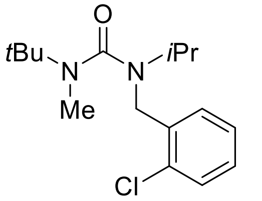

Synthesised according to general procedure 2, using isoproylamine (0.43 mL, 5 mmol), 2-chlorobenzaldehyde (0.56 mL, 5 mmol), *tert*-butyl isocyanate (0.57 mL, 5 mmol) and methyl iodide (0.78 mL, 12.5 mmol). The crude urea was purified by silica gel flash column chromatography (petroleum ether + EtOAC 0-20%) to afford the title product as a colourless oil (1.264 g, 85% yield).

^{1}H NMR (400 MHz, $CDCl_3$) δ 7.30 (dd, *J* = 7.8, 1.5 Hz, 1H), 7.28 – 7.23 (m, 1H), 7.19 (td, *J* = 7.5, 1.5 Hz, 1H), 7.13 (td, *J* = 7.6, 1.8 Hz, 1H), 4.43 (s, 2H), 4.12 (sept, *J* = 6.8 Hz, 1H), 2.81 (s, 3H), 1.27 (s, 9H), 1.15 (d, *J* = 6.8 Hz, 6H). **^{13}C NMR** (101 MHz, $CDCl_3$) δ 165.5, 137.9, 132.6, 129.4, 128.3, 127.7, 126.7, 54.6, 50.2, 43.1, 34.5, 27.5, 20.8. **IR** ν_{max} = 2968, 1645, 1470, 1443, 1362, 1334 cm^{-1}. **HRMS** (ESI$^+$) *m*/*z* calcd for $C_{16}H_{26}ClN_2O$ [M+H]$^+$ 297.1728, found 297.1717.

1-(2-Bromobenzyl)-3-(*tert*-butyl)-1-isopropyl-3-methylurea **6h**

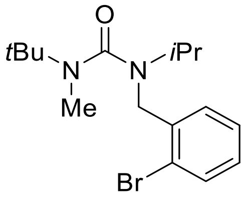

Synthesised according to general procedure 1, using *N*-(2-bromobenzyl)-isopropylamine (1.14 g, 5 mmol), *tert*-butyl isocyanate (0.57 mL, 5 mmol) and methyl iodide (0.78 mL, 12.5 mmol). The crude urea was purified by silica gel flash column chromatography (petroleum ether + EtOAC 0-30%) to afford the title product as a colourless solid (1.539 g, 90% yield).

m.p. = 55–57 °C. **^{1}H NMR** (400 MHz, $CDCl_3$) δ 7.49 (dt, *J* = 7.8, 0.9 Hz, 1H), 7.26 – 7.18 (m, 2H), 7.05 (ddd, *J* = 9.0, 4.7, 3.7 Hz, 1H), 4.41 (s, 2H), 4.13 (sept, *J* = 6.8 Hz, 1H), 2.82 (s, 3H), 1.28 (s, 9H), 1.15 (d, *J* = 6.8 Hz, 6H). **^{13}C NMR** (101 MHz, $CDCl_3$) δ 165.4, 139.4, 132.6, 128.2, 128.0, 127.3, 122.7, 54.6, 50.2, 45.8, 34.5, 27.5, 20.8. **IR** ν_{max} = 2974, 1641, 1365, 1329 cm^{-1}. **HRMS** (ESI$^+$) *m*/*z* calcd for $C_{16}H_{26}{}^{79}BrN_2O$ [M+H]$^+$ 341.1223, found 341.1228.

1-(3-Bromobenzyl)-3-(*tert*-butyl)-1-isopropyl-3-methylurea **6i**

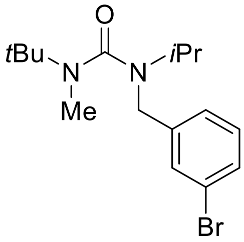

Synthesised according to general procedure 2, using isoproylamine (0.43 mL, 5 mmol), 3-bromobenzaldehyde (0.58 mL, 5 mmol), *tert*-butyl isocyanate (0.57 mL, 5 mmol) and methyl iodide (0.78 mL, 12.5 mmol). The crude urea was purified by silica gel flash column chromatography (petroleum ether + EtOAC 0-20%) to afford the title product as a colourless oil (1.468 g, 86% yield).

^{1}H NMR (400 MHz, $CDCl_3$) δ 7.38 (d, *J* = 1.9 Hz, 1H), 7.32 (dt, *J* = 7.7, 1.7 Hz, 1H), 7.22 – 7.09 (m, 2H), 4.23 (s, 2H), 4.09 (sept, *J* = 6.8 Hz, 1H), 2.76 (s, 3H), 1.23 (s, 9H), 1.16 (d, *J* = 6.8 Hz, 6H). **^{13}C NMR** (101 MHz, $CDCl_3$) δ^{13}C NMR (101 MHz, $CDCl_3$) δ 165.1, 143.2, 130.2, 129.9, 129.7, 125.9, 122.5, 54.5, 50.1, 44.9, 34.3, 27.5, 21.0. **IR** ν_{max} = 2970, 1643, 1472, 1362, 1330 cm^{-1}. **HRMS** (ESI$^+$) *m*/*z* calcd for $C_{16}H_{26}{}^{79}BrN_2O$ [M+H]$^+$ 341.1223, found 341.1213.

1-(*tert*-Butyl)-3-isopropyl-1-methyl-3-(2-methoxybenzyl)urea **6j**

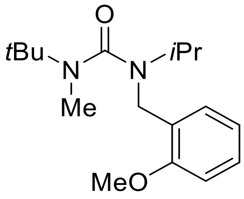

Synthesised according to general procedure 2, using isoproylamine (0.43 mL, 5 mmol), 2-methoxybenzaldehyde (0.816 g, 5 mmol), *tert*-butyl isocyanate (0.57 mL, 5 mmol) and methyl iodide (0.78 mL, 12.5 mmol). The crude urea was purified by silica gel flash column chromatography (petroleum ether + EtOAC 0-25%) to afford the title product as a colourless oil (0.633 g, 43% yield).

^{1}H NMR (400 MHz, $CDCl_3$) δ 7.23 – 7.10 (m, 2H), 6.89 (td, *J* = 7.5, 1.1 Hz, 1H), 6.82 (dd, *J* = 8.1, 1.2 Hz, 1H), 4.37 (s, 2H), 4.07 (sept, *J* = 6.7 Hz, 1H), 3.83 (s, 3H), 2.76 (s, 3H), 1.26 (s, 9H), 1.15 (d, *J* = 6.8 Hz, 6H). **^{13}C NMR** (101 MHz, $CDCl_3$) δ 165.6, 156.9, 128.7, 127.8, 127.4, 120.4, 110.0, 55.3, 54.4, 49.9, 40.7, 34.5, 27.5, 20.7. **IR** ν_{max} = 2966, 1643, 1460, 1336, 1237 cm^{-1}. **HRMS** (ESI^+) *m*/*z* calcd for $C_{17}H_{29}N_2O_2$ $[M+H]^+$ 293.2224, found 293.2225.

1-(*tert*-Butyl)-3-isopropyl-1-methyl-3-((4-methoxynaphthalen-1-yl)methyl)urea **6k**

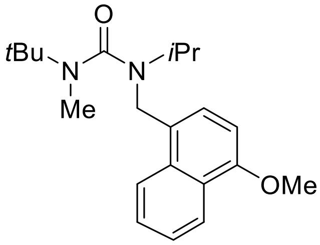

Synthesised according to general procedure 2, using isoproylamine (0.43 mL, 5 mmol), 4-methoxy-1-naphthaldehyde (0.931 g, 5 mmol), *tert*-butyl isocyanate (0.57 mL, 5 mmol) and methyl iodide (0.78 mL, 12.5 mmol). The crude urea was purified by silica gel flash column chromatography (petroleum ether + EtOAC 0-40%) to afford the title product as a colourless solid (1.465 g, 86% yield).

m.p. = 79–81 °C. **^{1}H NMR** (400 MHz, $CDCl_3$) δ 8.35 – 8.22 (m, 1H), 8.03 – 7.95 (m, 1H), 7.50 (dddd, *J* = 19.3, 8.1, 6.8, 1.4 Hz, 2H), 7.30 (dt, *J* = 7.8, 1.1 Hz, 1H), 6.76 (d, *J* = 7.9 Hz, 1H), 4.76 (s, 2H), 4.08 (sept, *J* = 6.8 Hz, 1H), 3.98 (s, 3H), 2.82 (s, 3H), 1.32 (s, 9H), 1.15 (d, *J* = 6.8 Hz, 6H). **^{13}C NMR** (101 MHz, $CDCl_3$) δ 165.6, 154.7, 132.3, 127.3, 126.4, 125.9, 125.0, 124.5, 123.1, 122.7, 103.3, 55.6, 54.6, 50.4, 44.3, 34.6, 27.6, 20.7. **IR** ν_{max} = 2965, 2935, 1621, 1585, 1440, 1338 cm^{-1}. **HRMS** (ESI^+) *m*/*z* calcd for $C_{21}H_{31}N_2O_2$ $[M+H]^+$ 343.2380, found 343.2379.

1-(*tert*-Butyl)-3-isopropyl-1-methyl-3-((4-methylnaphthalen-1-yl)methyl)urea **6l**

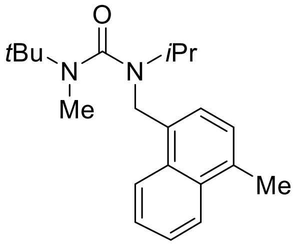

Synthesised according to general procedure 2, using isoproylamine (0.43 mL, 5 mmol), 4-methyl-1-naphthaldehyde (0.851 g, 5 mmol), *tert*-butyl isocyanate (0.57 mL, 5 mmol) and methyl iodide (0.78 mL, 12.5 mmol). The crude urea was purified by silica gel flash column chromatography (petroleum ether + EtOAC 0-20%) to afford the title product as a colourless oil (1.554 g, 95% yield).

^{1}H NMR (400 MHz, $CDCl_3$) δ 8.12 – 8.05 (m, 1H), 8.05 – 7.96 (m, 1H), 7.62 – 7.46 (m, 2H), 7.36 – 7.21 (m, 2H), 4.84 (s, 2H), 4.11 (sept, *J* = 6.7 Hz, 1H), 2.84 (s, 3H), 2.67 (s, 3H), 1.32 (s, 9H), 1.17 (d, *J* = 6.8 Hz, 6H). **^{13}C NMR** (101 MHz, $CDCl_3$) δ 165.6, 133.6, 133.2, 132.9, 131.5, 126.2, 125.5, 125.4, 125.0, 123.9, 123.7, 54.6, 50.4, 44.2, 34.6, 27.6, 20.7, 19.6. **IR** ν_{max} = 2967, 1643, 1456 cm^{-1}. **HRMS** (ESI$^+$) *m*/*z* calcd for $C_{21}H_{31}N_2O$ $[M+H]^+$ 327.2431, found 327.2419.

1-(*tert*-Butyl)-3-(2,6-dimethylbenzyl)-3-isopropyl-1-methylurea **SI-1**

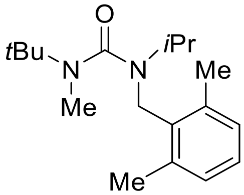

Synthesised according to general procedure 2, using isoproylamine (0.43 mL, 5 mmol), 2,6-dimethylbenzaldehyde (0.71 mL, 5 mmol), *tert*-butyl isocyanate (0.57 mL, 5 mmol) and methyl iodide (0.78 mL, 12.5 mmol). The crude urea was purified by silica gel flash column chromatography (petroleum ether + EtOAC 0-30%) to afford the title product as a colourless solid (0.917 g, 63% yield).

m.p. = 79–81 °C. **^{1}H NMR** (400 MHz, $CDCl_3$) δ7.07 (dd, *J* = 8.3, 6.5 Hz, 1H), 6.99 (d, *J* = 7.5 Hz, 2H), 4.42 (br s, 2H), 3.39 (sept, *J* = 6.8 Hz, 1H), 2.75 (s, 3H), 2.38 (s, 6H), 1.34 (s, 9H), 1.07 (br s, 6H). **^{13}C NMR** (101 MHz, $CDCl_3$) δ^{13}C NMR (101 MHz, $CDCl_3$) δ 165.1, 137.8, 135.5, 128.6, 127.2, 54.7, 50.4, 45.1, 34.2, 27.7, 20.6, 20.4 (br). **IR** ν_{max} = 2965, 1639, 1436, 1330 cm^{-1}. **HRMS** (ESI$^+$) *m*/*z* calcd for $C_{18}H_{31}N_2O$ $[M+H]^+$ 291.2431, found 291.2424.

1-Benzyl-3-(*tert*-butyl)-1-(dicyclopropylmethyl)-3-methylurea **8a**

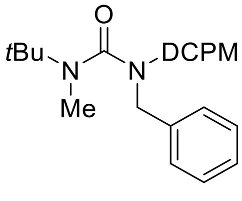

Synthesised according to general procedure 3, using benzylamine (0.56 mL, 5 mmol). The crude urea was purified by silica gel flash column chromatography (petroleum ether + EtOAC 0-20%) to afford the title product as a colourless solid (1.214 g, 77% yield).

m.p. = 74–76 °C. **^{1}H NMR** (400 MHz, $CDCl_3$) δ 7.39 (dt, *J* = 7.9, 0.9 Hz, 2H), 7.32 – 7.25 (m, 2H), 7.21 (t, *J* = 7.3 Hz, 1H), 4.52 (s, 2H), 2.82 (t, *J* = 8.6 Hz, 1H), 2.73 (s, 3H), 1.12 (s, 9H), 1.02 – 0.90 (m, 2H), 0.63 – 0.52 (m, 2H), 0.47 – 0.31 (m, 6H). **^{13}C NMR** (101 MHz, $CDCl_3$) δ 165.0, 140.7, 128.2, 127.5, 126.7, 66.1, 54.3, 48.2, 34.3, 27.2, 14.4, 5.2, 2.5. **IR** ν_{max} = 2958, 1613, 1485, 1453, 1355, 1329, 1158 cm^{-1}. **HRMS** (ESI^+) *m*/*z* calcd for $C_{20}H_{31}N_2O$ $[M+H]^+$ 315.2431, found 315.2419.

1-(2-Bromobenzyl)-3-(*tert*-butyl)-1-(dicyclopropylmethyl)-3-methylurea **8b**

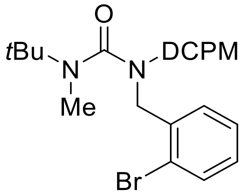

Synthesised according to general procedure 3, using 2-bromobenzylamine (0.73 g, 5 mmol). The crude urea was purified by silica gel flash column chromatography (petroleum ether + EtOAC 0-10%) to afford the title product as a colourless oil (0.752 g, 49% yield).

^{1}H NMR (400 MHz, $CDCl_3$) δ 7.48 (ddd, *J* = 7.9, 6.4, 1.5 Hz, 2H), 7.23 (td, *J* = 7.5, 1.3 Hz, 1H), 7.05 (td, *J* = 7.6, 1.7 Hz, 1H), 4.66 (s, 2H), 2.85 (t, *J* = 8.6 Hz, 1H), 2.78 (s, 3H), 1.20 (s, 9H), 0.95 – 0.84 (m, 3H), 0.60 – 0.48 (m, 2H), 0.45 – 0.22 (m, 6H). **^{13}C NMR** (101 MHz, $CDCl_3$) δ 165.5, 139.5, 132.4, 129.1, 128.0, 127.1, 122.5, 66.2, 54.5, 47.9, 34.7, 27.3, 20.8, 14.3, 10.7, 5.1, 2.7. **IR** ν_{max} = 2972, 1640, 1438, 1360, 1321, 1158 cm^{-1}. **HRMS** (ESI^+) *m*/*z* calcd for $C_{20}H_{30}{}^{79}BrN_2O$ $[M+H]^+$ 393.1536, found 393.1518.

1-(*tert*-Butyl)-3-(dicyclopropylmethyl)-1-methyl-3-(2-methylbenzyl)urea **8c**

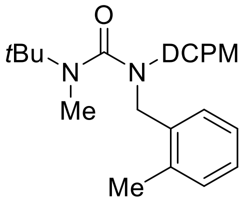

Synthesised according to general procedure 3, using 2-methylbenzylamine (0.62 mL, 5 mmol). The crude urea was purified by silica gel flash column chromatography (petroleum ether + EtOAC 0-20%) to afford the title product as a colourless solid (1.429 g, 70% yield).

m.p. = 37–39 °C. **^{1}H NMR** (400 MHz, $CDCl_3$) δ 7.42 – 7.31 (m, 1H), 7.20 – 7.06 (m, 3H), 4.56 (s, 2H), 2.78 (s, 3H), 2.71 (t, *J* = 8.7 Hz, 1H), 2.36 (s, 3H), 1.24 (s, 9H), 0.99 – 0.83 (m, 2H), 0.57 – 0.45 (m, 2H), 0.41 – 0.22 (m, 6H). **^{13}C NMR** (101 MHz, $CDCl_3$) δ 165.7, 138.2, 135.8, 130.1, 127.7, 126.6, 125.7, 66.7, 54.6, 46.5, 34.6, 27.4, 19.6, 14.35, 5.1, 2.7. **IR** ν_{max} = 2959, 1610, 1482, 1452, 1357, 1325, 1149 cm^{-1}. **HRMS** (ESI^+) *m*/*z* calcd for $C_{21}H_{33}N_2O$ $[M+H]^+$ 329.2587, found 329.2572.

1-(*tert*-Butyl)-3-(dicyclopropylmethyl)-1-methyl-3-(naphthalen-1-ylmethyl)urea **8e**

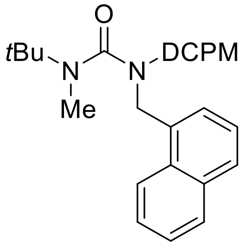

Synthesised according to general procedure 3, using 1-naphthyllamine (0.73 mL, 5 mmol). The crude urea was purified by silica gel flash column chromatography (petroleum ether + EtOAC 0-20%) to afford the title product as a colourless oil (01.392 g, 76% yield).

^{1}H NMR (400 MHz, $CDCl_3$) δ 8.24 – 8.10 (m, 1H), 7.90 – 7.81 (m, 1H), 7.73 (d, *J* = 8.2 Hz, 1H), 7.58 (dd, *J* = 7.1, 1.2 Hz, 1H), 7.54 – 7.44 (m, 2H), 7.41 (dd, *J* = 8.2, 7.1 Hz, 1H), 5.07 (s, 2H), 2.82 (s, 4H), 1.23 (s, 9H), 0.92 (dtd, *J* = 13.5, 8.3, 5.1 Hz, 2H), 0.47 (tdd, *J* = 8.8, 5.4, 4.2 Hz, 2H), 0.41 – 0.16 (m, 6H). **^{13}C NMR** (101 MHz, $CDCl_3$) δ 165.7, 135.6, 133.7, 131.7, 128.8, 127.4, 125.8, 125.5, 125.3, 125.3, 123.5, 66.8, 54.6, 46.4, 34.7, 27.3, 14.4, 5.2, 2.8. **IR** ν_{max} = 3004, 2970, 1635, 1469, 1359, 1317, 1158 cm^{-1}. **HRMS** (ESI^+) *m*/*z* calcd for $C_{24}H_{33}N_2O$ $[M+H]^+$ 365.2587, found 365.2570.

1-(Dicyclopropylmethyl)-3-methyl-1-(2-methylbenzyl)urea **10**

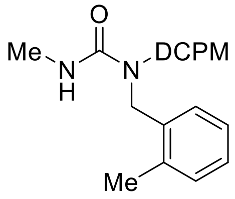

$Ti(OiPr)_4$ (2.53 mL, 8.5 mmol, 1.7 equiv.) was added to the benzylamine (1.0 equiv.) and dicyclopropylketone (0.62 mL, 5.5 mmol, 1.1 equiv.) under a nitrogen atmosphere. The mixture was stirred at RT for 18 h, before cooling to 0°C. Anhydrous MeOH (7.5 mL) and $NaBH_4$ (0.302 g, 8 mmol, 1.6 equiv.) (portion-wise) were added and the mixture allowed to warm to RT. After 3 h, 1 M NaOH (aqu., 20 mL) and DCM (20 mL) were added, and the mixture was filtered through celite (washed with DCM). The layers were separated, and the aqueous layer was extracted with DCM (2 × 20 mL). The combined organic layers were washed with brine (20 mL), dried over $MgSO_4$ and then evaporated under reduced pressure to give the secondary amine. The product was used in the next step without further purification.

The secondary amine (1 equiv.) and *N*-methylcarbamoyl chloride (0.935 g, 10 mmol, 2 equiv.) were dissolved in anhydrous acetonitrile (20 mL) under a nitrogen atmosphere. Et_3N (1.74 mL, 12.5 mmol, 2.5 equiv.) was added and the mixture was stirred at 40 °C for 18 h. Water (30 mL) was added, and the mixture was extracted with DCM (3 × 30 mL). The combined organic layers were dried over $MgSO_4$ and then the solvent was removed under reduced pressure.

The crude urea was purified by silica gel flash column chromatography (petroleum ether + EtOAC 0-60%) to afford the title product as a colourless solid (0.739 g, 57% yield).

m.p. = 123–125 °C. **^{1}H NMR** (400 MHz, $CDCl_3$) δ 7.48 (d, *J* = 6.3 Hz, 1H), 7.24 – 7.14 (m, 3H), 4.46 (s, 2H), 4.07 (q, *J* = 4.8 Hz, 1H), 3.36 (t, *J* = 9.0 Hz, 1H), 2.69 (d, *J* = 4.6 Hz, 3H), 2.33 (s, 3H), 0.97 – 0.78 (m, 2H), 0.66 – 0.51 (m, 2H), 0.50 – 0.37 (m, 4H), 0.37 – 0.21 (m, 2H). **^{13}C NMR** (101 MHz, $CDCl_3$) δ[1] 159.6, 135.8, 134.9, 130.4, 127.1, 126.3, 126.1, 63.6, 44.9, 27.8, 19.2, 14.4, 5.1, 2.8. **IR** ν_{max} = 3354, 2973, 1611, 1532, 1373, 1328 cm^{-1}. **HRMS** (ESI$^+$) *m*/*z* calcd for $C_{17}H_{25}N_2O$ [M+H]$^+$ 273.1961, found 273.1952.

1.4.2 Enantioselective deprotonation-alkylation

1-(*tert*-Butyl)-3-ethyl-1-methyl-3-(1-phenylethyl)urea **2a**

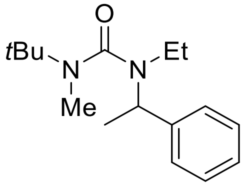

Synthesised according to general procedure 4 using 1-benzyl-3-(*tert*-butyl)-1-ethyl-3-methylurea **1a** (62.1 mg, 0.25 mmol). The reaction mixture was stirred for 15 min, before addition of MeI (3 equiv.). The crude urea was purified by silica gel flash column chromatography (petroleum ether + EtOAC 0-15%) to afford the title product as a colourless oil (48 mg, 73% yield).

^{1}H NMR (400 MHz, $CDCl_3$) δ 7.39 – 7.28 (m, 4H), 7.28 – 7.17 (m, 1H), 5.23 (q, *J* = 7.0 Hz, 1H), 3.21 – 3.01 (m, 1H), 2.87 – 2.66 (m, 4H), 1.57 (d, *J* = 7.1 Hz, 3H), 1.33 (s, 9H), 0.99 (t, *J* = 7.0 Hz, 3H). **^{13}C NMR** (101 MHz, $CDCl_3$) δ 165.3, 142.6, 128.4, 127.2, 127.0, 55.5, 54.6, 38.1, 34.5, 27.5, 17.6, 15.0. **IR** ν_{max} = 2971, 1640, 1474, 1387, 1362, 1323 cm^{-1}. **HRMS** (ESI$^+$) *m/z* calcd for $C_{16}H_{27}N_2O$ $[M+H]^+$ 263.2118, found 263.2117. **HPLC**: *er* 50:50; CHIRALCEL® OD-H, hexane:IPA = 99:1, flow = 1.0 mL/min, λ = 254 nm, tR = 5.25 mins, and 6.01 mins.

(*S*)-1-(*tert*-Butyl)-3-isopropyl-1-methyl-3-(1-phenylethyl)urea **2b = 7a**

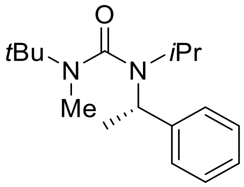

Synthesised according to general procedure 4 using 1-benzyl-3-(*tert*-butyl)-1-isopropyl-3-methylurea **6a** (65.6 mg, 0.25 mmol). The crude urea was purified by silica gel flash column chromatography (petroleum ether + EtOAC 0-15%) to afford the title product as a colourless oil (52 mg, 75% yield).

^{1}H NMR (400 MHz, $CDCl_3$) δ 7.43 – 7.34 (m, 2H), 7.34 – 7.27 (m, 2H), 7.25 – 7.18 (m, 1H), 5.06 (q, *J* = 7.0 Hz, 1H), 3.53 (sept, *J* = 6.8 Hz, 1H), 2.68 (s, 3H), 1.65 (d, *J* = 7.0 Hz, 3H), 1.43 – 1.19 (m, 12H), 1.06 (d, *J* = 6.7 Hz, 3H). **^{13}C NMR** (101 MHz, $CDCl_3$) δ 164.9 (br), 142.8 (br), 128.3, 127.3, 126.9, 54.7 (br), 54.4, 47.7, 34.3, 27.5, 21.5, 21.1, 18.1 (br). **IR** ν_{max} = 2967, 1644, 1433, 1328 cm^{-1}. **HRMS** (ESI$^+$) *m/z* calcd for $C_{17}H_{29}N_2O$ $[M+H]^+$ 277.2274, found 277.2272.

$[\alpha]_D^{23}$ = -16 (c = 1 in $CHCl_3$). **HPLC**: *er* 81:19; CHIRALCEL® OD-H, hexane:IPA = 99:1, flow = 1.0 mL/min, λ = 254 nm, tR = 4.23 min (minor), and 5.34 min (major).

(*R*)-**7a** with known absolute stereochemistry was synthesised for comparison

(*R*)-1-(*tert*-Butyl)-3-isopropyl-1-methyl-3-(1-phenylethyl)urea

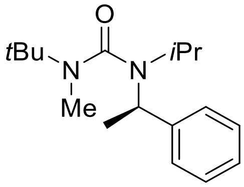

Synthesised according to general procedure 1, using (*R*)-*N*-(1-phenylethyl)propan-2-amine (0.83 mL, 5 mmol), *tert*-butyl isocyanate (0.57 mL, 5 mmol) and methyl iodide (0.78 mL, 12.5 mmol). The crude urea was purified by silica gel flash column chromatography (petroleum ether + EtOAC 0-20%) to afford the title product as a colourless oil (0.735 g, 53% yield).

^{1}H NMR (400 MHz, $CDCl_3$) δ 7.43 – 7.34 (m, 2H), 7.34 – 7.27 (m, 2H), 7.25 – 7.18 (m, 1H), 5.06 (q, *J* = 7.0 Hz, 1H), 3.53 (sept, *J* = 6.8 Hz, 1H), 2.68 (s, 3H), 1.65 (d, *J* = 7.0 Hz, 3H), 1.43 – 1.19 (m, 12H), 1.06 (d, *J* = 6.7 Hz, 3H). $[\alpha]_D^{23}$ = +28 (c = 1 in $CHCl_3$). **HPLC**: *er* 99:1; CHIRALCEL® OD-H, hexane:IPA = 99:1, flow = 1.0 mL/min, λ = 254 nm, tR = 4.23 min (major), and 5.44 min (minor).

(*S*)-1-(*tert*-Butyl)-1-ethyl-3-isopropyl -3-(1-phenylethyl)urea **SI-2**

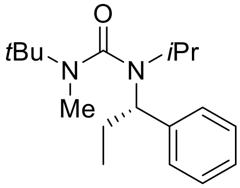

Synthesised according to general procedure 4 using 1-benzyl-3-(*tert*-butyl)-1-isopropyl-3-methylurea **6a** (65.6 mg, 0.25 mmol). Instead of MeI, ethyl iodide (5 equiv., 1.25 mmol, 0.10 mL) was added quickly as the electrophile and the solution stirred for a further 10 minutes. The crude urea was purified by silica gel flash column chromatography (petroleum ether + EtOAC 0-10%) to afford the title product as a colourless oil (54 mg, 77% yield).

^{1}H NMR (500 MHz, $CDCl_3$) δ 7.41 (b d, *J* = 7.9 Hz, 2H), 7.33 – 7.27 (m, 2H), 7.25 – 7.17 (m, 1H), 4.56 (br t, *J* = 7.3 Hz, 1H), 3.65 (br s, 1H), 2.67 (s, 3H), 2.49 – 1.78 (br m, 2H), 1.31 (s, 9H), 1.28 (br s, 3H), 1.04 – 0.86 (m, 6H). **^{13}C NMR** (126 MHz, $CDCl_3$) δ 164.9 (br), 141.0 (br), 128.3, 128.2, 127.0 (br), 62.0, 54.4, 48.4 (br), 34.2, 27.5, 25.7 (br), 21.3, 12.4. **IR** ν_{max} = 2968, 1644, 1433, 1328 cm^{-1}. **HRMS** (ESI^+) *m*/*z* calcd for $C_{18}H_{31}N_2O$ $[M+H]^+$ 291.2431, found 291.2430.

$[\alpha]_D^{25}$ = +4 (c = 1 in $CHCl_3$). **HPLC**: *er* 67:33; CHIRALCEL® OD-H, hexane:IPA = 99:1, flow = 1.0 mL/min, λ = 254 nm, tR = 3.83 min (minor), and 4.16 min (major).

(*S*)-1-Ethyl-1,3-diisopropyl-3-(1-phenylethyl)urea **2d**

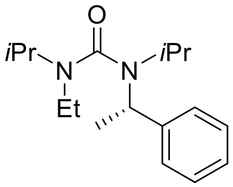

Synthesised according to general procedure 4 using 1-benzyl-3-ethyl-1,3-diisopropylurea **1d** (65.6 mg, 0.25 mmol). The crude urea was purified by silica gel flash column chromatography (petroleum ether + EtOAC 0-15%) to afford the title product as a colourless oil (53 mg, 77% yield).

^{1}H NMR (400 MHz, $CDCl_3$) δ 7.40 – 7.33 (m, 2H), 7.30 (ddd, *J* = 7.8, 6.7, 1.3 Hz, 2H), 7.25 – 7.19 (m, 1H), 4.77 (q, *J* = 6.9 Hz, 1H), 3.95 (sept, *J* = 6.7 Hz, 1H), 3.39 (sept, *J* = 6.7 Hz, 1H), 3.08 (qd, *J* = 7.0, 1.3 Hz, 2H), 1.61 (d, *J* = 6.9 Hz, 3H), 1.24 (d, *J* = 6.8 Hz, 3H), 1.15 – 1.08 (m, 9H), 1.06 (d, *J* = 6.7 Hz, 3H). **^{13}C NMR** (101 MHz, $CDCl_3$) δ 164.6, 143.4, 128.3, 127.4, 127.0, 76.8, 55.6, 50.2, 48.4, 36.3, 21.9, 20.8, 20.7, 20.4, 19.3, 15.1. **IR** ν_{max} = 2969, 2932, 1641, 1417, 1365, 1301 cm^{-1}. **HRMS** (ESI^+) *m*/*z* calcd for $C_{17}H_{29}N_2O$ $[M+H]^+$ 277.2274, found 277.2272. $[\alpha]_D^{23}$ = +4 (c = 1 in $CHCl_3$). **HPLC**: *er* 54:46; CHIRALCEL® OD-H, hexane:IPA = 99:1, flow = 1.0 mL/min, λ = 254 nm, tR = 5.04 min (major), and 5.81 min (minor).

(*S*)-1-(*tert*-Butyl)-1-ethyl-3-isopropyl-3-(1-phenylethyl)urea **2e**

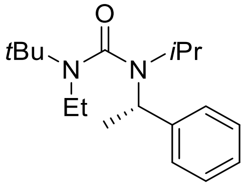

Synthesised according to general procedure 4 using 1-benzyl-3-(*tert*-butyl)-3-ethyl-1-isopropylurea **1e** (69.1 mg, 0.25 mmol). The crude urea was purified by silica gel flash column chromatography (petroleum ether + EtOAC 0-10%) to afford the title product as a colourless oil (58 mg, 80% yield).

^{1}H NMR (400 MHz, $CDCl_3$) δ 7.41 (dt, *J* = 8.3, 1.1 Hz, 2H), 7.35 – 7.27 (m, 2H), 7.25 – 7.18 (m, 1H), 5.01 (q, *J* = 7.0 Hz, 1H), 3.99 (br sept, *J* = 6.7, 6.2 Hz, 1H), 3.02 (qd, *J* = 7.1, 2.5 Hz, 2H), 1.68 (d, *J* = 7.0 Hz, 3H), 1.31 (d, *J* = 6.8 Hz, 3H), 1.25 (s, 9H), 1.18 (d, *J* = 6.8 Hz, 3H), 1.06 (t, *J* = 7.2 Hz, 3H). **^{13}C NMR** (101 MHz, $CDCl_3$) δ 164.6 (br), 142.7 (br), 128.1, 127.4, 126.7, 55.3,

54.4 (br), 48.3, 40.8, 28.8, 21.5 (br), 21.3, 18.5 (br), 15.7. **IR** v_{max} = 2970, 1643, 1421, 1359, 1339, 1274 cm^{-1}. **HRMS** (ESI$^+$) *m*/*z* calcd for $C_{18}H_{31}N_2O$ [M+H]$^+$ 291.2431, found 291.2421. $[\alpha]_D^{23}$ = -12 (c = 1 in $CHCl_3$). **HPLC**: *er* 69:31; CHIRALCEL® OD-H, hexane:IPA = 99:1, flow = 1.0 mL/min, λ = 254 nm, tR = 4.11 min (minor), and 5.57 min (major).

(*S*)-1-(*tert*-Butyl)-3-cyclohexyl-1-methyl-3-(1-phenylethyl)urea **7b**

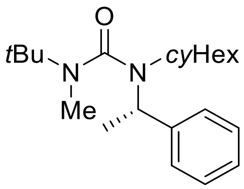

Synthesised according to general procedure 4 using 1-benzyl-3-(*tert*-butyl)-1-cyclohexyl-3-methylurea **6b** (75.6 mg, 0.25 mmol). The crude urea was purified by silica gel flash column chromatography (petroleum ether + EtOAC 0-15%) to afford the title product as a colourless oil (59 mg, 75% yield).

^{1}H NMR (400 MHz, $CDCl_3$) δ 7.37 (dt, *J* = 8.3, 1.2 Hz, 2H), 7.33 – 7.27 (m, 2H), 7.24 – 7.19 (m, 1H), 4.99 (q, *J* = 6.5 Hz, 1H), 3.16 (br t, *J* = 10.8 Hz, 1H), 2.66 (s, 3H), 1.94 (br s, 2H), 1.81 – 1.73 (m, 1H), 1.73 – 1.57 (m, 5H), 1.57 – 1.51 (m, 1H), 1.49 – 1.16 (m, 11H), 1.16 – 0.95 (m, 2H). **^{13}C NMR** (101 MHz, $CDCl_3$) δ 165.0 (br), 143.0 (br), 128.1, 127.2, 126.8, 57.2 (br), 54.7 (br), 54.3, 34.3, 31.6, 31.3, 27.5, 26.8, 26.7, 25.7, 18.1 (br). **IR** v_{max} = 2927, 2853, 1645, 1451, 1433, 1357, 1312 cm^{-1}. **HRMS** (ESI$^+$) *m*/*z* calcd for $C_{20}H_{33}N_2O$ [M+H]$^+$ 317.2597, found 317.2586. $[\alpha]_D^{23}$ = -8 (c = 1 in $CHCl_3$). **HPLC**: *er* 78:22; CHIRALCEL® OD-H, hexane:IPA = 99:1, flow = 1.0 mL/min, λ = 254 nm, tR = 4.24 min (minor), and 4.81 min (major).

(*S*)-1-(*tert*-Butyl)-3-isopropyl-1-methyl-3-(1-(*o*-tolyl)ethyl)urea **7c**

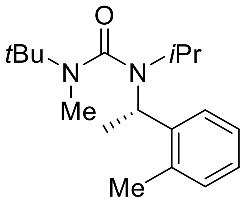

Synthesised according to general procedure 4 using 1-(*tert*-butyl)-3-isopropyl-1-methyl-3-(2-methylbenzyl)urea **6c** (69.1 mg, 0.25 mmol). The crude urea was purified by silica gel flash column chromatography (petroleum ether + EtOAC 0-15%) to afford the title product as a colourless oil (57 mg, 82% yield).

^{1}**H NMR** (400 MHz, $CDCl_3$) δ 7.49 (d, *J* = 7.2 Hz, 1H), 7.23 – 6.90 (m, 3H), 5.01 (s, 1H), 3.60 (br s, 1H), 2.74 (br s, 3H), 2.37 (s, 3H), 1.58 (d, *J* = 6.9 Hz, 3H), 1.32 (s, 9H), 1.24 (d, *J* = 6.8 Hz, 3H), 0.90 (d, *J* = 6.7 Hz, 3H). ^{13}**C NMR** (101 MHz, $CDCl_3$) δ 165.2 (br), 141.7 (br), 136.4 (br), 130.4, 127.0, 126.0, 54.9 (br), 52.7, 48.3 (br), 35.0, 27.5, 21.5 (br), 20.0 (br), 19.0. **IR** ν_{max} = 2968, 1640, 1434, 1314 cm^{-1}. **HRMS** (ESI$^+$) *m/z* calcd for $C_{18}H_{30}N_2O$ $[M+H]^+$ 291.2431, found 291.2432. $[\alpha]_D^{23}$ = +44 (c = 1 in $CHCl_3$). **HPLC**: *er* 95:5; CHIRALCEL® OD-H, hexane:IPA = 99:1, flow = 1.0 mL/min, λ = 254 nm, tR = 4.81 min (minor), and 5.55 min (major).

(*S*)-1-(*tert*-Butyl)-3-(1-(4-fluoro-2-methylphenyl)ethyl)-3-isopropyl-1-methylurea **7d**

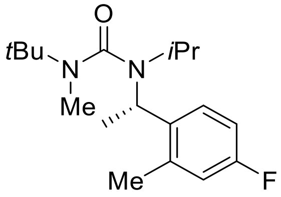

Synthesised according to general procedure 4 using 1-(*tert*-butyl)-3-(4-fluoro-2-methylbenzyl)-3-isopropyl-1-methylurea **6d** (73.6 mg, 0.25 mmol). The crude urea was purified by silica gel flash column chromatography (petroleum ether + EtOAC 0-15%) to afford the title product as a colourless solid (51 mg, 66% yield).

m.p. = 47–50 °C. ^{1}**H NMR** (400 MHz, $CDCl_3$) δ 7.47 (br s, 1H), 6.84 (ddd, *J* = 12.2, 8.7, 4.1 Hz, 2H), 4.92 (q, *J* = 7.2 Hz, 1H), 3.59 (br s, 1H), 2.74 (br s, 3H), 2.35 (s, 3H), 1.56 (d, *J* = 6.9 Hz, 3H), 1.31 (s, 9H), 1.22 (d, *J* = 6.8 Hz, 3H), 0.89 (d, *J* = 6.7 Hz, 3H). ^{13}**C NMR** (126 MHz, $CDCl_3$) δ 165.1 (br), 161.7 (d, *J* = 244.9 Hz), 138.2 (br d, *J* = 130.2 Hz), 128.7 (d, *J* = 8.2 Hz), 116.9 (d, *J* = 20.7 Hz), 112.5 (d, *J* = 20.4 Hz), 55.0 (br), 51.9 (br), 48.4 (br), 35.0, 27.5, 21.4 (br), 20.0 (br), 19.2. ^{19}**F NMR** (377 MHz, $CDCl_3$) δ −120.1 (br s, 1F). **IR** (film) ν_{max} = 2963, 1629, 1476, 1435, 1343, 1314 cm^{-1}. **HRMS** (ESI$^+$) *m/z* calcd for $C_{18}H_{30}FN_2O$ $[M+H]^+$ 309.2337, found 309.2326. $[\alpha]_D^{23}$ = +56 (c = 1 in $CHCl_3$). **HPLC**: *er* 83:17; CHIRALPAK® IJ-3, MeCN:H_2O (0.5% Formic Acid) gradient: 0 min: 40:60; 20 min: 70:30; 22 min: 70:30; flow = 1.0 mL/min, λ = 254 nm, tR = 16.76 min (major), and 17.18 min (minor).

(*S*)-1-(*tert*-Butyl)-3-isopropyl-1-methyl-3-(1-(*p*-tolyl)ethyl)urea **7e**

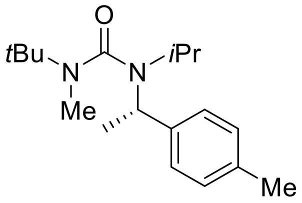

Synthesised according to general procedure 4 using 1-(*tert*-butyl)-3-isopropyl-1-methyl-3-(4-methylbenzyl)urea **6e** (69.1 mg, 0.25 mmol). The crude urea was purified by silica gel flash column chromatography (petroleum ether + EtOAC 0-10%) to afford the title product as a colourless oil (24 mg, 28% yield).

^{1}H NMR (400 MHz, $CDCl_3$) δ 7.25 (d, *J* = 7.9 Hz, 2H), 7.12 (d, *J* = 8.0 Hz, 2H), 5.06 (br q, *J* = 7.2 Hz, 1H), 3.49 (sept, *J* = 6.7 Hz, 1H), 2.67 (s, 3H), 2.33 (s, 3H), 1.63 (d, *J* = 7.0 Hz, 3H), 1.33 – 1.25 (m, 12H), 1.06 (br d, *J* = 6.7 Hz, 3H). **^{13}C NMR** (126 MHz, $CDCl_3$) δ 165.0 (br), 139.7, 136.5, 129.0, 127.2, 54.5 (br), 54.3, 47.6, 34.3, 27.5, 21.5, 21.1, 21.1, 17.8 (br). **IR** v_{max} = 2970, 1646, 1433, 1312 cm^{-1}. **HRMS** (ESI^+) *m*/*z* calcd for $C_{18}H_{31}N_2O$ $[M+H]^+$ 291.2431, found 291.2421. $[\alpha]_D^{23}$ = -12 (c = 1 in $CHCl_3$). **HPLC**: *er* 70:30; Regis (*R,R*) Whelk-O® 1, hexane:IPA = 99:1, flow = 1.0 mL/min, λ = 230 nm, tR = 6.39 min (minor), and 6.90 min (major).

(*S*)-1-(*tert*-Butyl)-3-isopropyl-1-methyl-3-(1-(*m*-tolyl)ethyl)urea **7f**

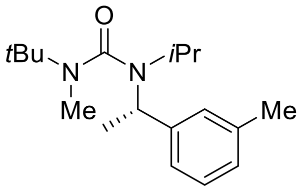

Synthesised according to general procedure 4 using 1-(*tert*-butyl)-3-isopropyl-1-methyl-3-(3-methylbenzyl)urea **6f** (69.1 mg, 0.25 mmol). The crude urea was purified by silica gel flash column chromatography (petroleum ether + EtOAC 0-10%) to afford the title product as a colourless solid (57 mg, 78% yield).

m.p. = 57–59 °C. **^{1}H NMR** (400 MHz, $CDCl_3$) δ 7.23 – 7.13 (m, 3H), 7.07 – 7.02 (m, 1H), 5.05 (br q, *J* = 7.1 Hz, 1H), 3.50 (sept, *J* = 6.8 Hz, 1H), 2.68 (s, 3H), 2.34 (s, 3H), 1.63 (d, *J* = 7.0 Hz, 3H), 1.38 – 1.21 (m, 12H), 1.06 (br d, *J* = 6.0 Hz, 3H). **^{13}C NMR** (101 MHz, $CDCl_3$) δ 165.0 (br), 142.7, 137.7, 128.1, 128.1, 127.6, 124.3, 54.9 (br), 54.4, 47.6, 34.3, 27.5, 21.7, 21.5, 21.1, 17.7 (br). **IR** v_{max} = 2970, 1646, 1433, 1316 cm^{-1}. **HRMS** (ESI^+) *m*/*z* calcd for $C_{18}H_{31}N_2O$ $[M+H]^+$ 291.2431, found 291.2420. $[\alpha]_D^{23}$ = -16 (c = 1 in $CHCl_3$). **HPLC**: *er* 76:24; Regis (*R,R*) Whelk-O®

1, hexane:IPA = 99:1, flow = 1.0 mL/min, λ = 254 nm, tR = 6.29 min (minor), and 6.77 min (major).

(*S*)-1-(*tert*-Butyl)-3-(1-(2-chlorophenyl)ethyl)-3-isopropyl-1-methylurea **7g**

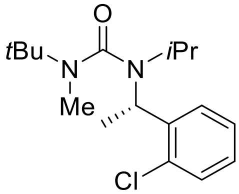

Synthesised according to general procedure 4 using 1-(*tert*-butyl)-3-(2-chlorobenzyl)-3-isopropyl-1-methylurea **6g** (74.2 mg, 0.25 mmol). The crude urea was purified by silica gel flash column chromatography (petroleum ether + EtOAC 0-15%) to afford the title product as a colourless oil (73 mg, 94% yield).

^{1}H NMR (400 MHz, $CDCl_3$) δ 7.77 (br s, 1H), 7.29 (dd, *J* = 7.8, 1.5 Hz, 1H), 7.21 (td, *J* = 7.6, 1.5 Hz, 1H), 7.13 (td, *J* = 7.6, 1.7 Hz, 1H), 5.00 (q, *J* = 6.8 Hz, 1H), 3.95 (br s, 1H), 2.67 (s, 3H), 1.63 (d, *J* = 7.0 Hz, 3H), 1.31 (s, 12H), 0.97 (br d, *J* = 6.7 Hz, 3H). **^{13}C NMR** (101 MHz, $CDCl_3$) δ 165.2, 142.6 (br), 132.6 (br), 129.4, 129.4, 127.9, 127.1, 54.5, 52.0, 49.9, 34.5, 27.5, 22.0 (br), 21.4, 19.7. **IR** v_{max} = 2972, 1643, 1472, 1433, 1331 cm^{-1}. **HRMS** (ESI^+) *m*/*z* calcd for $C_{17}H_{28}ClN_2O$ $[M+H]^+$ 311.1885, found 311.1873. $[\alpha]_D^{23}$ = +88 (c = 1 in $CHCl_3$). **HPLC**: *er* 83:17; CHIRALCEL® OD-H, hexane:IPA = 99.5:0.5, flow = 1.0 mL/min, λ = 254 nm, tR = 7.57 min (major), and 9.82 min (minor).

(*S*)-1-(1-(2-Bromophenyl)ethyl)-3-(*tert*-butyl)-1-isopropyl-3-methylurea **7h**

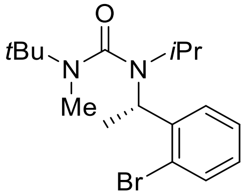

Synthesised according to general procedure 4 using 1-(2-bromobenzyl)-3-(tert-butyl)-1-isopropyl-3-methylurea **6h** (69.1 mg, 0.25 mmol). The crude urea was purified by silica gel flash column chromatography (petroleum ether + EtOAC 0-15%) to afford the title product as a colourless oil (77 mg, 87% yield).

^{1}H NMR (400 MHz, $CDCl_3$) δ 7.84 (br s, 1H), 7.49 (dd, *J* = 8.0, 1.3 Hz, 1H), 7.35 – 7.20 (m, 1H), 7.13 – 6.96 (m, 1H), 4.92 (br q, *J* = 6.6, 1H), 4.01 (br s, 1H), 2.67 (s, 3H), 1.62 (d, *J* = 7.0 Hz, 3H), 1.31 (s, 9H), 1.29 (br s, 3H), 0.97 (br s, 3H). **^{13}C NMR** (101 MHz, $CDCl_3$) δ ^{13}C NMR (101

MHz, $CDCl_3$) δ 165.3 (br), 144.8 (br), 132.8, 129.5 (br), 128.2, 127.8, 123.0, 54.6, 54.5, 50.2, 34.5, 27.5, 22.2 (br), 21.4, 19.8 (br). **IR** v_{max} = 2970, 1643, 1467, 1432, 1330 cm^{-1}. **HRMS** (ESI^+) *m/z* calcd for $C_{17}H_{28}{}^{79}BrN_2O$ $[M+H]^+$ 355.1380, found 355.1382. $[\alpha]_D^{23}$ = +100 (c = 1 in $CHCl_3$). **HPLC**: *er* 84:16; CHIRALCEL® OD-H, hexane:IPA = 99:1, flow = 1.0 mL/min, λ = 254 nm, tR = 3.91 min (major), and 4.46 min (minor).

(*S*)-1-(1-(3-Bromophenyl)ethyl)-3-(*tert*-butyl)-1-isopropyl-3-methylurea **7i**

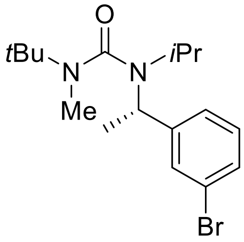

Synthesised according to general procedure 4 using 1-(3-bromobenzyl)-3-(tert-butyl)-1-isopropyl-3-methylurea **6i** (69.1 mg, 0.25 mmol). The crude urea was purified by silica gel flash column chromatography (petroleum ether + EtOAC 0-15%) to afford the title product as a colourless oil (39 mg, 44% yield).

^{1}H NMR (400 MHz, $CDCl_3$) δ 7.51 – 7.46 (m, 1H), 7.34 (ddd, *J* = 8.0, 2.2, 1.1 Hz, 1H), 7.30 (ddt, *J* = 7.8, 1.8, 1.0 Hz, 1H), 7.17 (t, *J* = 7.9 Hz, 1H), 4.86 (q, *J* = 6.9 Hz, 1H), 3.67 (sept, *J* = 6.7 Hz, 1H), 2.65 (s, 3H), 1.65 (d, *J* = 7.0 Hz, 3H), 1.33 – 1.21 (m, 12H), 1.10 (d, *J* = 6.7 Hz, 3H). **^{13}C NMR** (101 MHz, $CDCl_3$) δ^{13}C NMR (101 MHz, $CDCl_3$) δ 164.7, 145.5 (br), 130.3, 129.8, 129.8, 125.9, 122.5, 54.4, 53.8 (br), 48.3, 34.2, 27.5, 21.6, 21.3, 18.3 (br). **IR** v_{max} = 2970, 1645, 1474, 1433, 1318 cm^{-1}. **HRMS** (ESI^+) *m/z* calcd for $C_{17}H_{28}{}^{79}BrN_2O$ $[M+H]^+$ 355.1380, found 355.1371. $[\alpha]_D^{23}$ = -8 (c = 1 in $CHCl_3$). **HPLC**: *er* 70:30; CHIRALCEL® OD-H, hexane:IPA = 99:1, flow = 1.0 mL/min, λ = 230 nm, tR = 4.68 min (minor), and 6.90 min (major).

(*S*)-1-(*tert*-Butyl)-3-isopropyl-3-(1-(2-methoxyphenyl)ethyl)-1-methylurea **7j**

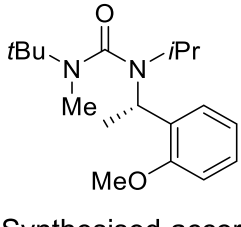

Synthesised according to general procedure 4, but on an increased scale of 0.5 mmol, using 1-(*tert*-butyl)-3-isopropyl-1-methyl-3-(2-methoxybenzyl)urea **6j** (146 mg, 0.5 mmol). The crude

urea was purified by silica gel flash column chromatography (petroleum ether + EtOAC 0-15%) to afford the title product as a colourless oil (100 mg, 65% yield).

^{1}H NMR (400 MHz, $CDCl_3$) δ 7.49 (d, *J* = 7.6 Hz, 1H), 7.24 – 7.16 (m, 1H), 6.91 (td, *J* = 7.5, 1.2 Hz, 1H), 6.83 (dd, *J* = 8.2, 1.2 Hz, 1H), 5.14 (q, *J* = 7.0 Hz, 1H), 3.82 (s, 3H), 3.73 (br s, 1H), 2.66 (s, 3H), 1.60 (d, *J* = 7.0 Hz, 3H), 1.31 (s, 9H), 1.28 (d, *J* = 6.7 Hz, 3H), 0.97 (d, *J* = 6.8 Hz, 3H). **^{13}C NMR** (101 MHz, $CDCl_3$) δ 165.3, 156.7, 132.0 (br), 128.4, 127.9, 120.4, 110.2, 55.2, 54.2, 49.6 (br), 48.5 (br), 34.4, 27.5, 21.5, 21.3 (br), 19.2. **IR** v_{max} = 2967, 1642, 1461, 1433, 1321, 1238 cm^{-1}. **HRMS** (ESI^+) *m*/*z* calcd for $C_{18}H_{31}N_2O_2$ $[M+H]^+$ 307.2380, found 307.2380. $[\alpha]_D^{23}$ = +64 (c = 1 in $CHCl_3$). **HPLC**: *er* 94:6; CHIRALCEL® OD-H, hexane:IPA = 99:1, flow = 1.0 mL/min, λ = 254 nm, tR = 4.57 min (minor), and 4.91 min (major).

(*S*)-1-(*tert*-Butyl)-3-isopropyl-1-methyl-3-(1-(4-methoxynaphthalen-1-yl)ethyl)urea **7k**

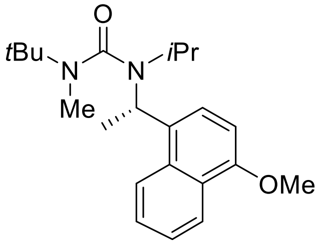

Synthesised according to general procedure 4 using 1-(*tert*-butyl)-3-isopropyl-1-methyl-3-((4-methylnaphthalen-1-yl)methyl)urea **6k** (85.6 mg, 0.25 mmol). The crude urea was purified by silica gel flash column chromatography (petroleum ether + EtOAC 0-15%) to afford the title product as a colourless solid (54 mg, 60% yield).

m.p. = 156–158 °C. **^{1}H NMR** (400 MHz, $CDCl_3$) δ^{1}H NMR (400 MHz, Chloroform-*d*) δ 8.30 (dd, *J* = 8.1, 1.6 Hz, 2H), 7.62 – 7.38 (m, 3H), 6.78 (d, *J* = 8.1 Hz, 1H), 5.71 (br s, 1H), 4.00 (s, 3H), 3.55 (br s, 1H), 2.81 (br s, 3H), 1.73 (d, *J* = 6.9 Hz, 3H), 1.41 (s, 9H), 1.21 (d, *J* = 6.8 Hz, 3H), 0.67 (br s, 3H). **^{13}C NMR** (126 MHz, $CDCl_3$) δ 164.3 (br), 155.1, 133.0, 130.4 (br), 126.7, 125.8, 125.0, 124.8, 124.2 (br), 122.6, 102.9, 55.6, 55.0 (br), 51.5, 47.0 (br), 35.3 (br), 27.5, 21.4 (br), 18.5. **IR** v_{max} = 2963, 2930, 1621, 1469, 1342, 1318 cm^{-1}. **HRMS** (ESI^+) *m*/*z* calcd for $C_{22}H_{33}N_2O_2$ $[M+H]^+$ 357.2537, found 357.2546. $[\alpha]_D^{23}$ = +20 (c = 1 in $CHCl_3$). **HPLC**: *er* 67:33; CHIRALCEL® OD-H, hexane:IPA = 99:1, flow = 1.0 mL/min, λ = 254 nm, tR = 6.00 min (minor), and 10.23 min (major).

(*S*)-1-(*tert*-Butyl)-3-isopropyl-1-methyl-3-(1-(4-methylnaphthalen-1-yl)ethyl)urea **7l**

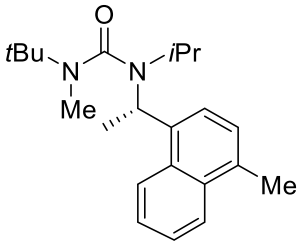

Synthesised according to general procedure 4 using 1-(*tert*-butyl)-3-isopropyl-1-methyl-3-((4-methylnaphthalen-1-yl)methyl)urea **6l** (81.6 mg, 0.25 mmol). The crude urea was purified by silica gel flash column chromatography (petroleum ether + EtOAC 0-15%) to afford the title product as a colourless solid (74 mg, 87% yield).

m.p. = 135–138 °C. **^{1}H NMR** (400 MHz, $CDCl_3$) δ 8.32 (br s, 1H), 8.06 – 7.96 (m, 1H), 7.61 – 7.37 (m, 3H), 7.30 (dd, *J* = 7.3, 1.0 Hz, 1H), 5.77 (br s, 1H), 3.60 (br s, 1H), 2.79 (br s, 3H), 2.69 (s, 3H), 1.74 (d, *J* = 6.9 Hz, 3H), 1.40 (s, 9H), 1.21 (d, *J* = 6.8 Hz, 3H), 0.67 (br s, 3H). **^{13}C NMR** (126 MHz, $CDCl_3$) δ 164.4 (br), 136.7 (br), 134.1 (br), 132.9, 132.1, 126.1, 125.8, 125.5, 124.9, 124.8 (br), 124.4, 55.0 (br), 51.7, 47.4 (br), 35.2, 27.5, 21.3 (br), 19.7, 18.6. **IR** v_{max} = 2979, 1627, 1435, 1341, 1318 cm^{-1}. **HRMS** (ESI$^+$) *m*/*z* calcd for $C_{22}H_{33}N_2O$ $[M+H]^+$ 341.2587, found 341.2572. $[\alpha]_D^{23}$ = +56 (c = 1 in $CHCl_3$). **HPLC**: *er* 87:13; CHIRALCEL® OD-H, hexane:IPA = 99:1, flow = 1.0 mL/min, λ = 254 nm, tR = 4.84 min (minor), and 11.19 min (major).

(*S*)-1-(*tert*-Butyl)-3-(dicyclopropylmethyl)-1-methyl-3-(1-phenylethyl)urea **9a**

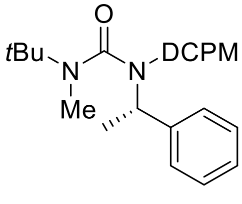

Synthesised according to general procedure 5 using 1-benzyl-3-(*tert*-butyl)-1-(dicyclopropylmethyl)-3-methylurea **8a** (79 mg, 0.25 mmol), stirring for 15 min before addition of MeI. The crude urea was purified by silica gel flash column chromatography (petroleum ether + EtOAC 0-15%) to afford the title product as a colourless oil (54 mg, 66% yield).

^{1}H NMR (400 MHz, $CDCl_3$) δ 7.44 (d, *J* = 7.3 Hz, 2H), 7.36 – 7.24 (m, 3H), 7.24 – 7.12 (m, 1H), 5.03 (q, *J* = 7.0 Hz, 1H), 2.70 (s, 3H), 2.40 (br s, 1H), 1.73 (d, *J* = 7.0 Hz, 3H), 1.39 – 1.13 (m, 11H), 0.70 – 0.55 (m, 1H), 0.55 – 0.43 (m, 1H), 0.43 – 0.08 (m, 5H), -0.20 (br s, 1H). **^{13}C NMR** (101 MHz, $CDCl_3$) δ 165.1, 143.5, 127.9, 127.5, 126.5, 65.3, 54.3, 53.9 (br), 34.6, 27.3, 18.6 (br), 14.6 (br), 14.3 (br), 6.1 (br), 3.0 (br), 3.0. **IR** v_{max} = 2971, 1645, 1430, 1360, 1308 cm^{-1}. **HRMS** (ESI$^+$) *m*/*z* calcd for $C_{21}H_{33}N_2O$ $[M+H]^+$ 329.2587, found 329.2571. $[\alpha]_D^{23}$ = -8 (c = 1 in

$CHCl_3$). **HPLC**: *er* 79:21; CHIRALCEL® OD-H, hexane:IPA = 99:1, flow = 1.0 mL/min, λ = 230 nm, tR = 4.63 min (minor), and 6.55 min (major).

(*S*)-1-(1-(2-Bromophenyl)ethyl)-3-(*tert*-butyl)-1-(dicyclopropylmethyl)-3-methylurea **9b**

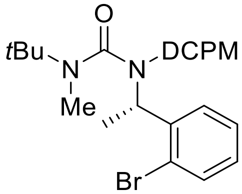

Synthesised according to general procedure 5 using 1-(2-bromobenzyl)-3-(tert-butyl)-1-(dicyclopropylmethyl)-3-methylurea **8b** (98 mg, 0.25 mmol). The crude urea was purified by silica gel flash column chromatography (petroleum ether + EtOAC 0-15%) to afford the title product as a colourless oil (79 mg, 78% yield).

m.p. = 92–96 °C. **^{1}H NMR** (400 MHz, $CDCl_3$) δ 8.57 – 7.60 (br m, 1H), 7.45 (dd, *J* = 8.0, 1.3 Hz, 1H), 7.33 – 7.16 (m, 1H), 7.01 (td, *J* = 7.6, 1.7 Hz, 1H), 5.21 (br s, 1H), 3.38 – 2.14 (m, 4H), 1.65 (d, *J* = 7.0 Hz, 3H), 1.55 – 0.93 (m, 11H), 0.75 – -0.33 (m, 8H). **^{13}C NMR** (151 MHz, $CDCl_3$) δ 165.2 (br), 145.1, 132.4, 131.3 (br), 127.9, 127.5, 122.7, 66.6, 54.8, 54.6, 34.9, 27.4, 19.8 (br), 14.3, 13.9 (br), 6.8 (br), 4.2 (br), 2.9 (br), 2.0 (br). **IR** ν_{max} = 2963, 2928, 1635, 1468, 1435, 1347, 1322 cm^{-1}. **HRMS** (ESI^+) *m/z* calcd for $C_{21}H_{32}{}^{79}BrN_2O$ $[M+H]^+$ 407.1693, found 407.1676. $[\alpha]_D^{23}$ = +148 (c = 1 in $CHCl_3$). **HPLC**: *er* 86:14; CHIRALCEL® OD-H, hexane:IPA = 99:1, flow = 0.5 mL/min, λ = 254 nm, tR = 7.80 min (major), and 8.71 min (minor).

(*S*)-1-(*tert*-Butyl)-3-(dicyclopropylmethyl)-1-methyl-3-(1-(o-tolyl)ethyl)urea **9c**

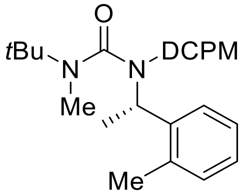

Synthesised according to general procedure 5 using 1-(*tert*-butyl)-3-(dicyclopropylmethyl)-1-methyl-3-(2-methylbenzyl)urea **8c** (82 mg, 0.25 mmol). The crude urea was purified by silica gel flash column chromatography (petroleum ether + EtOAC 0-15%) to afford the title product as a colourless oil (67 mg, 78% yield).

m.p. = 75–77 °C. **^{1}H NMR** (400 MHz, $CDCl_3$) δ 7.58 (br s, 1H), 7.21 – 6.98 (m, 3H), 5.16 (br s, 1H), 2.85 (s, 3H), 2.67 – 1.80 (m, 4H), 1.61 (d, *J* = 6.9 Hz, 3H), 1.52 – 0.99 (m, 11H), 0.68 – 0.52 (m, 1H), 0.50 – 0.21 (m, 4H), 0.18 – -0.11 (m, 2H), -0.40 (br s, 1H). **^{13}C NMR** (151 MHz,

$CDCl_3$) δ 165.5, 142.3 (br), 136.1 (br), 130.0, 127.4 (br), 126.7, 125.8, 65.7, 54.8 (br), 52.0 (br), 35.2, 27.4, 19.7 (br), 14.7, 14.4, 5.3 (br), 3.0 (br). **IR** ν_{max} = 2974, 2928, 1635, 1435, 1347, 1320 cm^{-1}. **HRMS** (ESI^+) *m/z* calcd for $C_{22}H_{35}N_2O$ $[M+H]^+$ 343.2744, found 343.2730. $[\alpha]_D^{23}$ = +100 (c = 1 in $CHCl_3$). **HPLC**: *er* 97:3; CHIRALCEL® OD-H, hexane:IPA = 99:1, flow = 0.2 mL/min, λ = 254 nm, tR = 24.56 min (minor), and 25.38 min (major).

(*R*)-1-(*tert*-Butyl)-3-(dicyclopropylmethyl)-3-((1-hydroxycyclobutyl)(phenyl)methyl)-1-methylurea **9d**

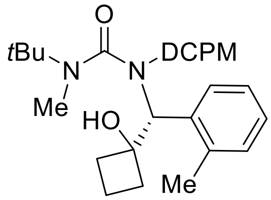

Synthesised according to general procedure 5 using 1-(*tert*-butyl)-3-(dicyclopropylmethyl)-1-methyl-3-(2-methylbenzyl)urea **8c** (82 mg, 0.25 mmol). Instead of MeI, cyclobutanone (5 equiv., 1.25 mmol, 0.09 mL) was added quickly as the electrophile and the solution stirred for a further 5 minutes. The crude urea was purified by silica gel flash column chromatography (petroleum ether + EtOAC 0-15%) to afford the title product as a colourless solid (66 mg, 66% yield).

NMR reported as a rotameric mixture.

m.p. = 100–103 °C. **^{1}H NMR** (600 MHz, $CDCl_3$) δ 8.13 (br s, 0.6H), 7.39 (br s, 0.4H), 7.22 – 6.95 (m, 3H), 6.59 (br s, 0.4H), 6.45 (br s, 0.6H), 5.13 (br s, 0.6 H), 4.95 (br s, 0.4H), 3.16 (br s, 1H), 2.86 – 2.20 (m, 8H), 2.04 (br s, 3H), 1.70 (br s, 1H), 1.30 (s, 9H), 1.11 (ddt, *J* = 13.7, 8.1, 5.6 Hz, 1H), 0.88 – -0.59 (m, 9H). **^{13}C NMR** (151 MHz, $CDCl_3$) δ 167.5, 140.0, 133.8 (br), 132.3 (br), 130.3, 126.6, 125.7, 78.6 (br), 67.2, 63.3, 54.8, 36.4 (br), 35.6, 34.8 (br), 27.3, 20.9, 15.0 (br), 13.9, 13.7, 7.0 (br), 5.0 (br), 4.4 (br), 2.5 (br), 1.8 (br). **IR** ν_{max} = 2369, 2952, 1615, 1473, 1442, 1401, 1333 cm^{-1}. **HRMS** (ESI^+) *m/z* calcd for $C_{25}H_{39}N_2O_2$ $[M+H]^+$ 399.3006, found 399.2987. $[\alpha]_D^{23}$ = +184 (c = 1 in $CHCl_3$). **HPLC**: *er* 94:6; CHIRALCEL® OD-H, hexane:IPA = 99:1, flow = 0.5 mL/min, λ = 230 nm, tR = 12.00 min (major), and 13.10 min (minor).

(*S*)-1-(*tert*-Butyl)-3-(dicyclopropylmethyl)-1-methyl-3-(1-(naphthalen-1-yl)ethyl)urea **9e**

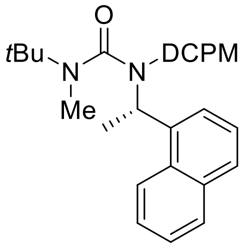

Synthesised according to general procedure 5 using 1-(*tert*-butyl)-3-(dicyclopropylmethyl)-1-methyl-3-(naphthalen-1-ylmethyl)urea **8e** (91 mg, 0.25 mmol). The crude urea was purified by silica gel flash column chromatography (petroleum ether + EtOAC 0-15%) to afford the title product as a colourless oil (71 mg, 75% yield).

1 mmol scale: The same general procedure was followed, using 1-(*tert*-butyl)-3-(dicyclopropylmethyl)-1-methyl-3-(naphthalen-1-ylmethyl)urea **8e** (364 mg, 1 mmol) affording the title product as a colourless oil (291 mg, 71% yield).

NMR reported as a rotameric mixture.

^{1}H NMR (400 MHz, $CDCl_3$) δ 8.32 (br s, 0.5H), 8.10 (br s, 0.5H), 7.90 – 7.54 (m, 3H), 7.54 – 7.35 (m, 3H), 5.88 (br s, 1H), 3.12 – 2.43 (m, 3.5 H), 2.06 (br s, 0.5H), 1.78 (d, *J* = 6.9 Hz, 3H), 1.36 (s, 9H), 1.13 (s, 1H), 0.78 – 0.35 (m, 2H), 0.35 – 0.08 (m, 4H), 0.07 – -0.06 (m, 1H), -0.13 – -0.31 (m, 1H), -0.48 (br s, 0.5H), -0.75 (br s, 0.5H). **^{13}C NMR** (101 MHz, $CDCl_3$) δ 165.6 (br), 139.6, 133.8, 132.0 (br), 128.8, 127.8 (br), 126.0, 125.4, 125.3, 125.2 (br), 124.1 (br), 65.6, 54.9 (br), 51.0 (br), 35.3 (br), 27.5, 19.9 (br), 14.8, 14.6, 5.2 (br), 3.0 (br). **IR** v_{max} = 2971, 1634, 1460, 1433, 1358, 1306 cm^{-1}. **HRMS** (ESI$^+$) *m/z* calcd for $C_{25}H_{35}N_2O$ $[M+H]^+$ 379.2744, found 379.2732. $[\alpha]_D^{23}$ = +120 (c = 1 in $CHCl_3$). **HPLC**: *er* 94:6; CHIRALCEL® OD-H, hexane:IPA = 99:1, flow = 1.0 mL/min, λ = 230 nm, tR = 8.02 min (minor), and 10.15 min (major).

(*S*)-1-(*tert*-Butyl)-3-(dicyclopropylmethyl)-1-methyl-3-(phenyl(trimethylsilyl)methyl)urea **11**

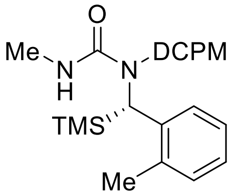

To a solution of (*R*)-*N*-(1-phenylethyl)-1-methylethylamine **10** (4 equiv.) in dry THF (0.1 M) under a nitrogen atmosphere at -78 °C, was added *n*-butyl lithium (4.4 equiv., 2.5 M in hexanes) dropwise. The resulting mixture was allowed to warm to room temperature over 15 minutes,

resulting in a clear yellow solution. This solution was then cooled back down to -78 °C, chlorotrimethylsilane (1.5 equiv., 0.375 mmol, 41 mg) was added and the mixture stirred for 5 min. A solution of 1-(dicyclopropylmethyl)-3-methyl-1-(2-methylbenzyl)urea (1 equiv., 0.25 mmol, 68 mg) in dry THF (0.5 mL) was added dropwise and the solution was stirred for 1.5 h while warming slowly to -50 °C. After another hour at -50 °C, MeOH was added to quench the reaction, the mixture allowed to warm to RT and concentrated under reduced pressure to yield the crude product. The crude urea was purified by silica gel flash column chromatography (petroleum ether + EtOAC 0-40%) to afford the title product as a colourless solid (64 mg, 74% yield).

m.p. = 103–106 °C. **^{1}H NMR** (400 MHz, $CDCl_3$) δ 7.55 (d, *J* = 7.8 Hz, 1H), 7.10 (td, *J* = 8.0, 7.1, 2.4 Hz, 1H), 7.05 – 6.96 (m, 2H), 4.41 (br s, 1H), 4.01 (s, 1H), 2.84 (d, *J* = 4.6 Hz, 3H), 2.30 (s, 3H), 2.24 (t, *J* = 7.6 Hz, 1H), 1.11 – 0.99 (m, 1H), 0.76 – 0.58 (m, 2H), 0.47 – 0.27 (m, 3H), 0.18 – 0.10 (m, 1H), 0.06 (s, 9H), 0.03 – -0.24 (m, 3H). **^{13}C NMR** (101 MHz, $CDCl_3$) δ 160.2, 142.5, 133.2 (br), 130.4, 129.9, 126.1, 125.3, 67.7, 51.1 (br), 28.3, 20.3, 14.3, 14.1, 5.5, 5.2, 3.0, 2.5, 1.1. **IR** ν_{max} = 3363, 3007, 2952, 1615, 1511, 1377, 1317, 1239 cm^{-1}. **HRMS** (ESI^+) *m*/*z* calcd for $C_{20}H_{33}N_2OSi$ $[M+H]^+$ 345.2357, found 345.2344. $[\alpha]_D^{23}$ = +92 (c = 1 in $CHCl_3$). **HPLC**: *er* 95:5; CHIRALCEL® OD-H, hexane:IPA = 99:1, flow = 1.0 mL/min, λ = 230 nm, tR = 6.32 min (major), and 6.95 min (minor).

1.4.2.1 Unsuccessful reactions

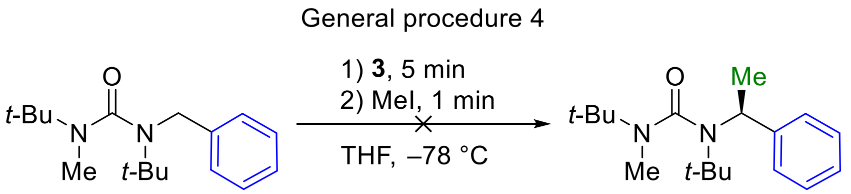

1c

no conversion of **1c** observed

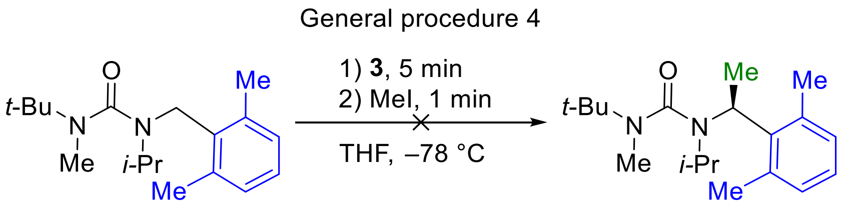

SI-2

no conversion of **SI-2** observed

1.4.3 Conversion to benzylamine derivatives

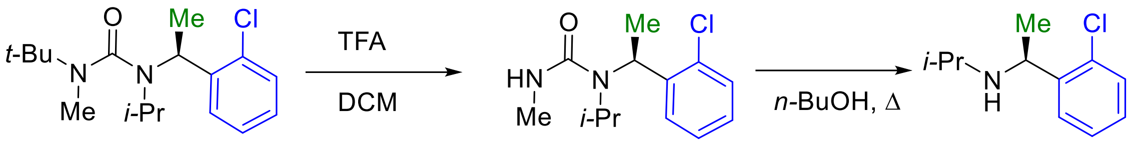

7g
er 83:17

SI-3, 97%
er 83:17

12, 67%
er 83:17

(*S*)-1-(1-(2-Chlorophenyl)ethyl)-1-isopropyl-3-methylurea **SI-3**

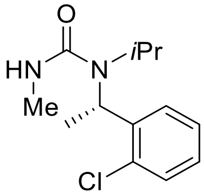

(*S*)-1-(*tert*-Butyl)-3-(1-(2-chlorophenyl)ethyl)-3-isopropyl-1-methylurea **7g** (1 equiv., 0.19 mmol, 58 mg) was dissolved in anhydrous DCM (0.7 mL) under a nitrogen atmosphere. Triflouroacetic acid (2 equiv, 0.38 mmol, 0.029 mL) was added and the solution stirred for 19 h at RT. Saturated, aqueous $NaHCO_3$ (2 mL) was added, and the layers separated. The aqueous layer was extracted with DCM (2 × 2 mL) and the combined organic layers were dried over $MgSO_4$ and then evaporated under reduced pressure to afford the title product as a colourless oil (46 mg, 97% yield).

^{1}H NMR (400 MHz, $CDCl_3$) δ 7.47 (dd, *J* = 7.6, 2.0 Hz, 1H), 7.34 (dd, *J* = 7.8, 1.7 Hz, 1H), 7.23 (dtd, *J* = 21.5, 7.5, 1.7 Hz, 2H), 5.34 (q, *J* = 7.0 Hz, 1H), 4.31 (q, *J* = 4.1 Hz, 1H), 3.41 (sept, *J* = 7.0 Hz, 1H), 2.84 (d, *J* = 4.6 Hz, 3H), 1.55 (d, *J* = 6.9 Hz, 3H), 1.30 (d, *J* = 6.9 Hz, 3H), 0.90 (d, *J* = 7.0 Hz, 3H). **^{13}C NMR** (101 MHz, $CDCl_3$) δ 158.4, 139.6, 134.4, 129.9, 128.7, 128.7, 126.8, 51.8, 46.0, 27.6, 21.8, 21.2, 17.9. **IR** v_{max} = 3361, 2967, 1622, 1520, 1330 cm^{-1}. **HRMS** (ESI$^+$) *m*/*z* calcd for $C_{13}H_{20}ClN_2O$ $[M+H]^+$ 255.1259, found 255.1247. $[\alpha]_D^{26}$ = +4 (c = 1 in $CHCl_3$). **HPLC**: *er* 83:17; CHIRALPAK® IJ-3, MeCN:H_2O (0.5% Formic Acid) gradient: 0 min: 30:70; 1 min: 30:70; 16 min: 95:5; flow = 1.0 mL/min, λ = 254 nm, tR = 8.16 min (major), and 8.55 min (minor).

(*S*)-*N*-(1-(2-Chlorophenyl)ethyl)propan-2-amine **12**

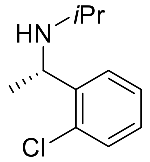

(*S*)-1-(1-(2-Chlorophenyl)ethyl)-1-isopropyl-3-methylurea **SI-3** (1 equiv., 0.165 mmol, 42 mg) was dissolved in *n*-butanol (0.83 mL) under a nitrogen atmosphere and heated to 115 °C for 17 h. The reaction mixture was concentrated under reduced pressure and purified by silica gel flash column chromatography (petroleum ether + EtOAC 0-40%) to afford the title product as a colourless oil (24 mg mixture of **12** and EtOAC containing 22 mg product **12**, 67 % yield). Due to the volatility of the product, EtOAc was not removed completely and the amount of product (90.5 wt% **12**) was calculated from the ratio of integrals (1:0.24) in ^{1}H-NMR and molecular weights.

^{1}H NMR (600 MHz, $CDCl_3$) δ 7.43 (dd, *J* = 7.8, 1.9 Hz, 1H), 7.32 (dd, *J* = 7.9, 1.4 Hz, 1H), 7.28 – 7.23 (m, 1H), 7.15 (td, *J* = 7.5, 1.8 Hz, 1H), 4.40 (q, *J* = 6.6 Hz, 1H), 2.60 (sept, *J* = 6.3 Hz, 1H), 1.32 (d, *J* = 6.6 Hz, 3H), 1.03 (d, *J* = 6.1 Hz, 3H), 1.01 (d, *J* = 6.4 Hz, 3H). **^{13}C NMR** (151 MHz, $CDCl_3$) δ 143.2, 133.2, 129.7, 127.8, 127.5, 127.2, 51.5, 45.9, 24.2, 23.4, 22.4. **IR** ν_{max} = 2962, 1470, 1369 cm^{-1}. **HRMS** (ESI$^+$) *m*/*z* calcd for $C_{11}H_{17}ClN$ [M+H]$^+$ 198.1044, found 198.1036. $[\alpha]_D^{23}$ = -36 (c = 1 in $CHCl_3$).

Chiral HPLC analysis was carried out on the *N*-isopropyl urea derivative which was prepared by mixing equimolar amounts of amine **12** and isopropyl isocyanate in anhydrous DCM (0.1 M), stirring for 1 h at RT and evaporating all volatiles. Previous reports[1, 3] have shown that reaction of an amine with an isocyanate does not affect the chiral center.

HPLC: *er* 83:17; CHIRALPAK® IJ-3, MeCN:H_2O (0.5% Formic Acid) gradient: 0 min: 30:70; 1 min: 30:70; 16 min: 95:5; flow = 1.0 mL/min, λ = 254 nm, tR = 10.32 min (major), and 11.60 min (minor).

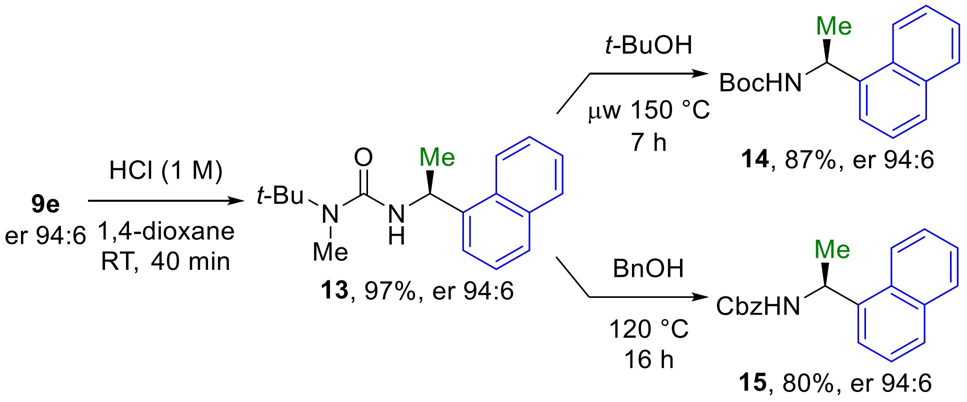

(*S*)-1-(*tert*-Butyl)-1-methyl-3-(1-(naphthalen-1-yl)ethyl)urea **13**

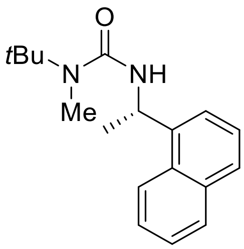

(S)-1-(*tert*-Butyl)-3-(dicyclopropylmethyl)-1-methyl-3-(1-(naphthalen-1-yl)ethyl)urea **9e** (1 equiv., 0.66 mmol, 0.250 g) was dissolved in anhydrous 1,4-dioxane (6 mL) under a nitrogen atmosphere. HCl in 1,4-dioxane (4 M, 2 mL) was added and the solution stirred for 40 min at RT. 1 M NaOH (aqu., 20 mL) and DCM (20 mL) were added, and the layers separated. The aqueous layer was extracted with DCM (2 × 20 mL) and the combined organic layers were dried over $MgSO_4$ and then evaporated under reduced pressure. Trituration with hexane afforded the title product as a colourless solid (0.183 g, 97% yield).

m.p. = 97–99 °C. **^{1}H NMR** (400 MHz, $CDCl_3$) δ^{1}H NMR (400 MHz, Chloroform-*d*) δ 8.19 (d, *J* = 8.5 Hz, 1H), 7.85 (dd, *J* = 7.8, 1.7 Hz, 1H), 7.77 (d, *J* = 8.1 Hz, 1H), 7.59 – 7.40 (m, 4H), 5.80 (dq, *J* = 6.9, 6.9 Hz, 1H), 4.55 (d, *J* = 7.1 Hz, 1H), 2.78 (s, 3H), 1.65 (d, *J* = 6.8 Hz, 3H), 1.40 (s, 9H). **^{13}C NMR** (101 MHz, $CDCl_3$) δ^{13}C NMR (101 MHz, $CDCl_3$) δ 158.5, 140.2, 134.1, 131.3, 128.8, 128.0, 126.4, 125.8, 125.3, 124.0, 122.3, 55.7, 45.9, 31.9, 29.1, 22.0. **IR** ν_{max} = 3335, 2979, 1617 ,1520, 1343 cm^{-1}. **HRMS** (ESI$^+$) *m*/*z* calcd for $C_{18}H_{25}N_2O$ [M+H]$^+$ 285.1961, found 285.1953. $[\alpha]_D^{23}$ = +28 (c = 1 in $CHCl_3$). **HPLC**: *er* 94:6; CHIRALCEL® OD-H, hexane:IPA = 90:10, flow = 1.0 mL/min, λ = 254 nm, tR = 5.56 min (minor), and 6.42 min (major).

tert-Butyl (*S*)-(1-(naphthalen-1-yl)ethyl)carbamate **14**

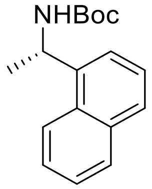

(S)-1-(*tert*-Butyl)-1-methyl-3-(1-(naphthalen-1-yl)ethyl)urea **13** (1 equiv., 0.2 mmol, 57 mg) was dissolved in *tert*-butanol (1 mL) under a nitrogen atmosphere and heated under microwave irradiation at 150 °C for 7 h. The reaction mixture was concentrated under reduced pressure and purified by silica gel flash column chromatography (petroleum ether + EtOAC 0-20%) to afford the title product as a colourless solid (47 mg, 87% yield).

m.p. = 106–108 °C. **^{1}H NMR** (400 MHz, $CDCl_3$) δ^{1}H NMR (400 MHz, Chloroform-*d*) δ 8.15 (d, *J* = 8.4 Hz, 1H), 7.87 (dd, *J* = 8.1, 1.6 Hz, 1H), 7.78 (d, *J* = 8.0 Hz, 1H), 7.59 – 7.41 (m, 4H), 5.75 – 5.47 (m, 1H), 4.90 (br s, 1H), 1.63 (d, *J* = 6.8 Hz, 3H), 1.45 (s, 9H). **^{13}C NMR** (101 MHz, $CDCl_3$) δ^{13}C NMR (101 MHz, $CDCl_3$) δ 155.2, 139.5, 134.1, 131.0, 128.9, 128.1, 126.4, 125.8, 125.4, 123.5, 122.2, 79.6, 46.3, 28.5, 22.0. $[\alpha]_D^{23}$ = -12 (c = 1 in $CHCl_3$); lit.[4] for *R*-enantiomer: $[\alpha]_D^{25}$ = +10.7 (c = 1 in $CHCl_3$, 95% ee). **HPLC**: *er* 94:6; CHIRALPAK® IA, hexane:IPA = 90:10, flow = 0.5 mL/min, λ = 254 nm, tR = 9.73 min (minor), and 11.35 min (major).
All data is in accordance with literature reports.[4]

Benzyl (*S*)-(1-(naphthalen-1-yl)ethyl)carbamate **15**

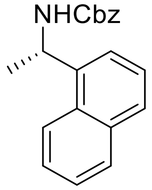

(*S*)-1-(*tert*-Butyl)-1-methyl-3-(1-(naphthalen-1-yl)ethyl)urea **13** (1 equiv., 0.2 mmol, 57 mg) was dissolved in benzyl alcohol (1 mL) under a nitrogen atmosphere and stirred at 120 °C for 16 h. The reaction mixture was concentrated under reduced pressure and purified by silica gel flash column chromatography (petroleum ether + EtOAC 0-20%) to afford the title product as a colourless solid (49 mg, 80% yield).

m.p. = 108–110 °C. **^{1}H NMR** (400 MHz, $CDCl_3$) δ 8.20 – 7.97 (m, 1H), 7.92 – 7.83 (m, 1H), 7.79 (dt, *J* = 8.0, 1.2 Hz, 1H), 7.61 – 7.42 (m, 4H), 7.42 – 6.93 (m, 5H), 5.70 (dq, *J* = 7.3 Hz, 1H), 5.27 – 4.99 (m, 3H), 1.67 (d, *J* = 6.8 Hz, 3H). **^{13}C NMR** (101 MHz, $CDCl_3$) δ^{13}C NMR (101 MHz, $CDCl_3$) δ 155.6, 138.9, 136.7, 134.1, 131.0, 129.0, 128.6, 128.4, 128.2, 126.6, 125.9, 125.4,

123.4, 122.4, 66.9, 46.9, 21.8. $[\alpha]_D^{23}$ = +8 (c = 1 in $CHCl_3$). **HPLC**: *er* 94:6; CHIRALPAK® IA, hexane:IPA = 90:10, flow = 0.5 mL/min, λ = 254 nm, tR = 18.36 min (minor), and 20.54 min (major).

All data is in accordance with literature reports.[5]

2 Analytical data and spectra

2.1 ^{1}H and ^{13}C NMR spectra

2.1.1 Starting materials

1-Benzyl-3-(*tert*-butyl)-1-ethyl-3-methylurea **1a**

^{1}H NMR (400 MHz, $CDCl_3$)

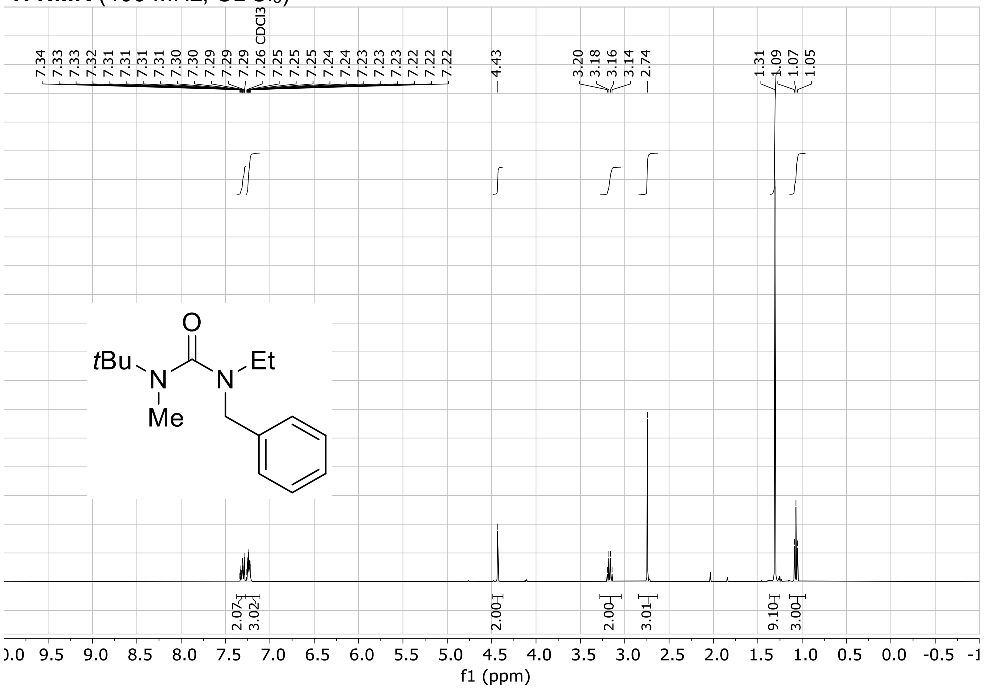

^{13}C NMR (101 MHz, $CDCl_3$)

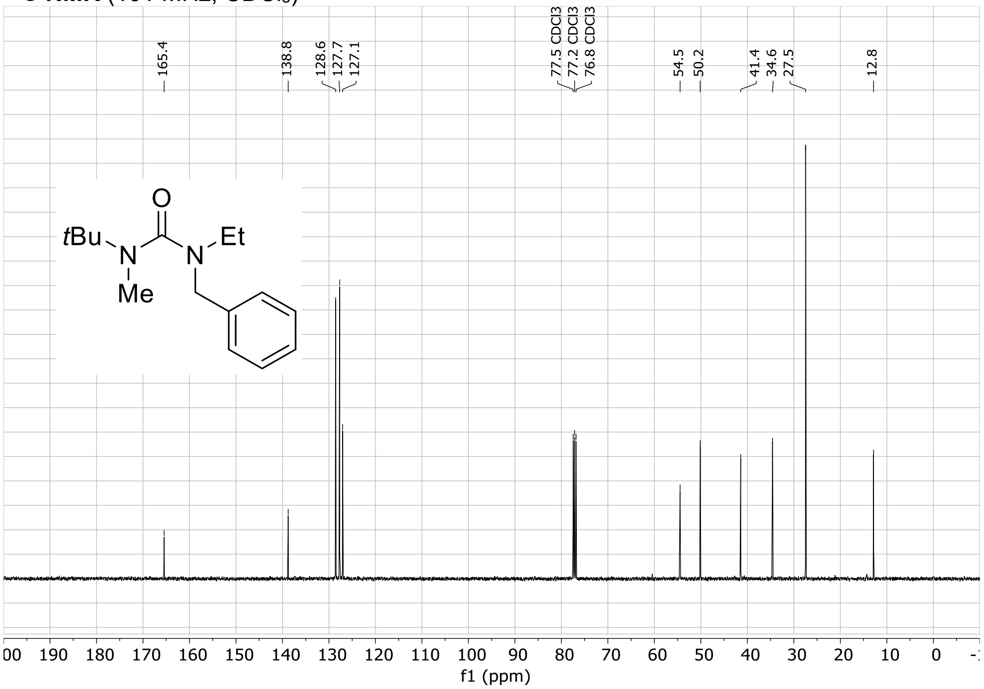

1-Benzyl-3-(*tert*-butyl)-1-isopropyl-3-methylurea **1b = 6a**

^{1}H NMR (400 MHz, $CDCl_3$)

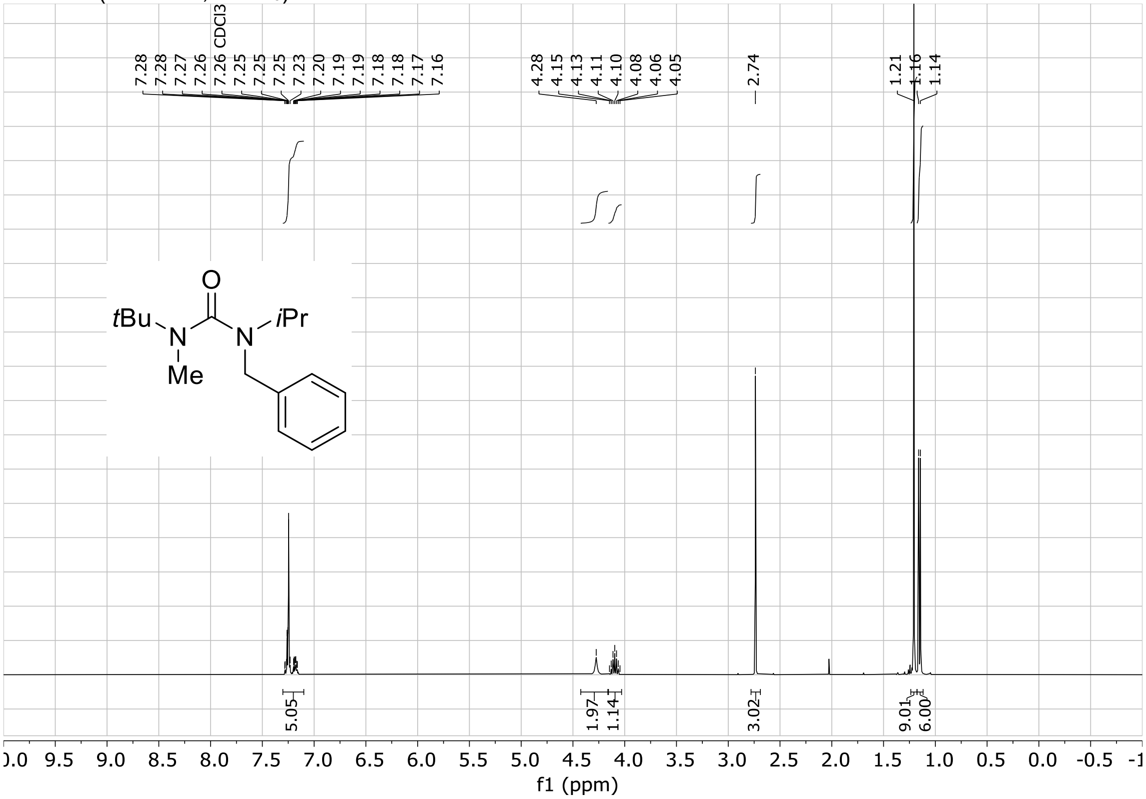

^{13}C NMR (101 MHz, $CDCl_3$)

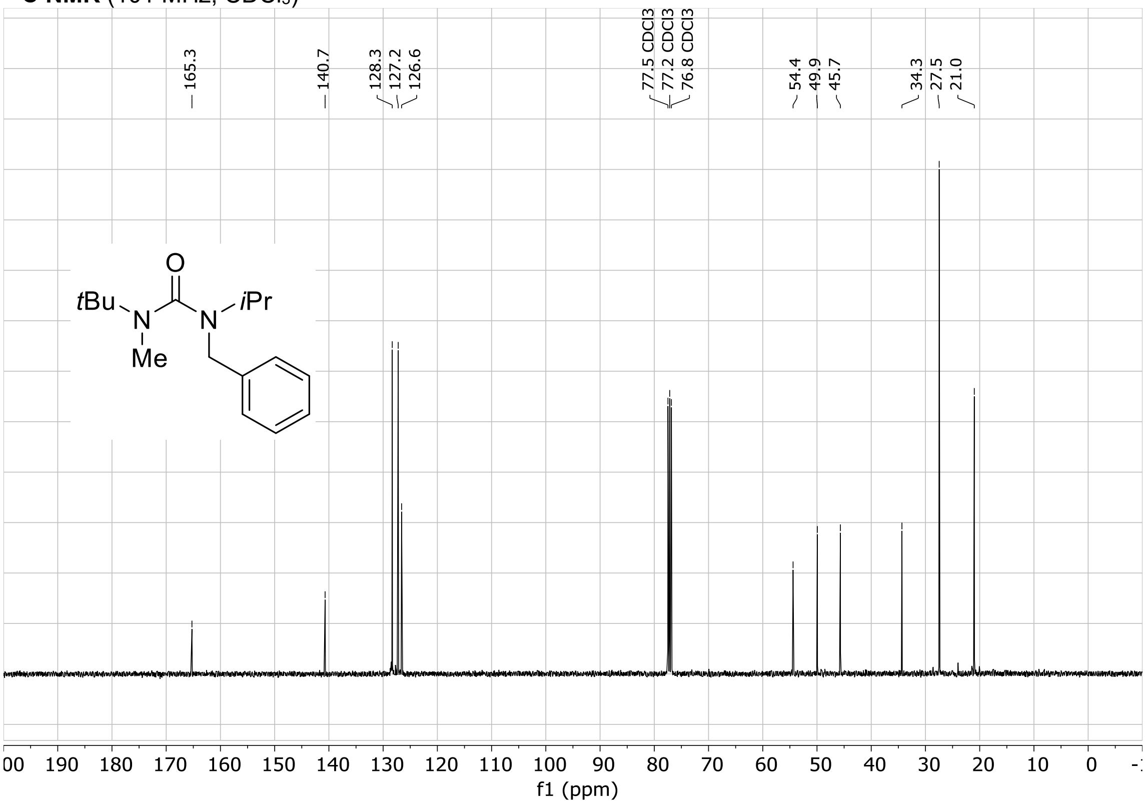

1-Benzyl-1,3-di-*tert*-butyl-3-methylurea **1c**

^{1}H NMR (400 MHz, $CDCl_3$)

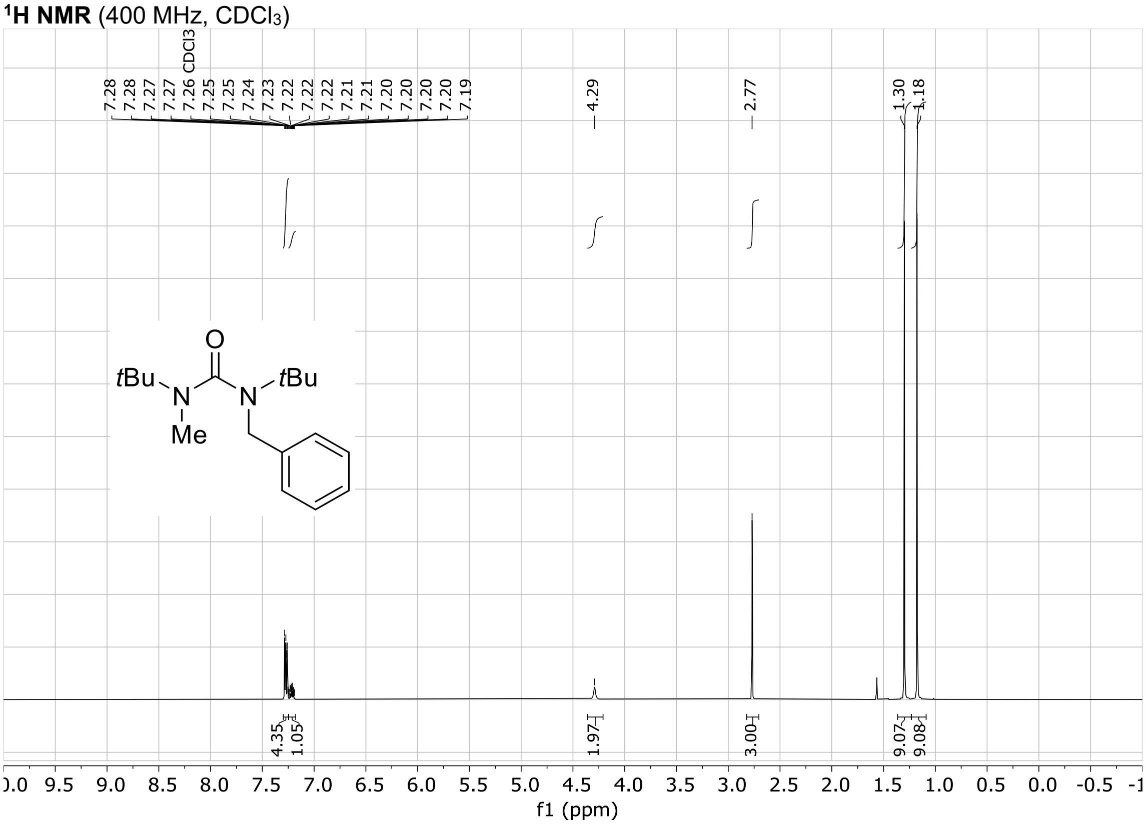

^{13}C NMR (101 MHz, $CDCl_3$)

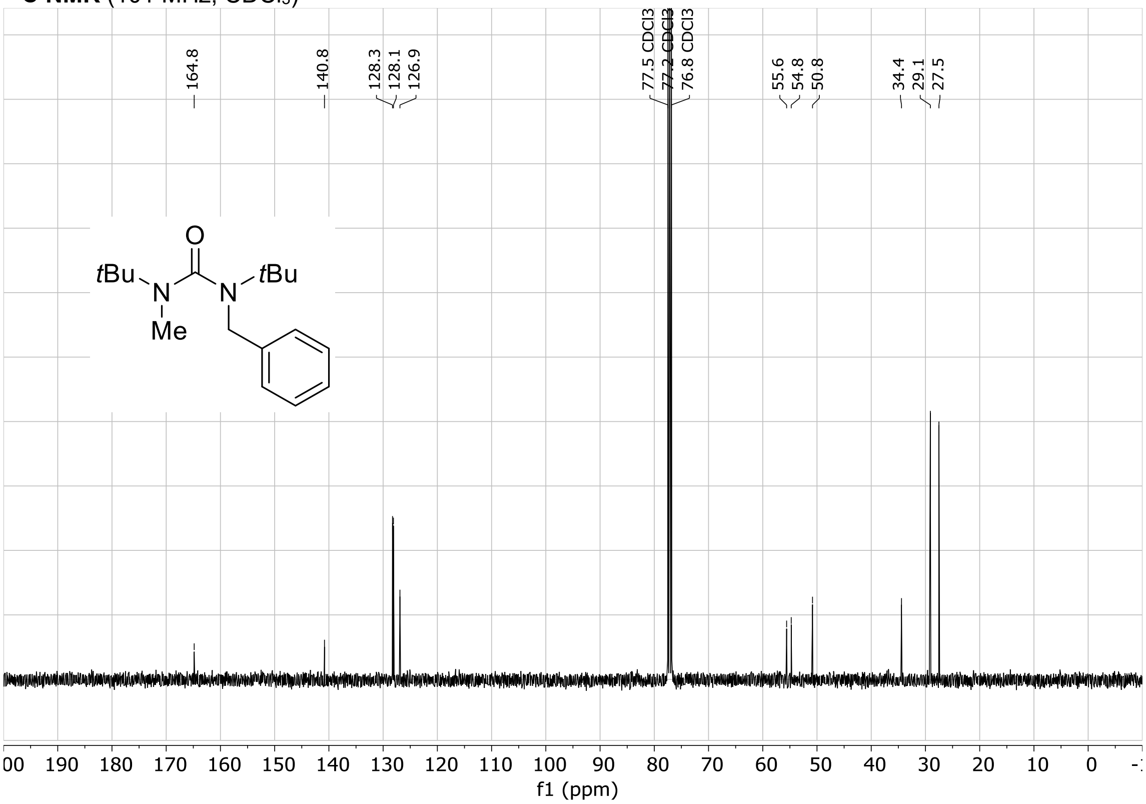

1-Benzyl-3-ethyl-1,3-diisopropylurea **1d**

^{1}**H NMR** (400 MHz, CDCl$_3$)

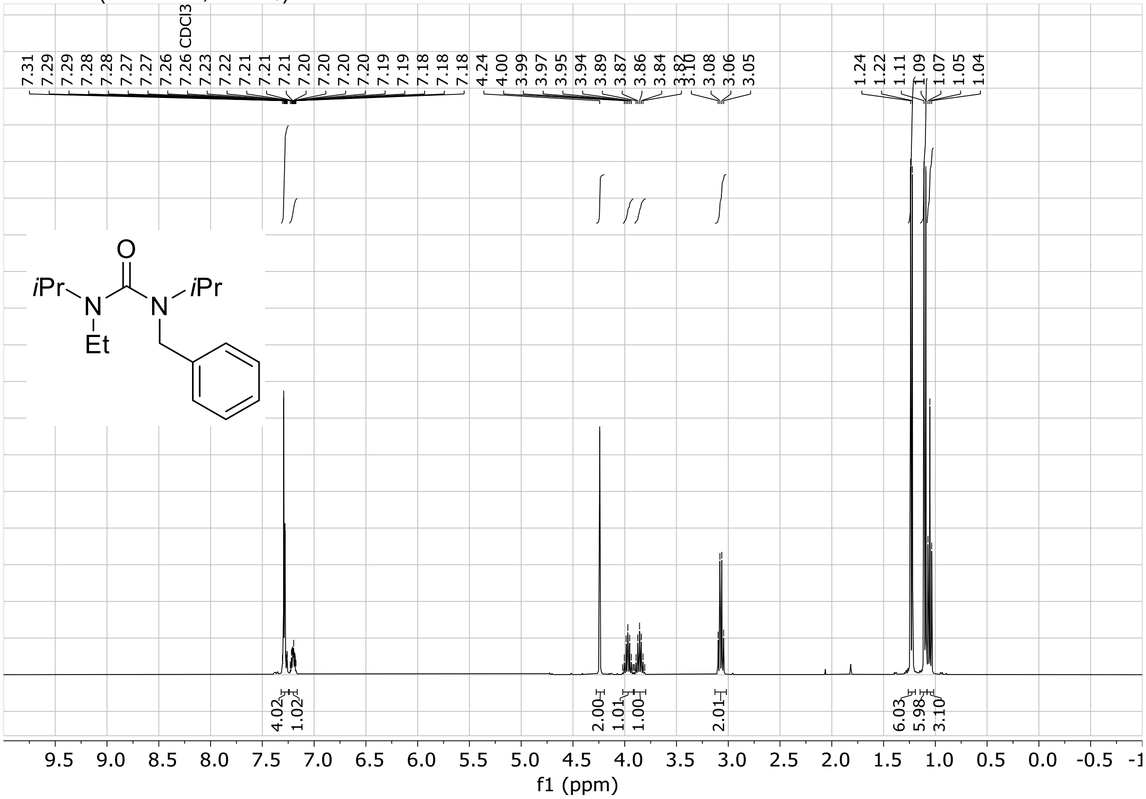

^{13}**C NMR** (101 MHz, CDCl$_3$)

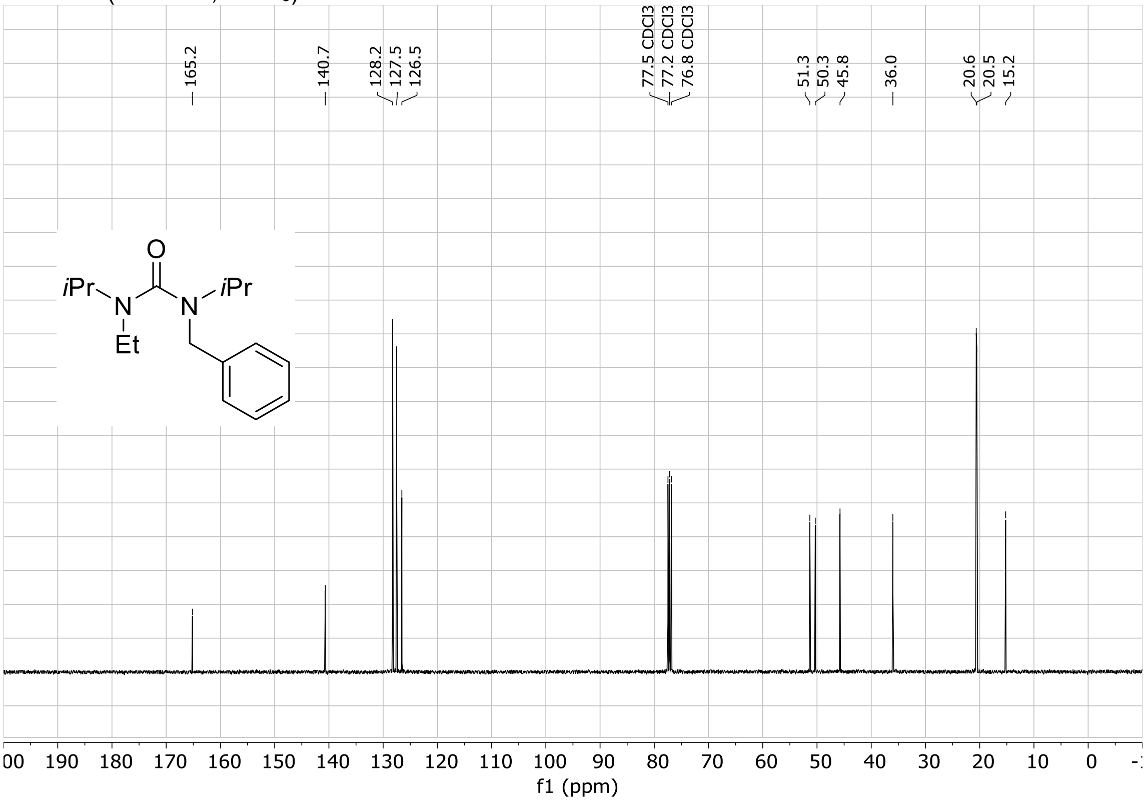

1-Benzyl-3-(*tert*-butyl)-3-ethyl-1-isopropylurea **1e**

^{1}H NMR (400 MHz, $CDCl_3$)

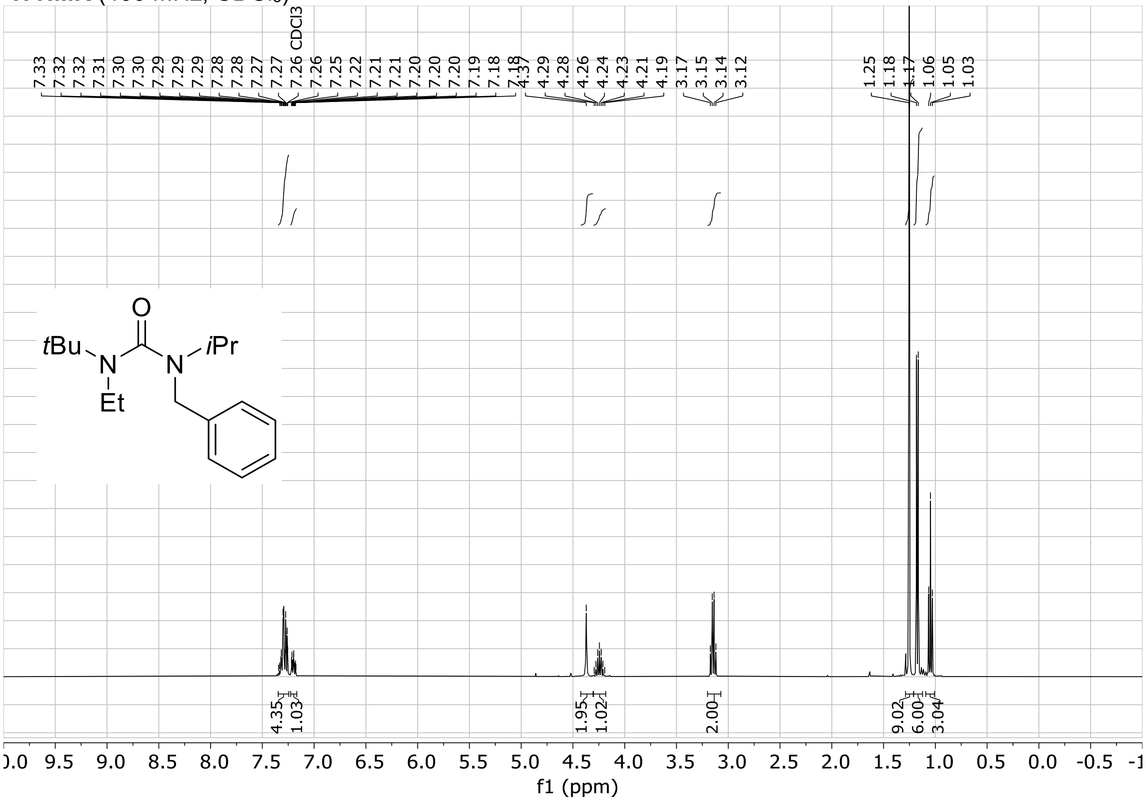

^{13}C NMR (101 MHz, $CDCl_3$)

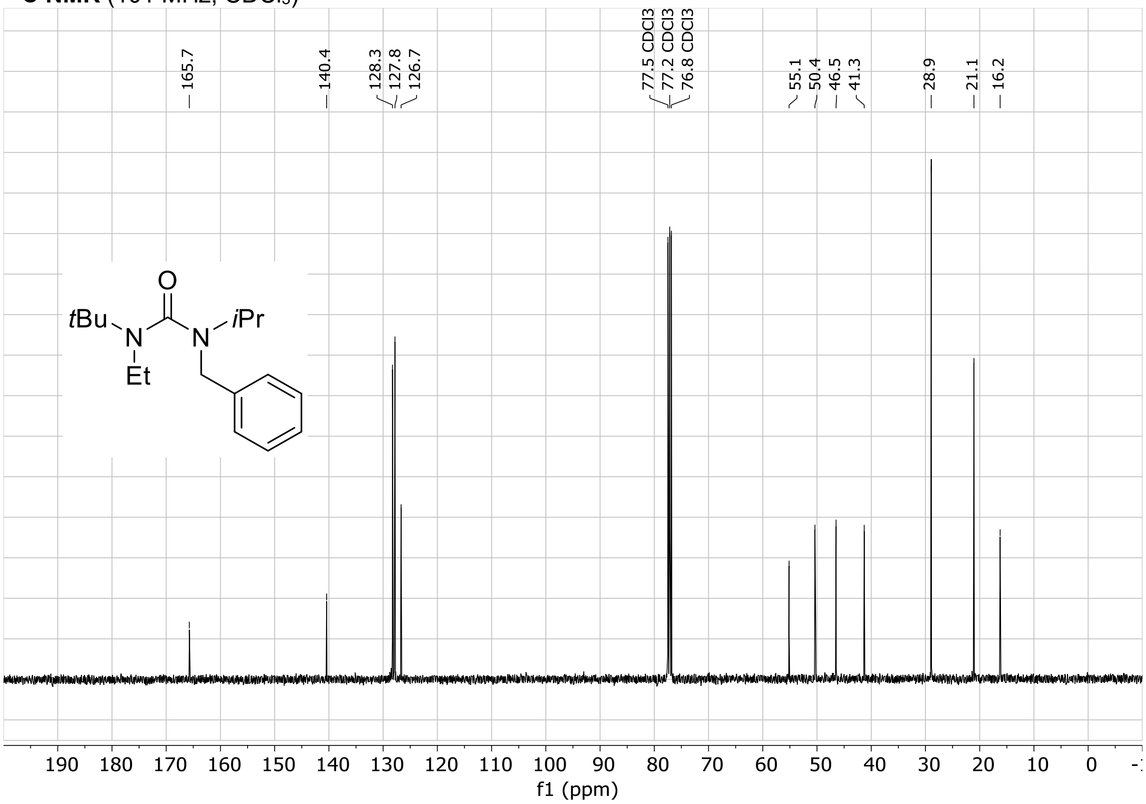

1-Benzyl-3-(*tert*-butyl)-1-cyclohexyl-3-methylurea **6b**

^{1}H NMR (400 MHz, $CDCl_3$)

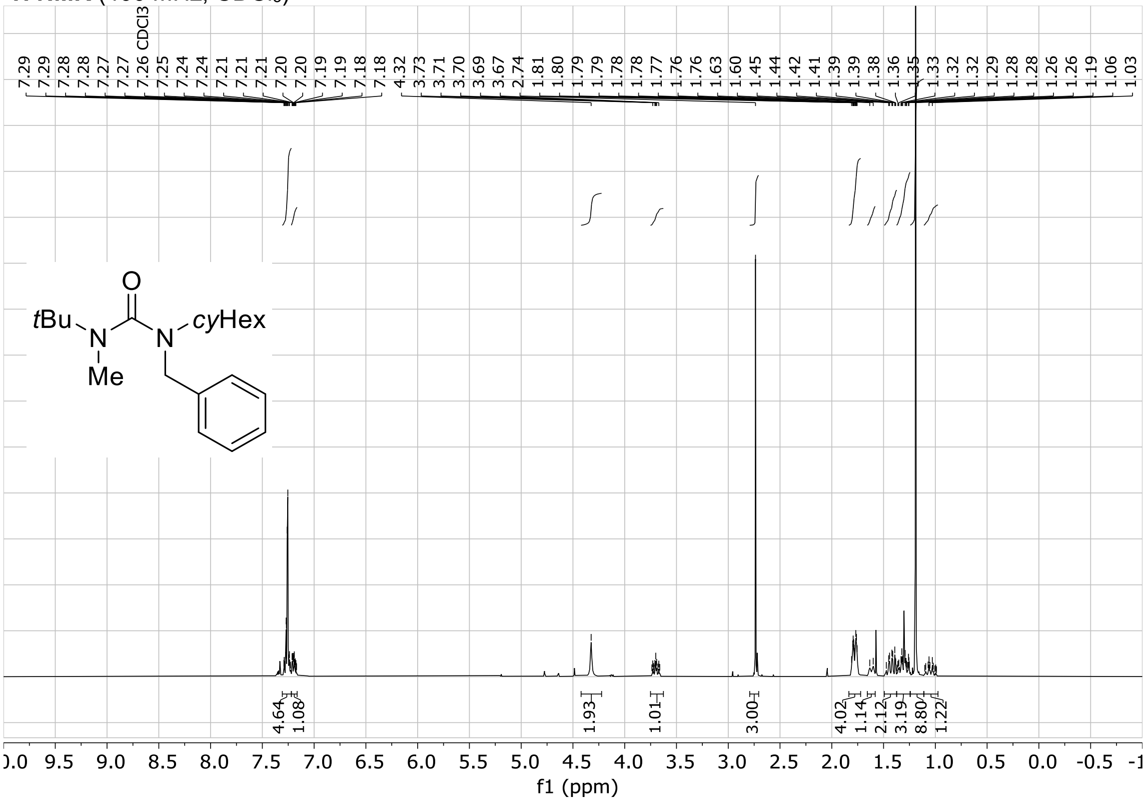

^{13}C NMR (101 MHz, $CDCl_3$)

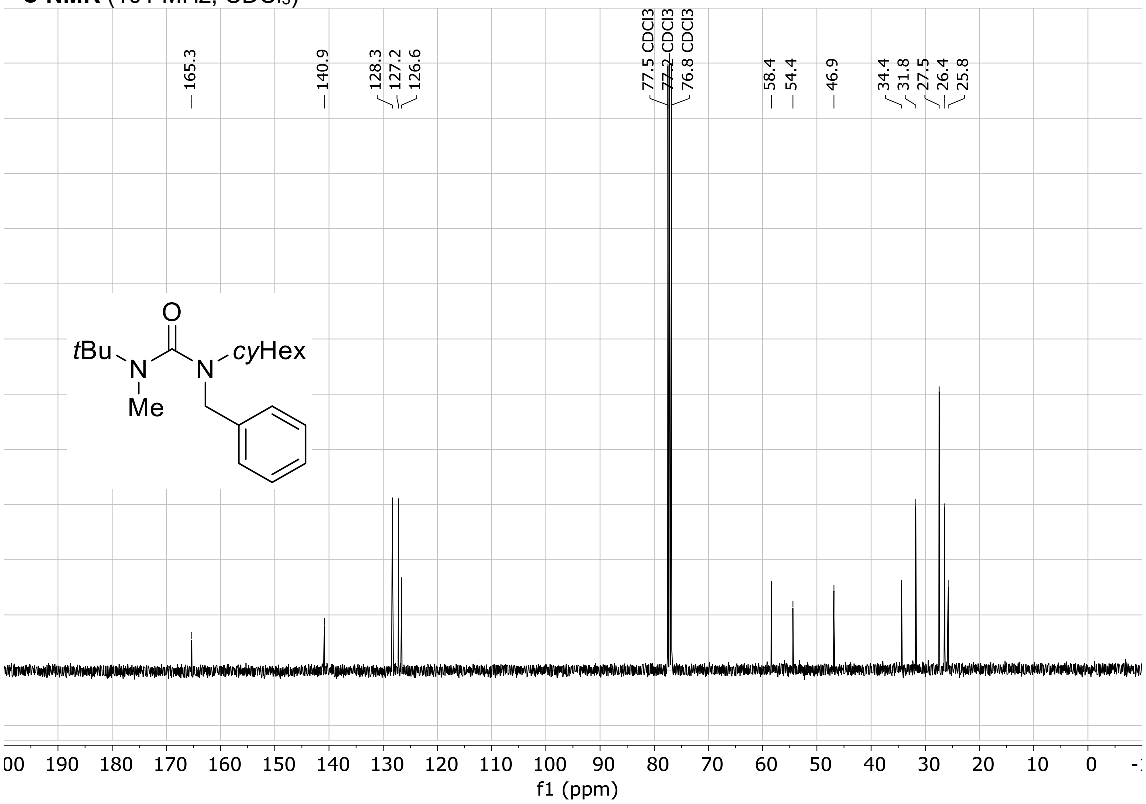

1-(*tert*-Butyl)-3-isopropyl-1-methyl-3-(2-methylbenzyl)urea **6c**

^{1}H NMR (400 MHz, $CDCl_3$)

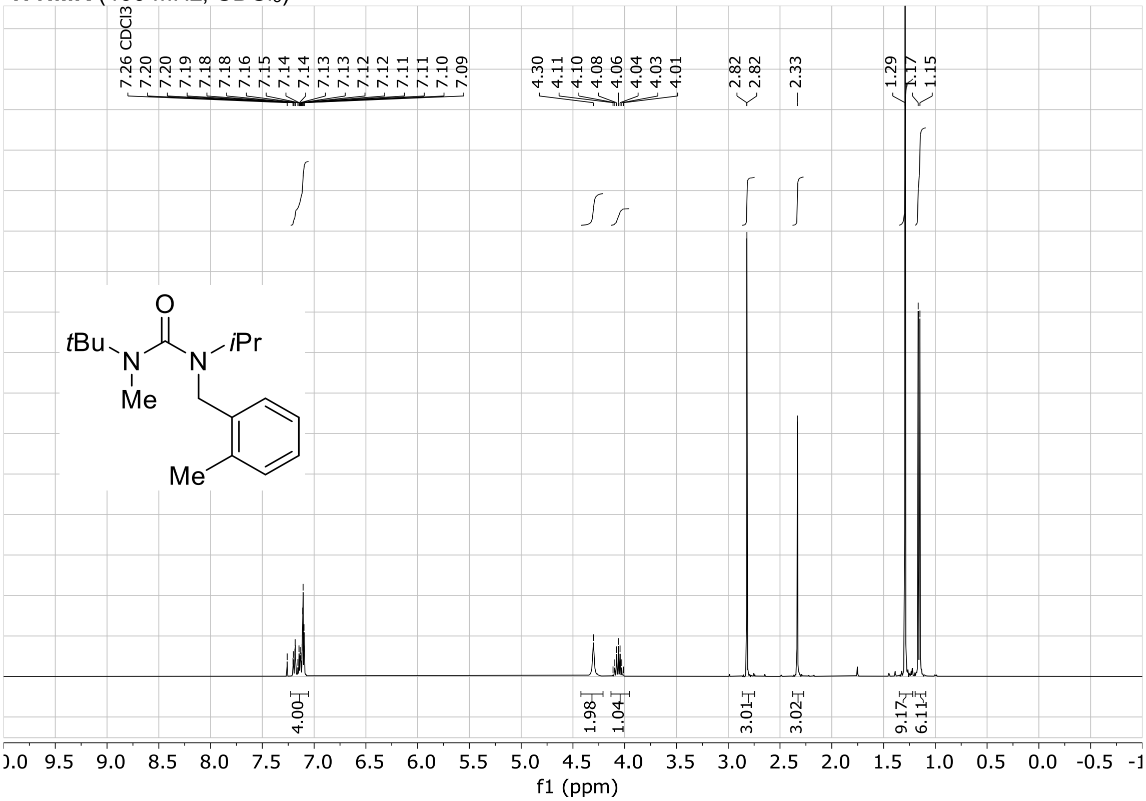

^{13}C NMR (101 MHz, $CDCl_3$)

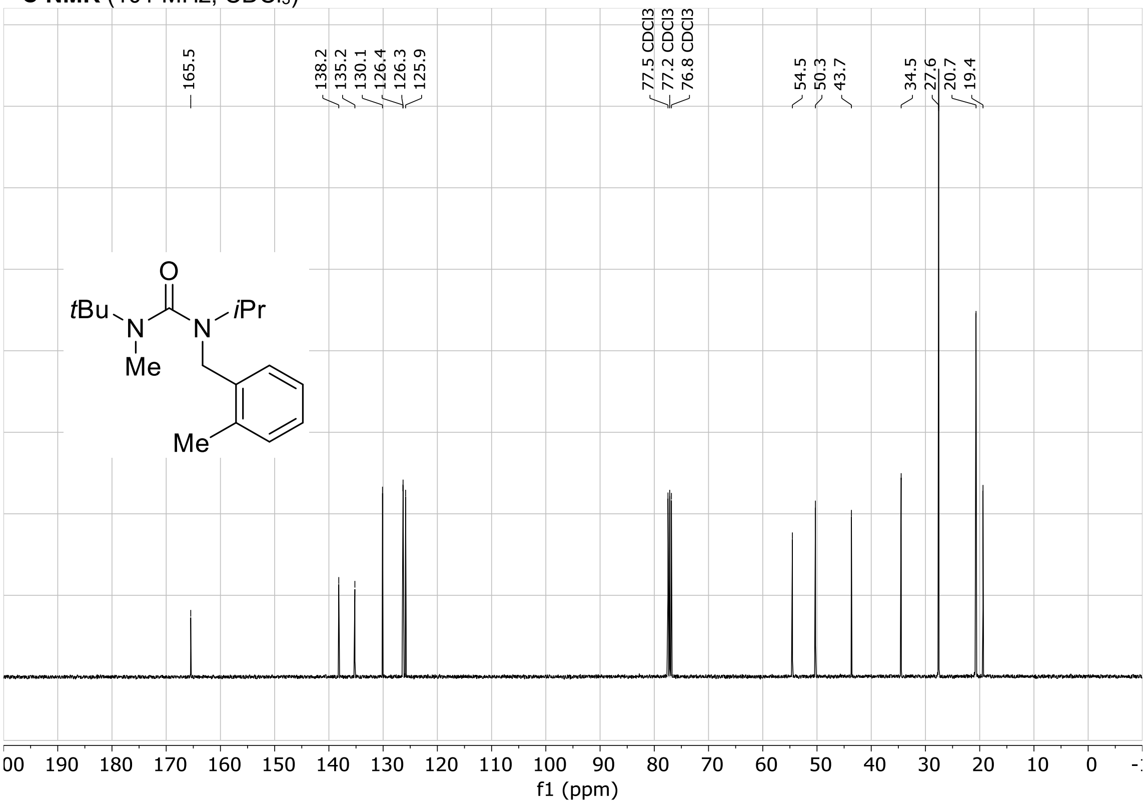

1-(*tert*-Butyl)-3-(4-fluoro-2-methylbenzyl)-3-isopropyl-1-methylurea **6d**

^{1}H NMR (400 MHz, $CDCl_3$)

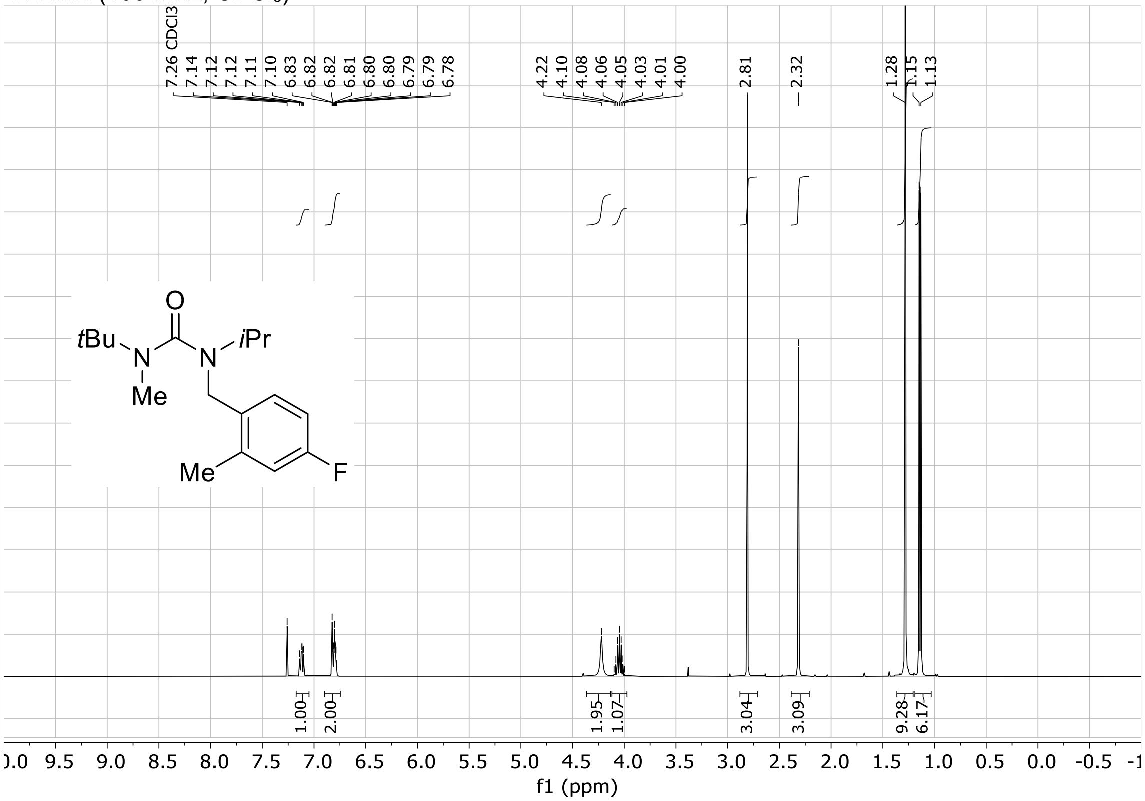

^{13}C NMR (101 MHz, $CDCl_3$)

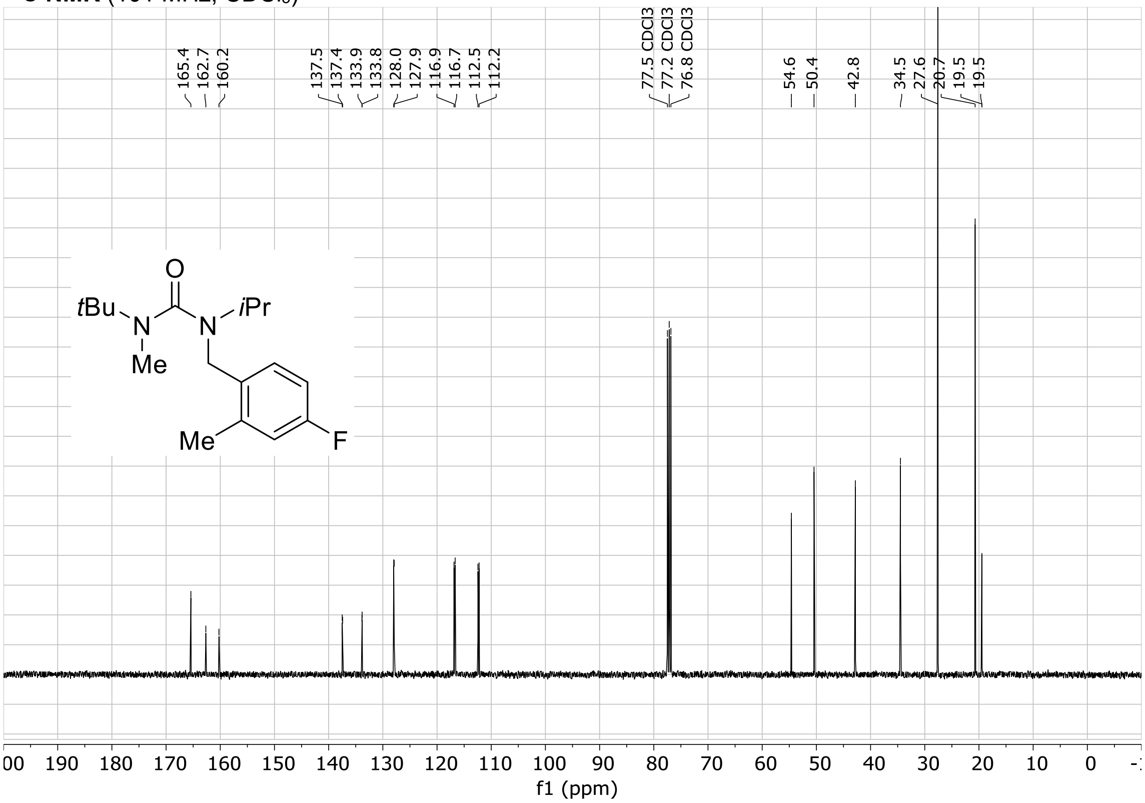

19F NMR (377 MHz, $CDCl_3$)

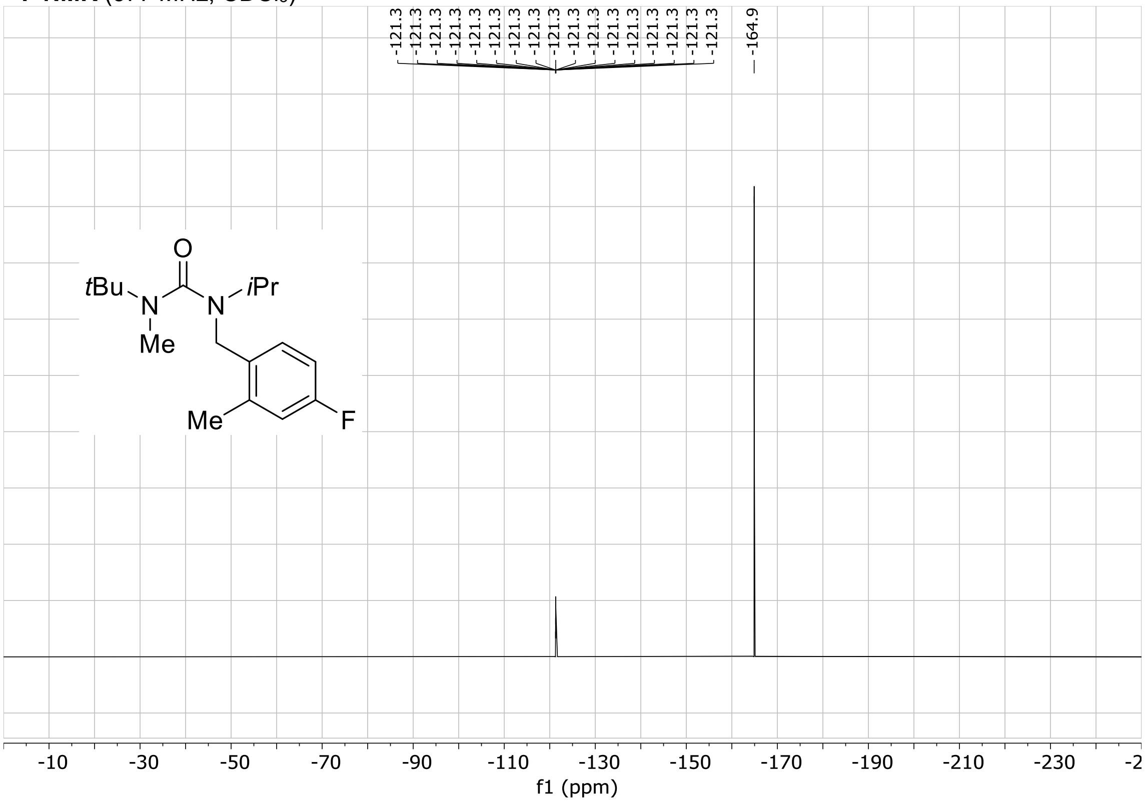

1-(*tert*-Butyl)-3-isopropyl-1-methyl-3-(4-methylbenzyl)urea **6e**

^{1}H NMR (400 MHz, $CDCl_3$)

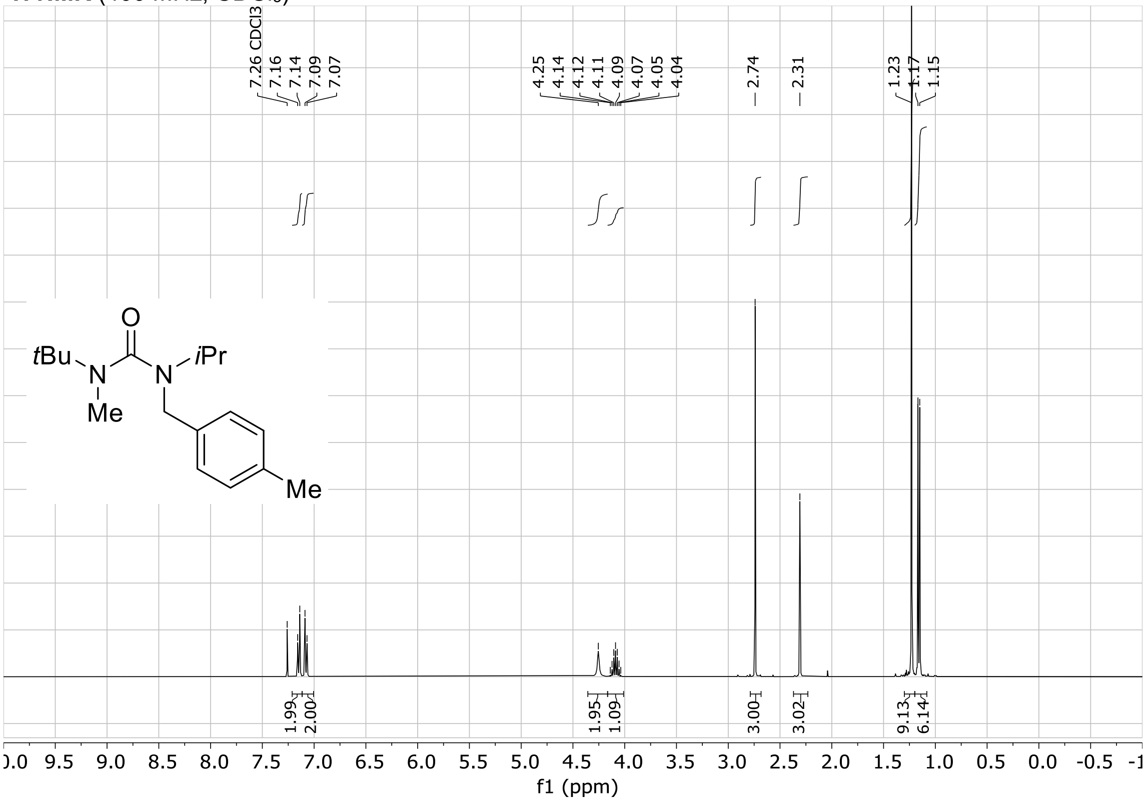

^{13}C NMR (101 MHz, $CDCl_3$)

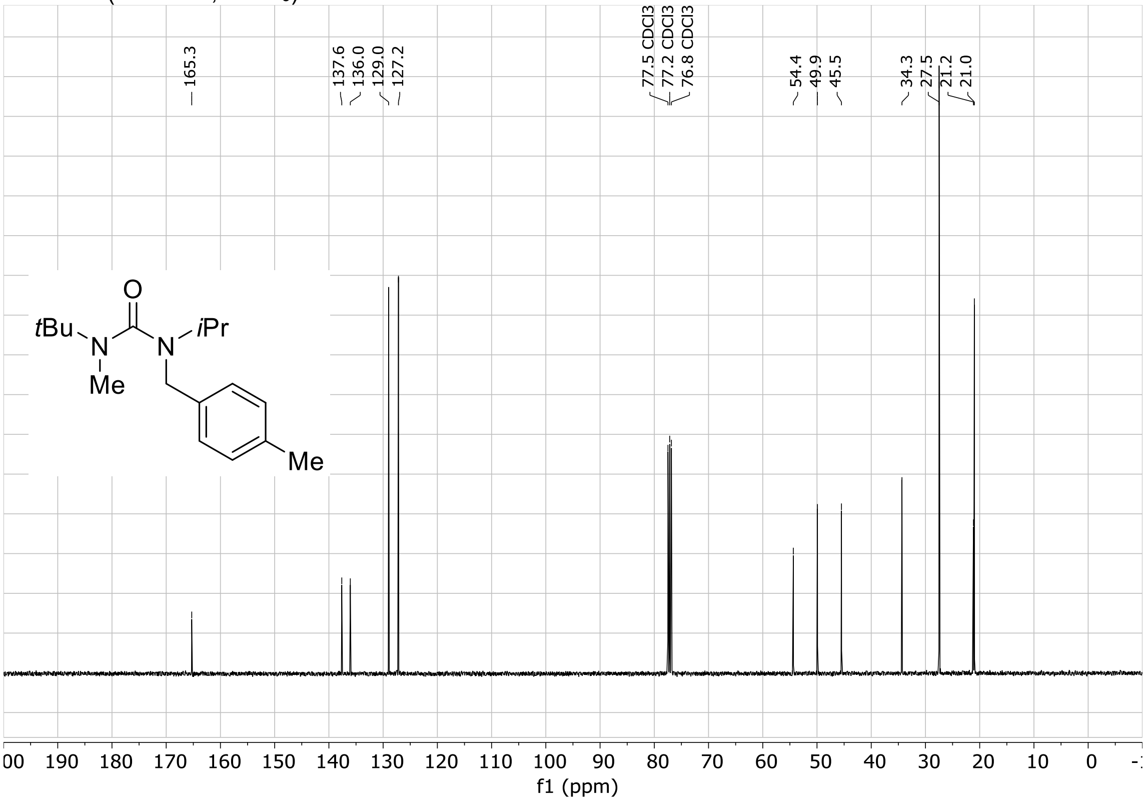

1-(*tert*-Butyl)-3-isopropyl-1-methyl-3-(3-methylbenzyl)urea **6f**

^{1}H NMR (400 MHz, $CDCl_3$)

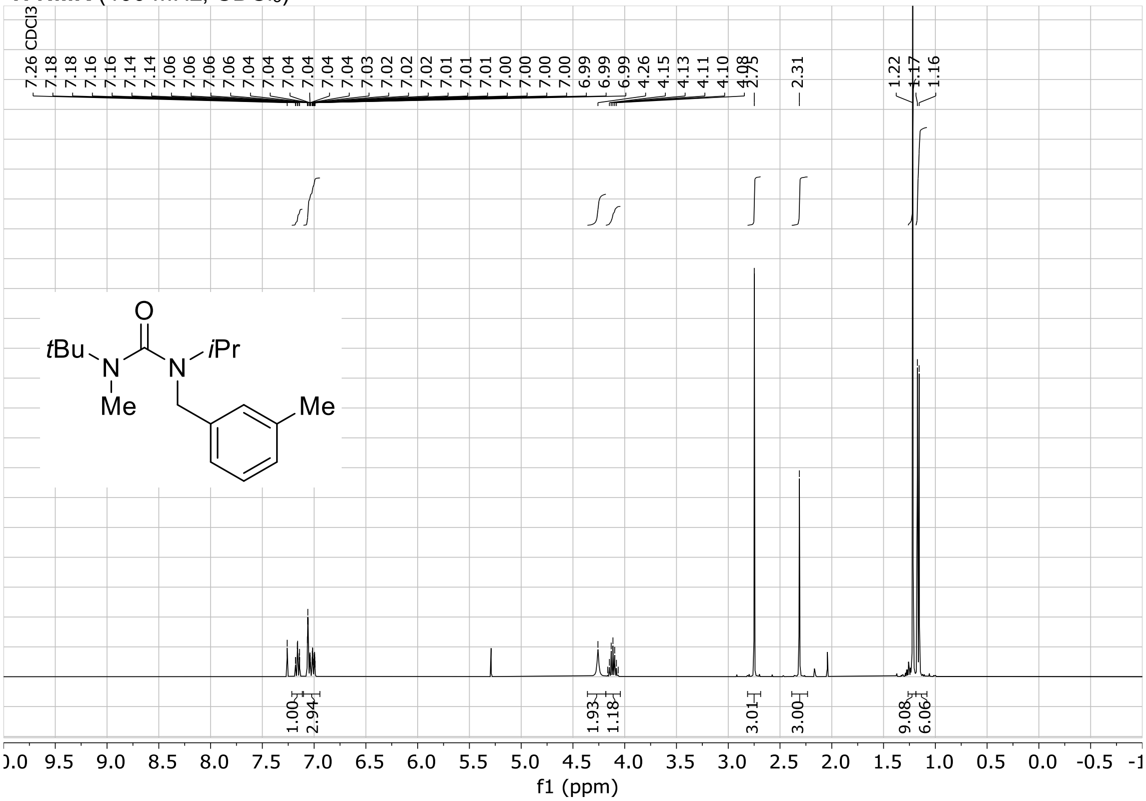

^{13}C NMR (101 MHz, $CDCl_3$)

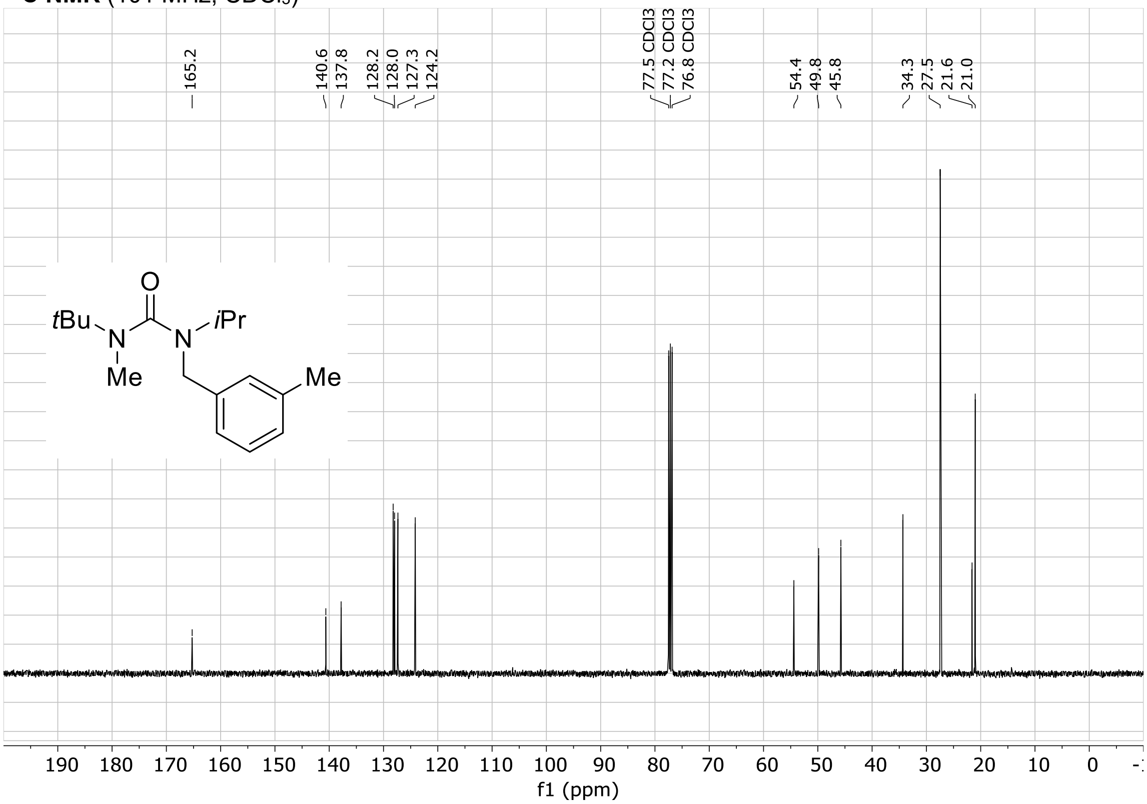

1-(*tert*-Butyl)-3-(2-chlorobenzyl)-3-isopropyl-1-methylurea **6g**

^{1}H NMR (400 MHz, $CDCl_3$)

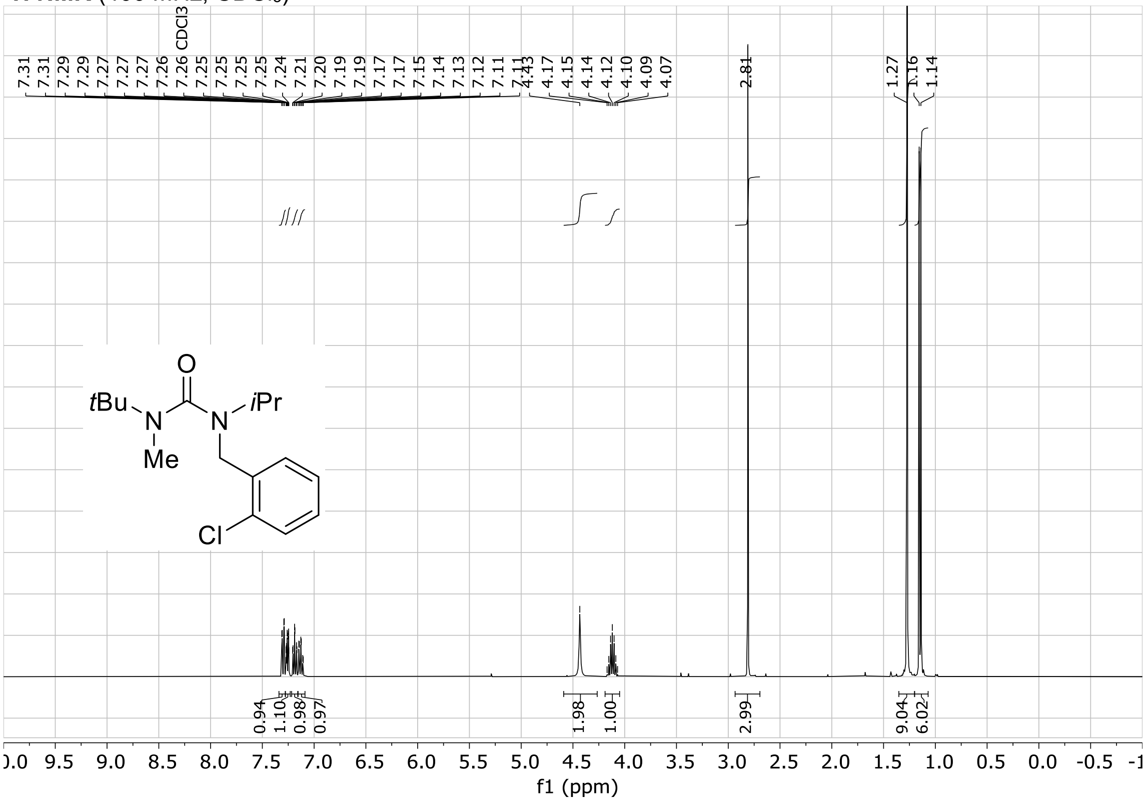

^{13}C NMR (101 MHz, $CDCl_3$)

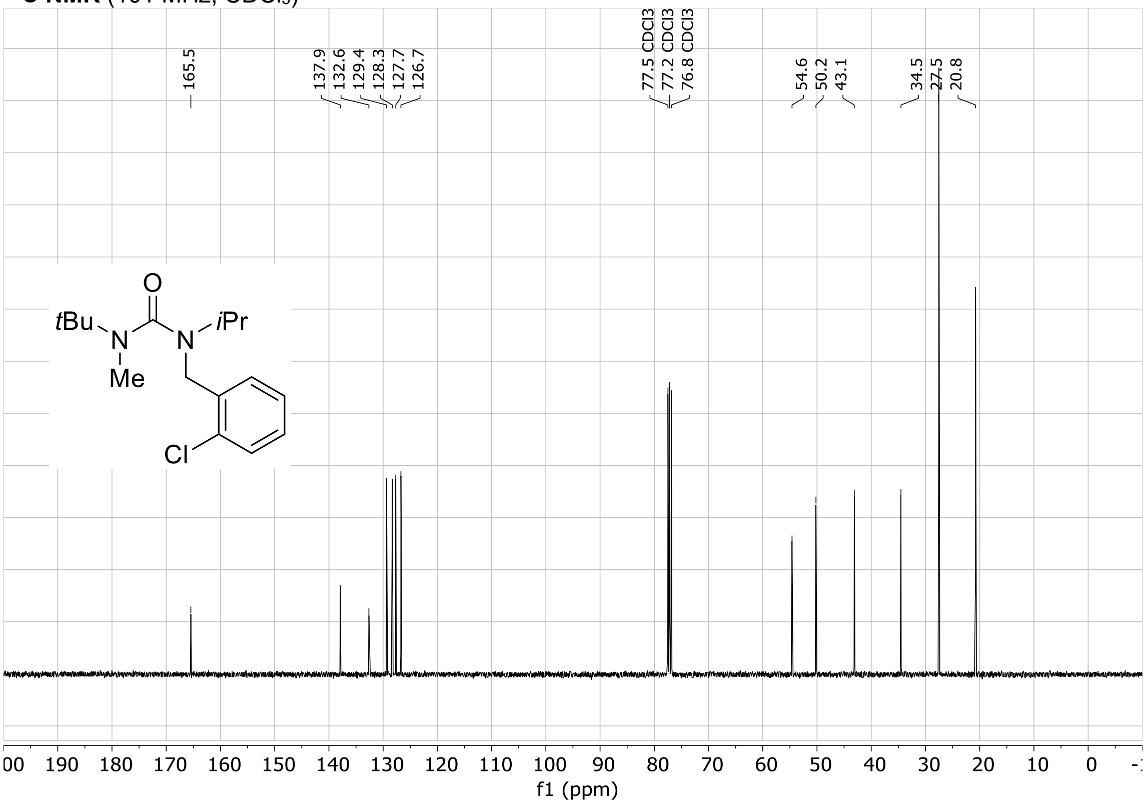

1-(2-Bromobenzyl)-3-(*tert*-butyl)-1-isopropyl-3-methylurea **6h**

^{1}H NMR (400 MHz, $CDCl_3$)

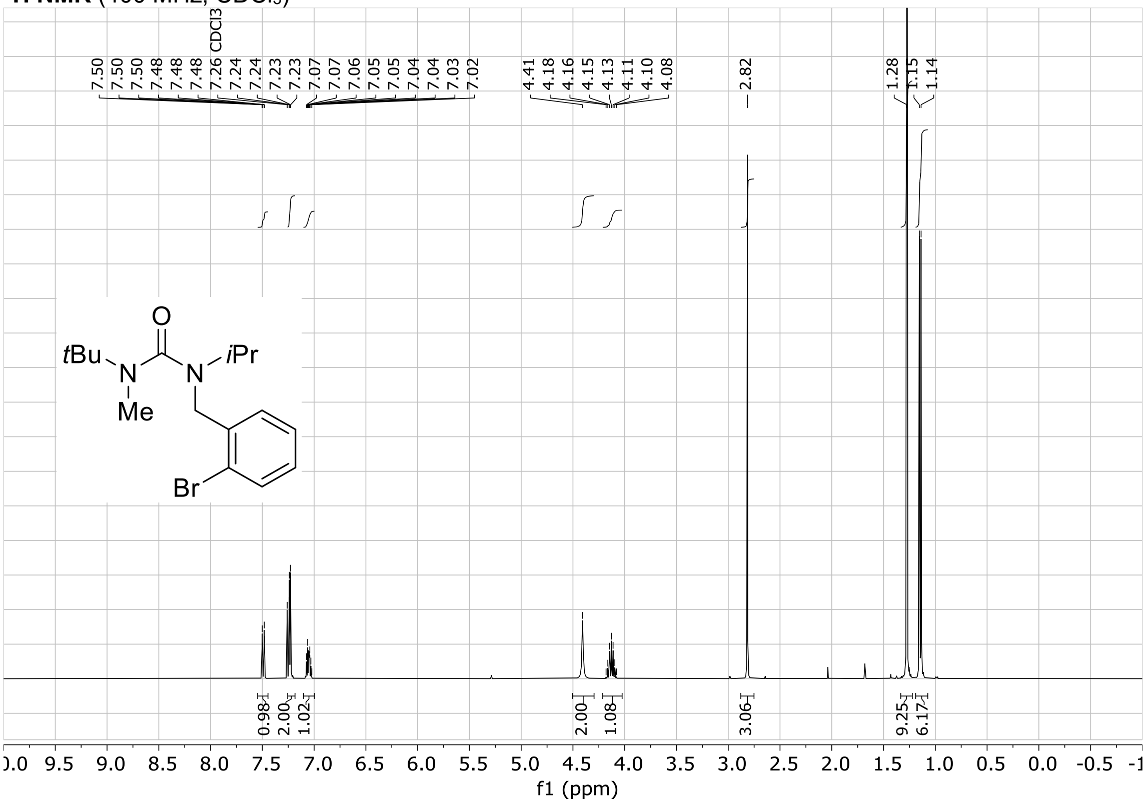

^{13}C NMR (101 MHz, $CDCl_3$)

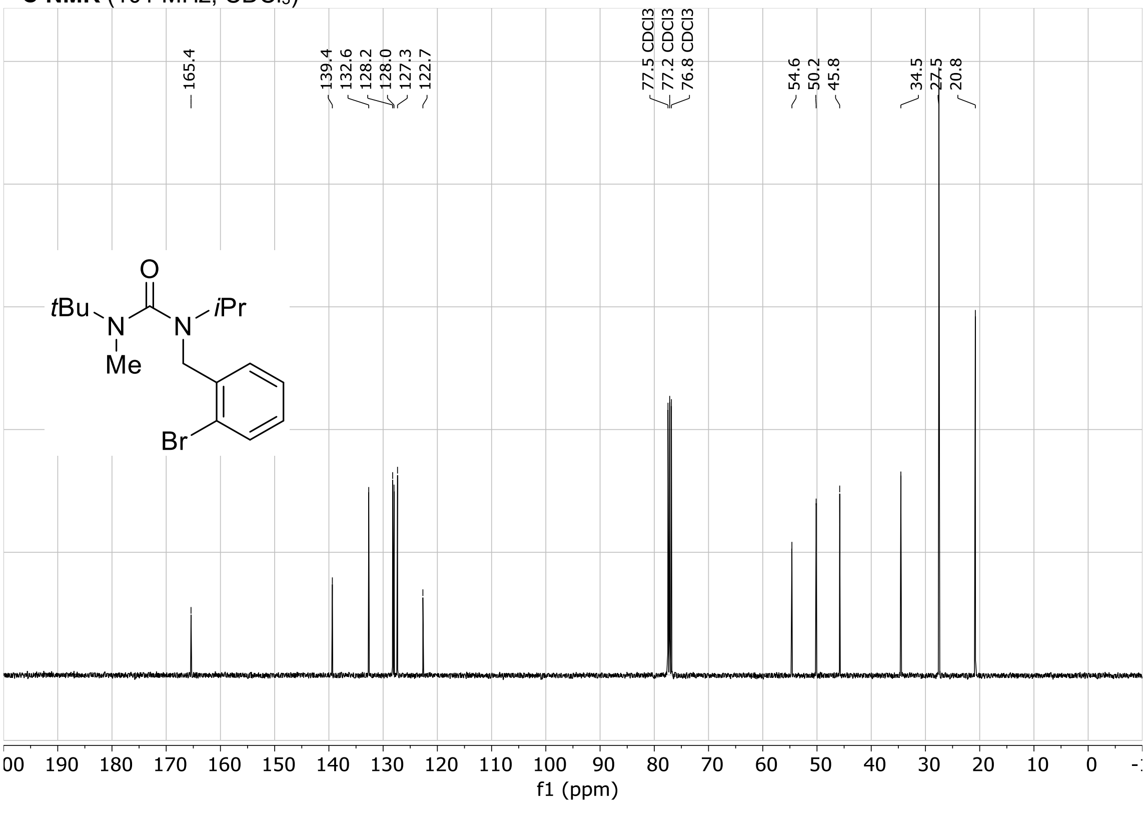

1-(3-Bromobenzyl)-3-(*tert*-butyl)-1-isopropyl-3-methylurea **6i**

¹H NMR (400 MHz, $CDCl_3$)

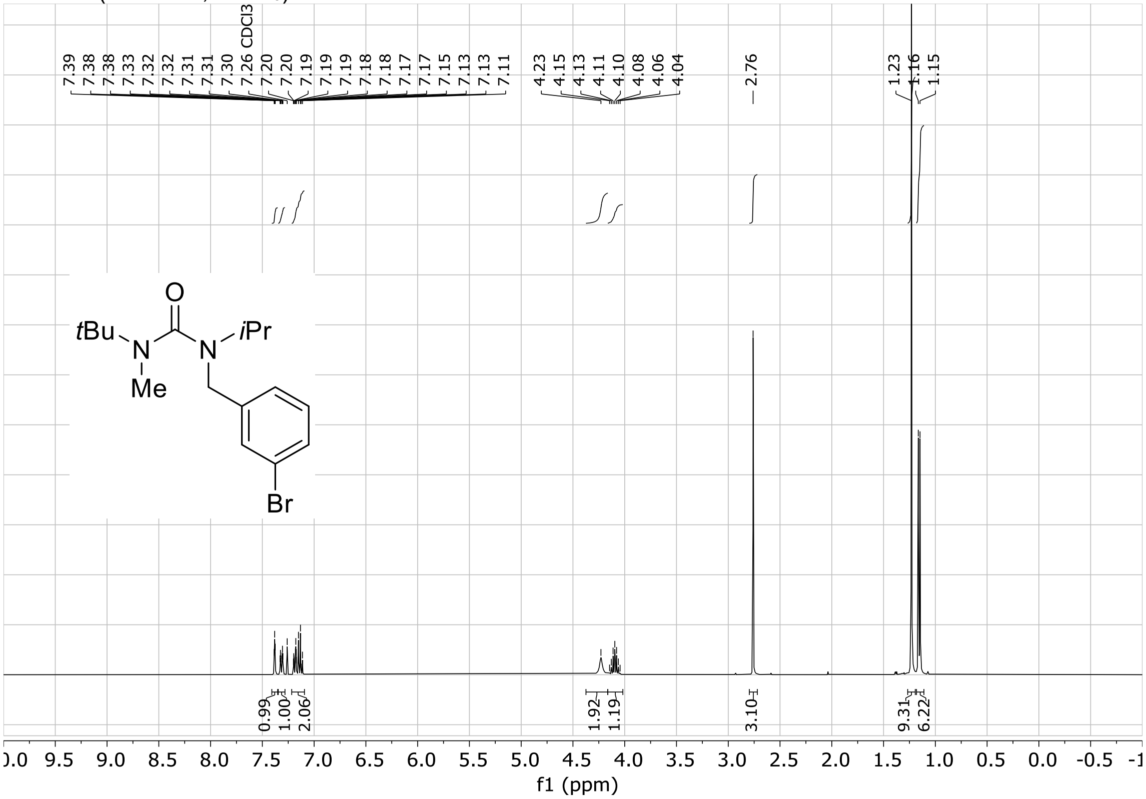

¹³C NMR (101 MHz, $CDCl_3$)

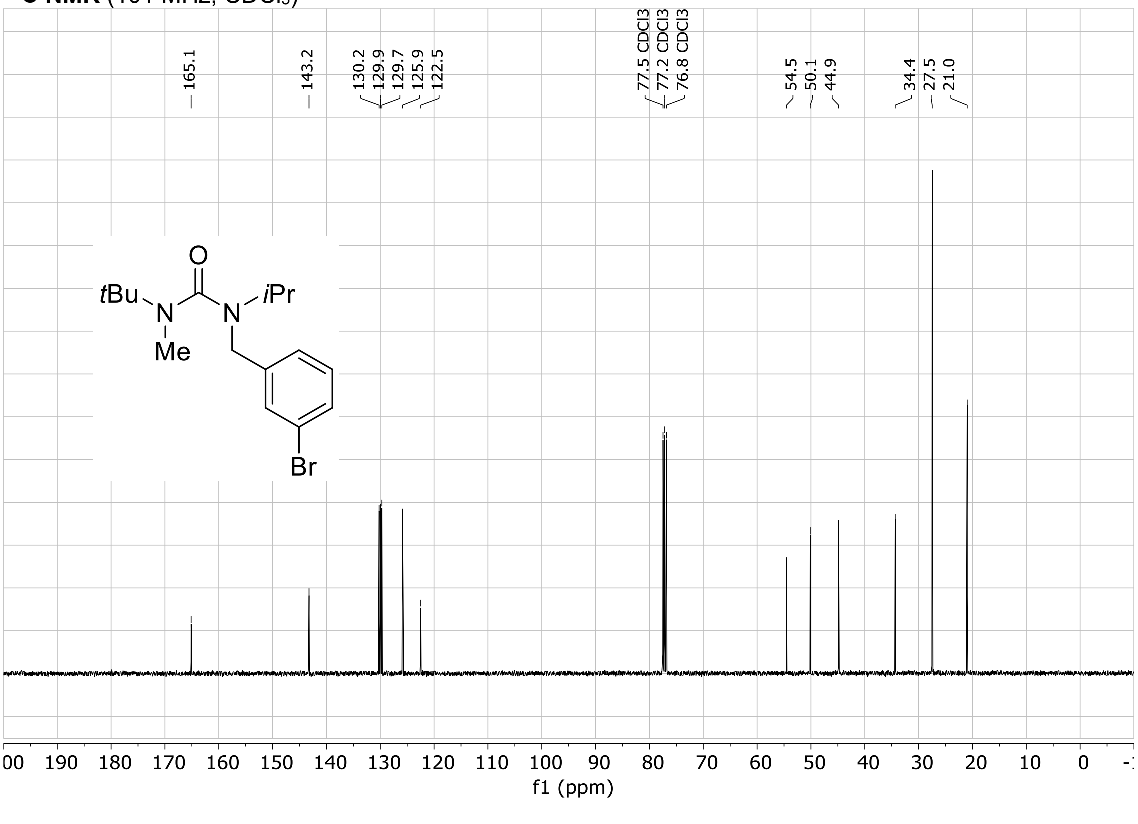

1-(*tert*-Butyl)-3-isopropyl-1-methyl-3-(2-methoxybenzyl)urea **6j**

^{1}H NMR (400 MHz, $CDCl_3$)

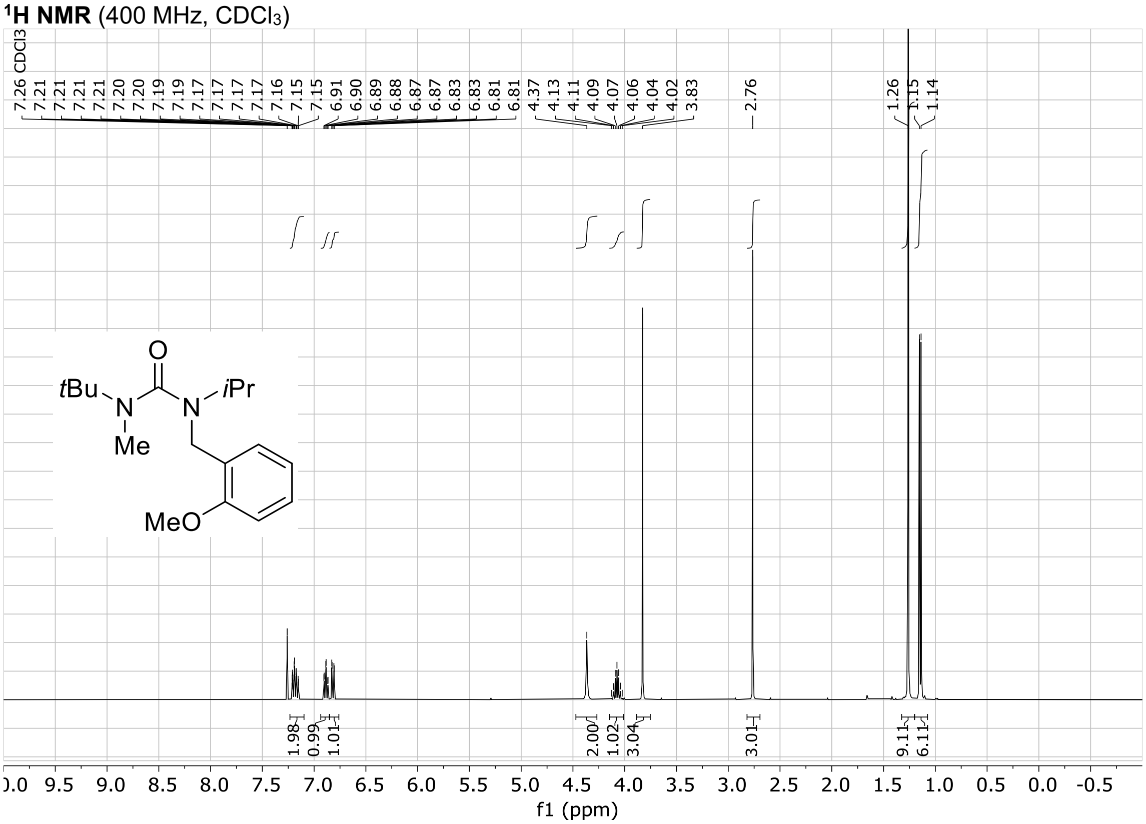

^{13}C NMR (101 MHz, $CDCl_3$)

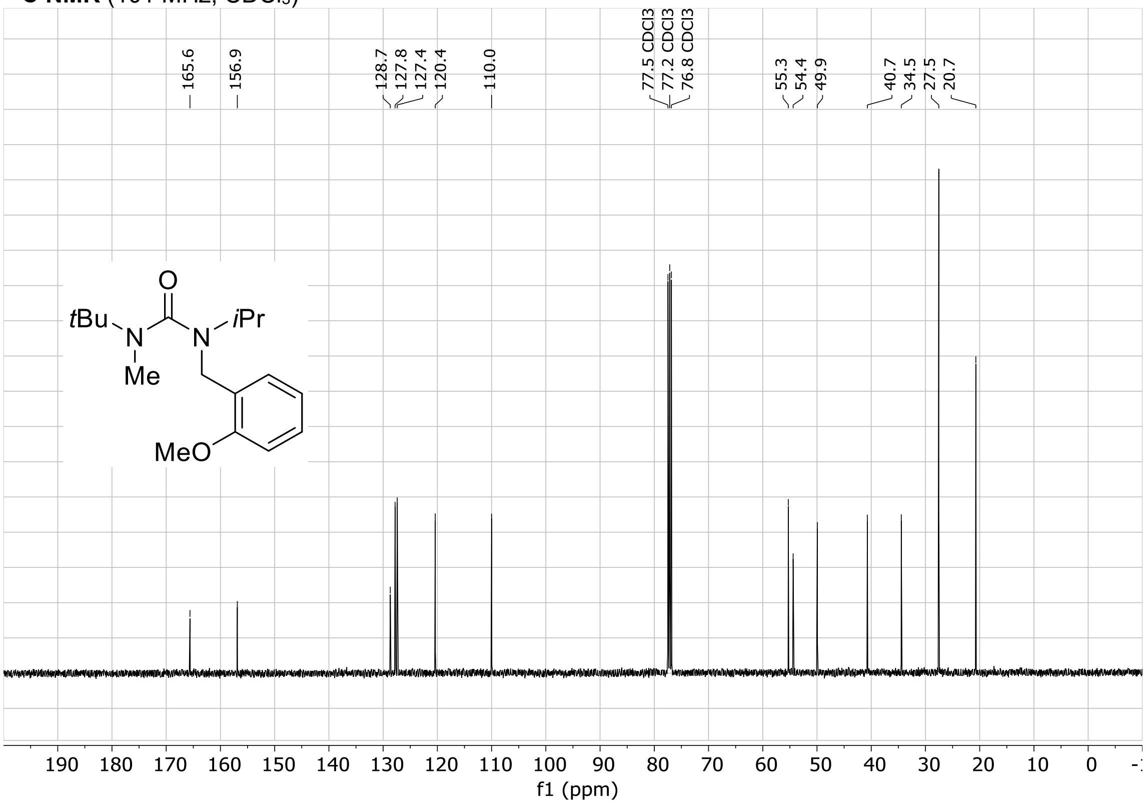

1-(*tert*-Butyl)-3-isopropyl-1-methyl-3-((4-methoxynaphthalen-1-yl)methyl)urea **6k**

^{1}H NMR (400 MHz, $CDCl_3$)

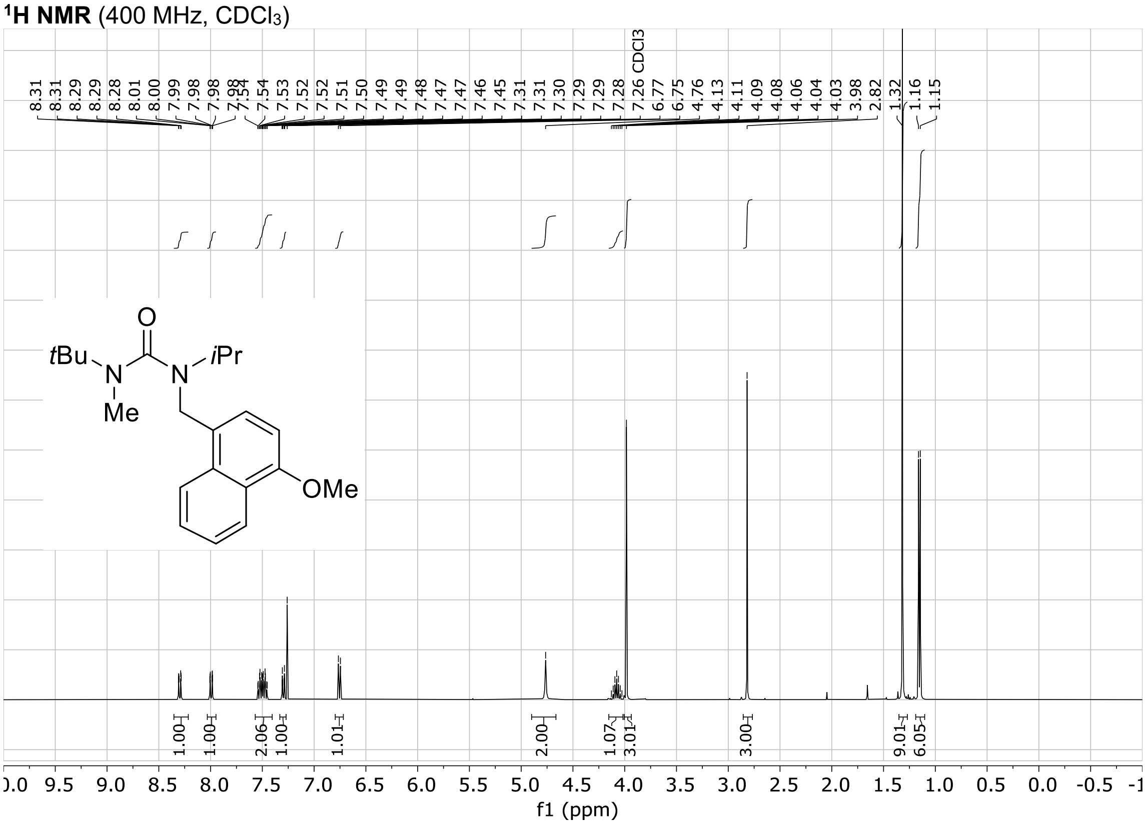

^{13}C NMR (101 MHz, $CDCl_3$)

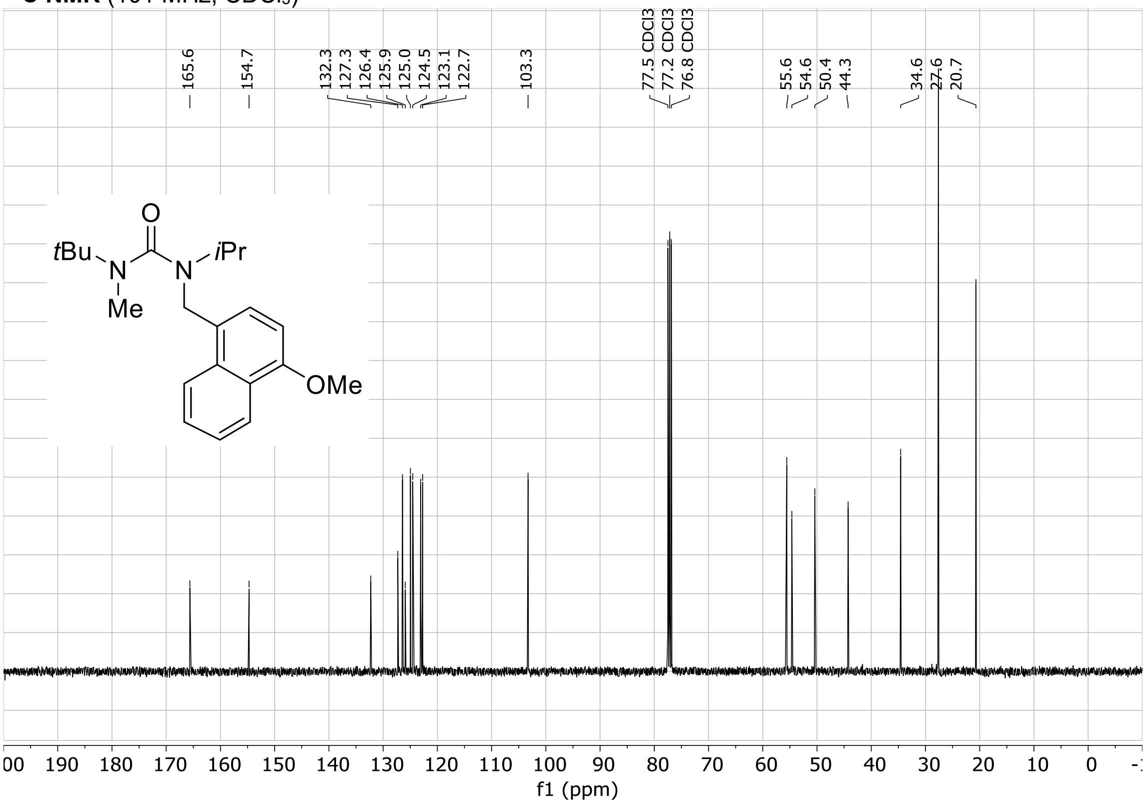

1-(*tert*-Butyl)-3-isopropyl-1-methyl-3-((4-methylnaphthalen-1-yl)methyl)urea **6l**

^{1}H NMR (400 MHz, $CDCl_3$)

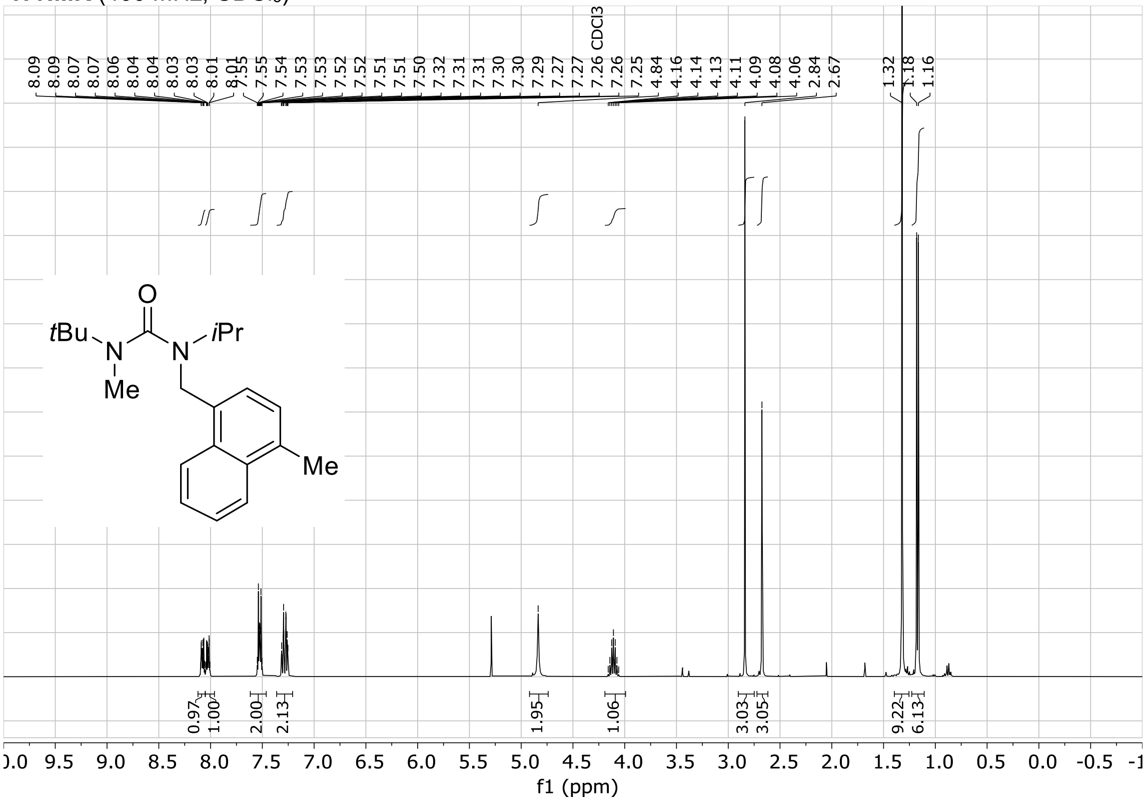

^{13}C NMR (101 MHz, $CDCl_3$)

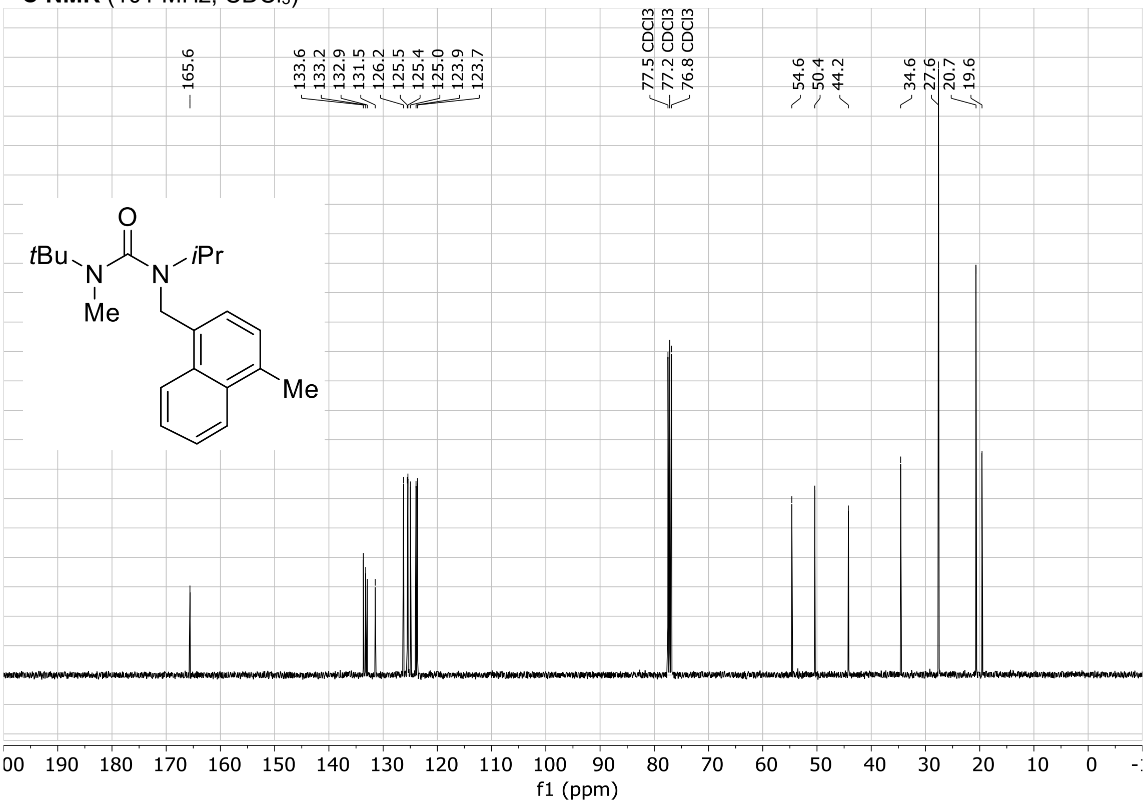

1-(*tert*-Butyl)-3-(2,6-dimethylbenzyl)-3-isopropyl-1-methylurea **SI-1**

^{1}H NMR (400 MHz, $CDCl_3$)

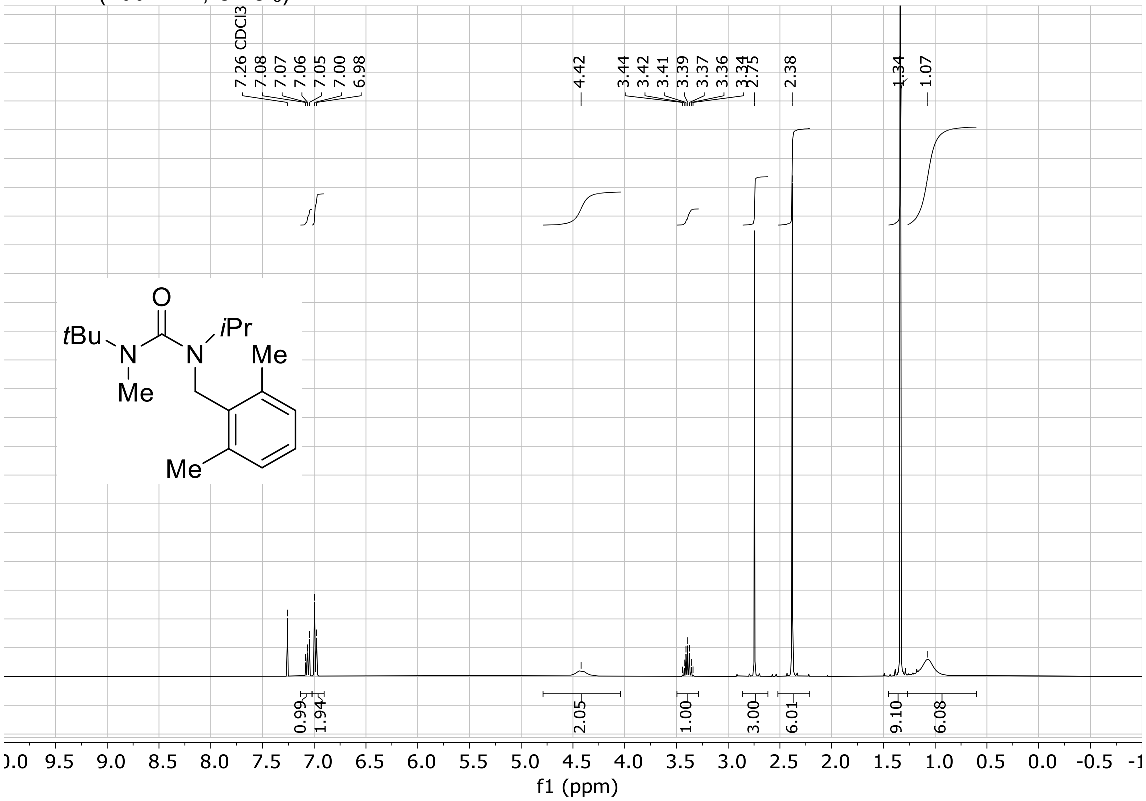

^{13}C NMR (101 MHz, $CDCl_3$)

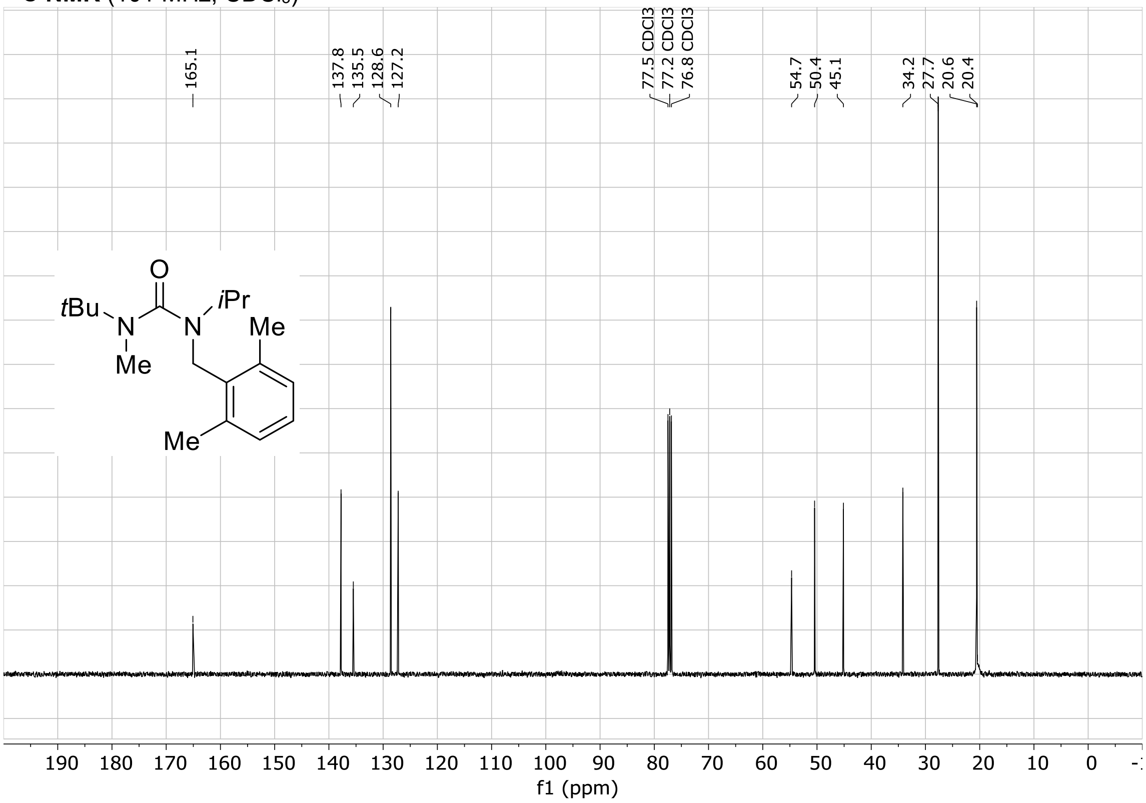

1-Benzyl-3-(*tert*-butyl)-1-(dicyclopropylmethyl)-3-methylurea **8a**

^{1}H NMR (400 MHz, $CDCl_3$)

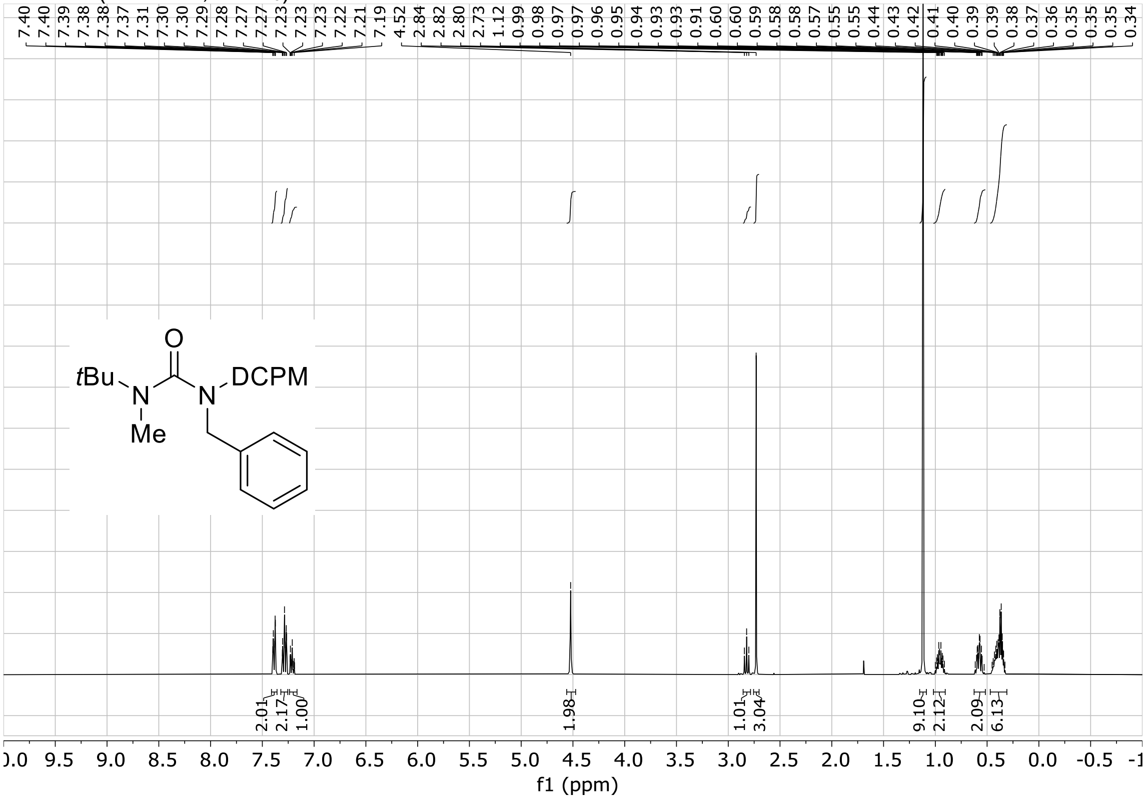

^{13}C NMR (101 MHz, $CDCl_3$)

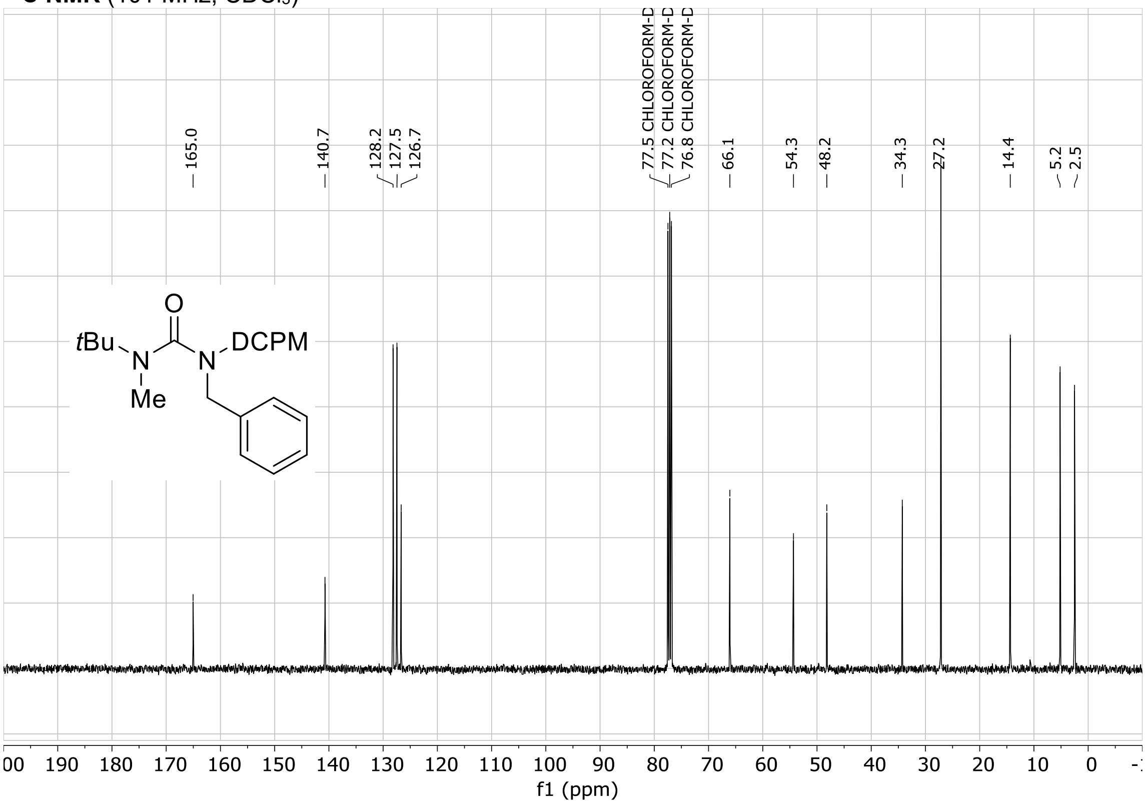

1-(2-Bromobenzyl)-3-(*tert*-butyl)-1-(dicyclopropylmethyl)-3-methylurea **8b**

^{1}H NMR (400 MHz, $CDCl_3$)

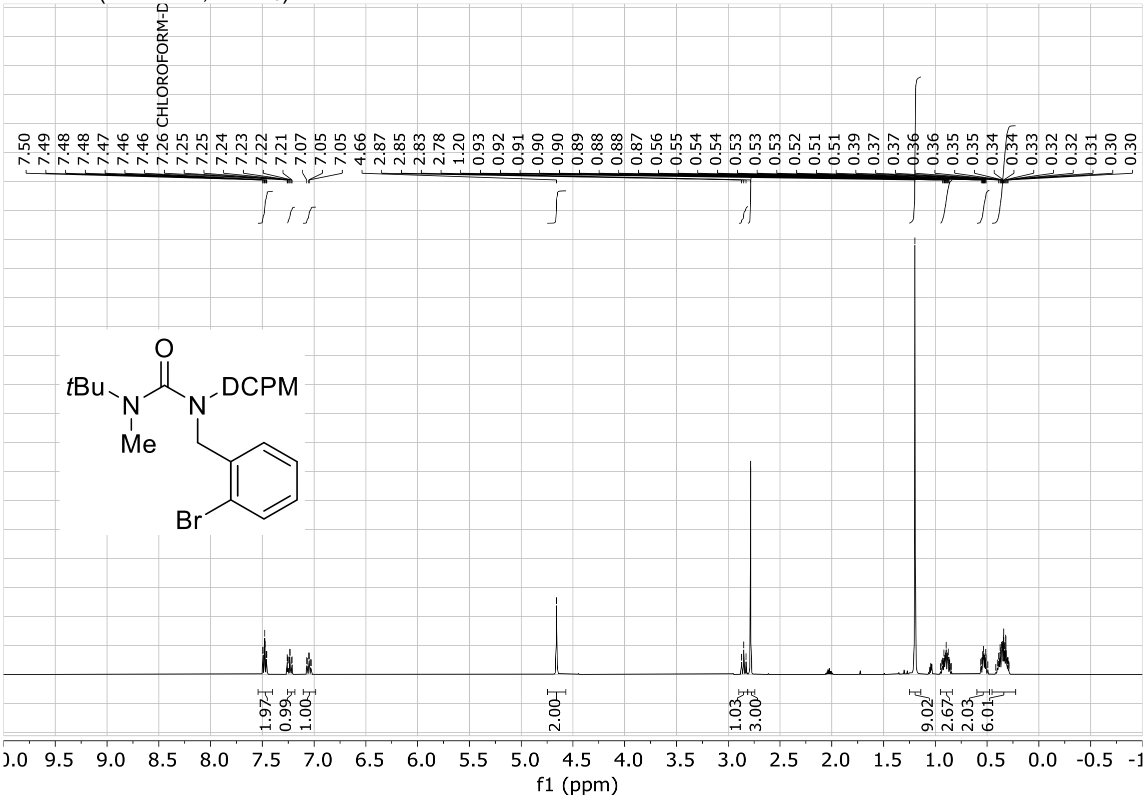

^{13}C NMR (101 MHz, $CDCl_3$)

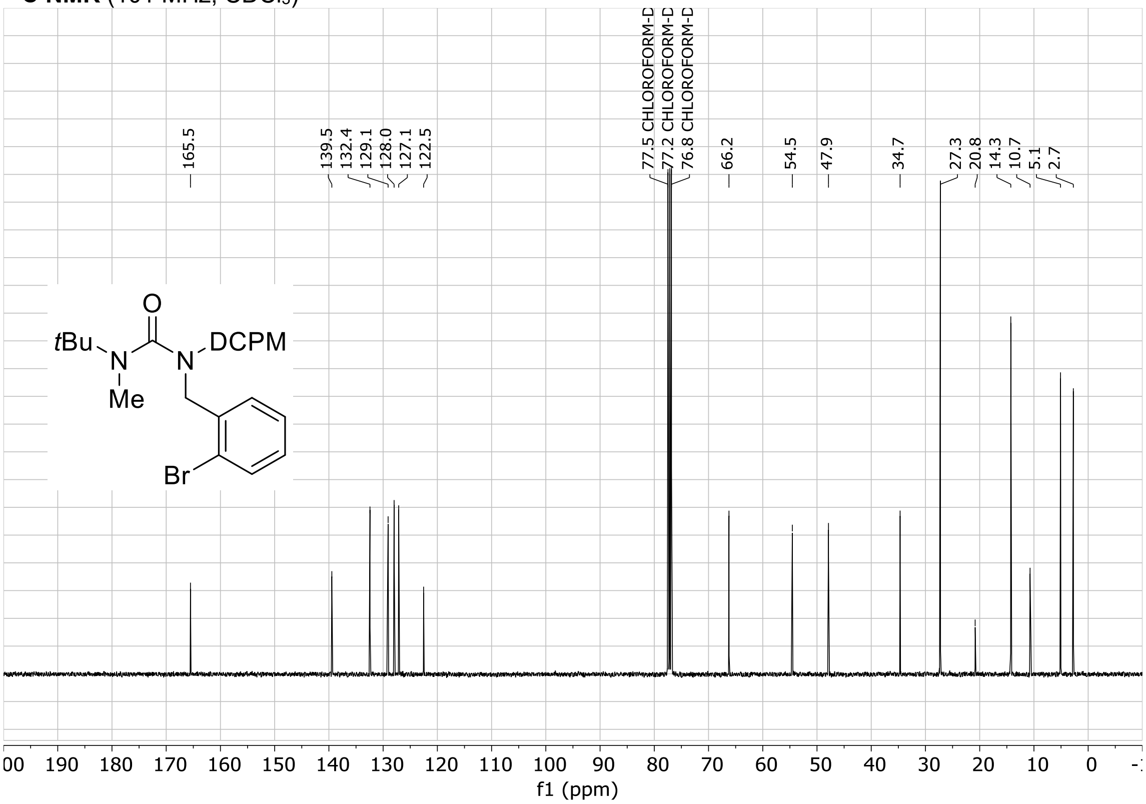

1-(*tert*-Butyl)-3-(dicyclopropylmethyl)-1-methyl-3-(2-methylbenzyl)urea **8c**

^{1}H NMR (400 MHz, $CDCl_3$)

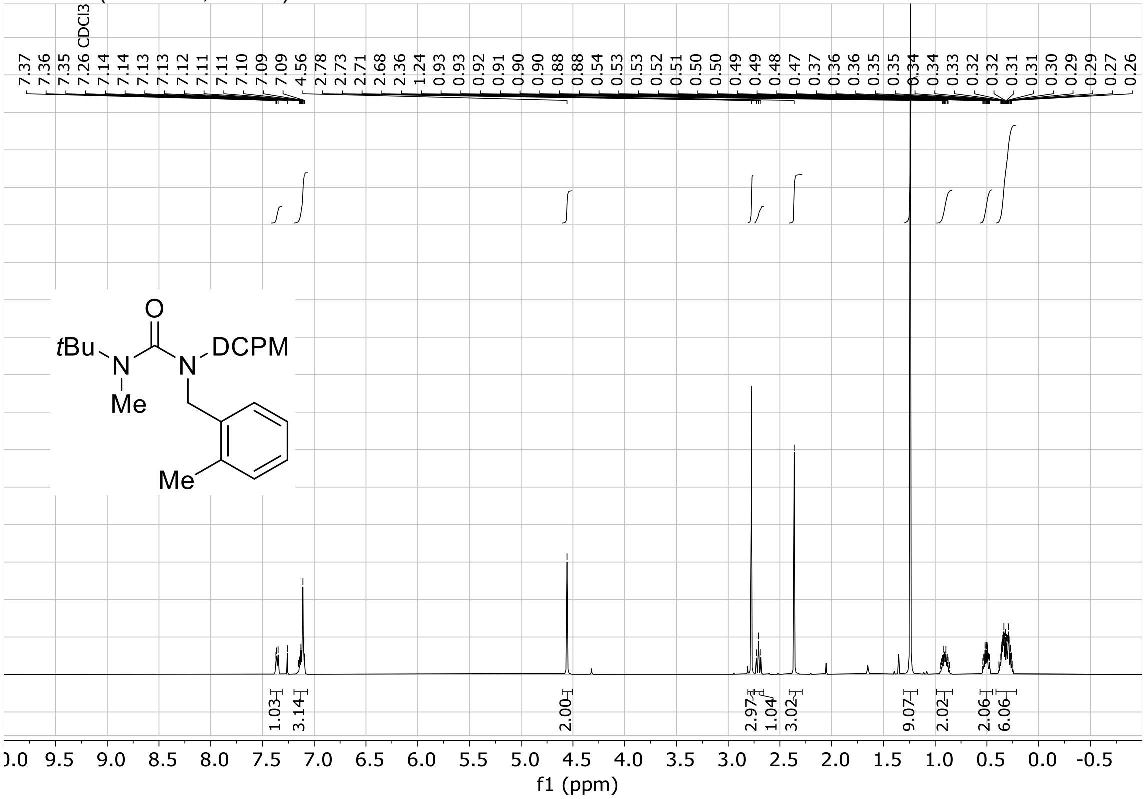

^{13}C NMR (101 MHz, $CDCl_3$)

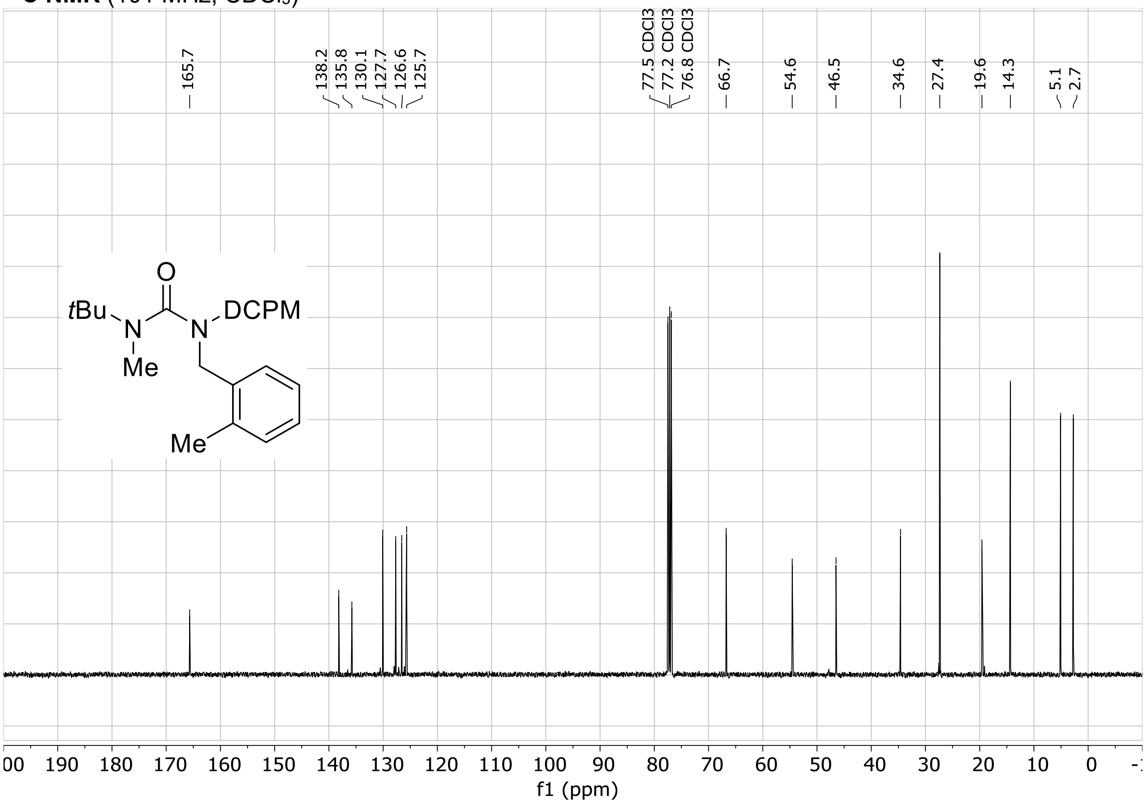

1-(*tert*-Butyl)-3-(dicyclopropylmethyl)-1-methyl-3-(naphthalen-1-ylmethyl)urea **8e**

^{1}H NMR (400 MHz, $CDCl_3$)

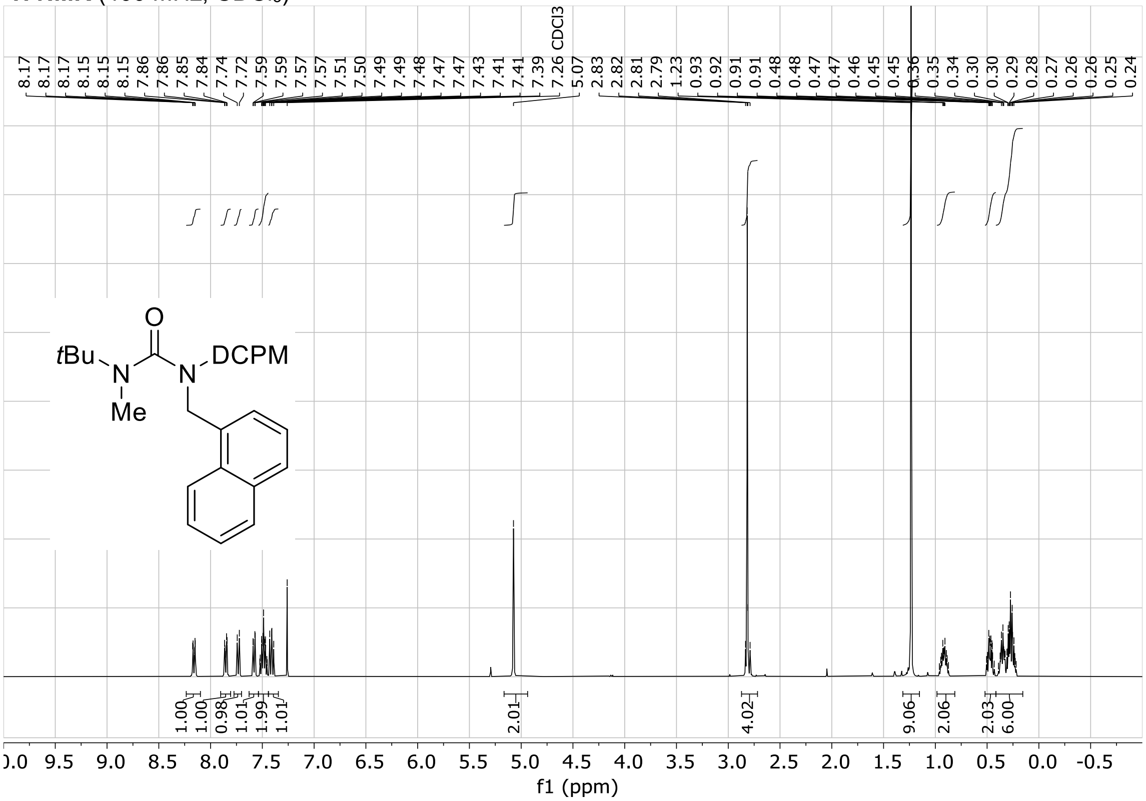

^{13}C NMR (101 MHz, $CDCl_3$)

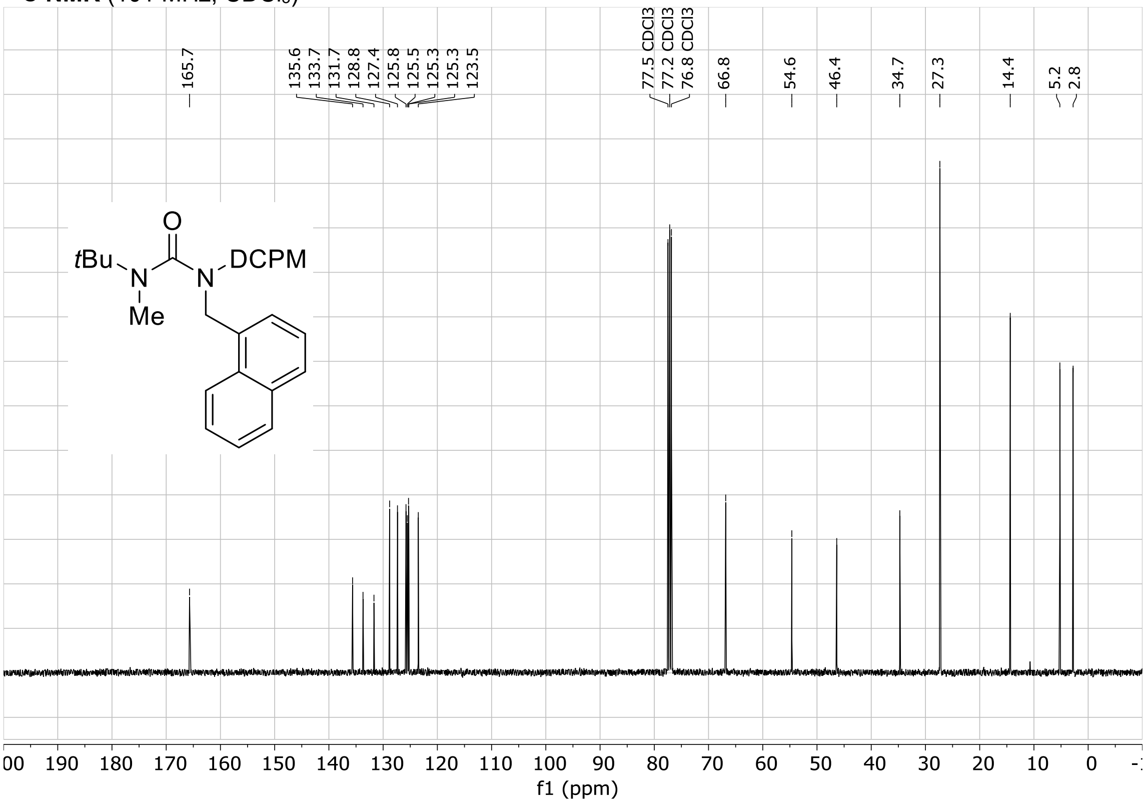

1-(*tert*-Butyl)-3-(dicyclopropylmethyl)-1-methyl-3-(2-methylbenzyl)urea **10**

^{1}H NMR (400 MHz, $CDCl_3$)

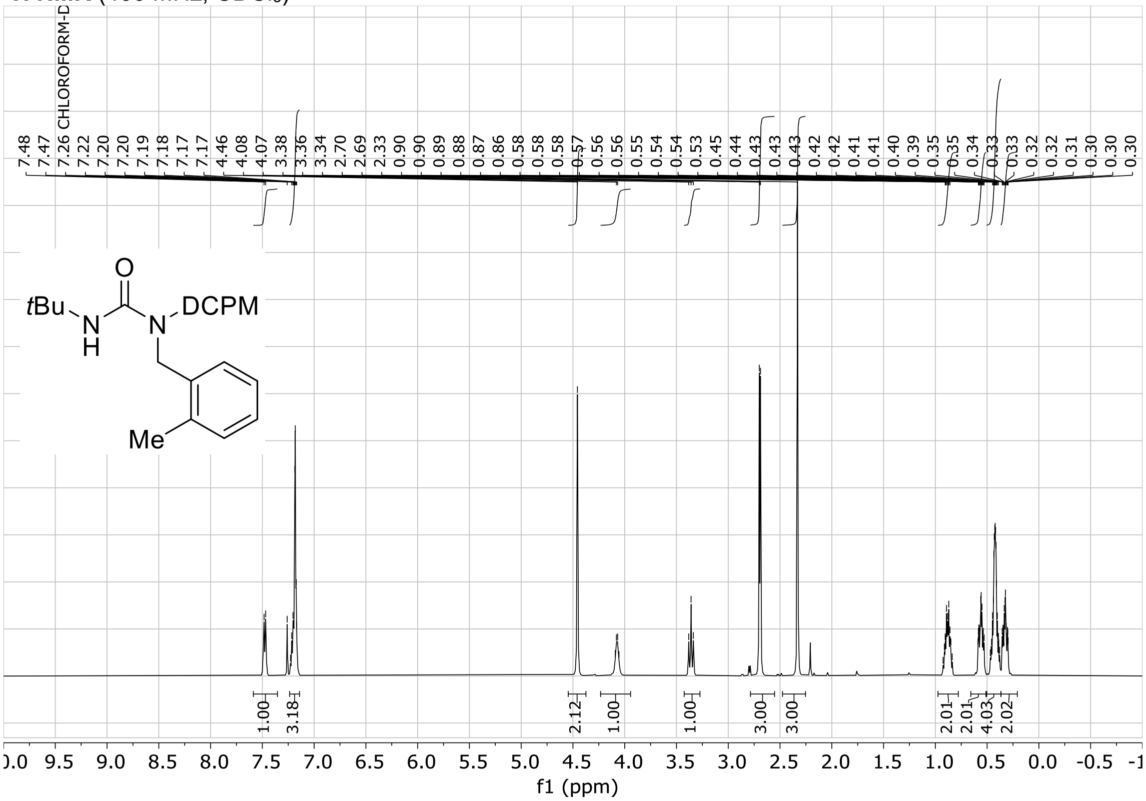

^{13}C NMR (101 MHz, $CDCl_3$)

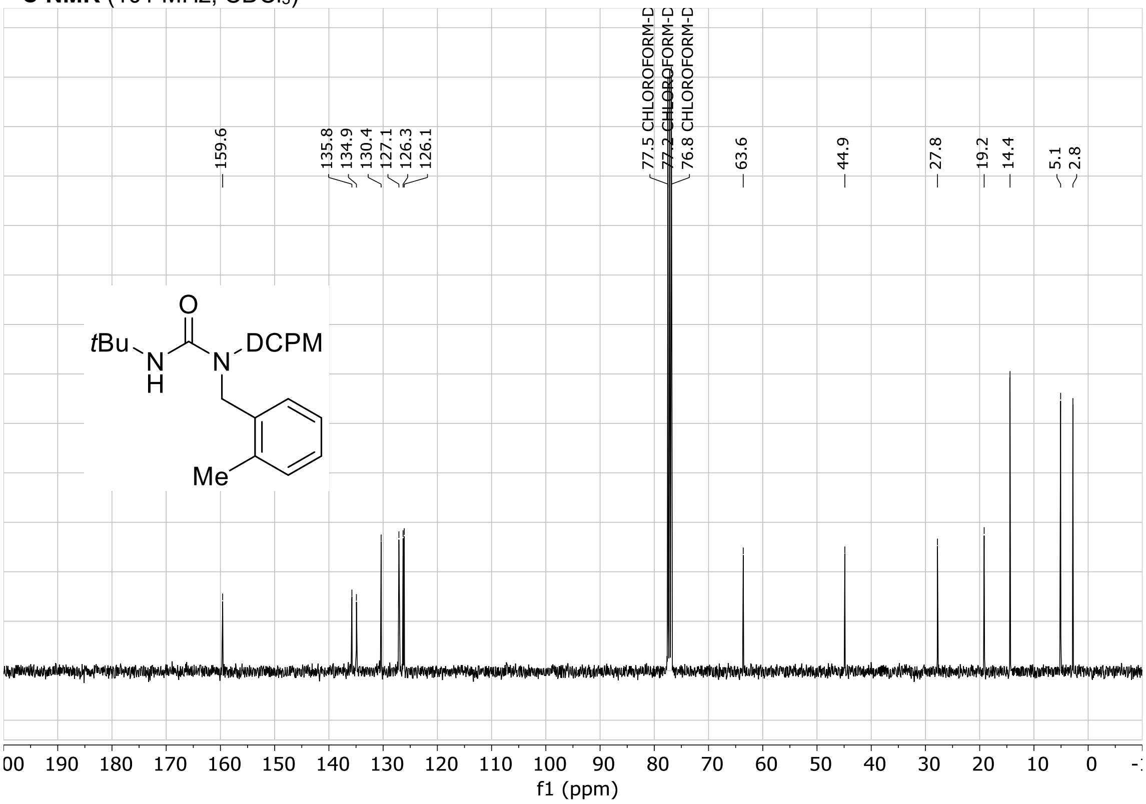

2.1.2 Products from enantioselective deprotonation-alkylation

1-(*tert*-Butyl)-3-ethyl-1-methyl-3-(1-phenylethyl)urea **2a**

^{1}H NMR (400 MHz, $CDCl_3$)

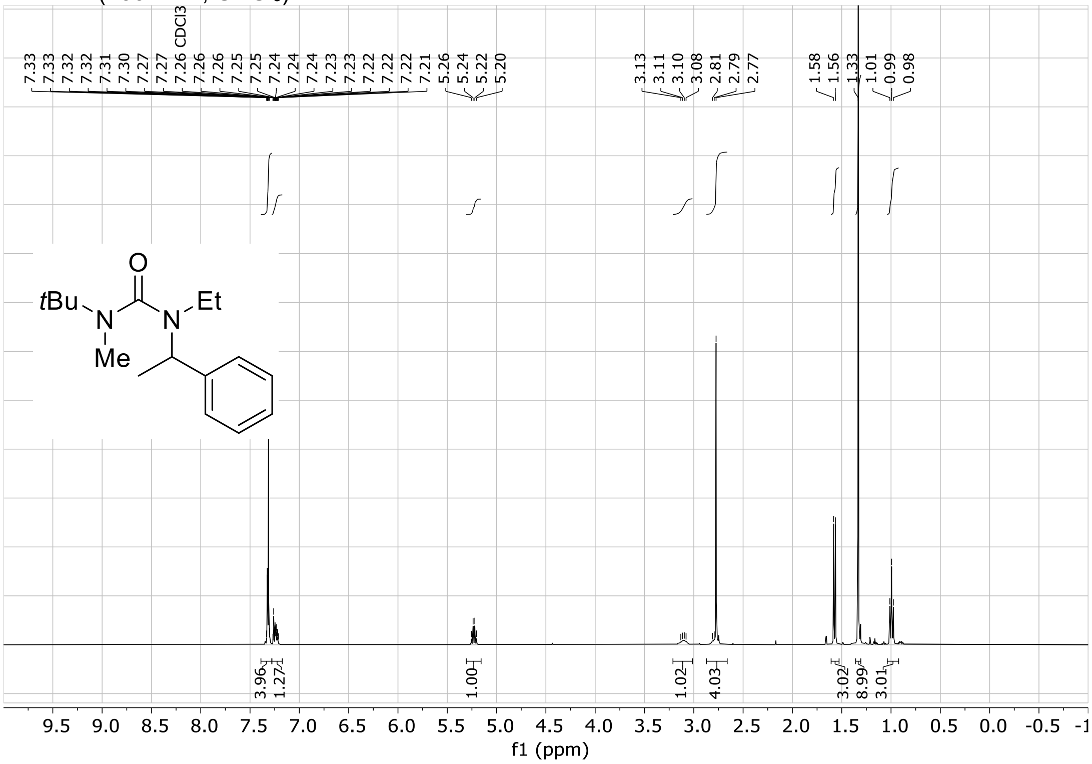

^{13}C NMR (101 MHz, $CDCl_3$)

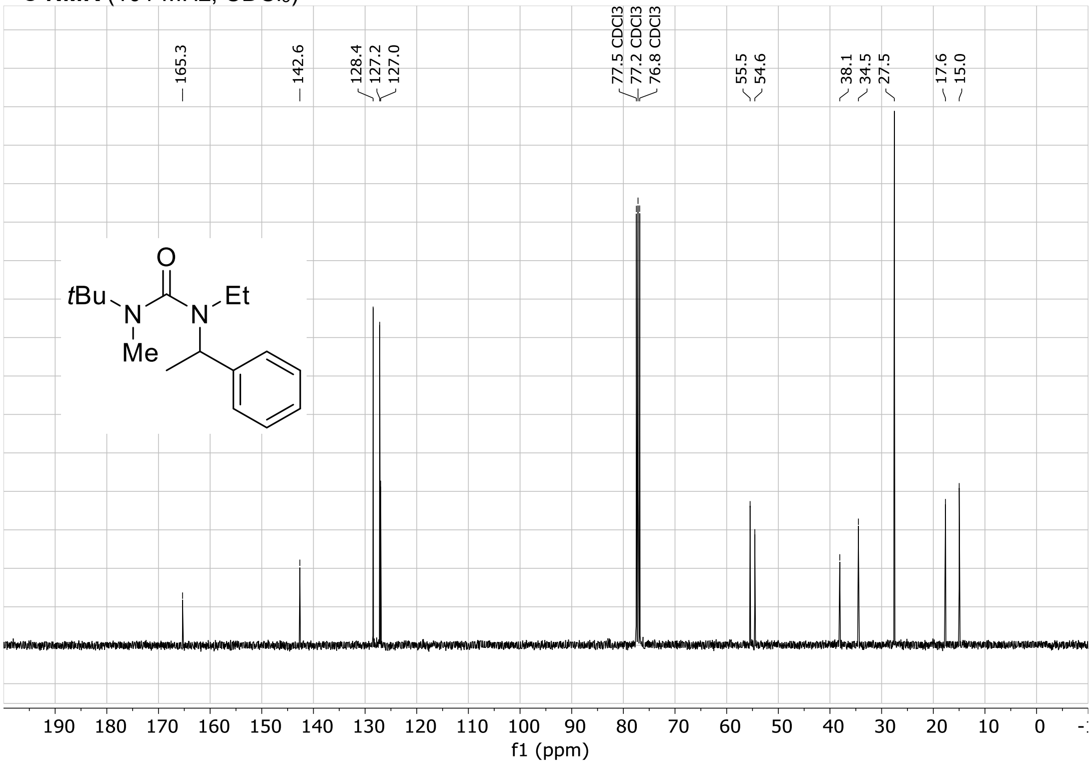

(*S*)-1-(*tert*-Butyl)-3-isopropyl-1-methyl-3-(1-phenylethyl)urea **2b = 7a**

^{1}H NMR (400 MHz, $CDCl_3$)

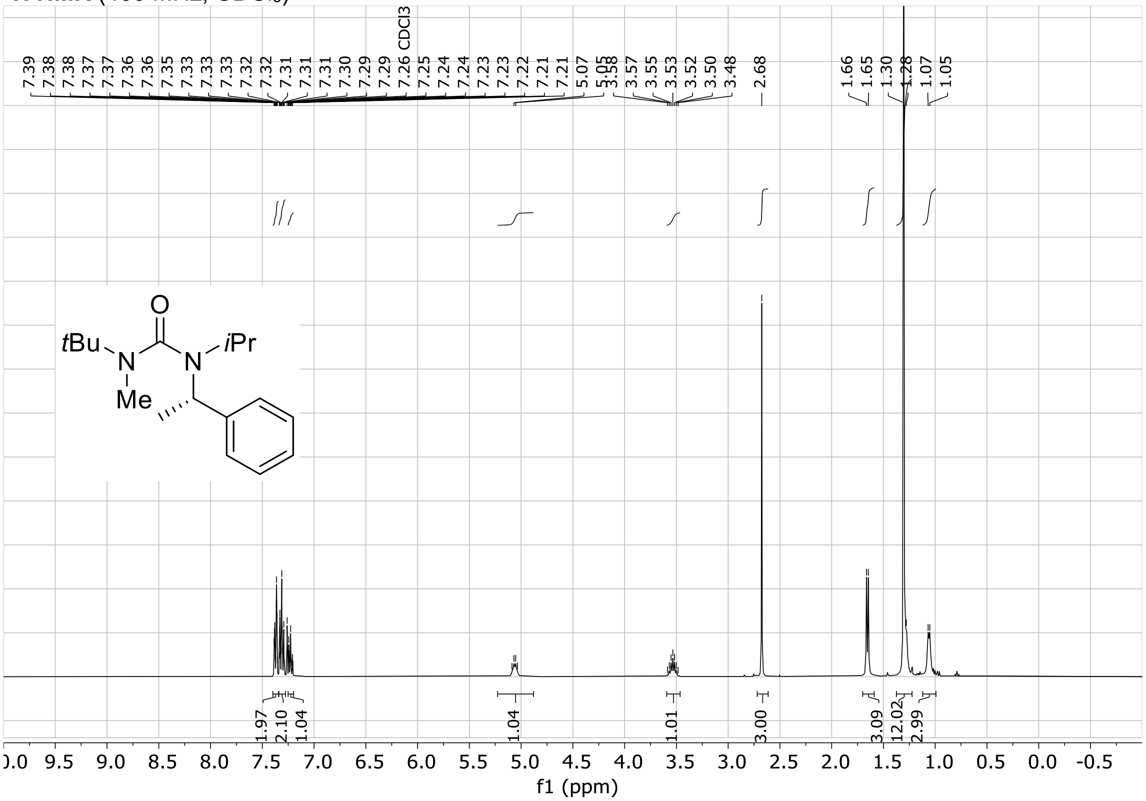

^{13}C NMR (101 MHz, $CDCl_3$)

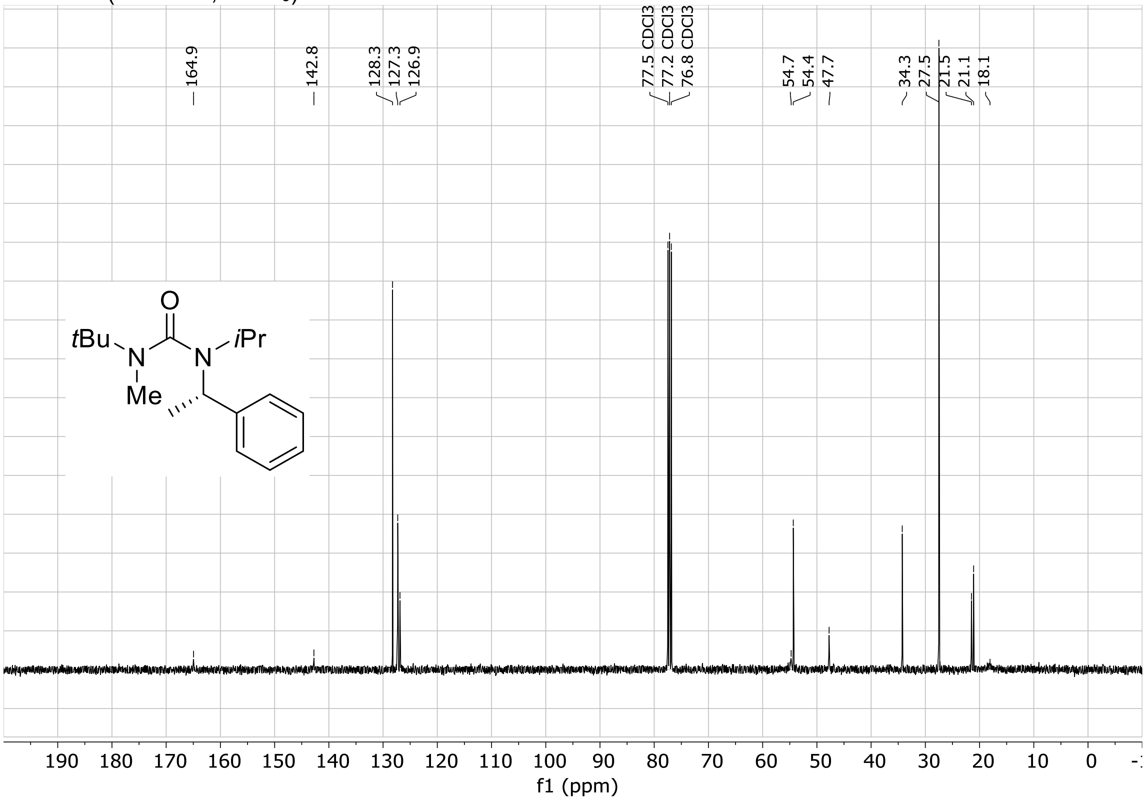

(*S*)-1-(*tert*-Butyl)-1-ethyl-3-isopropyl -3-(1-phenylethyl)urea **SI-2**

¹H NMR (500 MHz, $CDCl_3$)

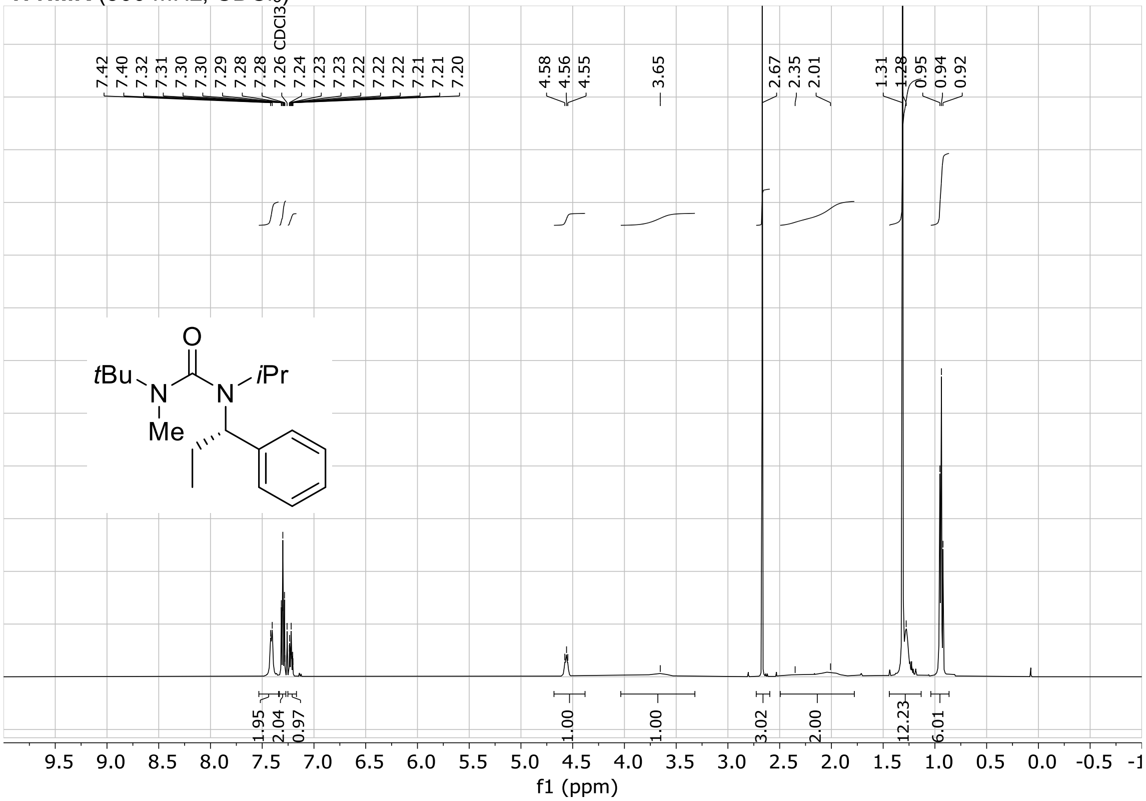

¹³C NMR (126 MHz, $CDCl_3$)

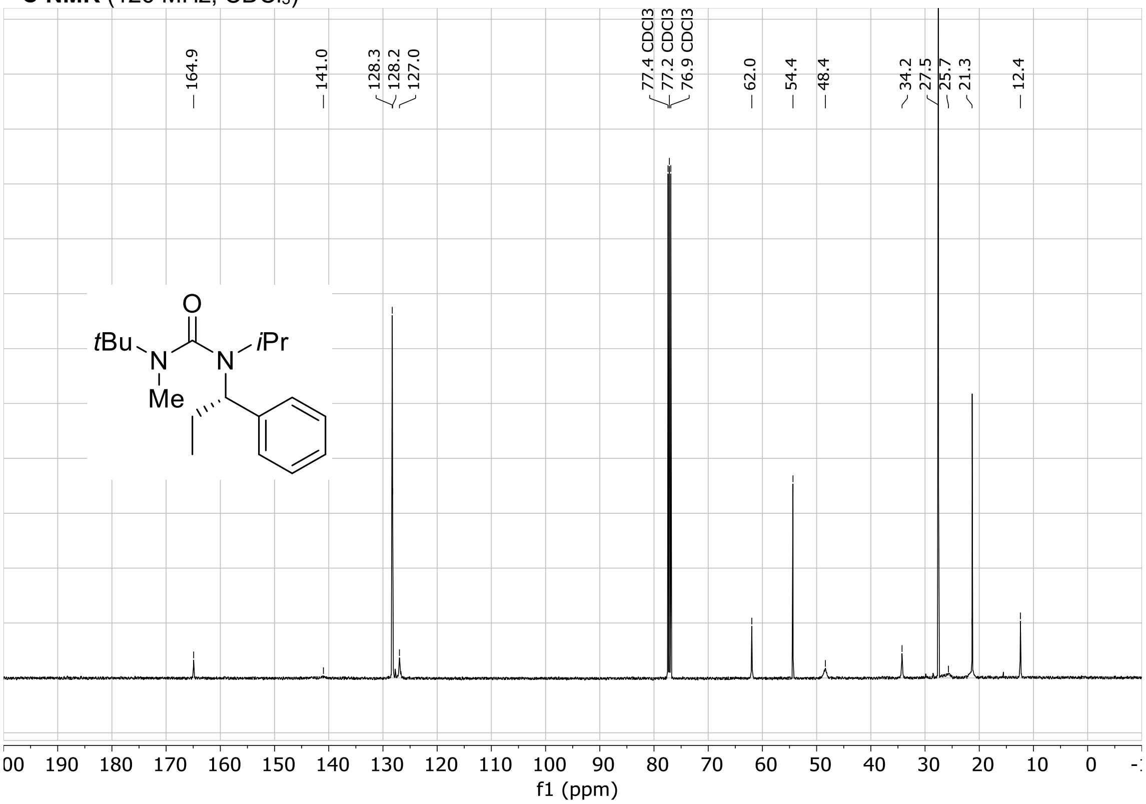

(*S*)-1-Ethyl-1,3-diisopropyl-3-(1-phenylethyl)urea **2d**

^{1}H NMR (400 MHz, $CDCl_3$)

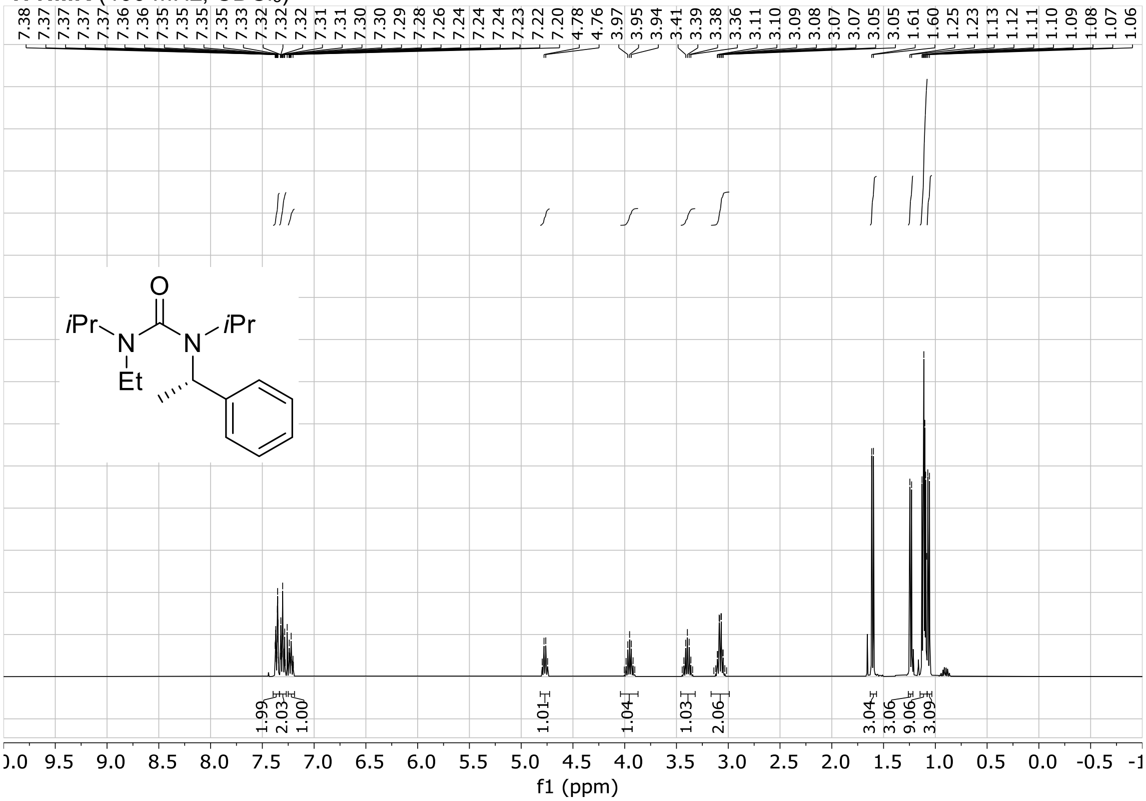

^{13}C NMR (101 MHz, $CDCl_3$)

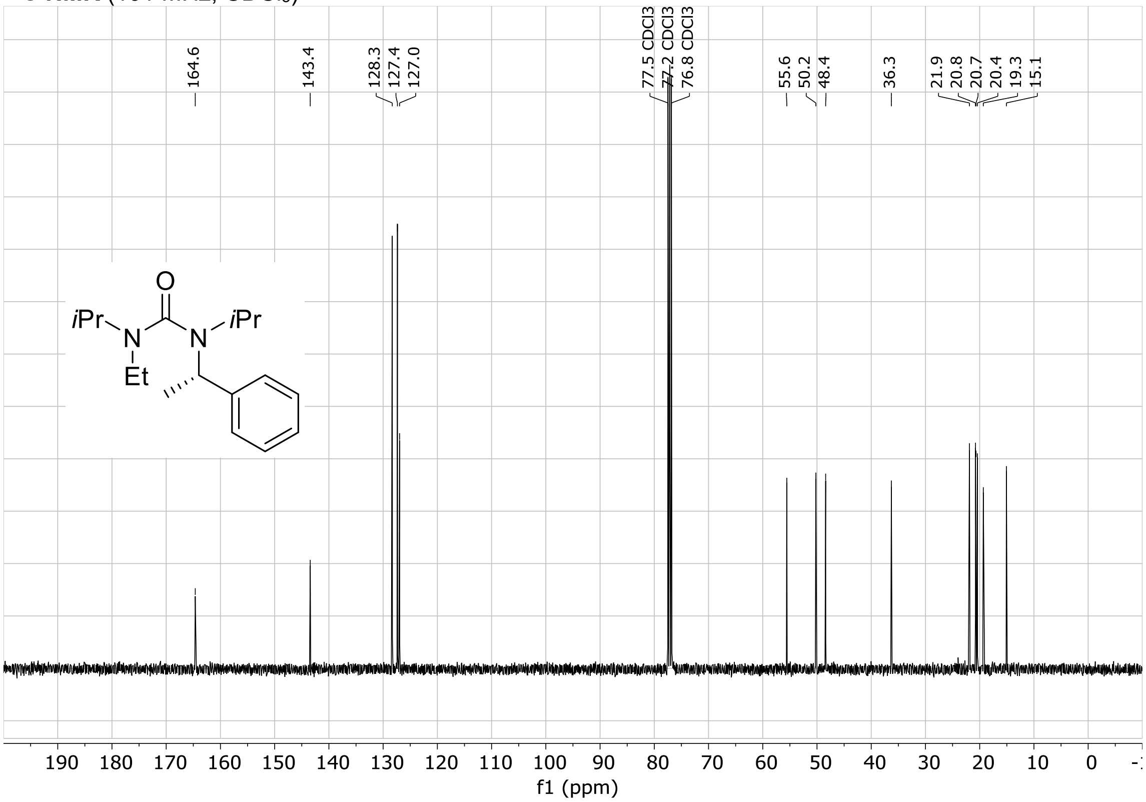

(*S*)-1-(*tert*-Butyl)-1-ethyl-3-isopropyl-3-(1-phenylethyl)urea **2e**

^{1}H NMR (400 MHz, $CDCl_3$)

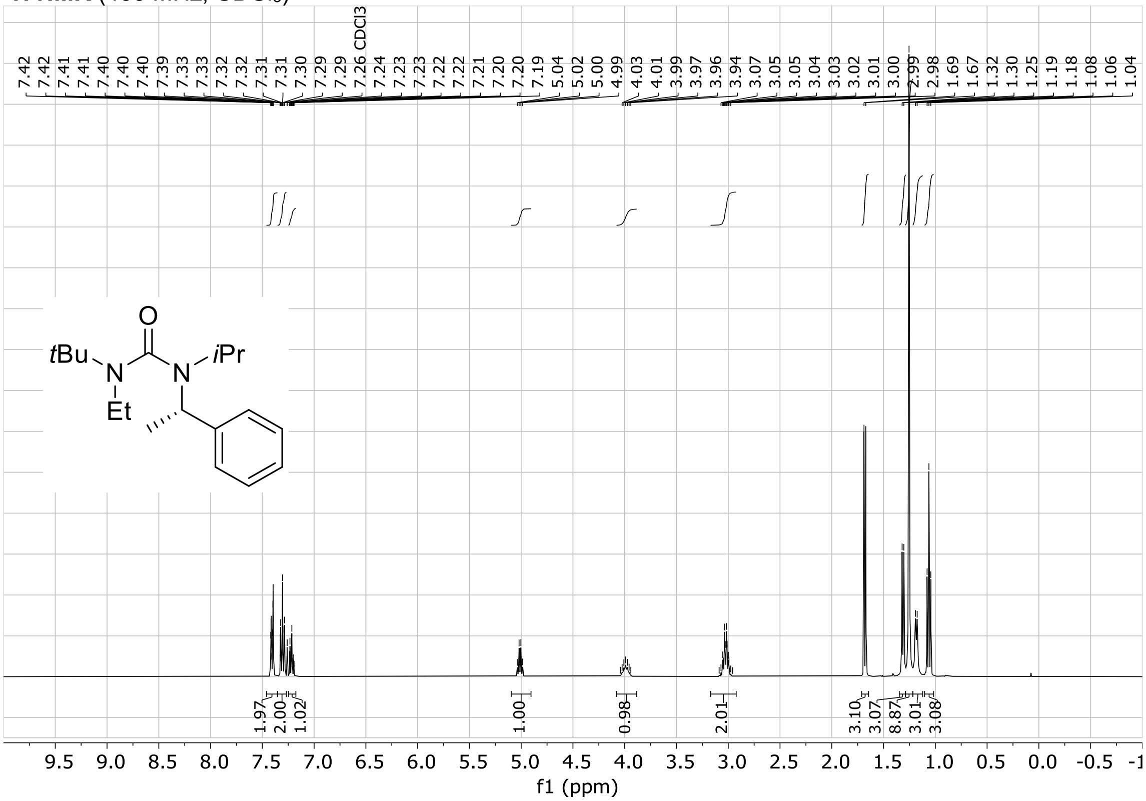

^{13}C NMR (101 MHz, $CDCl_3$)

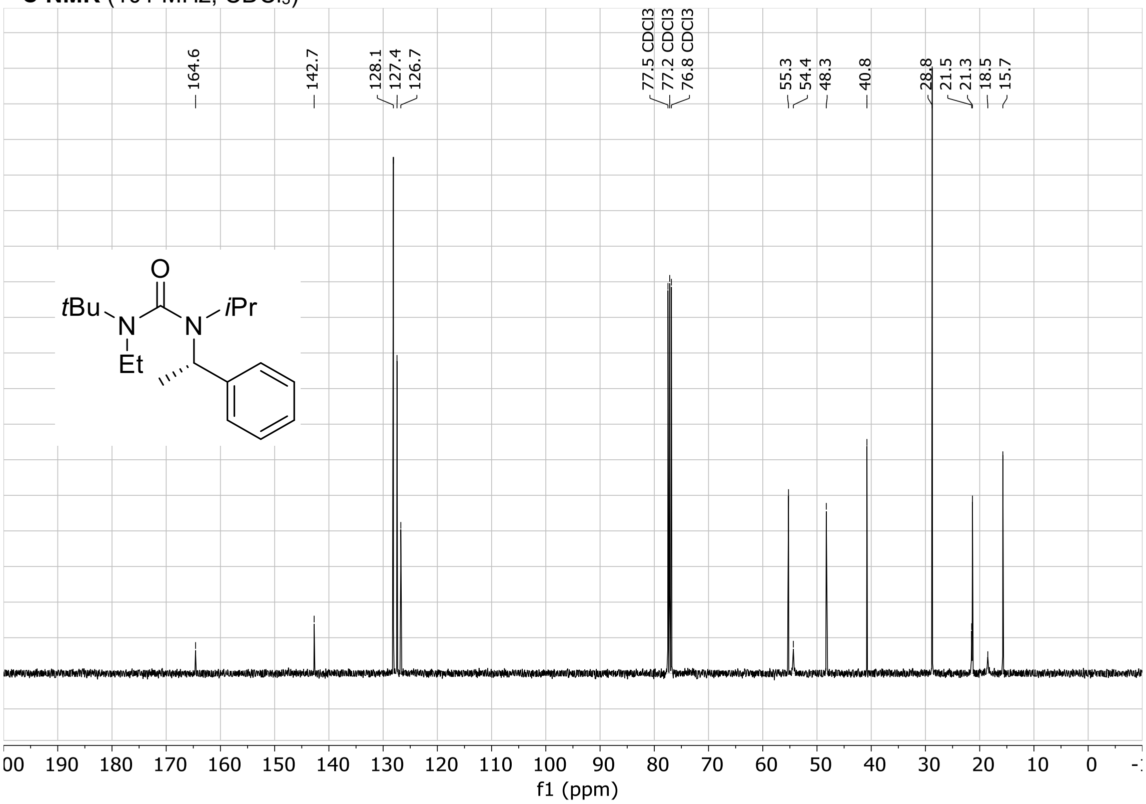

(*S*)-1-(*tert*-Butyl)-3-cyclohexyl-1-methyl-3-(1-phenylethyl)urea **7b**

^{1}H NMR (400 MHz, $CDCl_3$)

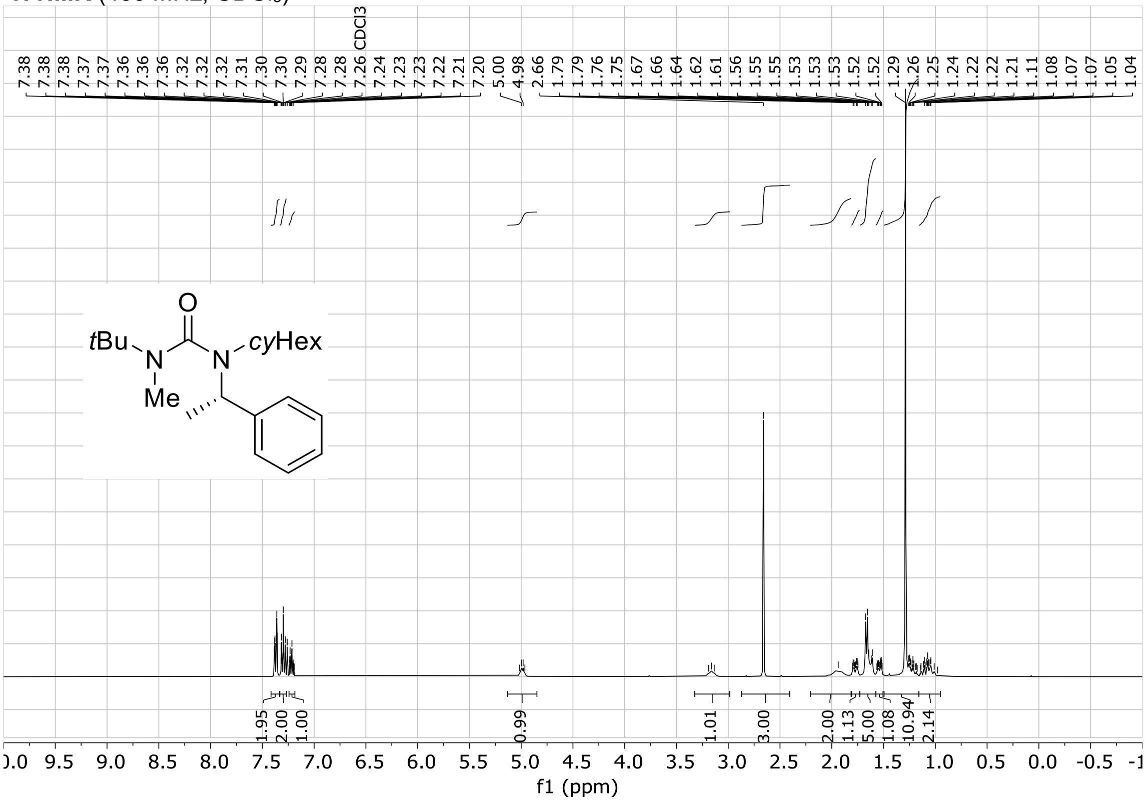

^{13}C NMR (101 MHz, $CDCl_3$)

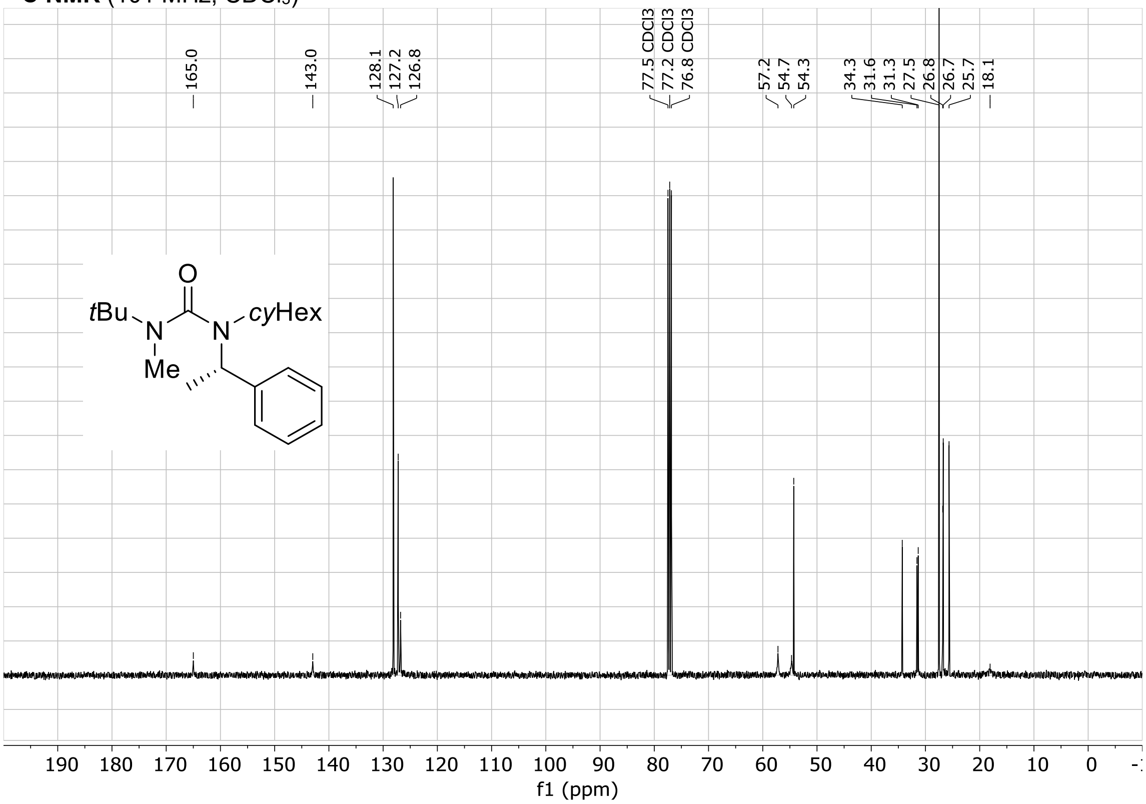

(*S*)-1-(*tert*-Butyl)-3-isopropyl-1-methyl-3-(1-(*o*-tolyl)ethyl)urea **7c**

^{1}H NMR (400 MHz, $CDCl_3$)

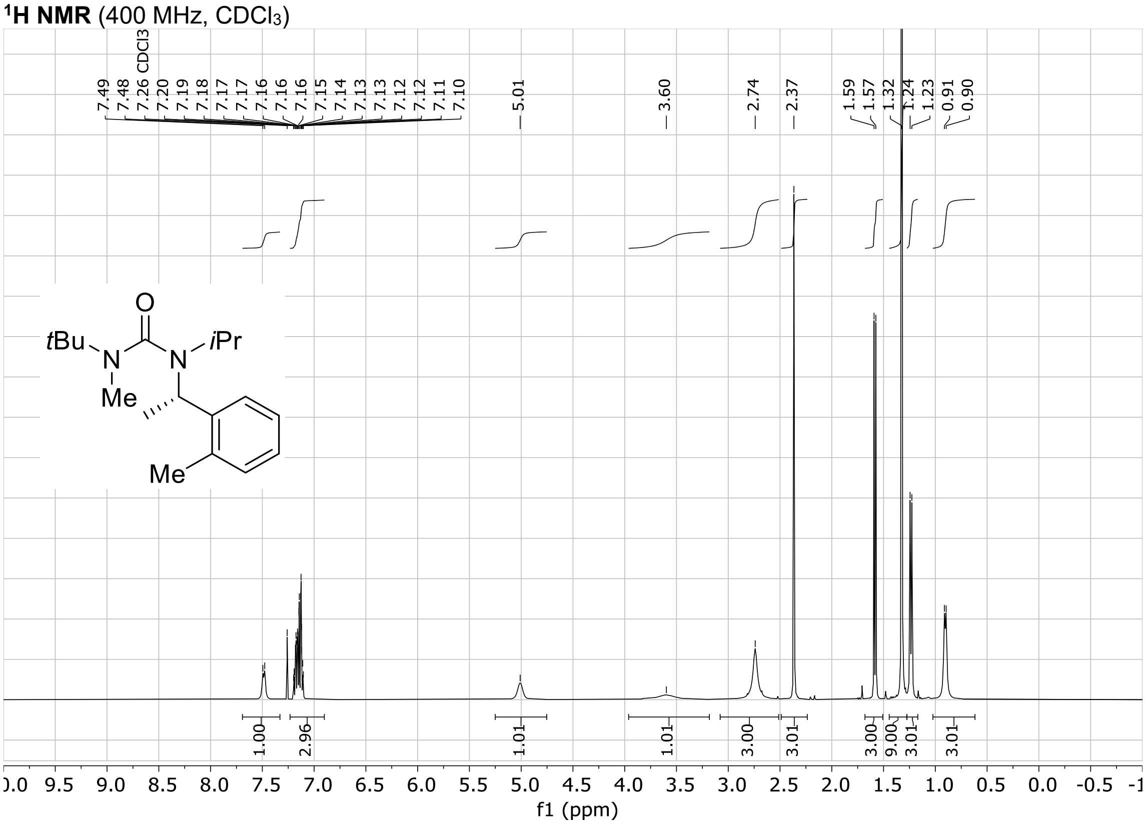

^{13}C NMR (101 MHz, $CDCl_3$)

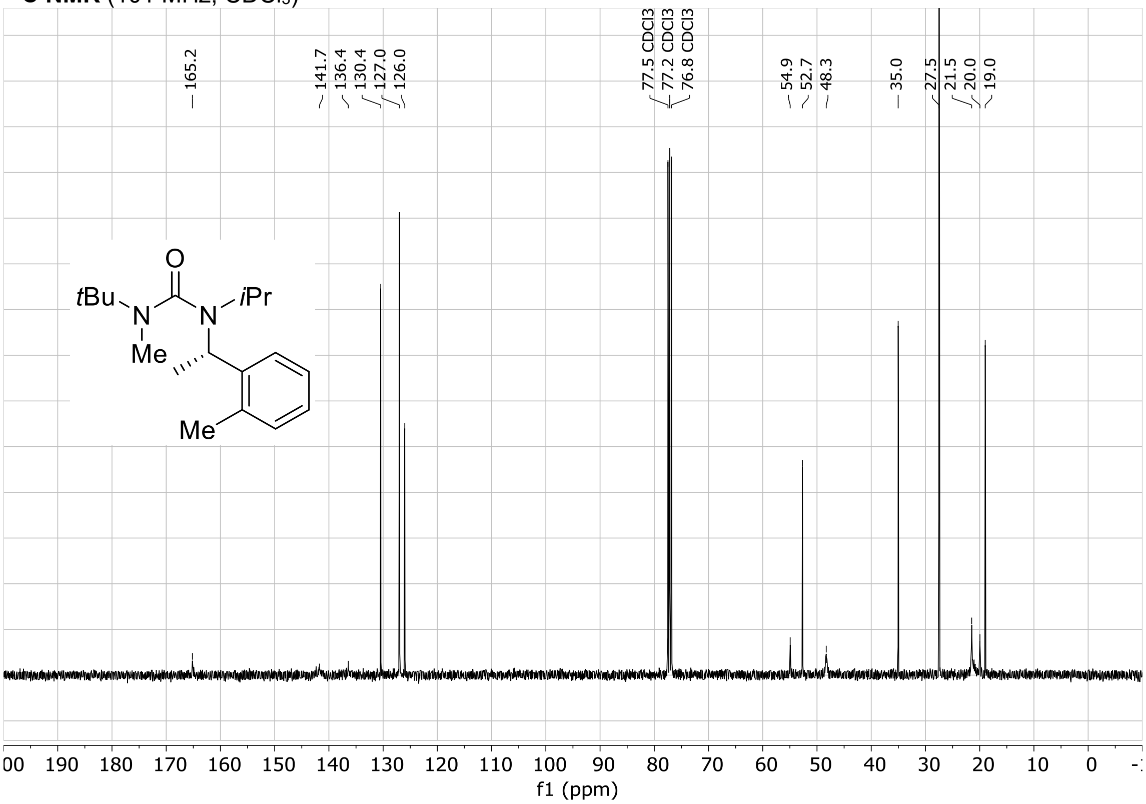

(*S*)-1-(*tert*-Butyl)-3-(1-(4-fluoro-2-methylphenyl)ethyl)-3-isopropyl-1-methylurea **7d**

^{1}H NMR (400 MHz, $CDCl_3$)

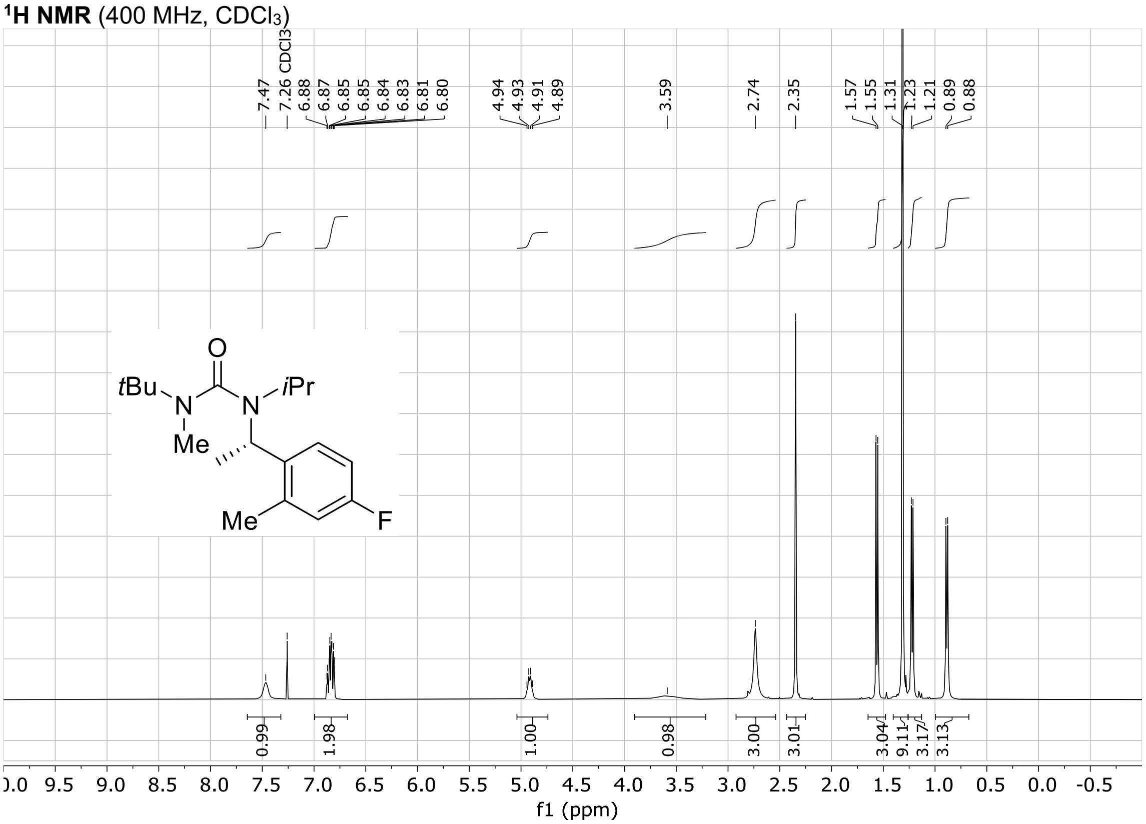

^{13}C NMR (126 MHz, $CDCl_3$)

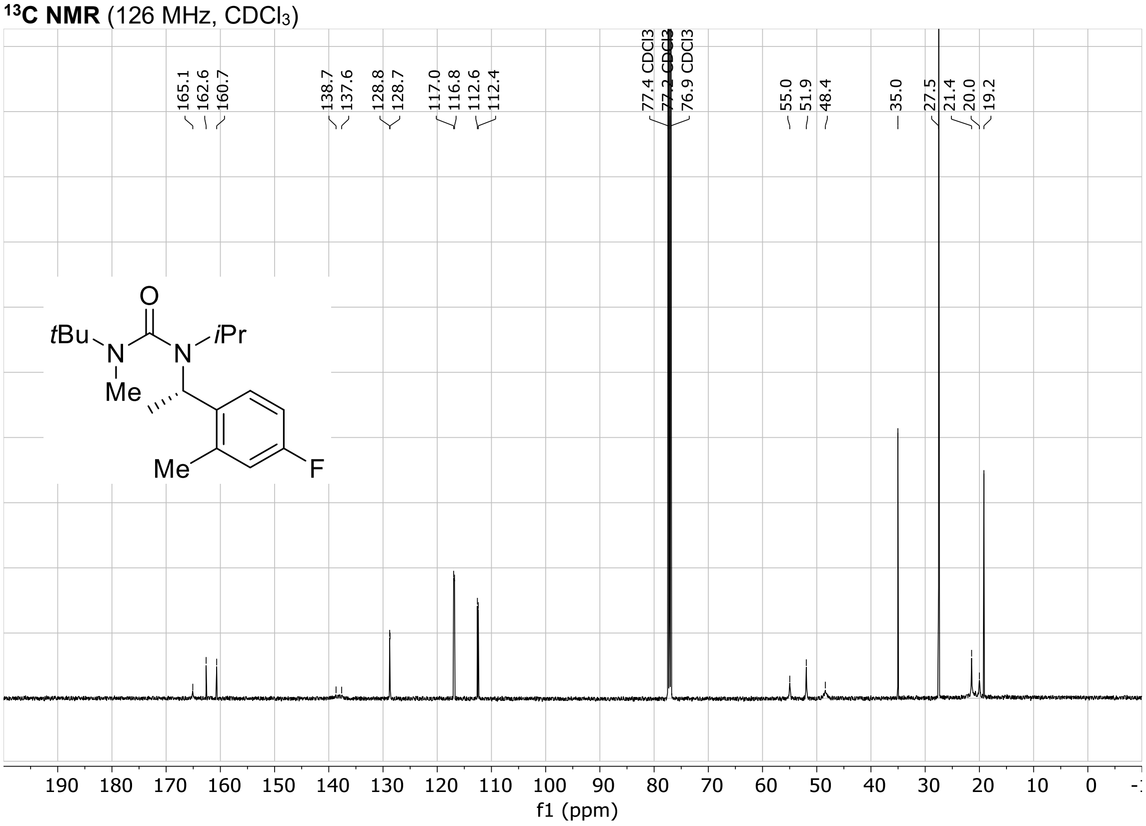

^{19}F NMR (377 MHz, $CDCl_3$)

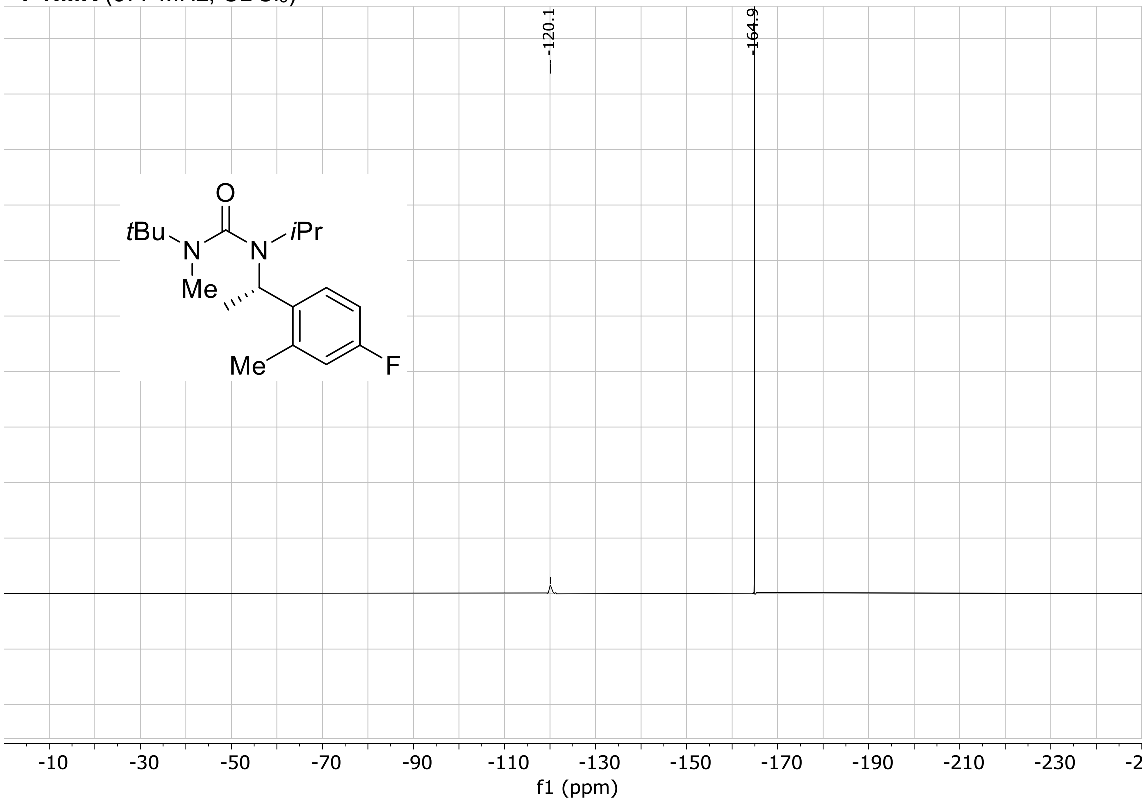

(*S*)-1-(*tert*-Butyl)-3-isopropyl-1-methyl-3-(1-(*p*-tolyl)ethyl)urea **7e**

^{1}H NMR (400 MHz, $CDCl_3$)

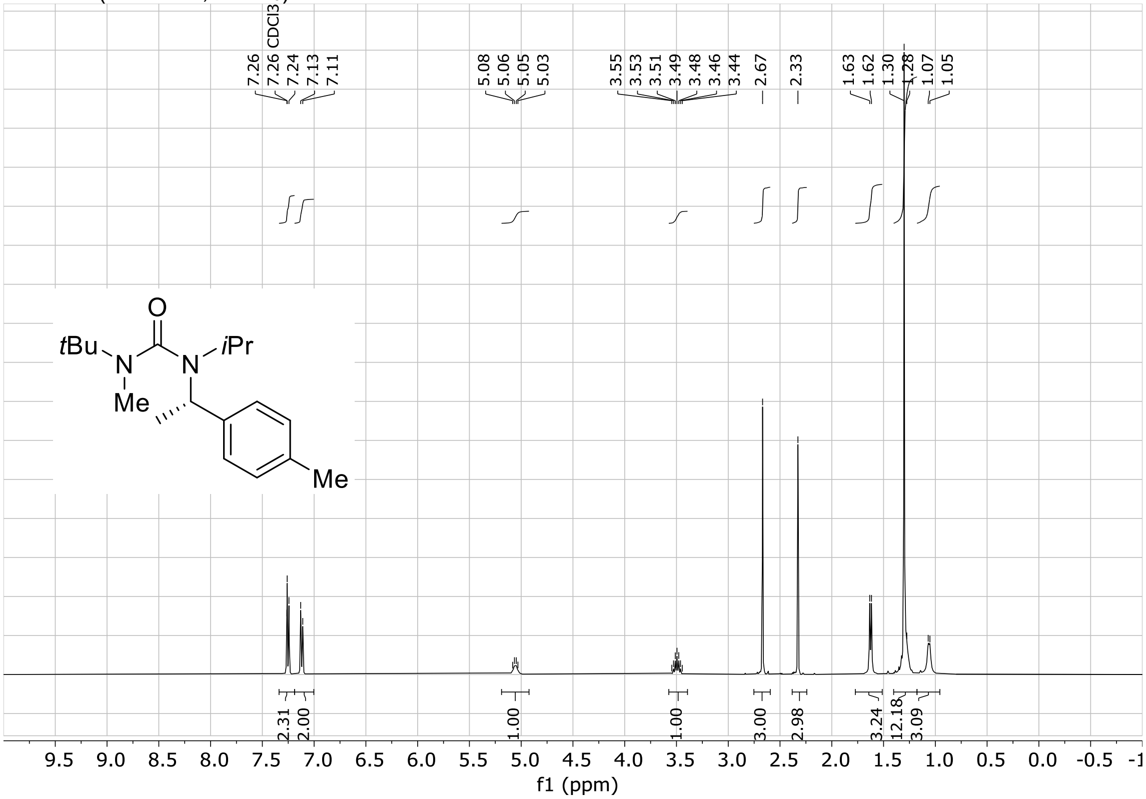

^{13}C NMR (126 MHz, $CDCl_3$)

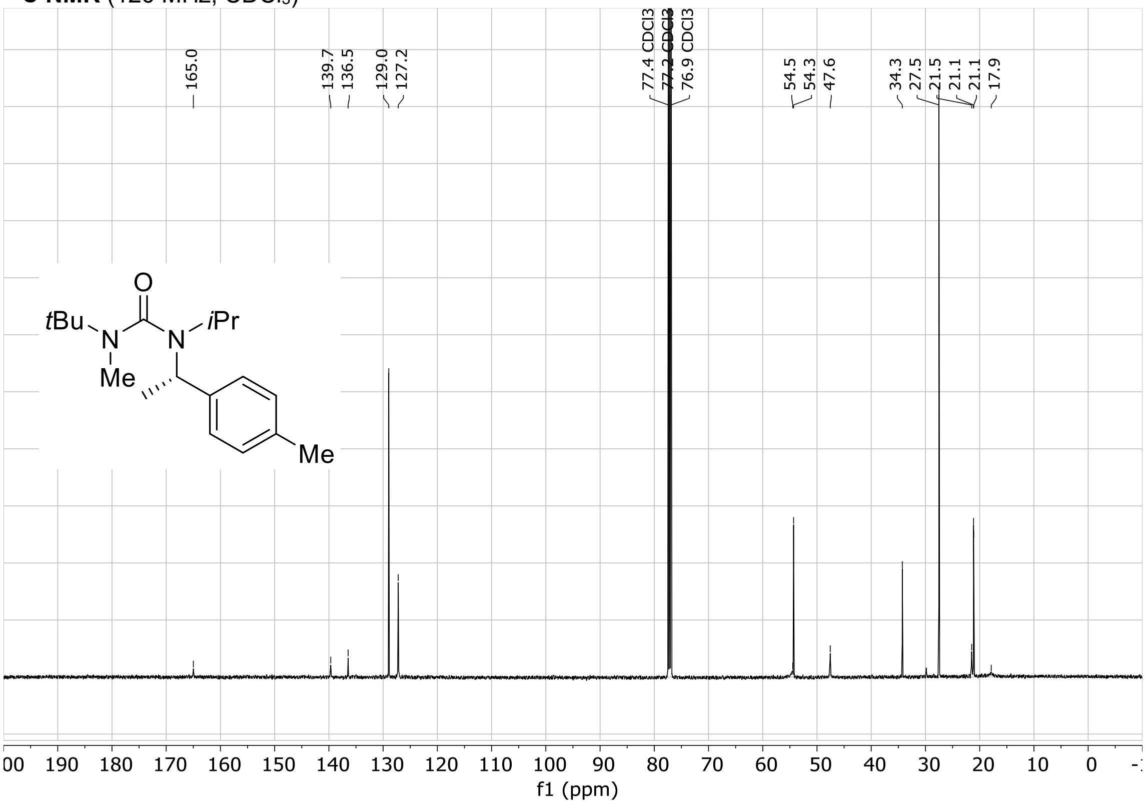

(*S*)-1-(*tert*-Butyl)-3-isopropyl-1-methyl-3-(1-(*m*-tolyl)ethyl)urea **7f**

^{1}H NMR (400 MHz, $CDCl_3$)

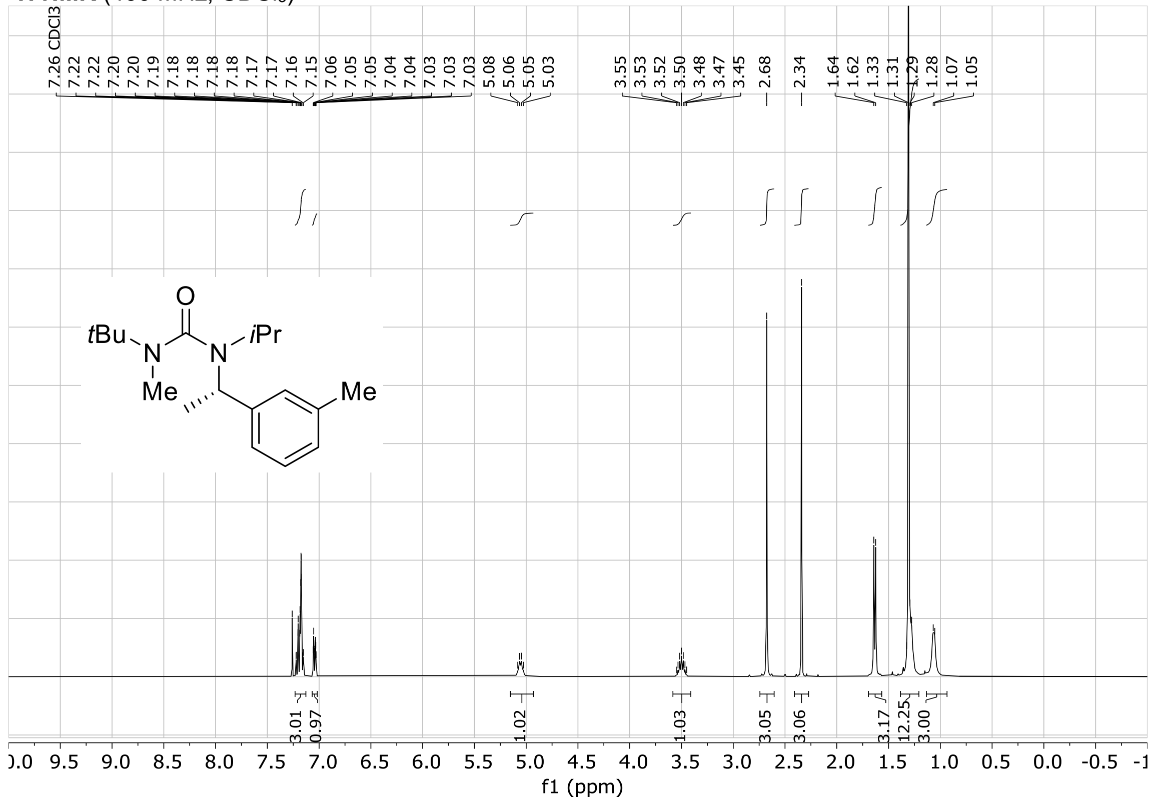

^{13}C NMR (101 MHz, $CDCl_3$)

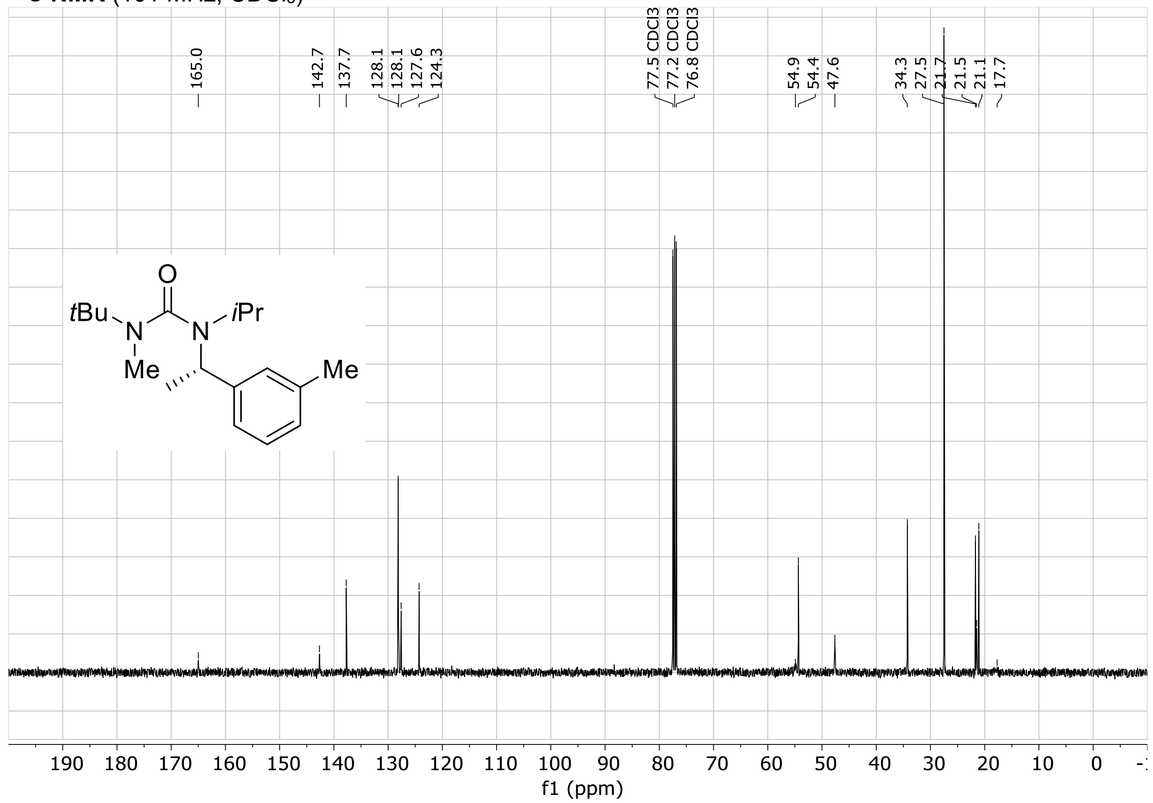

(*S*)-1-(*tert*-Butyl)-3-(1-(2-chlorophenyl)ethyl)-3-isopropyl-1-methylurea **7g**

^{1}H NMR (400 MHz, $CDCl_3$)

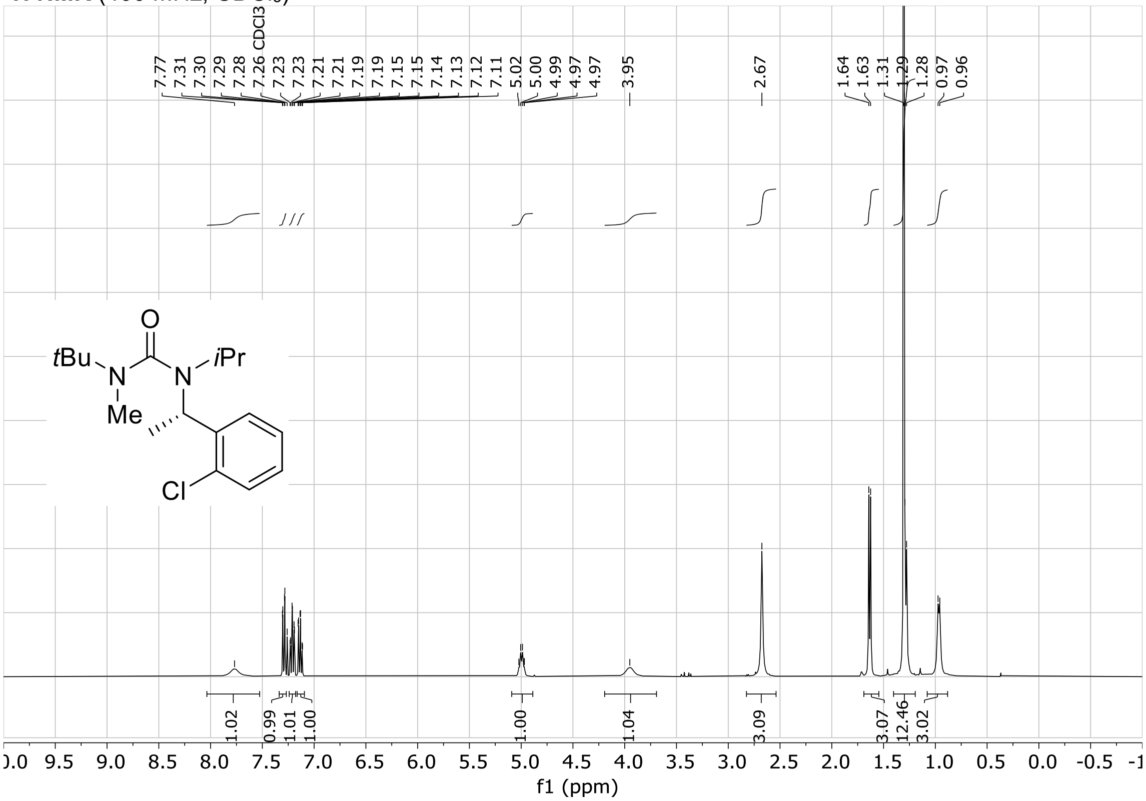

^{13}C NMR (101 MHz, $CDCl_3$)

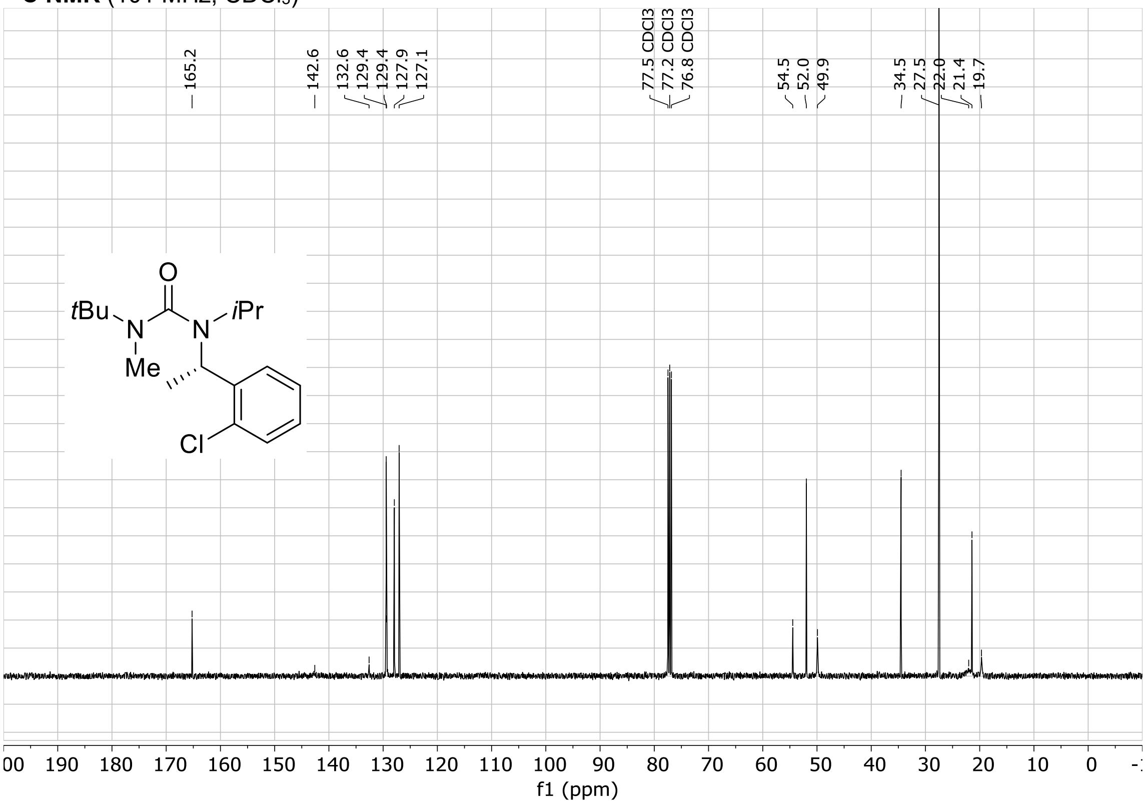

(*S*)-1-(1-(2-Bromophenyl)ethyl)-3-(*tert*-butyl)-1-isopropyl-3-methylurea **7h**

^{1}H NMR (400 MHz, $CDCl_3$)

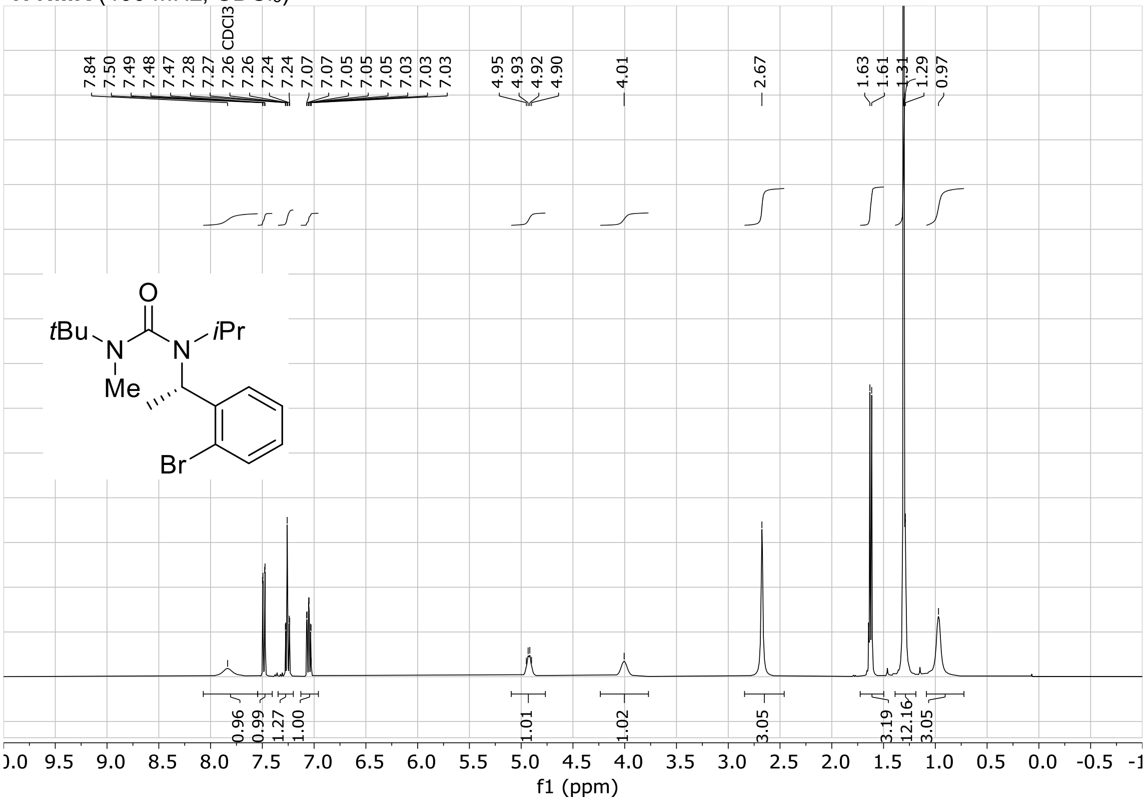

^{13}C NMR (101 MHz, $CDCl_3$)

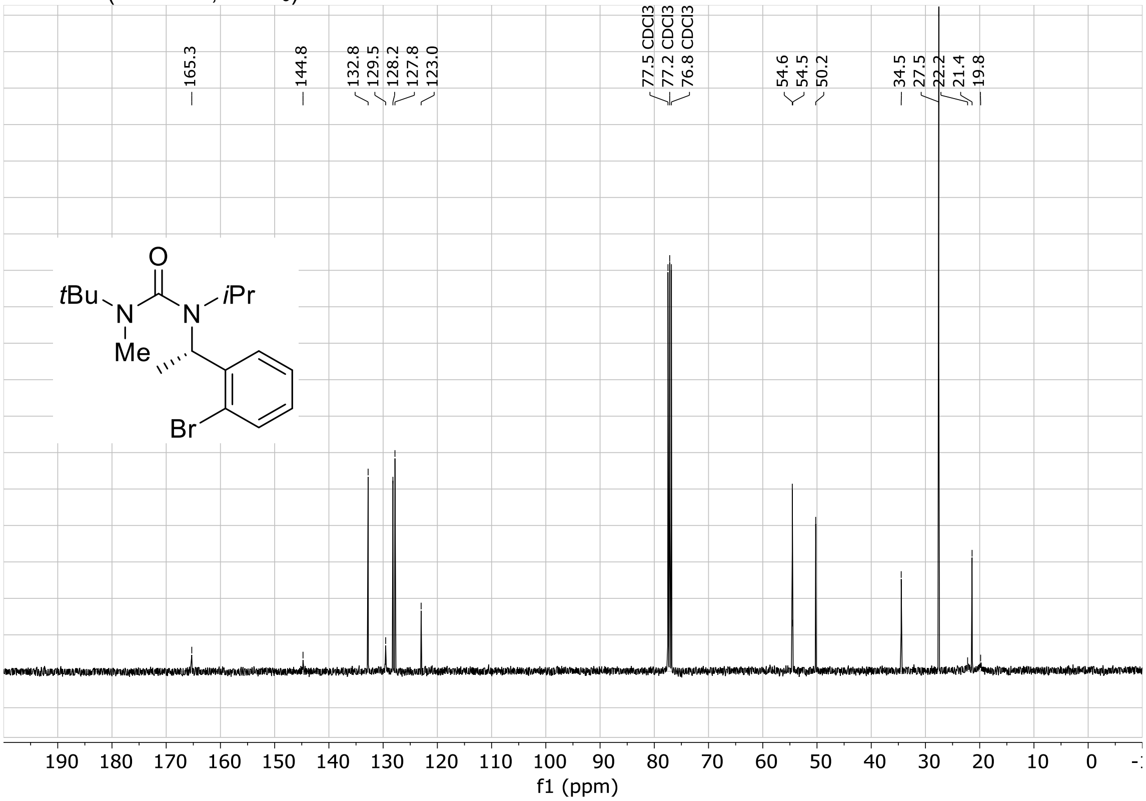

(*S*)-1-(1-(3-Bromophenyl)ethyl)-3-(*tert*-butyl)-1-isopropyl-3-methylurea **7i**

^{1}H NMR (400 MHz, $CDCl_3$)

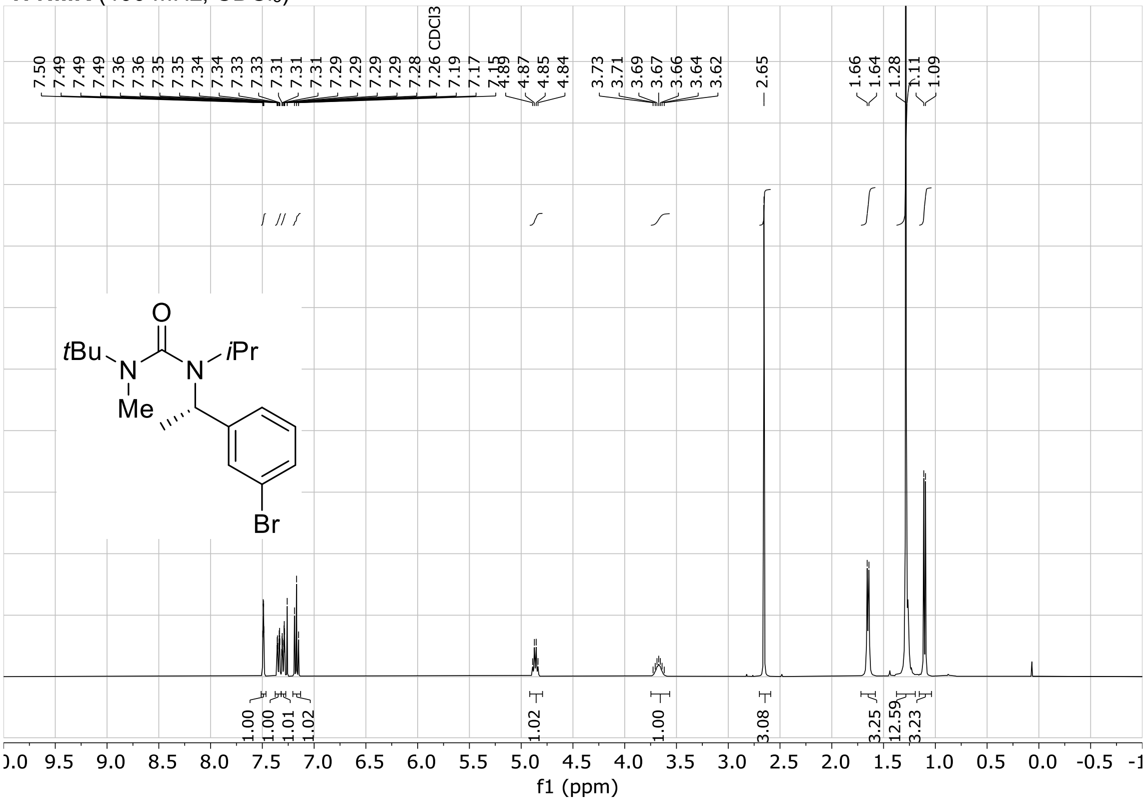

^{13}C NMR (101 MHz, $CDCl_3$)

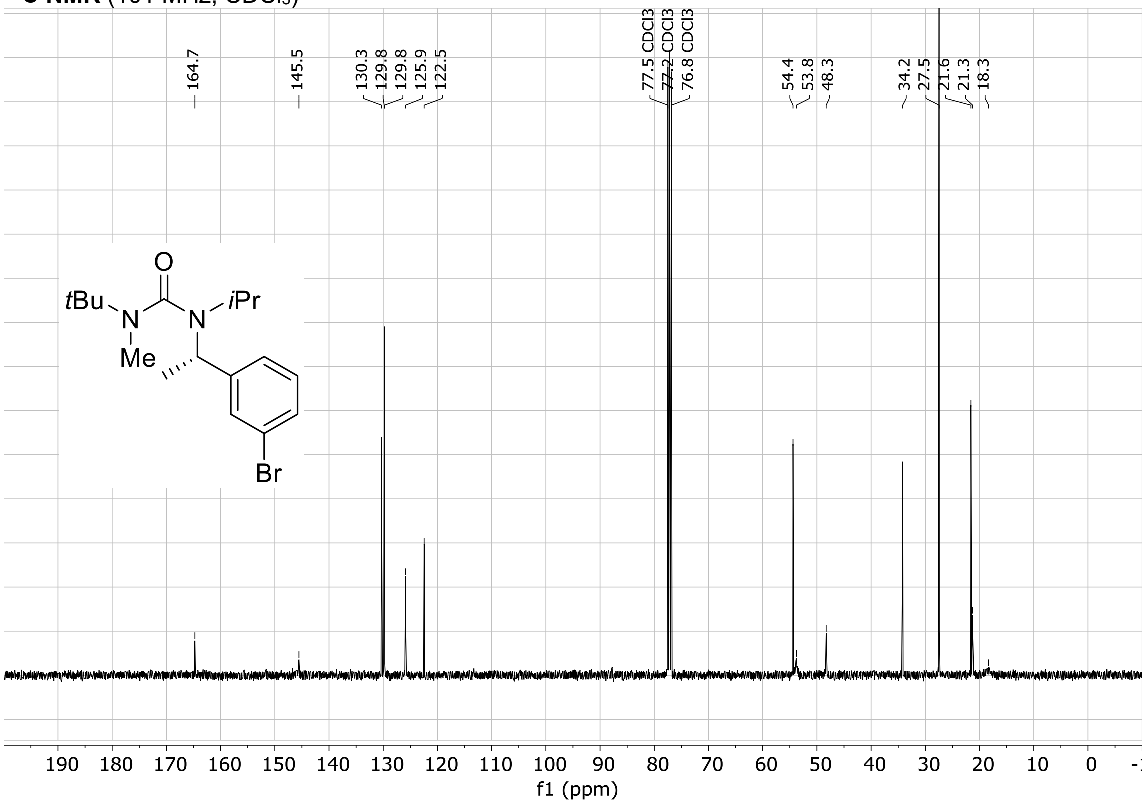

(*S*)-1-(*tert*-Butyl)-3-isopropyl-3-(1-(2-methoxyphenyl)ethyl)-1-methylurea **7j**

^{1}H NMR (400 MHz, $CDCl_3$)

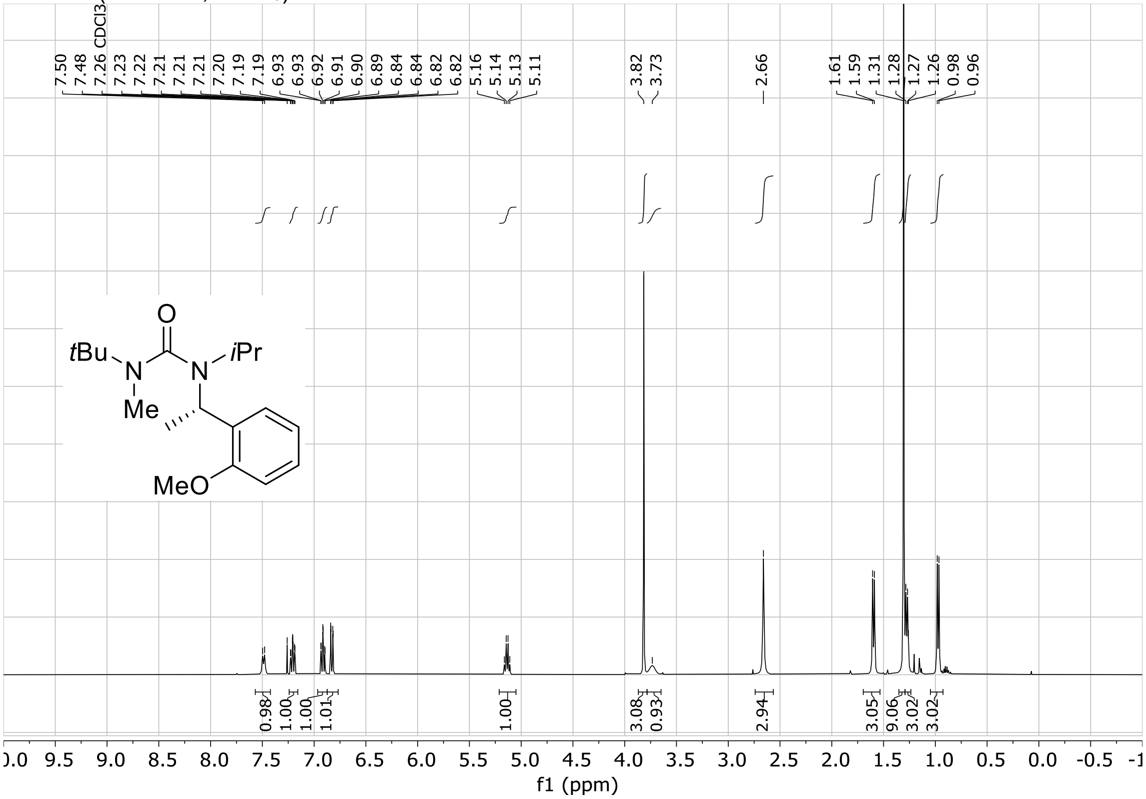

^{13}C NMR (101 MHz, $CDCl_3$)

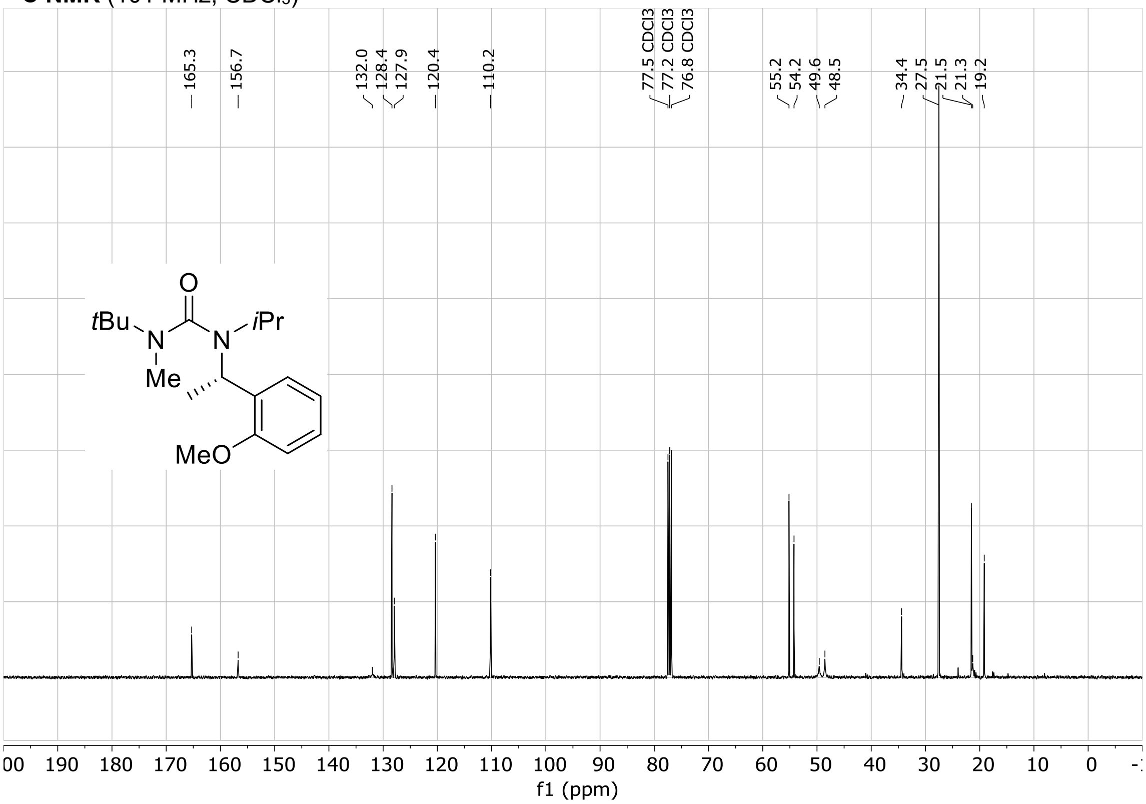

(*S*)-1-(*tert*-Butyl)-3-isopropyl-1-methyl-3-(1-(4-methoxynaphthalen-1-yl)ethyl)urea **7k**

^{1}H NMR (400 MHz, $CDCl_3$)

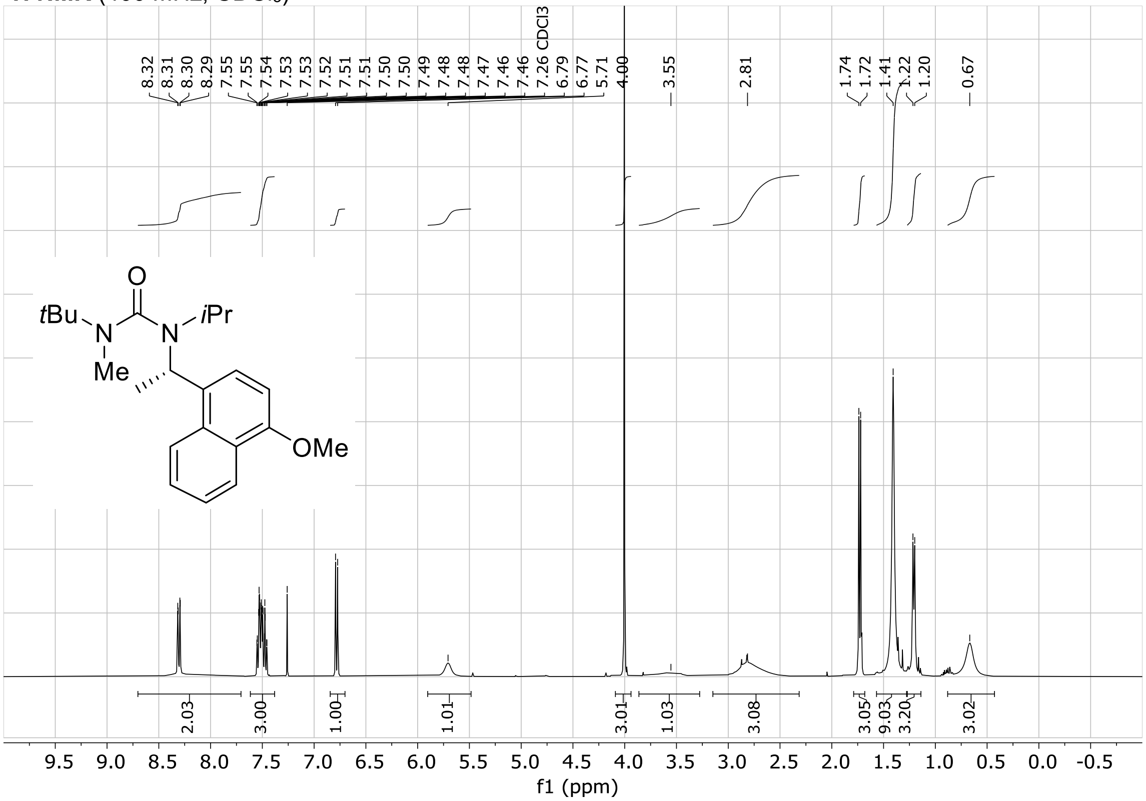

^{13}C NMR (126 MHz, $CDCl_3$)

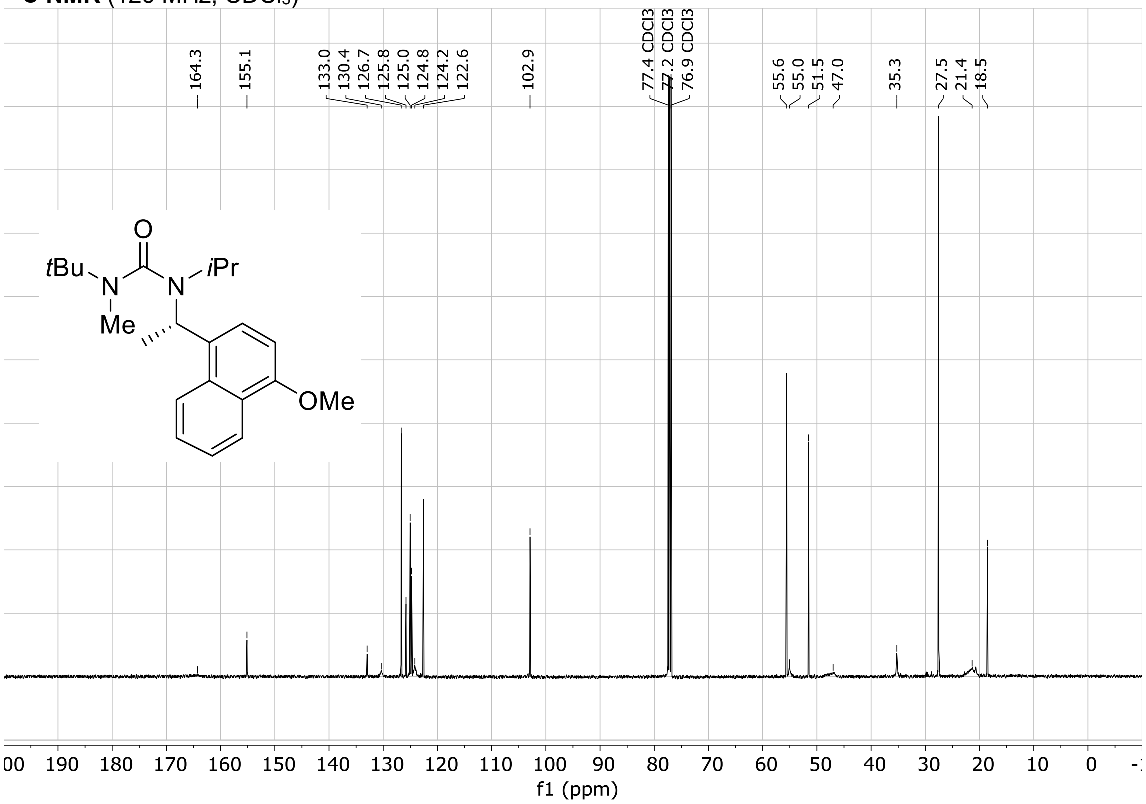

(*S*)-1-(*tert*-Butyl)-3-isopropyl-1-methyl-3-(1-(4-methylnaphthalen-1-yl)ethyl)urea **7l**

^{1}H NMR (400 MHz, $CDCl_3$)

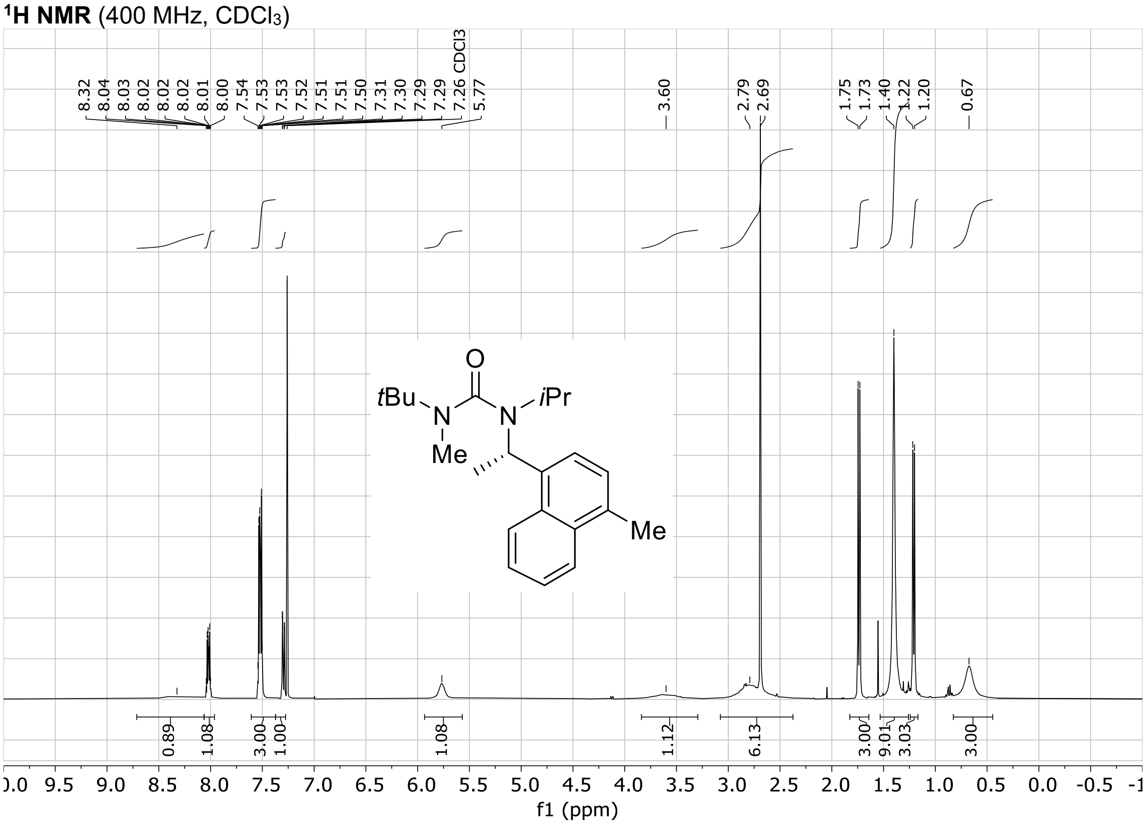

^{13}C NMR (126 MHz, $CDCl_3$)

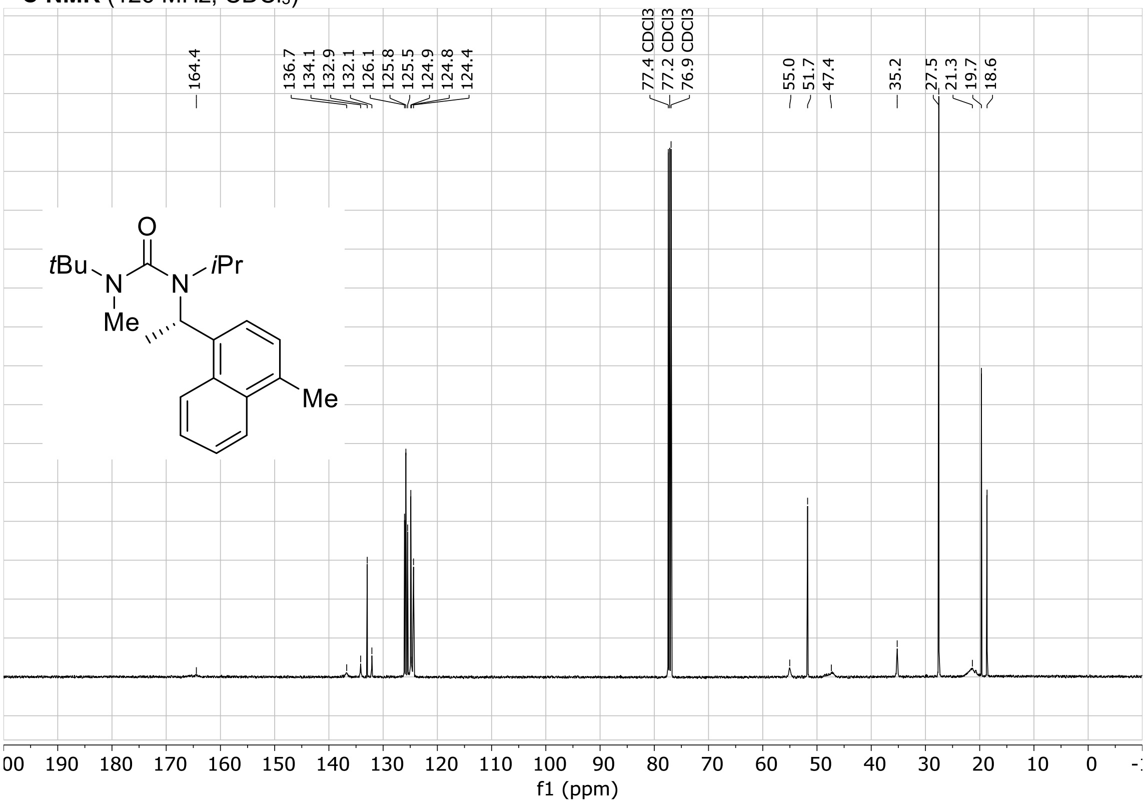

(*S*)-1-(*tert*-Butyl)-3-(dicyclopropylmethyl)-1-methyl-3-(1-phenylethyl)urea **9a**

^{1}H NMR (400 MHz, $CDCl_3$)

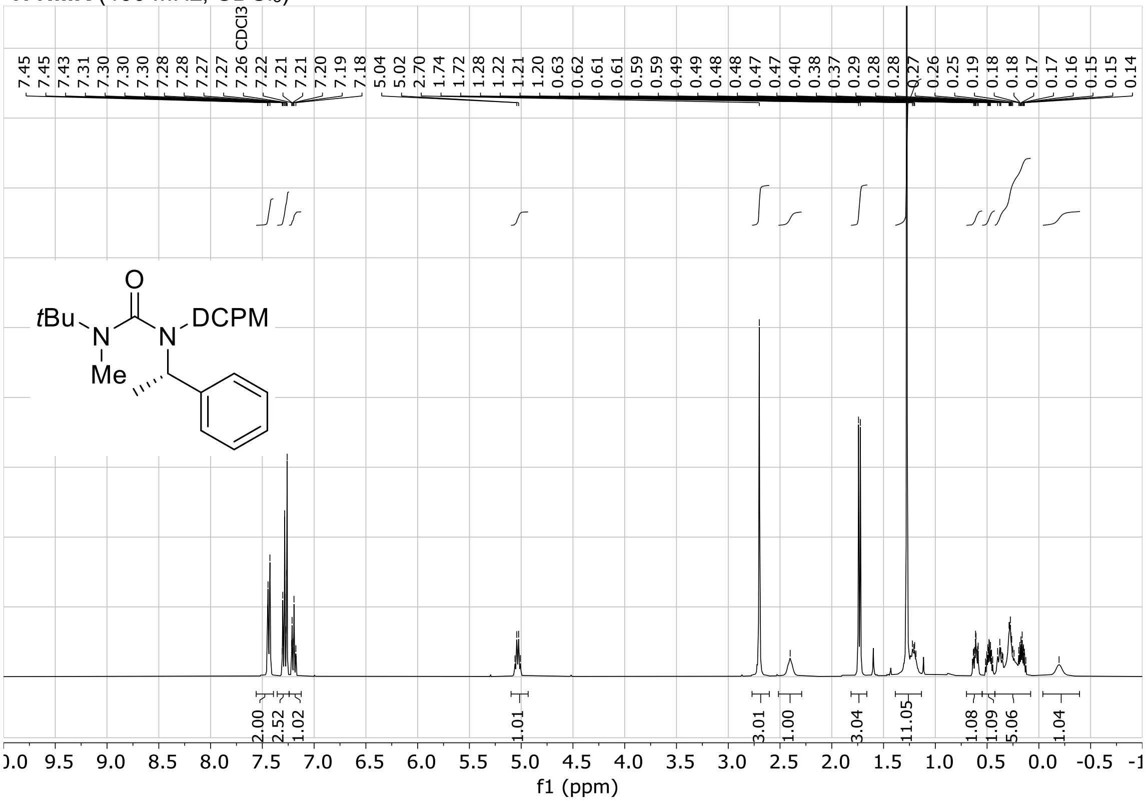

^{13}C NMR (101 MHz, $CDCl_3$)

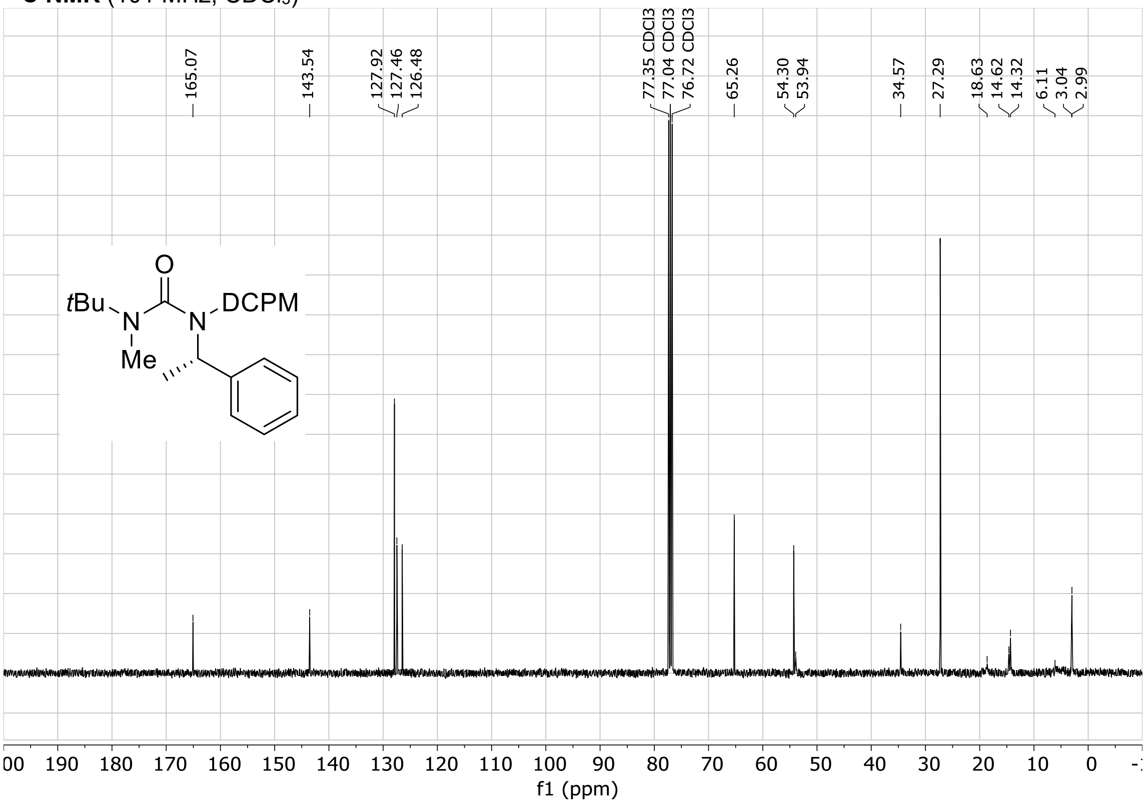

(*S*)-1-(1-(2-Bromophenyl)ethyl)-3-(*tert*-butyl)-1-(dicyclopropylmethyl)-3-methylurea **9b**

^{1}H NMR (400 MHz, $CDCl_3$)

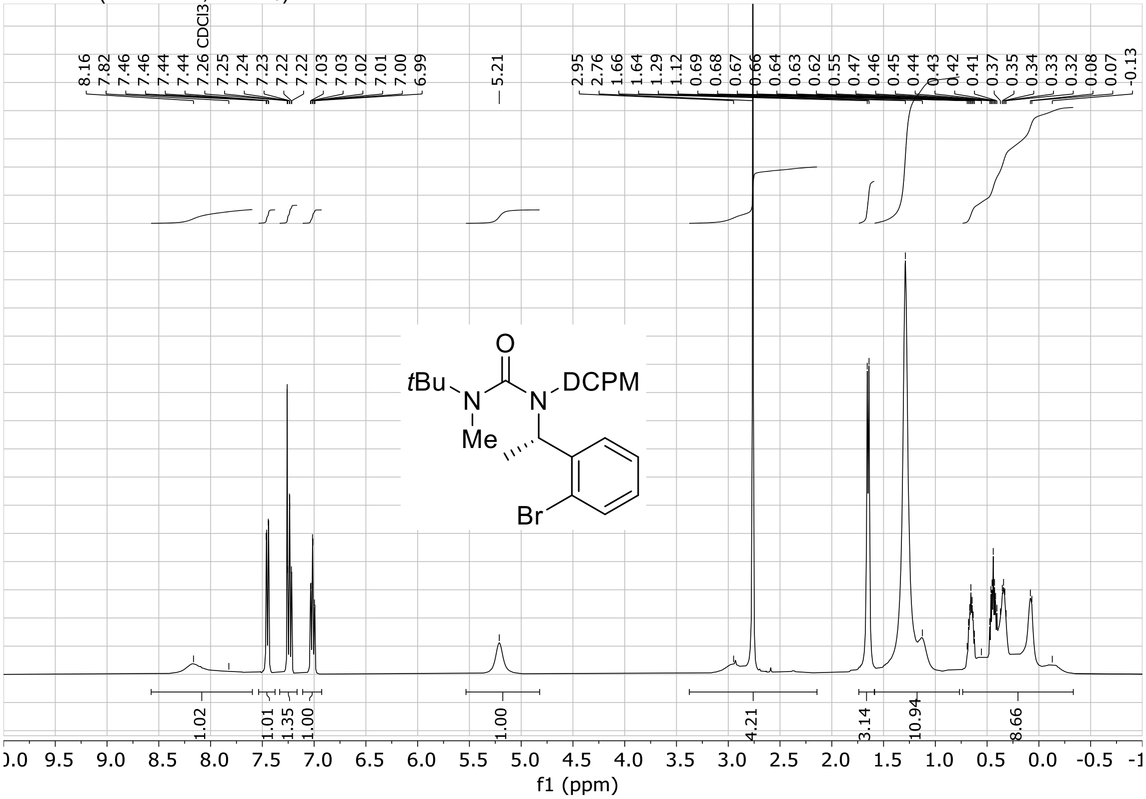

^{13}C NMR (151 MHz, $CDCl_3$)

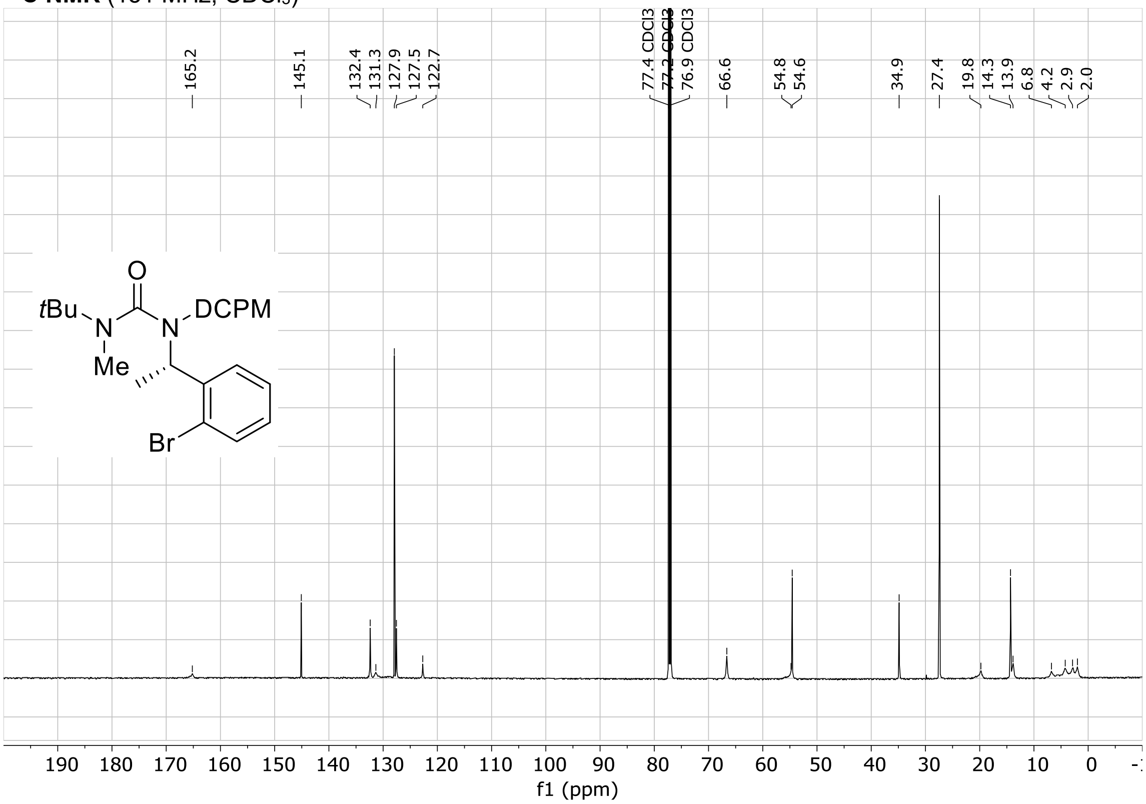

(*S*)-1-(*tert*-Butyl)-3-(dicyclopropylmethyl)-1-methyl-3-(1-(o-tolyl)ethyl)urea **9c**

^{1}H NMR (400 MHz, $CDCl_3$)

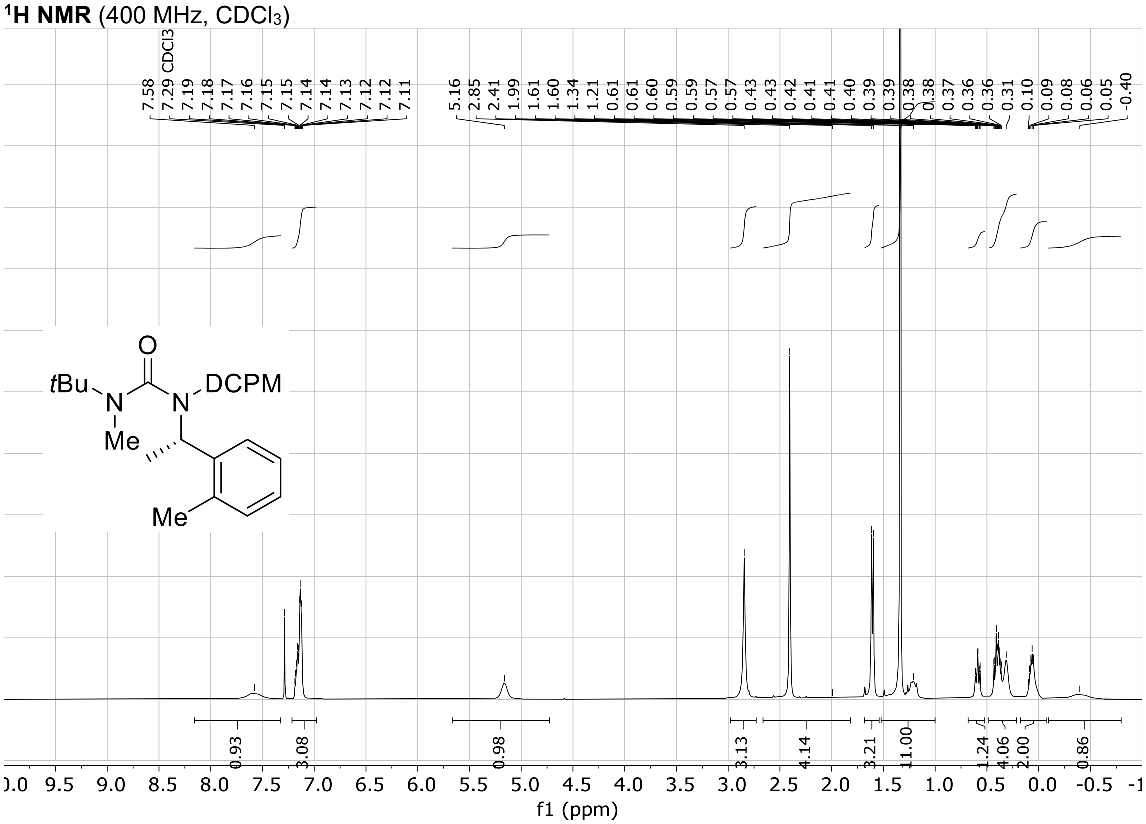

^{13}C NMR (151 MHz, $CDCl_3$)

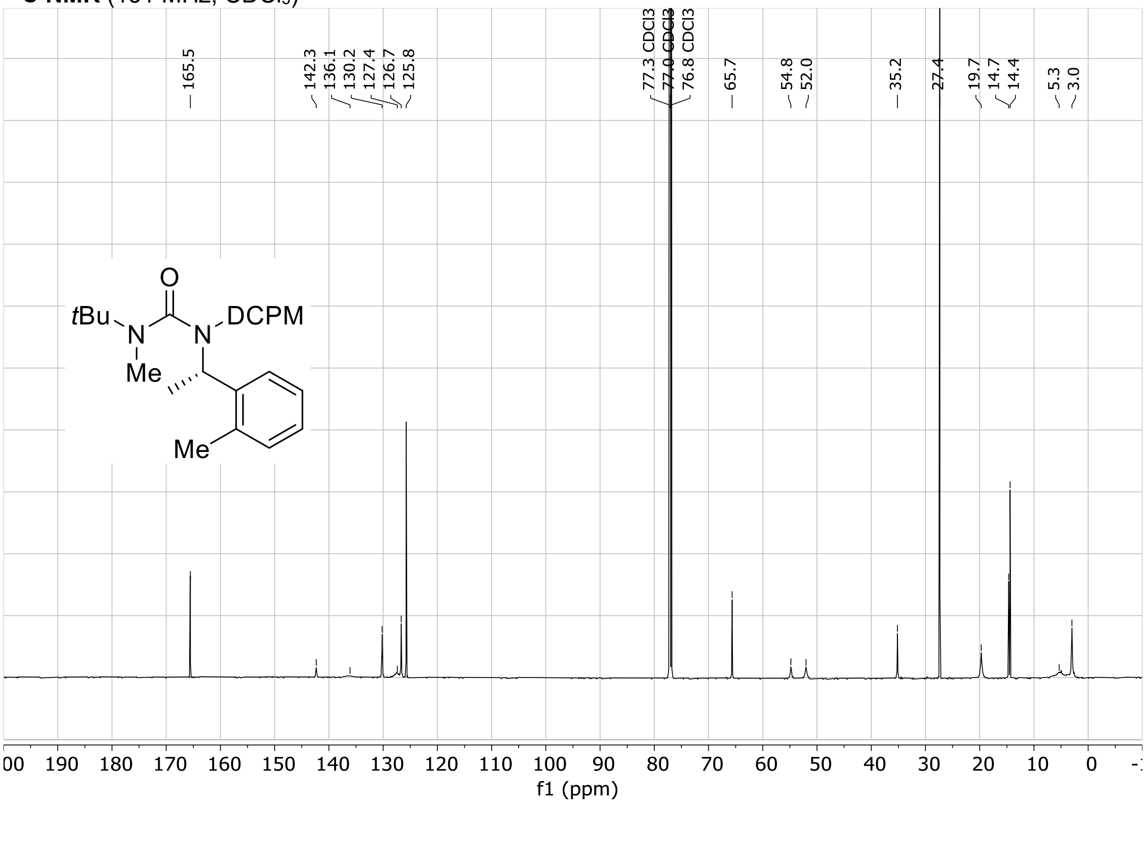

(*R*)-1-(*tert*-Butyl)-3-(dicyclopropylmethyl)-3-((1-hydroxycyclobutyl)(phenyl)methyl)-1-methylurea

9d

^{1}H NMR (600 MHz, $CDCl_3$)

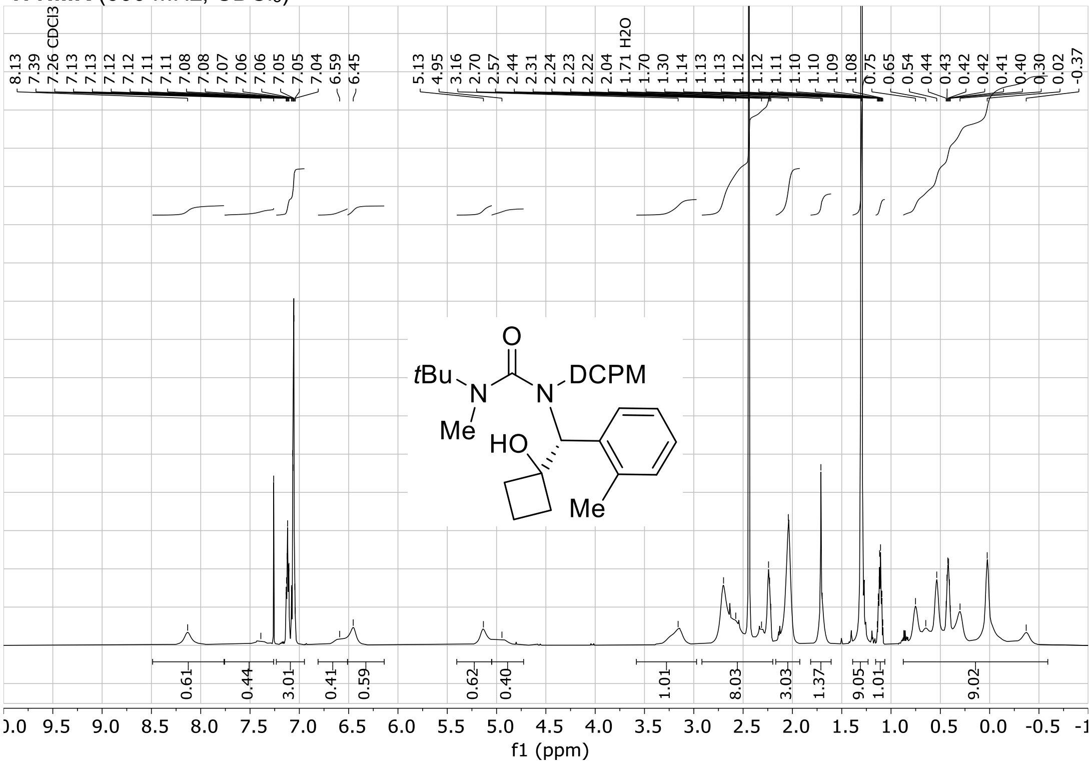

^{13}C NMR (151 MHz, $CDCl_3$)

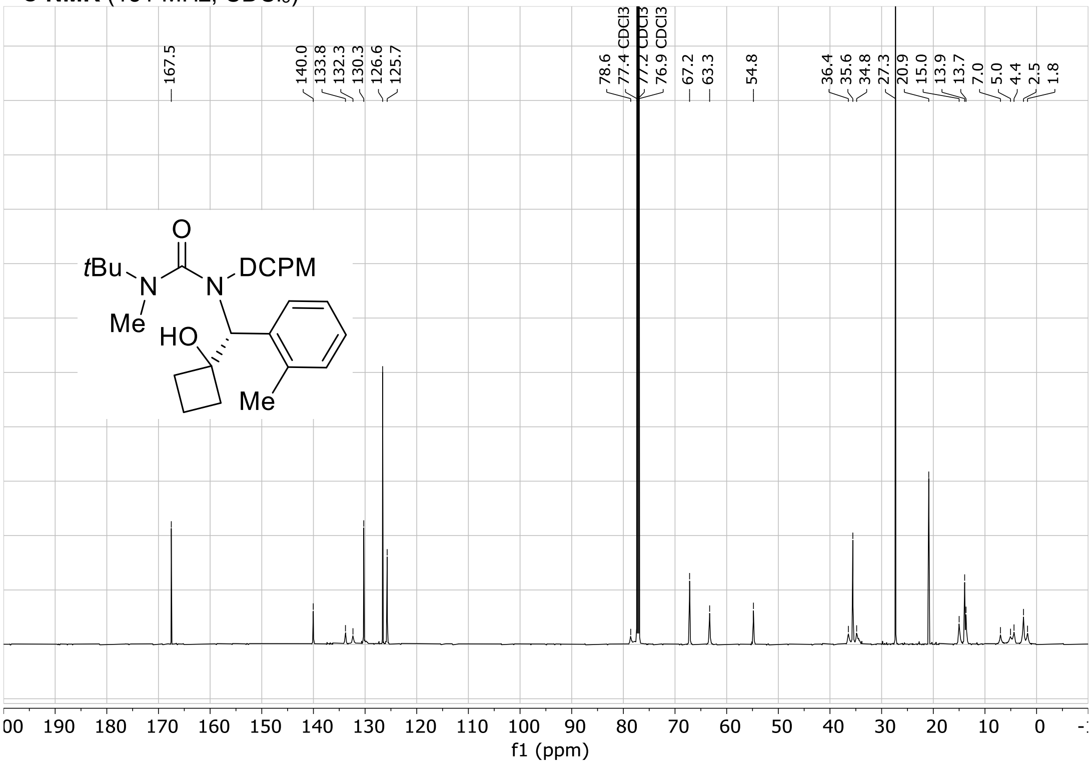

(*S*)-1-(*tert*-Butyl)-3-(dicyclopropylmethyl)-1-methyl-3-(1-(naphthalen-1-yl)ethyl)urea **9e**

^{1}H NMR (400 MHz, $CDCl_3$)

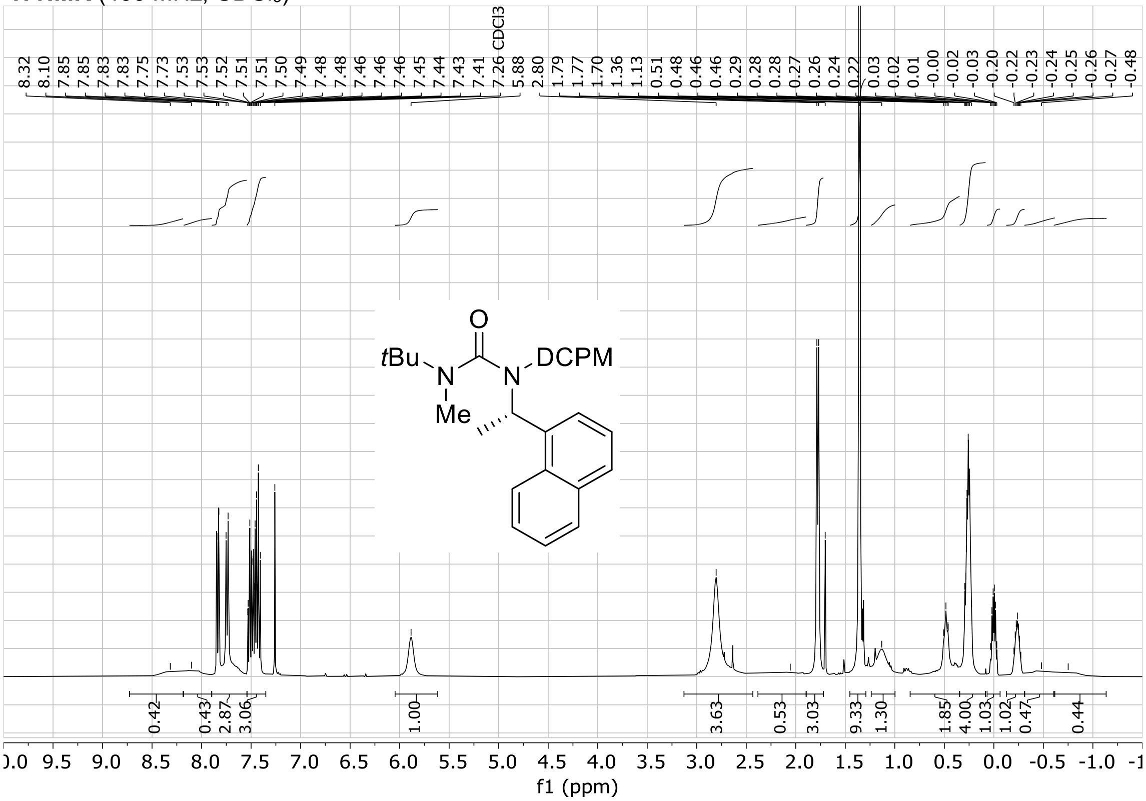

^{13}C NMR (101 MHz, $CDCl_3$)

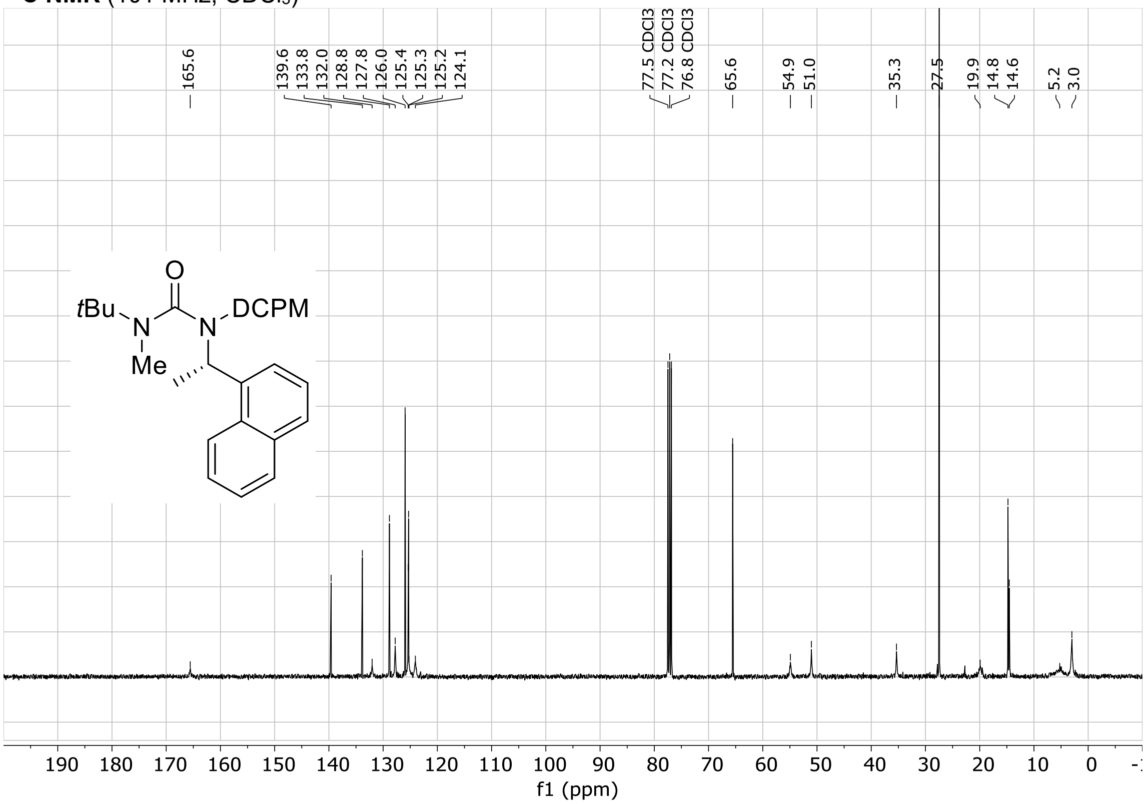

(*S*)-1-(*tert*-Butyl)-3-(dicyclopropylmethyl)-1-methyl-3-(phenyl(trimethylsilyl)methyl)urea **11**

^{1}H NMR (400 MHz, $CDCl_3$)

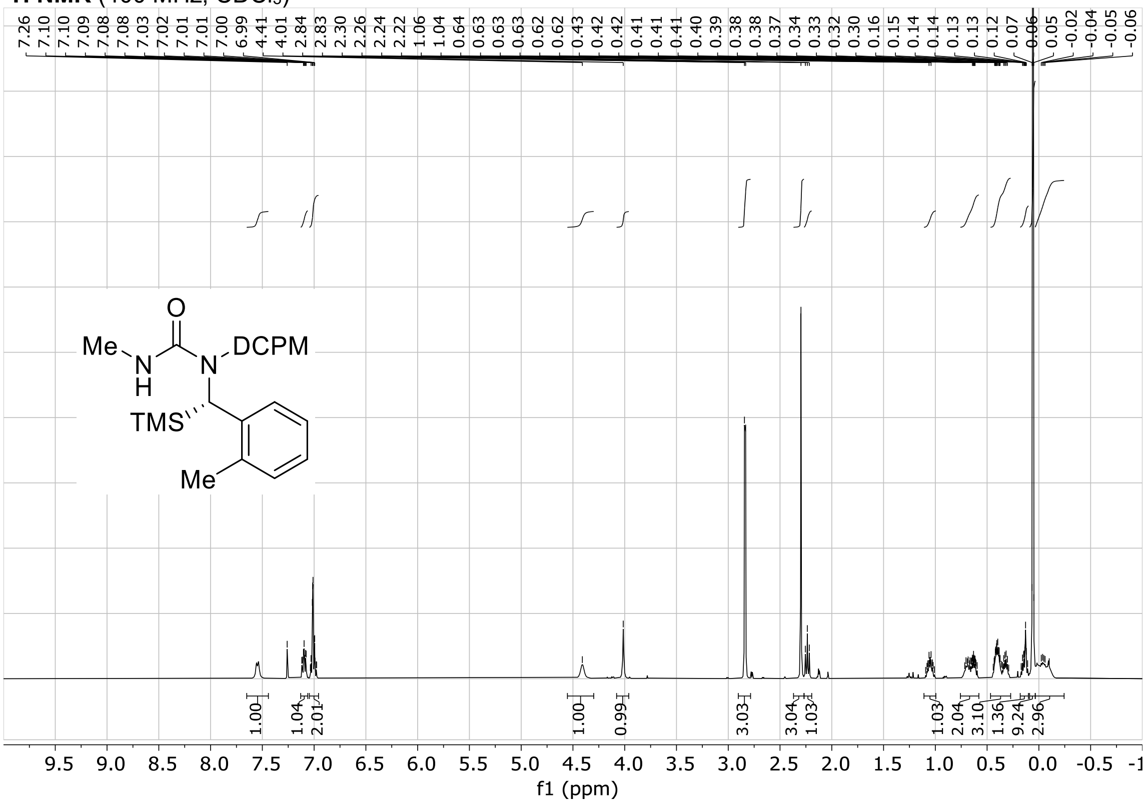

^{13}C NMR (101 MHz, $CDCl_3$)

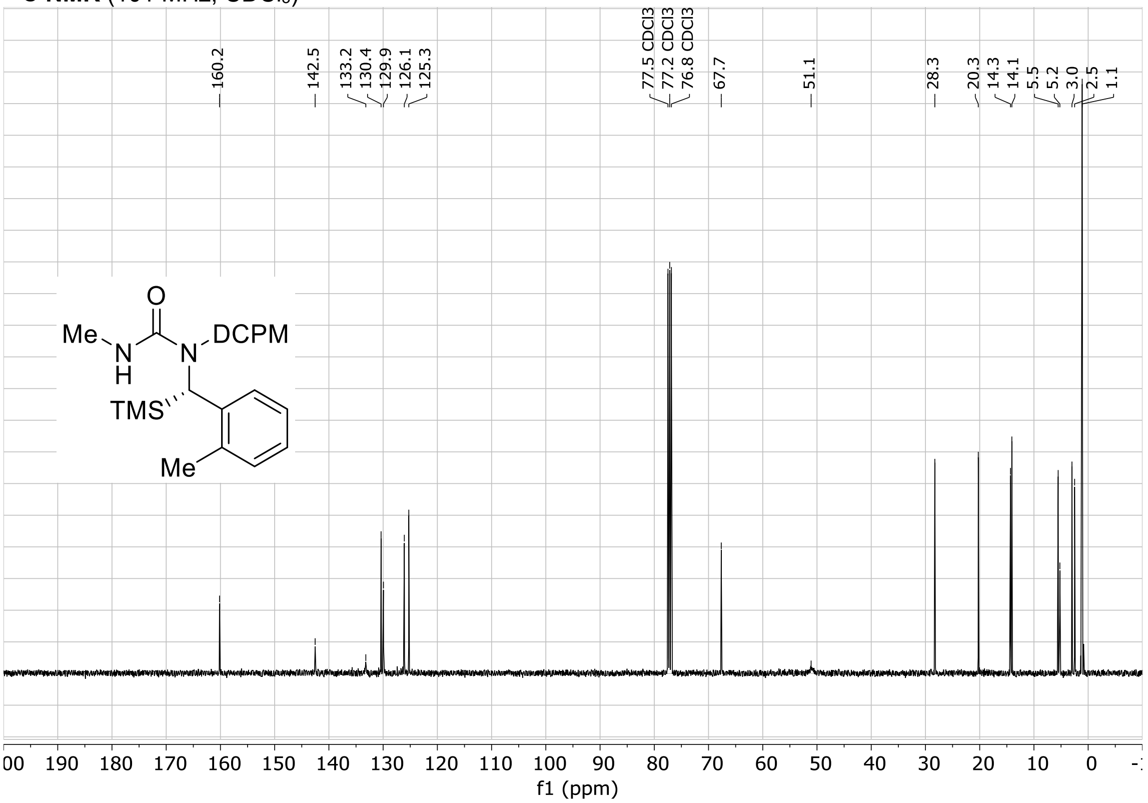

2.1.3 Products from conversion to benzylamine derivatives

(*S*)-1-(1-(2-Chlorophenyl)ethyl)-1-isopropyl-3-methylurea **SI-3**

^{1}H NMR (400 MHz, $CDCl_3$)

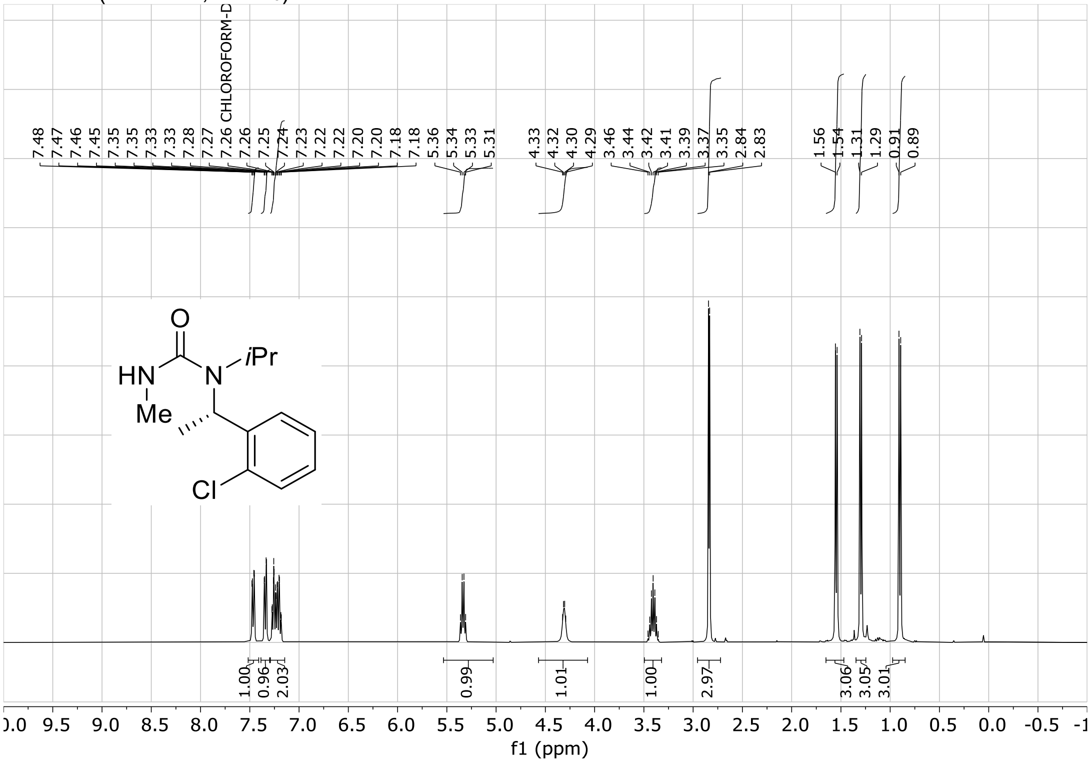

^{13}C NMR (101 MHz, $CDCl_3$)

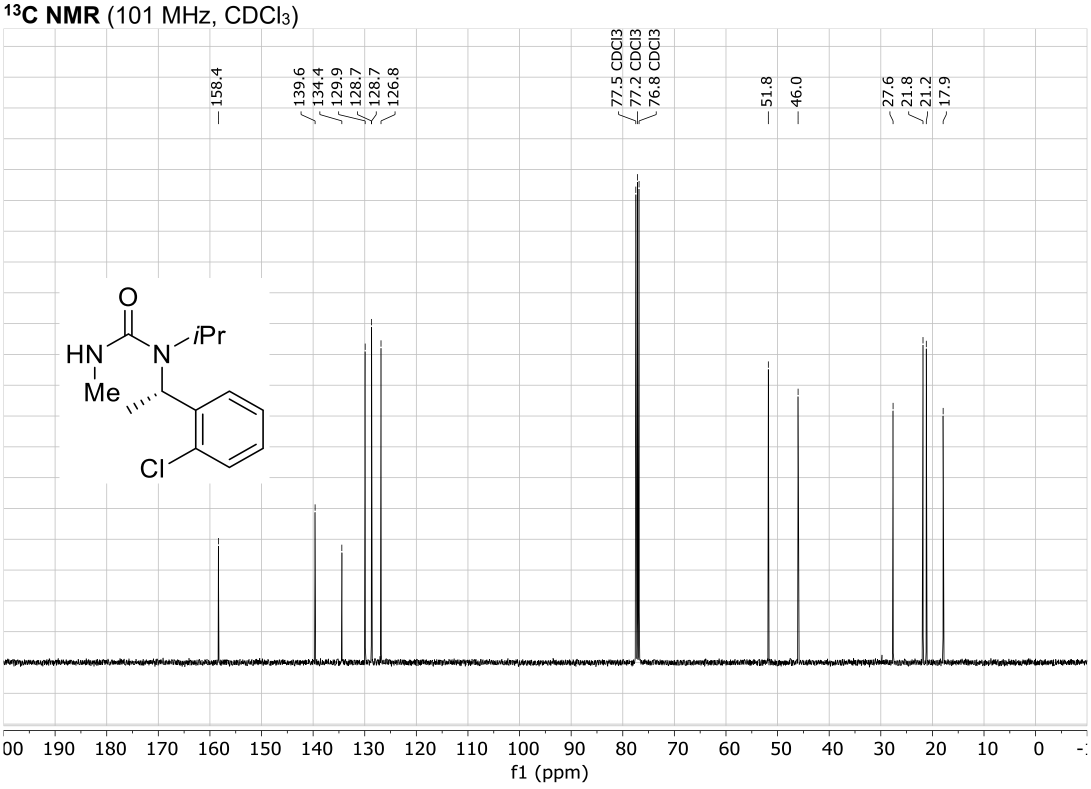

(*S*)-*N*-(1-(2-Chlorophenyl)ethyl)propan-2-amine **12**

^{1}H NMR (600 MHz, $CDCl_3$)

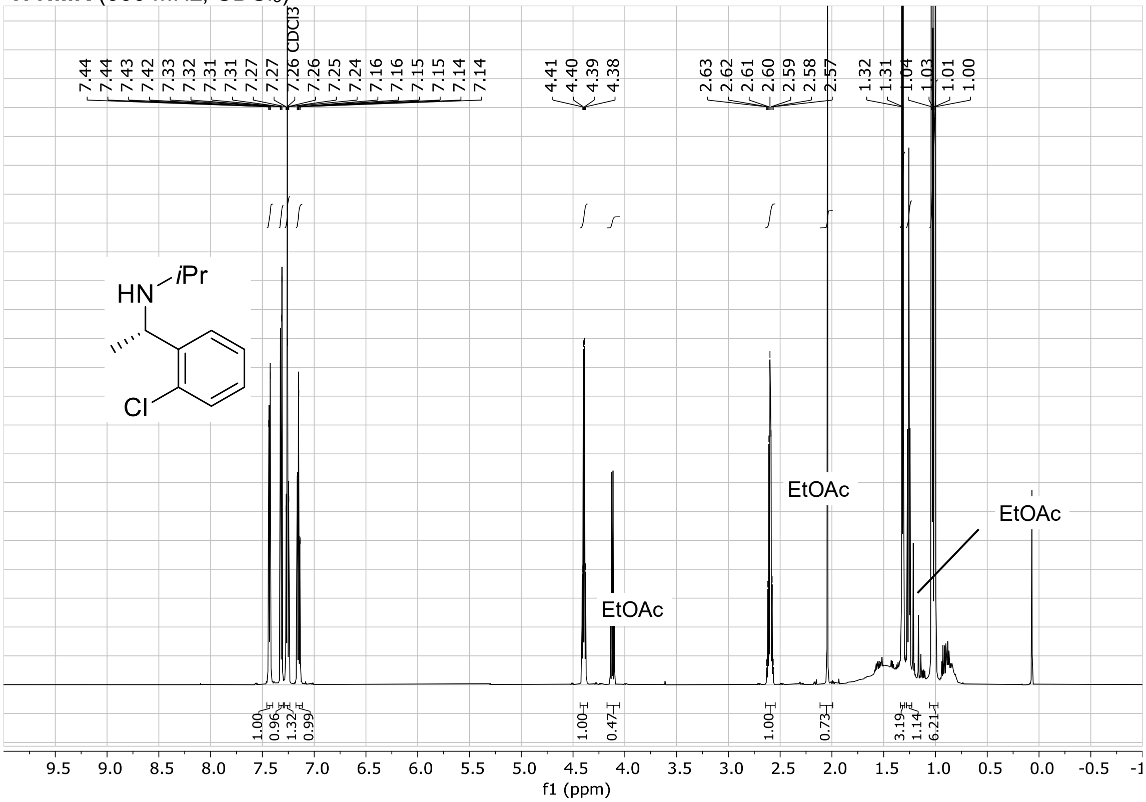

^{13}C NMR (151 MHz, $CDCl_3$)

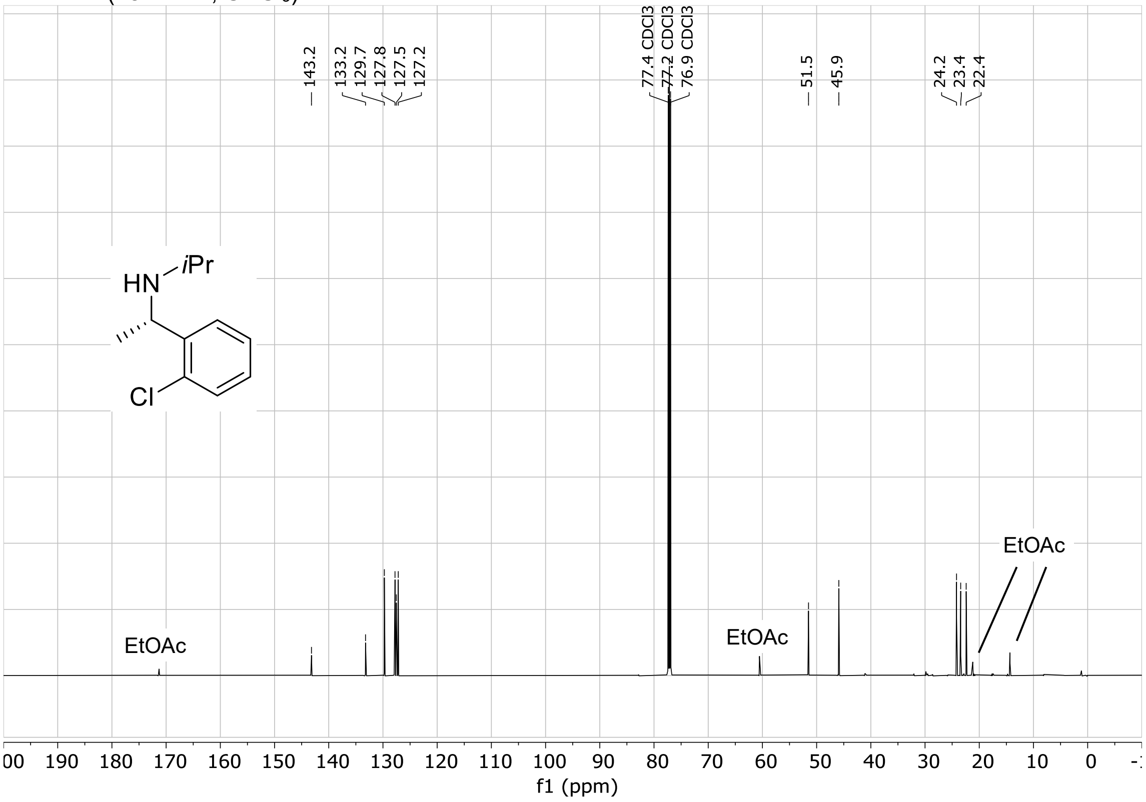

(*S*)-1-(*tert*-Butyl)-1-methyl-3-(1-(naphthalen-1-yl)ethyl)urea **13**

^{1}H NMR (400 MHz, $CDCl_3$)

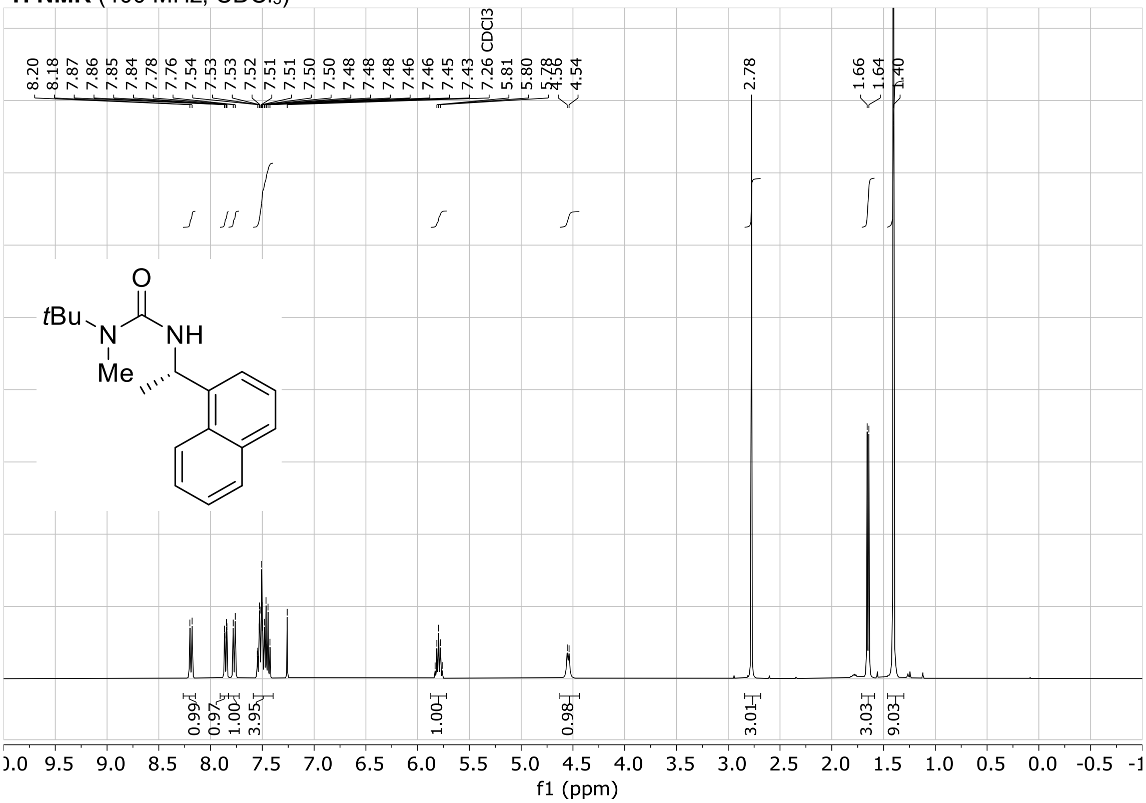

^{13}C NMR (101 MHz, $CDCl_3$)

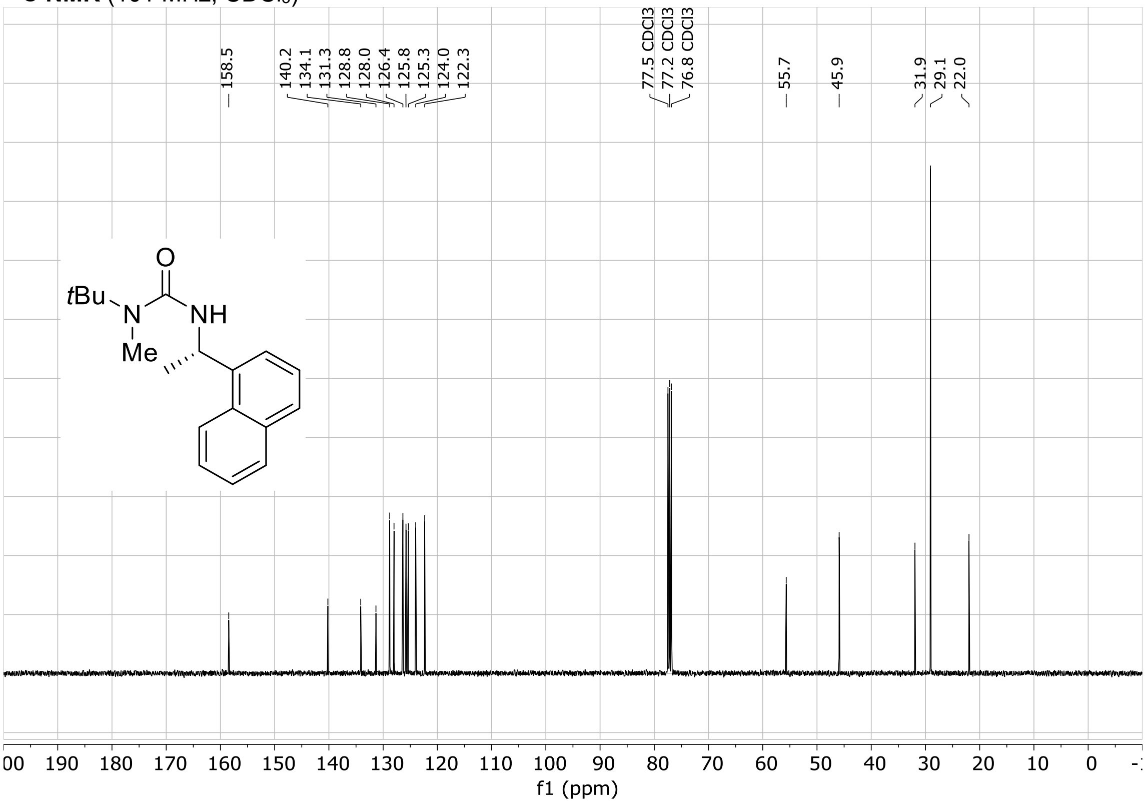

tert-Butyl (*S*)-(1-(naphthalen-1-yl)ethyl)carbamate **14**

¹H NMR (400 MHz, CDCl₃)

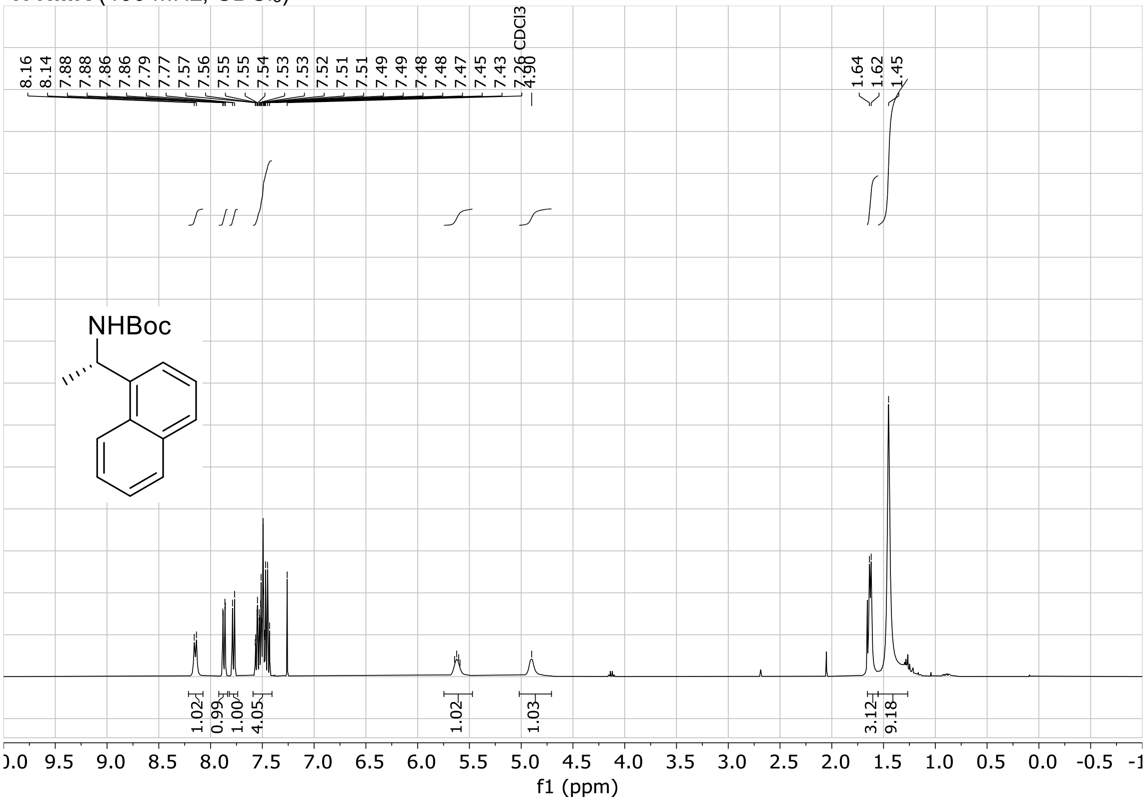

¹³C NMR (101 MHz, CDCl₃)

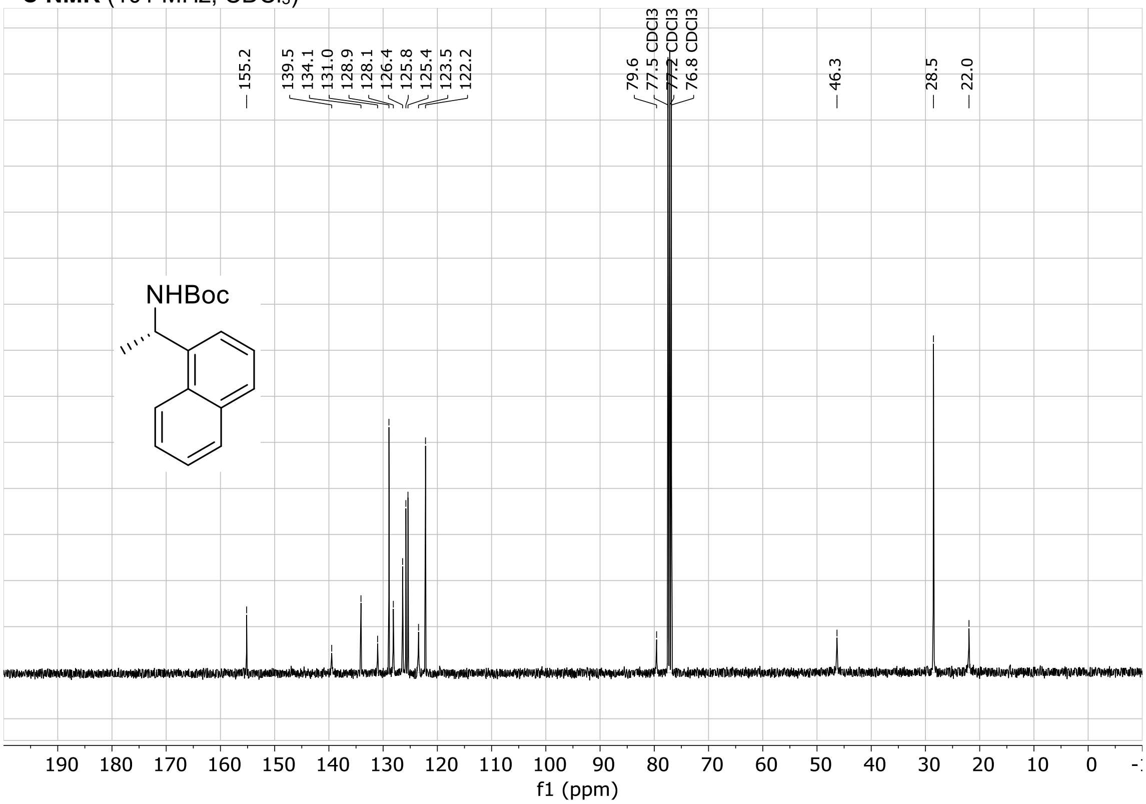

Benzyl (*S*)-(1-(naphthalen-1-yl)ethyl)carbamate **15**

^{1}H NMR (400 MHz, $CDCl_3$)

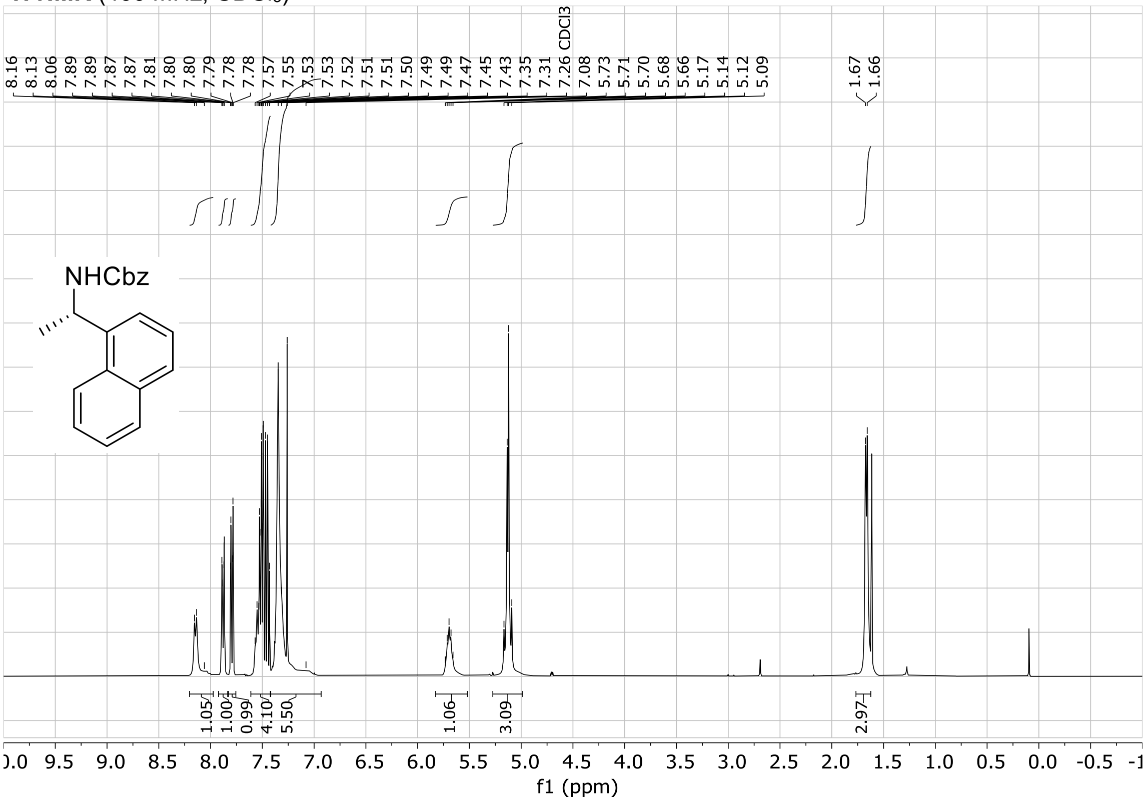

^{13}C NMR (101 MHz, $CDCl_3$)

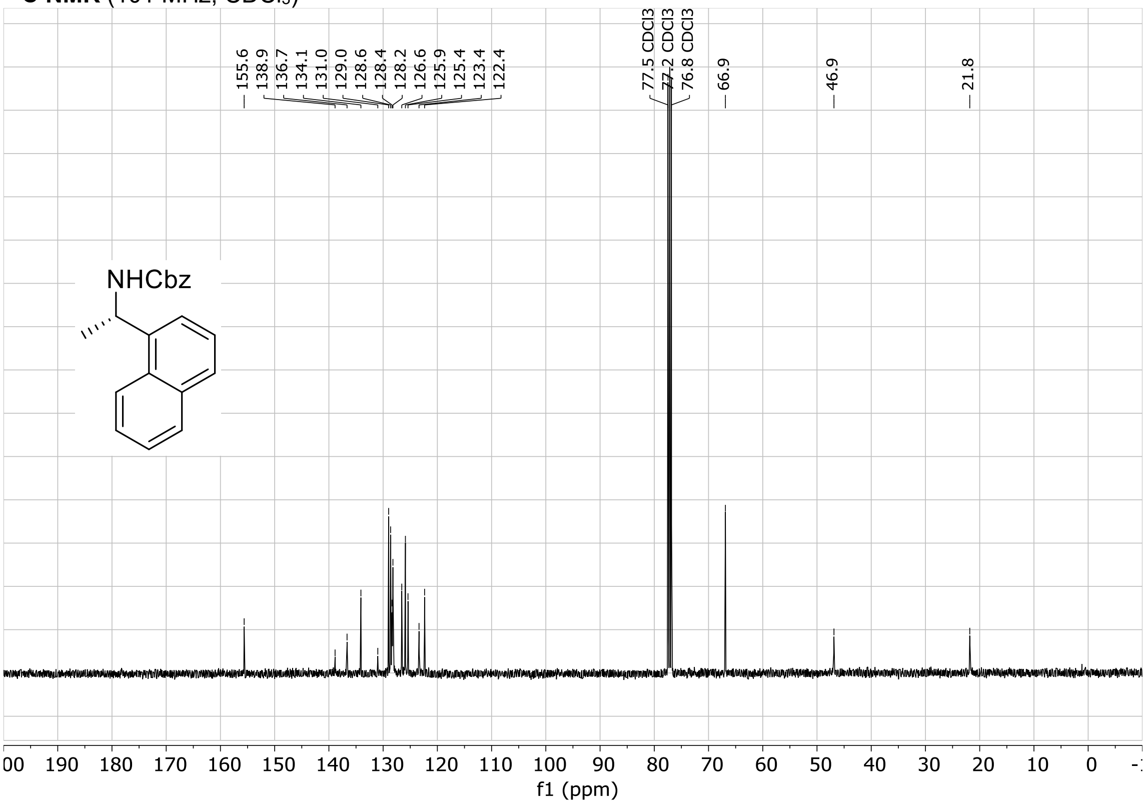

2.2 X-ray crystallography data

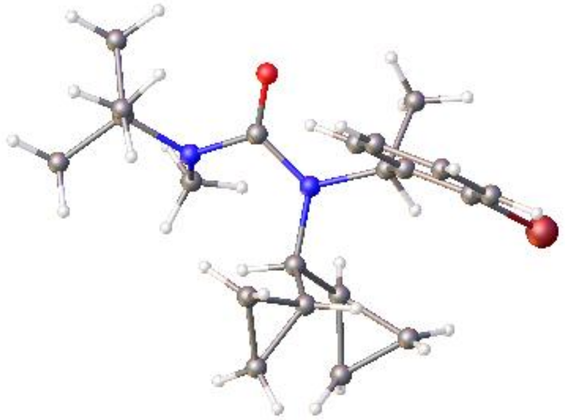

Colourless crystals of **9b** were grown from a $CHCl_3$ solution by slow vapour diffusion of petroleum ether and analysed by the technical staff at the University of Bristol on a Bruker D8 Venture single-crystal x-ray diffractometer.

Compound	**9b**
CCDC deposition number	2555138
Empirical formula	$C_{21}H_{31}BrN_2O$
Formula weight	407.39
Temperature/K	100(2)
Crystal system	orthorhombic
Space group	$P2_12_12_1$
a/Å	19.8048(9)
b/Å	11.5362(5)
c/Å	9.0948(4)
α/°	90
β/°	90
γ/°	90
Volume/Å^3	2077.91(16)
Z	4
ρ_{calc}g/cm^3	1.302
μ/mm^{-1}	1.989
F(000)	856.0
Crystal size/mm^3	0.321 × 0.213 × 0.196
Radiation	MoKα (λ = 0.71073)

2Θ range for data collection/°	4.086 to 55.776
Index ranges	-26 ≤ h ≤ 21, -14 ≤ k ≤ 15, -9 ≤ l ≤ 11
Reflections collected	30406
Independent reflections	4945 [R_{int} = 0.0619, R_{sigma} = 0.0507]
Data/restraints/parameters	4945/0/231
Goodness-of-fit on F^2	1.019
Final R indexes [I>=2σ (I)]	R_1 = 0.0328, wR_2 = 0.0574
Final R indexes [all data]	R_1 = 0.0484, wR_2 = 0.0623
Largest diff. peak/hole / e Å^{-3}	0.27/−0.47

2.3 HPLC spectra

1-(*tert*-Butyl)-3-ethyl-1-methyl-3-(1-phenylethyl)urea **2a**

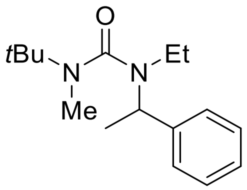

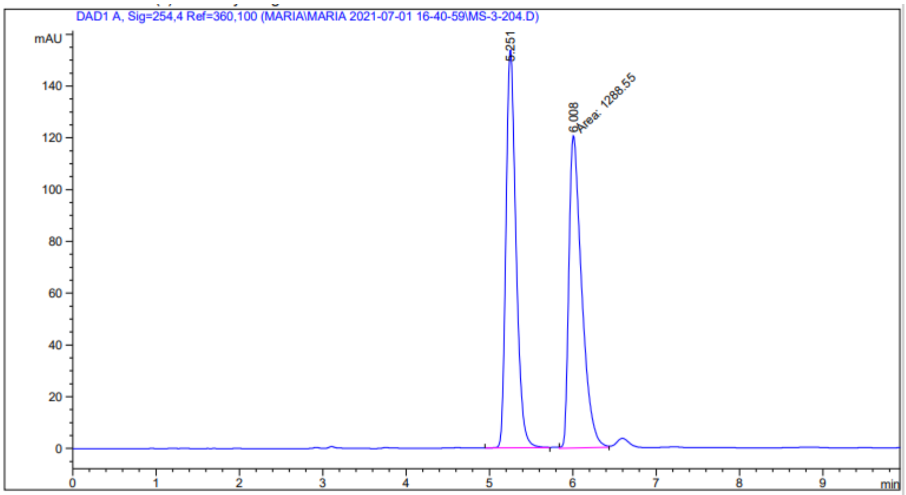

Peak #	RetTime [min]	Type	Width [min]	Area [mAU*s]	Height [mAU]	Area %
1	5.251	BB	0.1273	1281.36389	153.62350	49.8603
2	6.008	MM	0.1780	1288.54626	120.66818	50.1397

(*S*)-1-(*tert*-Butyl)-3-isopropyl-1-methyl-3-(1-phenylethyl)urea **2b = 7a**

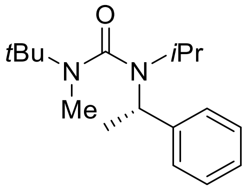

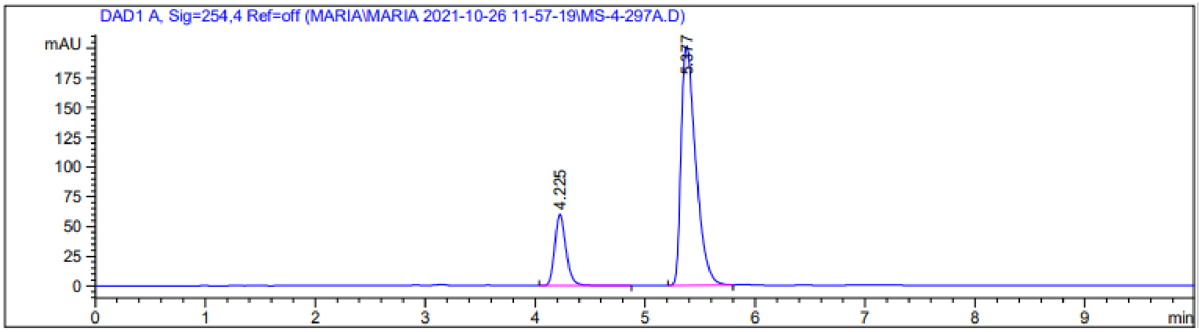

Peak #	RetTime [min]	Type	Width [min]	Area [mAU*s]	Height [mAU]	Area %
1	4.225	BB	0.1092	430.39160	60.35245	18.7617
2	5.377	BB	0.1420	1863.60034	201.38063	81.2383

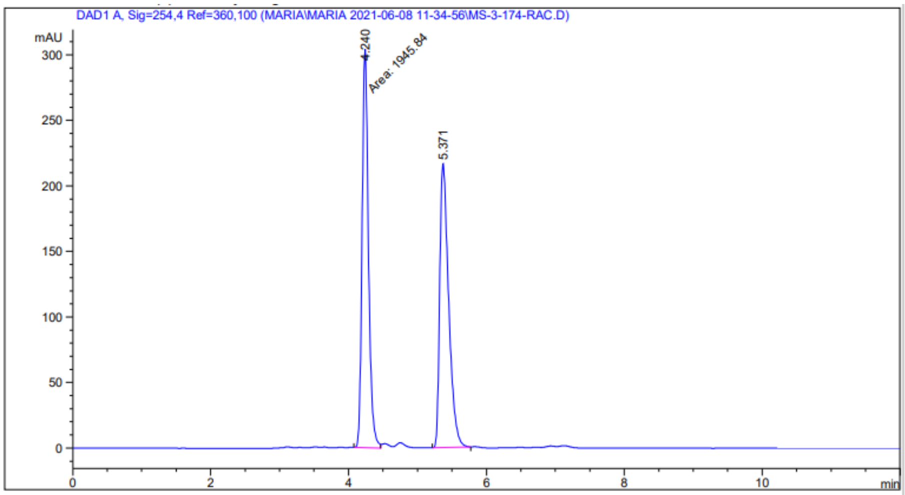

Peak #	RetTime [min]	Type	Width [min]	Area [mAU*s]	Height [mAU]	Area %
1	4.240	MM	0.1066	1945.84338	304.25436	49.9445
2	5.371	BB	0.1350	1950.16882	216.75589	50.0555

(*R*)-1-(*tert*-Butyl)-3-isopropyl-1-methyl-3-(1-phenylethyl)urea (*R*)-**7a**

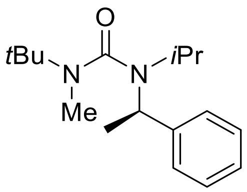

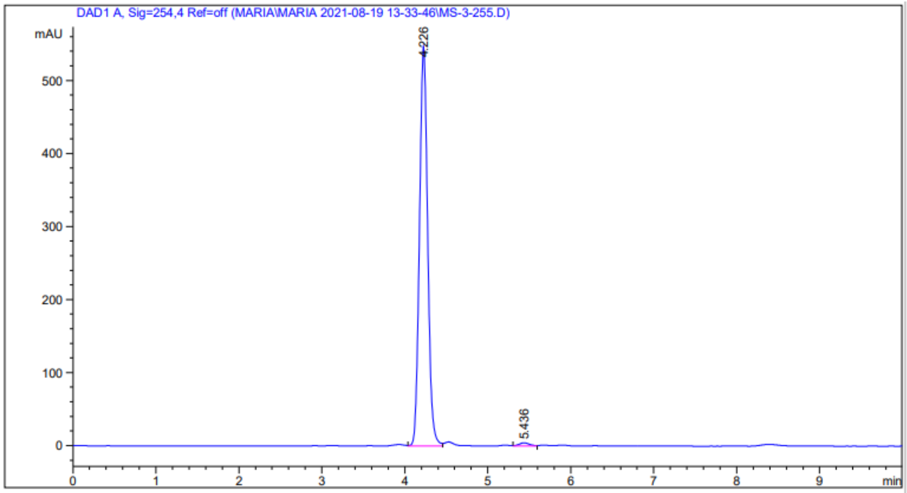

Peak #	RetTime [min]	Type	Width [min]	Area [mAU*s]	Height [mAU]	Area %
1	4.226	VV	0.1081	3753.23486	546.92841	98.9892
2	5.436	VV	0.1357	38.32481	4.31073	1.0108

(*S*)-1-(*tert*-Butyl)-1-ethyl-3-isopropyl -3-(1-phenylethyl)urea **SI-2**

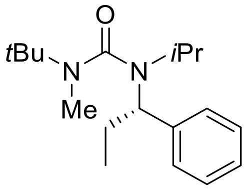

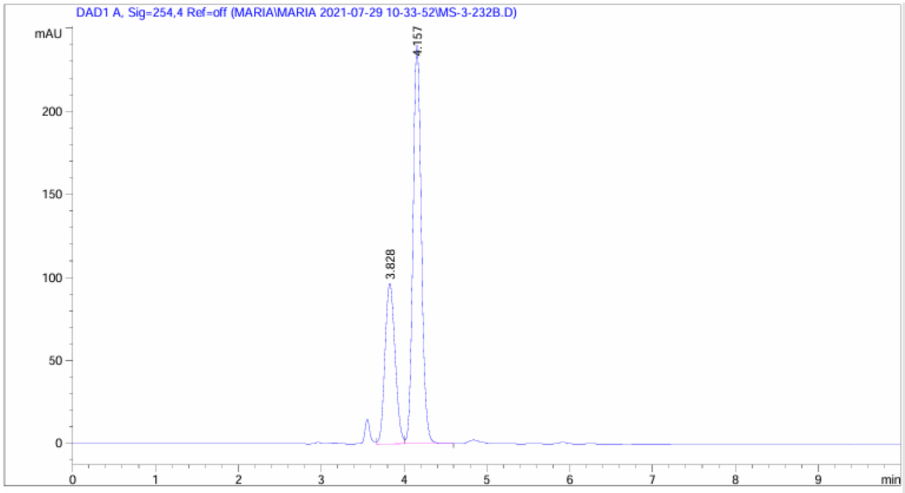

Peak #	RetTime [min]	Type	Width [min]	Area [mAU*s]	Height [mAU]	Area %
1	3.828	VV	0.1325	815.95038	96.69205	32.6550
2	4.157	VB	0.1097	1682.74976	240.16750	67.3450

(*S*)-1-Ethyl-1,3-diisopropyl-3-(1-phenylethyl)urea **2d**

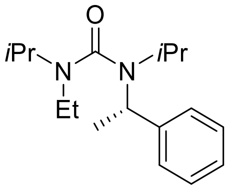

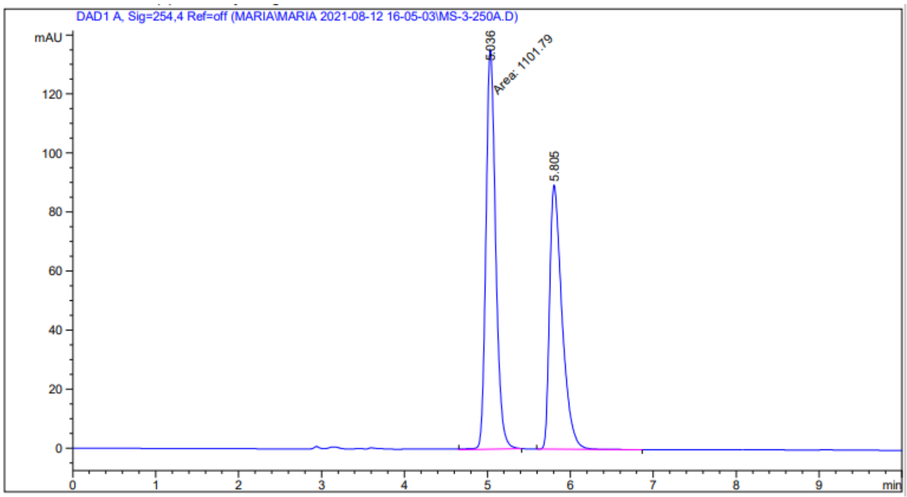

Peak #	RetTime [min]	Type	Width [min]	Area [mAU*s]	Height [mAU]	Area %
1	5.036	MM	0.1357	1101.78577	135.37051	54.1321
2	5.805	BB	0.1556	933.57947	89.59070	45.8679

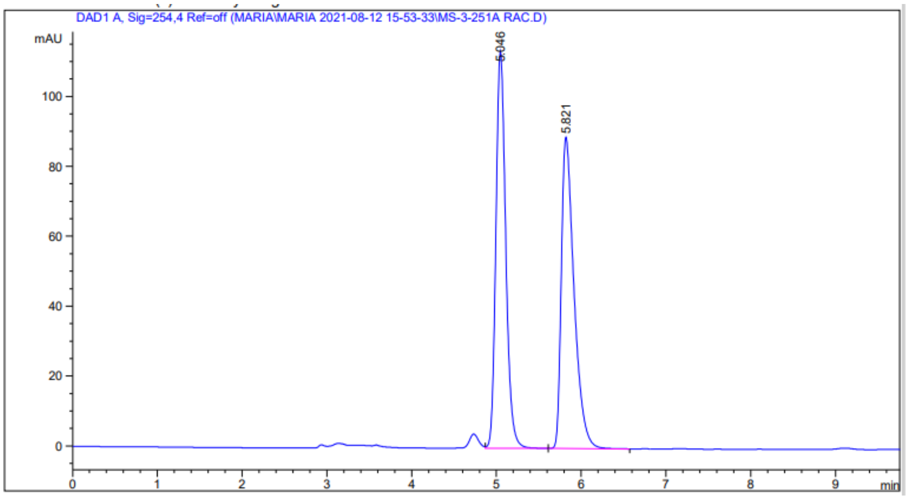

Peak #	RetTime [min]	Type	Width [min]	Area [mAU*s]	Height [mAU]	Area %
1	5.046	VB	0.1248	922.55896	113.55415	49.9298
2	5.821	BB	0.1572	925.15381	89.07916	50.0702

(*S*)-1-(*tert*-Butyl)-1-ethyl-3-isopropyl-3-(1-phenylethyl)urea **3e**

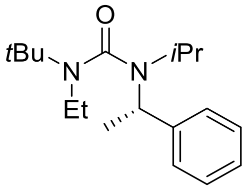

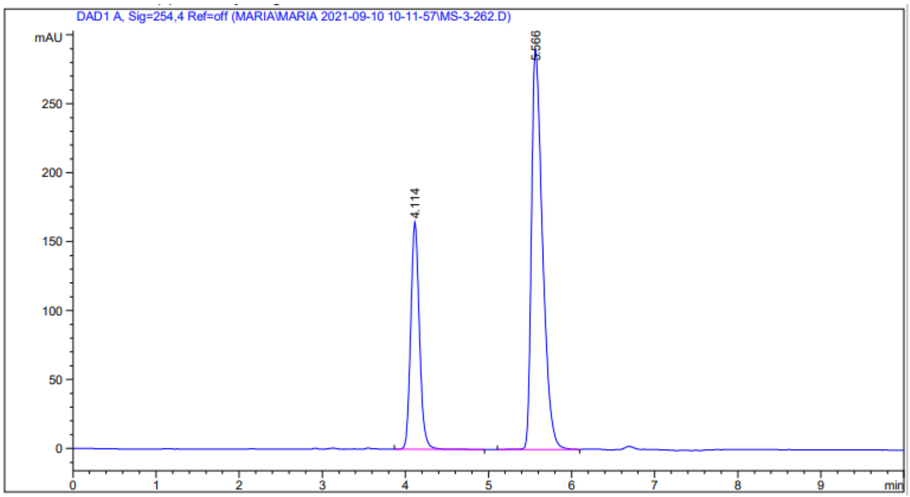

Peak #	RetTime [min]	Type	Width [min]	Area [mAU*s]	Height [mAU]	Area %
1	4.114	BB	0.1107	1195.62927	164.67836	30.7576
2	5.566	BB	0.1405	2691.63672	289.49286	69.2424

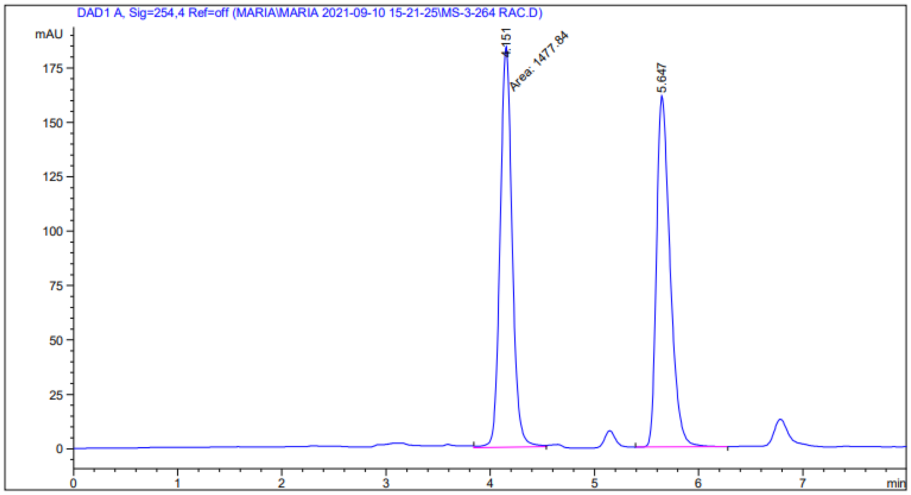

Peak #	RetTime [min]	Type	Width [min]	Area [mAU*s]	Height [mAU]	Area %
1	4.151	MM	0.1337	1477.84375	184.23967	50.2817
2	5.647	BB	0.1378	1461.28198	161.19658	49.7183

(*S*)-1-(*tert*-Butyl)-3-cyclohexyl-1-methyl-3-(1-phenylethyl)urea **7b**

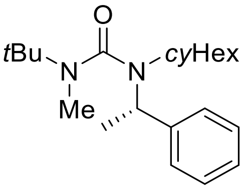

3-242B

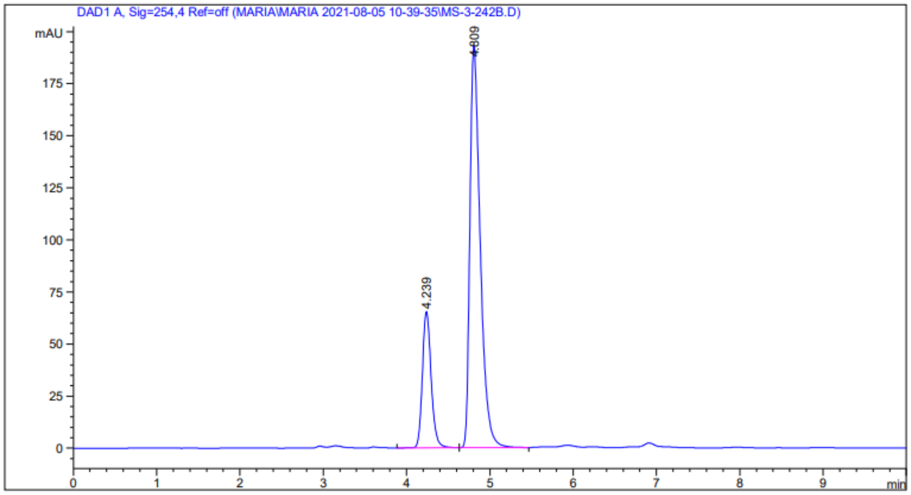

Peak #	RetTime [min]	Type	Width [min]	Area [mAU*s]	Height [mAU]	Area %
1	4.239	BB	0.1134	478.90836	65.41039	22.3813
2	4.809	BB	0.1325	1660.85913	192.81781	77.6187

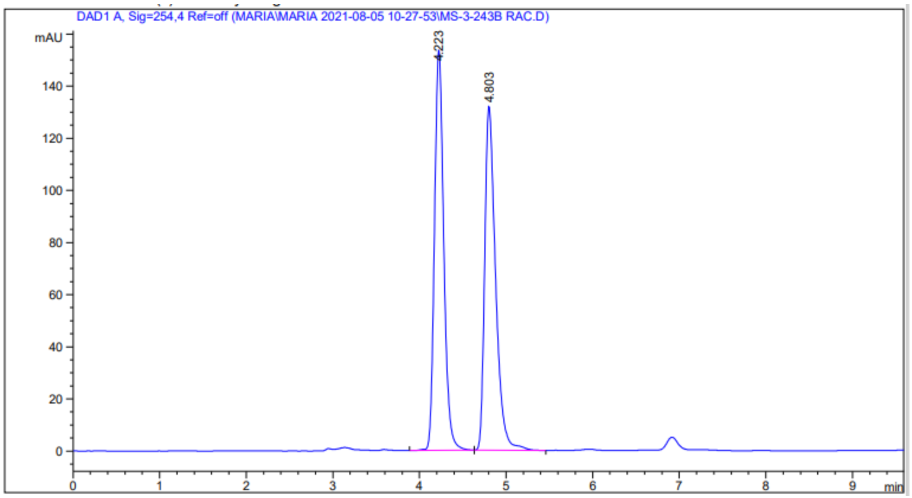

Peak #	RetTime [min]	Type	Width [min]	Area [mAU*s]	Height [mAU]	Area %
1	4.223	BB	0.1150	1147.16724	153.75652	49.8759
2	4.803	BB	0.1339	1152.87537	131.98633	50.1241

(*S*)-1-(*tert*-Butyl)-3-isopropyl-1-methyl-3-(1-(*o*-tolyl)ethyl)urea **7c**

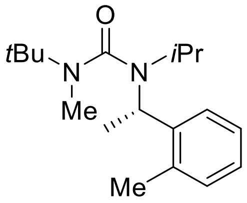

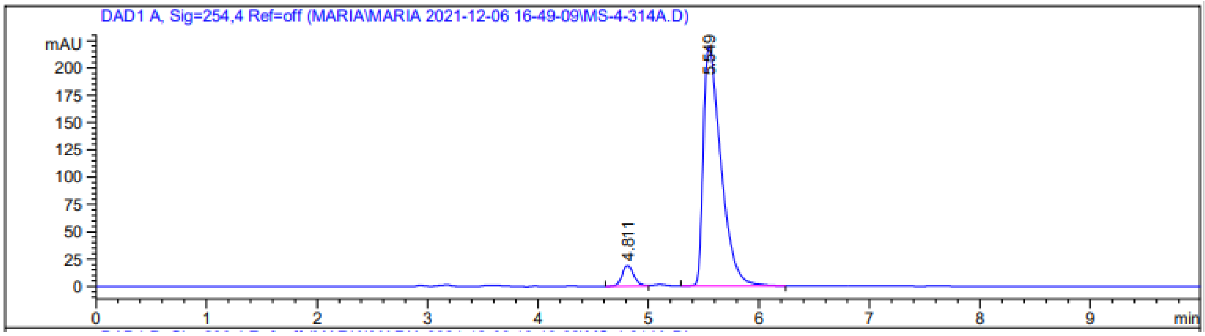

Peak #	RetTime [min]	Type	Width [min]	Area [mAU*s]	Height [mAU]	Area %
1	4.811	BV	0.1145	140.35687	18.93246	5.4569
2	5.549	BB	0.1673	2431.73804	219.41019	94.5431

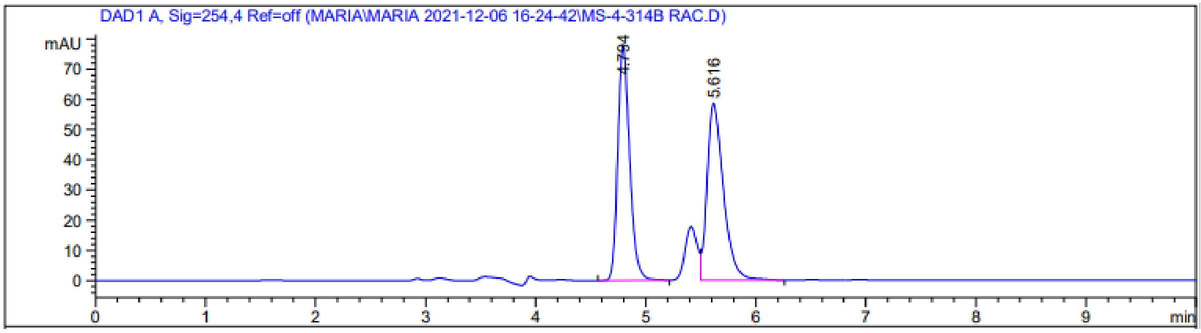

Peak #	RetTime [min]	Type	Width [min]	Area [mAU*s]	Height [mAU]	Area %
1	4.794	BV	0.1159	584.29199	77.54034	49.1182
2	5.616	VB	0.1566	605.27173	58.55116	50.8818

(*S*)-1-(*tert*-Butyl)-3-(1-(4-fluoro-2-methylphenyl)ethyl)-3-isopropyl-1-methylurea **7d**

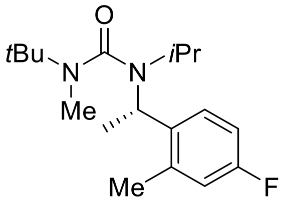

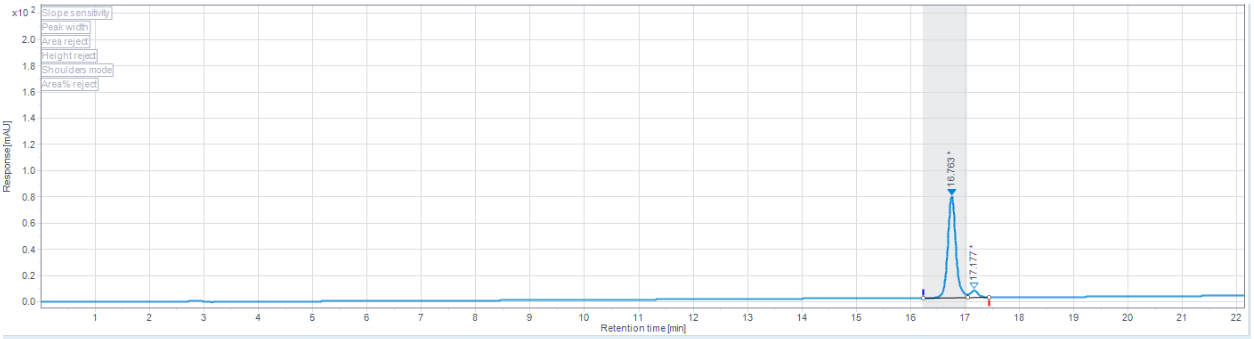

Injection Results

Peaks Summary

#	Name	Signal description	RT (min)	Area (mAU·s)	Area%	Height (mAU)	Height%	Amount	Concentration	Start time (min)	End time (min)
1		DAD1A,Sig=254,4 Ref=off	16.763	796.924	93.639	77.451	93.60			16.231	17.051
2		DAD1A,Sig=254,4 Ref=off	17.177	54.140	6.361	5.298	6.40			17.051	17.456

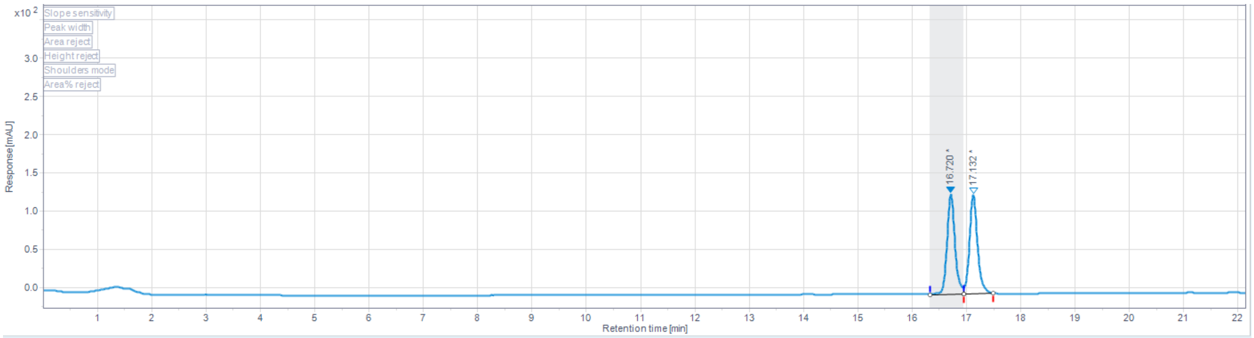

Injection Results

Peaks Summary

#	Name	Signal description	RT (min)	Area (mAU·s)	Area%	Height (mAU)	Height%	Amount	Concentration	Start time (min)	End time (min)
1		DAD1A,Sig=254,4 Ref=off	16.720	1341.311	50.029	130.382	50.20			16.332	16.952
2		DAD1A,Sig=254,4 Ref=off	17.132	1339.736	49.971	129.361	49.80			16.952	17.495

(*S*)-1-(*tert*-Butyl)-3-isopropyl-1-methyl-3-(1-(*p*-tolyl)ethyl)urea **7e**

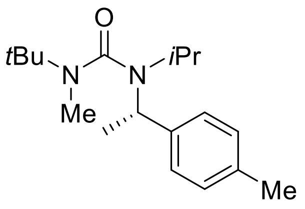

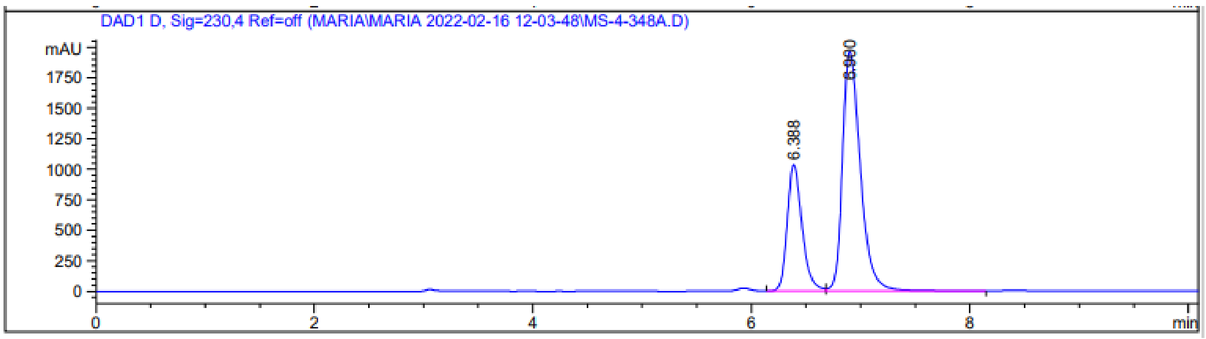

Peak #	RetTime [min]	Type	Width [min]	Area [mAU*s]	Height [mAU]	Area %
1	6.388	BV	0.1441	9779.53516	1036.65771	30.4914
2	6.900	VB	0.1745	2.22935e4	1962.60718	69.5086

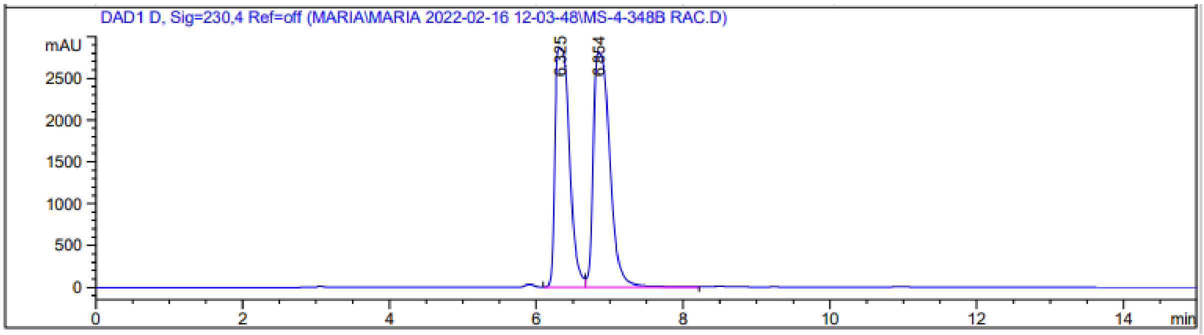

Peak #	RetTime [min]	Type	Width [min]	Area [mAU*s]	Height [mAU]	Area %
1	6.325	BV	0.2181	3.92391e4	2859.38989	47.2119
2	6.854	VV	0.2485	4.38735e4	2804.71045	52.7881

(*S*)-1-(*tert*-Butyl)-3-isopropyl-1-methyl-3-(1-(*m*-tolyl)ethyl)urea **7f**

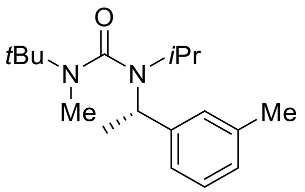

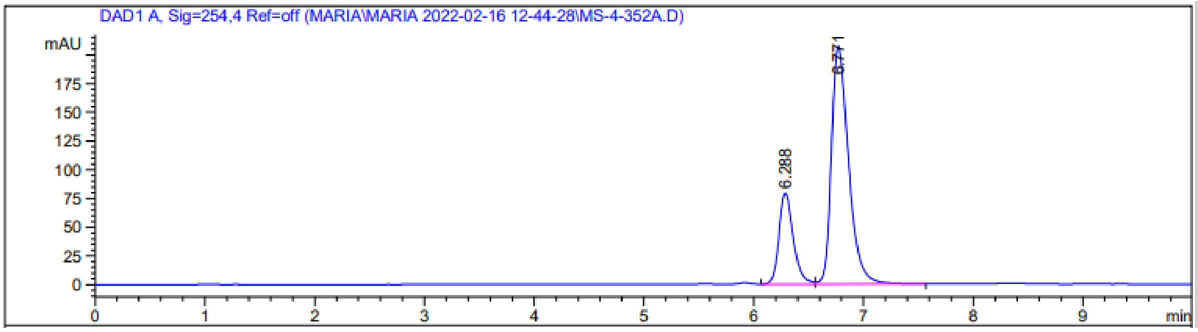

Peak #	RetTime [min]	Type	Width [min]	Area [mAU*s]	Height [mAU]	Area %
1	6.288	VV	0.1375	716.93835	79.29150	24.2717
2	6.771	VB	0.1640	2236.86694	207.14865	75.7283

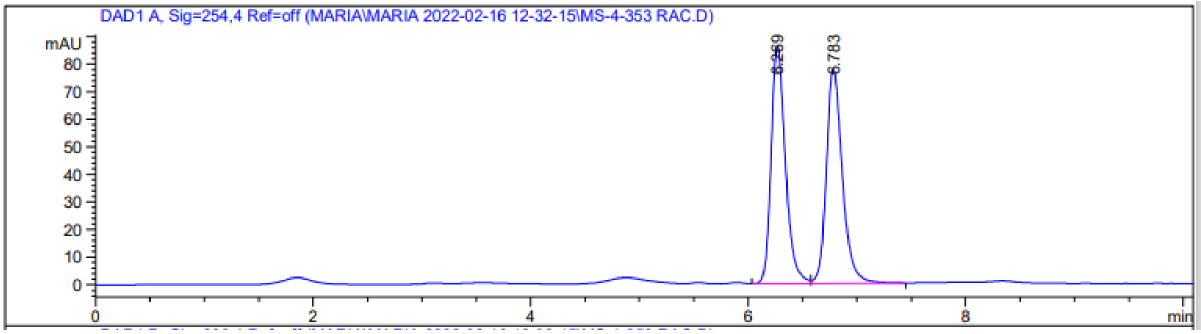

Peak #	RetTime [min]	Type	Width [min]	Area [mAU*s]	Height [mAU]	Area %
1	6.269	BV	0.1384	784.35040	86.02705	49.6357
2	6.783	VB	0.1557	795.86517	77.59390	50.3643

(*S*)-1-(*tert*-Butyl)-3-(1-(2-chlorophenyl)ethyl)-3-isopropyl-1-methylurea **7g**

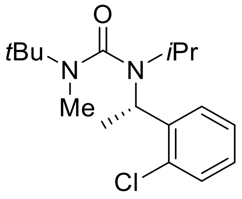

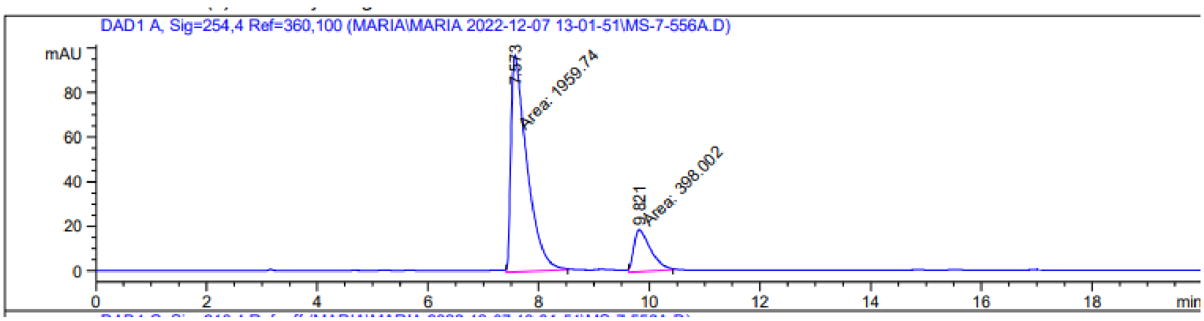

Peak #	RetTime [min]	Type	Width [min]	Area [mAU*s]	Height [mAU]	Area %
1	7.573	MM	0.3354	1959.73962	97.38288	83.1193
2	9.821	MM	0.3539	398.00235	18.74237	16.8807

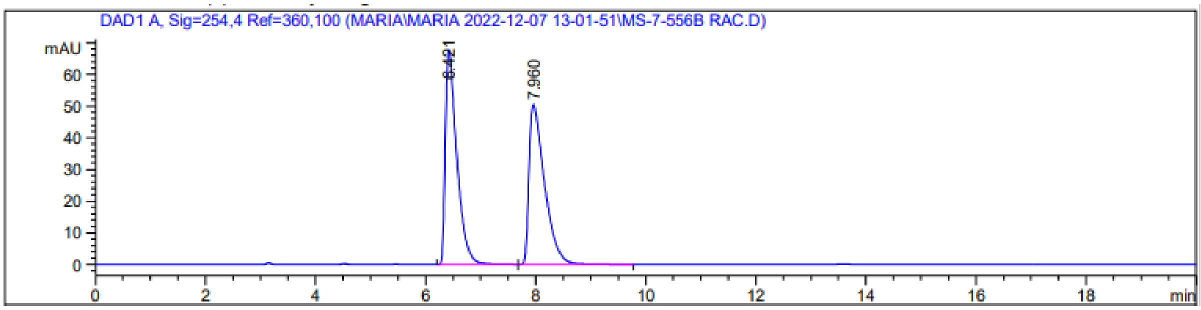

Peak #	RetTime [min]	Type	Width [min]	Area [mAU*s]	Height [mAU]	Area %
1	6.421	BB	0.2161	988.35632	67.80479	49.9355
2	7.960	BB	0.2933	990.90979	50.42249	50.0645

(*S*)-1-(1-(2-Bromophenyl)ethyl)-3-(*tert*-butyl)-1-isopropyl-3-methylurea **7h**

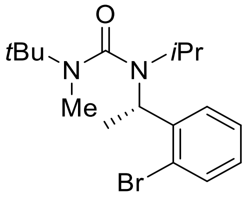

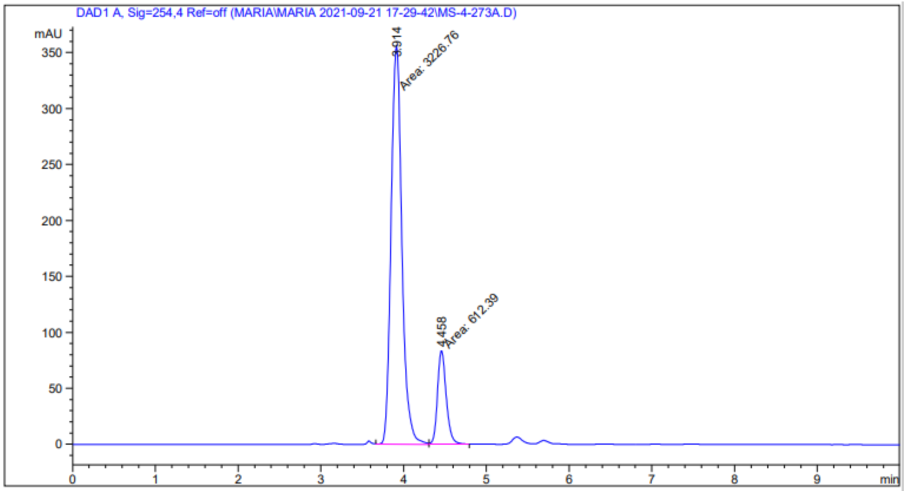

Peak #	RetTime [min]	Type	Width [min]	Area [mAU*s]	Height [mAU]	Area %
1	3.914	MM	0.1512	3226.75928	355.61319	84.0488
2	4.458	MM	0.1221	612.38977	83.58614	15.9512

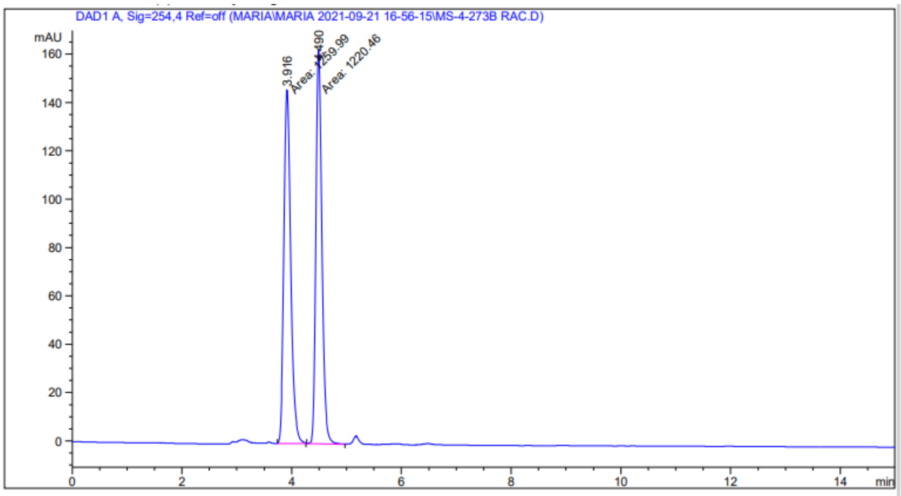

Peak #	RetTime [min]	Type	Width [min]	Area [mAU*s]	Height [mAU]	Area %
1	3.916	MM	0.1434	1259.99109	146.44498	50.7968
2	4.490	MM	0.1245	1220.46265	163.38548	49.2032

(*S*)-1-(1-(3-Bromophenyl)ethyl)-3-(*tert*-butyl)-1-isopropyl-3-methylurea **7i**

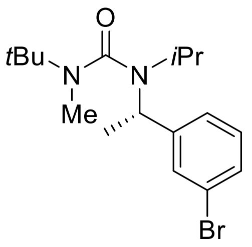

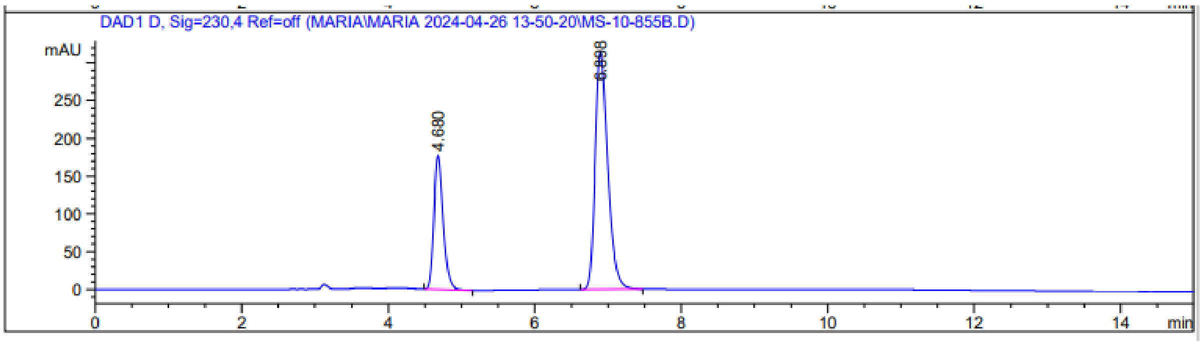

Peak #	RetTime [min]	Type	Width [min]	Area [mAU*s]	Height [mAU]	Area %
1	4.680	BB	0.1374	1574.02563	177.58388	29.6092
2	6.898	BB	0.1811	3741.98315	313.78000	70.3908

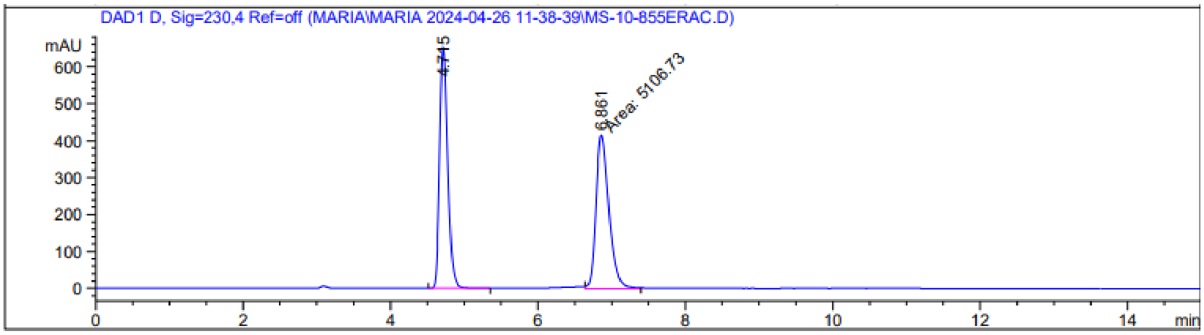

Peak #	RetTime [min]	Type	Width [min]	Area [mAU*s]	Height [mAU]	Area %
1	4.715	BB	0.1159	4903.28076	651.12115	48.9838
2	6.861	MM	0.2043	5106.72656	416.66885	51.0162

(*S*)-1-(*tert*-Butyl)-3-isopropyl-3-(1-(2-methoxyphenyl)ethyl)-1-methylurea **7j**

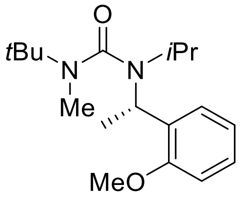

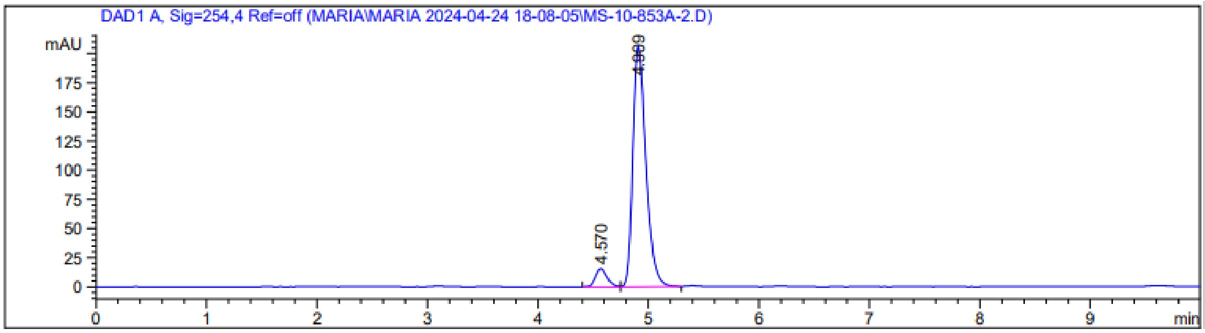

Peak #	RetTime [min]	Type	Width [min]	Area [mAU*s]	Height [mAU]	Area %
1	4.570	BV	0.1133	111.73793	15.28374	6.1882
2	4.909	VV	0.1259	1693.91577	206.09355	93.8118

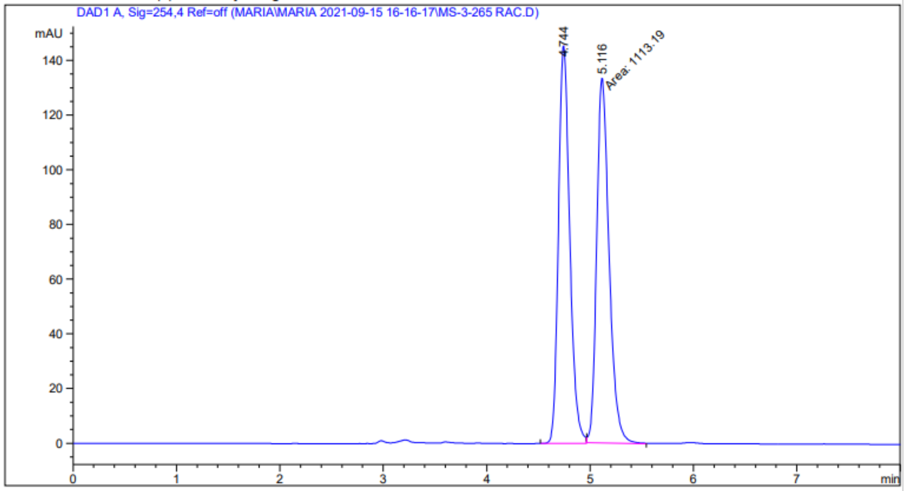

Peak #	RetTime [min]	Type	Width [min]	Area [mAU*s]	Height [mAU]	Area %
1	4.744	BV	0.1165	1104.64185	145.59314	49.8073
2	5.116	MM	0.1389	1113.19031	133.59885	50.1927

(*S*)-1-(*tert*-Butyl)-3-isopropyl-1-methyl-3-(1-(4-methoxynaphthalen-1-yl)ethyl)urea **7k**

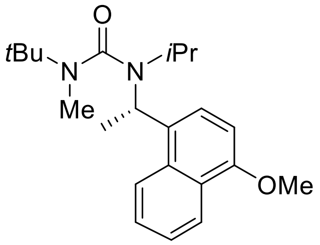

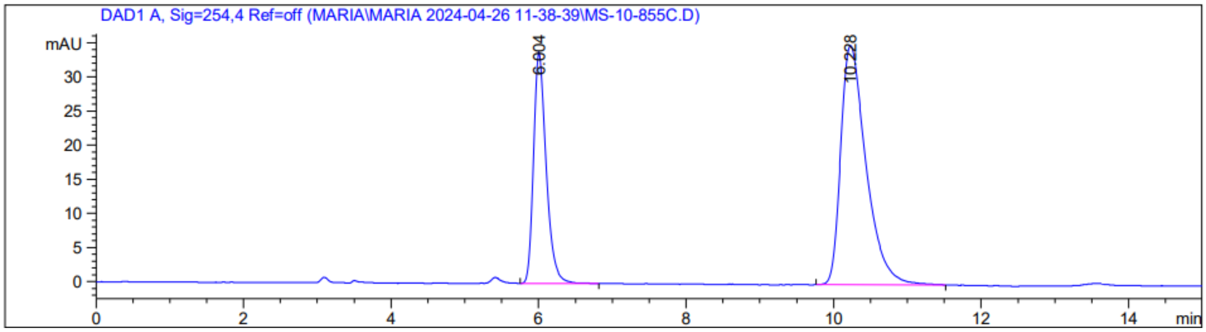

Peak #	RetTime [min]	Type	Width [min]	Area [mAU*s]	Height [mAU]	Area %
1	6.004	BB	0.1839	407.01004	33.93154	32.7390
2	10.228	BB	0.3643	836.18726	35.04921	67.2610

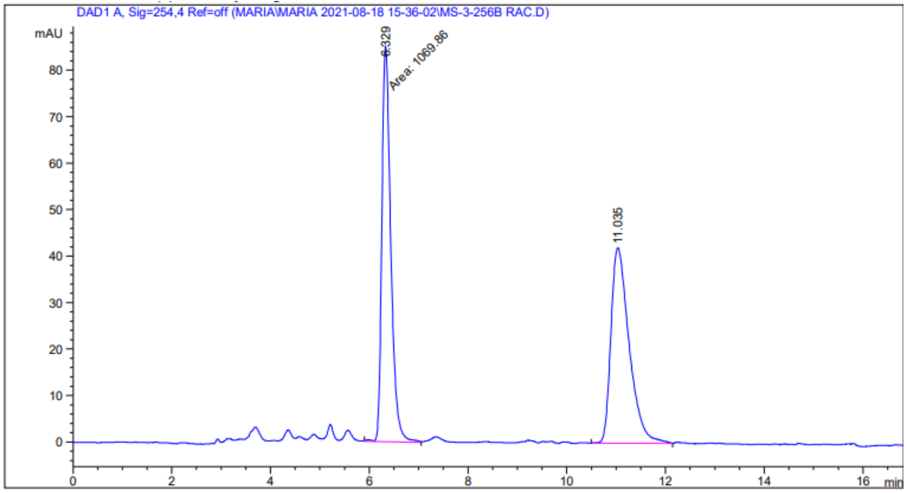

Peak #	RetTime [min]	Type	Width [min]	Area [mAU*s]	Height [mAU]	Area %
1	6.329	MM	0.2093	1069.85962	85.17505	49.9427
2	11.035	BB	0.3857	1072.31555	42.02739	50.0573

(*S*)-1-(*tert*-Butyl)-3-isopropyl-1-methyl-3-(1-(4-methylnaphthalen-1-yl)ethyl)urea **7l**

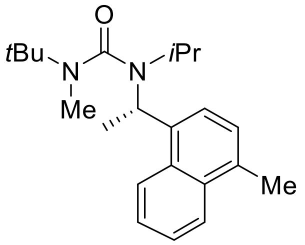

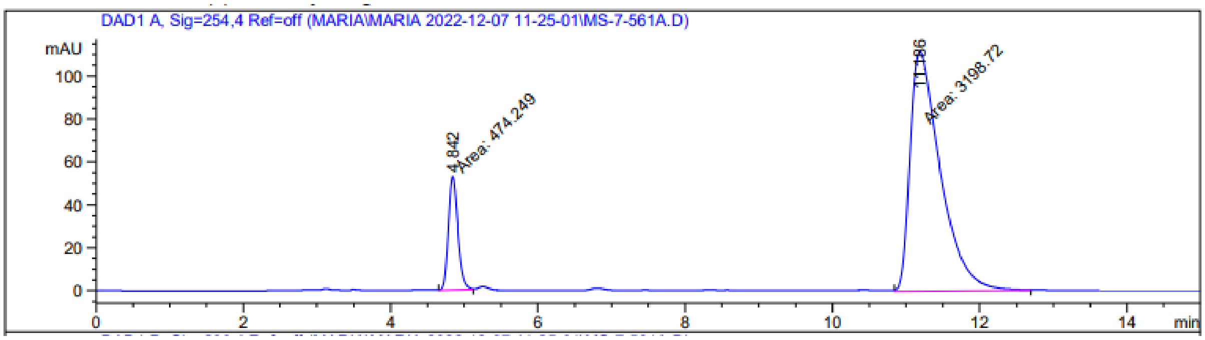

Peak #	RetTime [min]	Type	Width [min]	Area [mAU*s]	Height [mAU]	Area %
1	4.842	MM	0.1497	474.24899	52.79868	12.9119
2	11.186	MM	0.4766	3198.71680	111.84966	87.0881

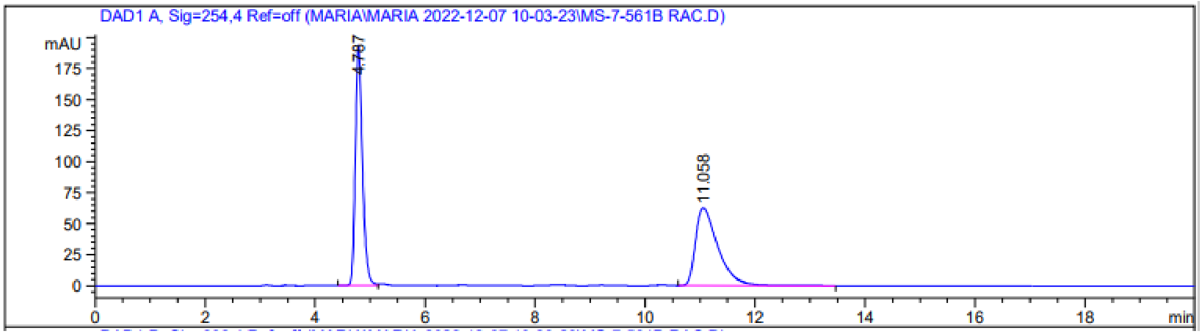

Peak #	RetTime [min]	Type	Width [min]	Area [mAU*s]	Height [mAU]	Area %
1	4.787	BV	0.1404	1758.30811	192.77060	49.6895
2	11.058	BB	0.4258	1780.28333	62.59901	50.3105

(*S*)-1-(*tert*-Butyl)-3-(dicyclopropylmethyl)-1-methyl-3-(1-phenylethyl)urea **9a**

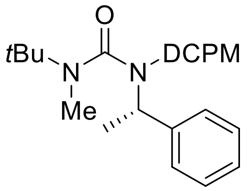

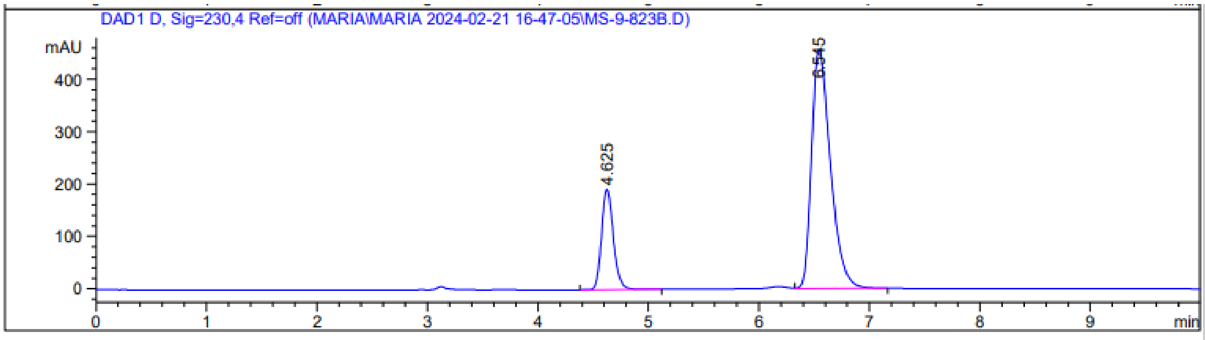

Peak #	RetTime [min]	Type	Width [min]	Area [mAU*s]	Height [mAU]	Area %
1	4.625	BB	0.1143	1418.95044	191.85509	20.7618
2	6.545	VB	0.1805	5415.48096	456.27362	79.2382

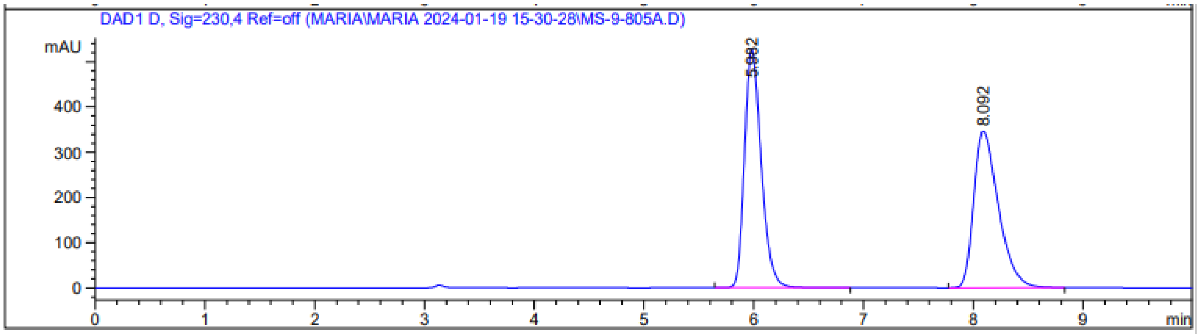

Peak #	RetTime [min]	Type	Width [min]	Area [mAU*s]	Height [mAU]	Area %
1	5.982	BB	0.1632	5566.47900	527.28796	50.6810
2	8.092	BB	0.2385	5416.88916	346.29816	49.3190

(*S*)-1-(1-(2-Bromophenyl)ethyl)-3-(*tert*-butyl)-1-(dicyclopropylmethyl)-3-methylurea **9b**

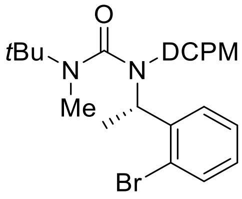

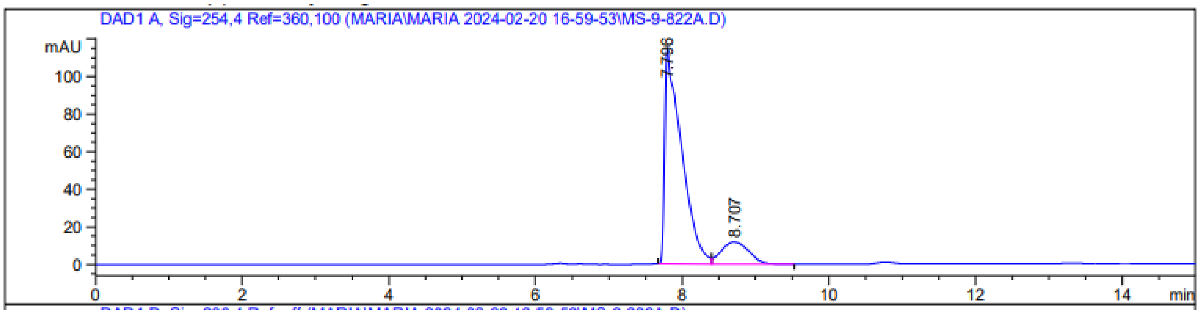

Peak #	RetTime [min]	Type	Width [min]	Area [mAU*s]	Height [mAU]	Area %
1	7.796	BV	0.1994	1824.96545	114.86082	85.6888
2	8.707	VB	0.4141	304.79449	11.69164	14.3112

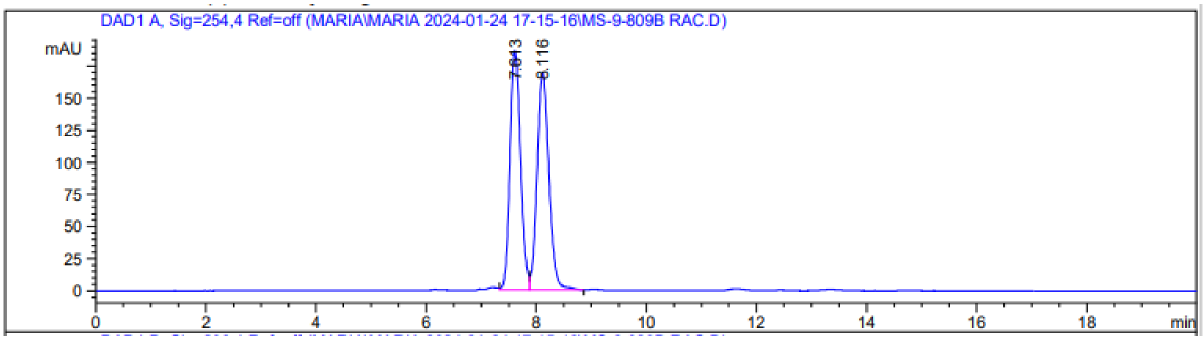

Peak #	RetTime [min]	Type	Width [min]	Area [mAU*s]	Height [mAU]	Area %
1	7.613	VV	0.2067	2463.99585	185.83015	49.4531
2	8.116	VB	0.2318	2518.49634	169.06409	50.5469

(*S*)-1-(*tert*-Butyl)-3-(dicyclopropylmethyl)-1-methyl-3-(1-(o-tolyl)ethyl)urea **9c**

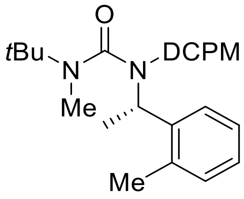

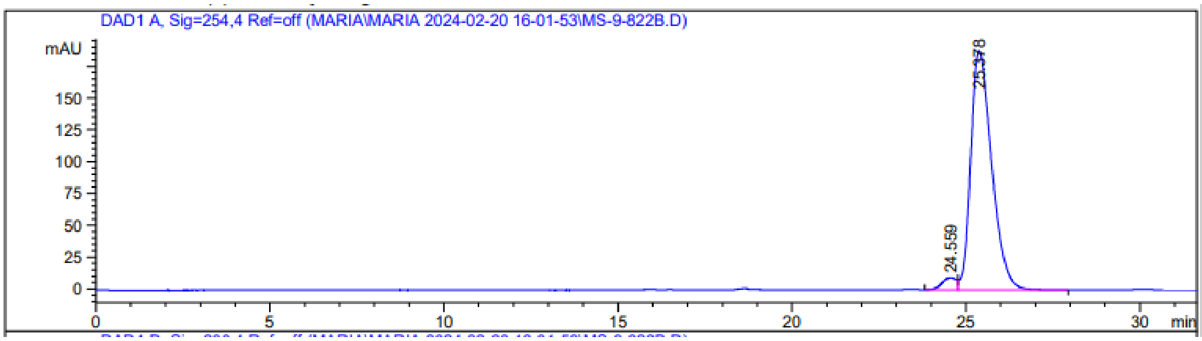

Peak #	RetTime [min]	Type	Width [min]	Area [mAU*s]	Height [mAU]	Area %
1	24.559	BV	0.4100	267.19989	9.44727	3.2855
2	25.378	VB	0.6069	7865.56006	187.71506	96.7145

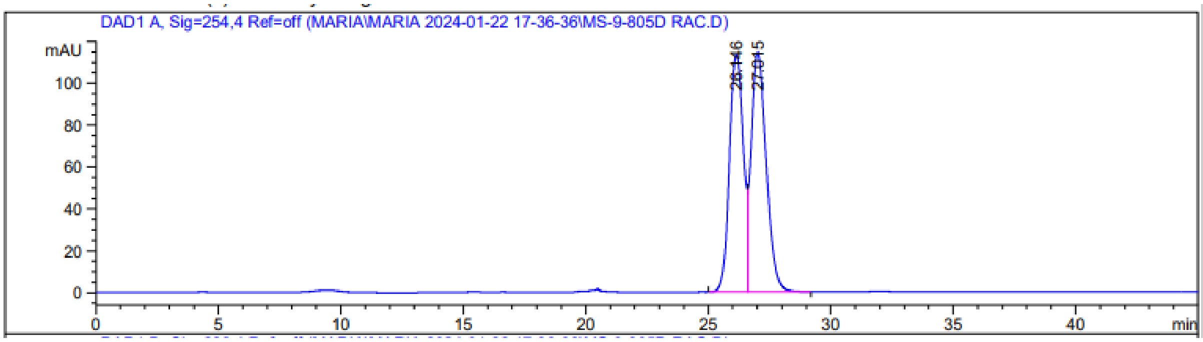

Peak #	RetTime [min]	Type	Width [min]	Area [mAU*s]	Height [mAU]	Area %
1	26.146	BV	0.6289	4443.20215	113.45805	47.4806
2	27.015	VB	0.6515	4914.73340	114.33488	52.5194

(*R*)-1-(*tert*-Butyl)-3-(dicyclopropylmethyl)-3-((1-hydroxycyclobutyl)(phenyl)methyl)-1-methylurea

9d

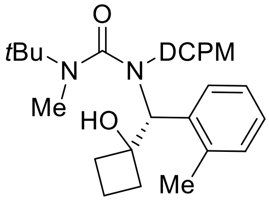

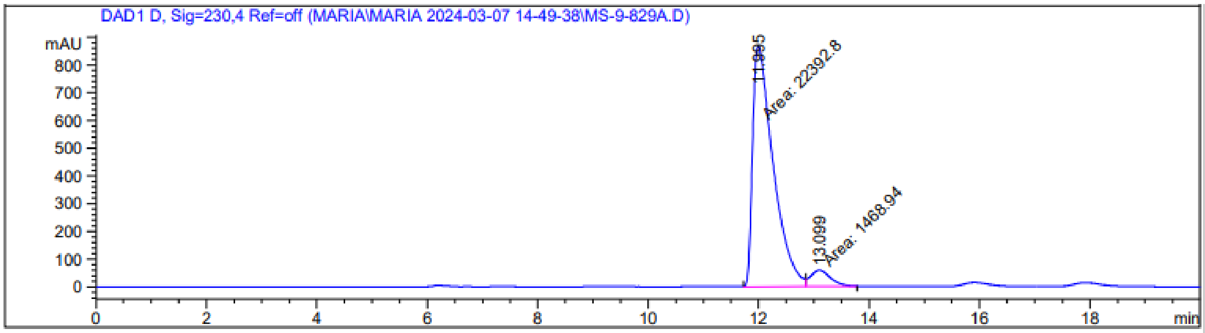

Peak #	RetTime [min]	Type	Width [min]	Area [mAU*s]	Height [mAU]	Area %
1	11.995	MM	0.4309	2.23928e4	866.11176	93.8440
2	13.099	MM	0.4219	1468.94067	58.03061	6.1560

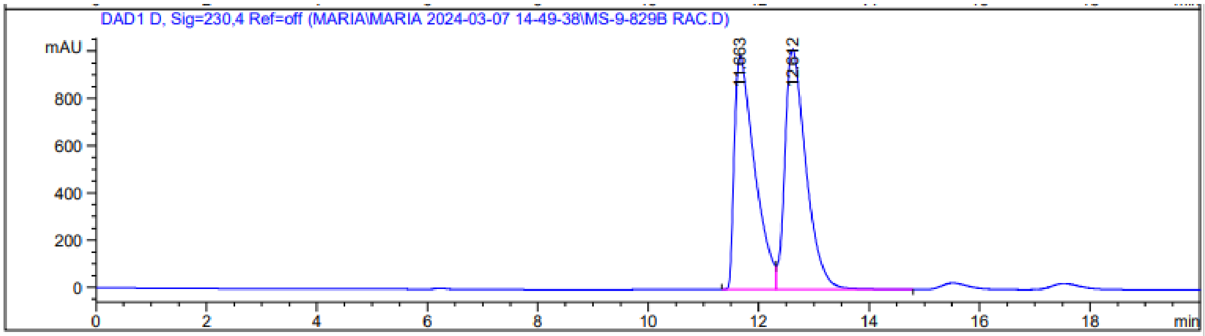

Peak #	RetTime [min]	Type	Width [min]	Area [mAU*s]	Height [mAU]	Area %
1	11.663	BV	0.3781	2.52914e4	989.74817	48.4059
2	12.612	VB	0.4018	2.69572e4	1015.17542	51.5941

(*S*)-1-(*tert*-Butyl)-3-(dicyclopropylmethyl)-1-methyl-3-(1-(naphthalen-1-yl)ethyl)urea **9e**

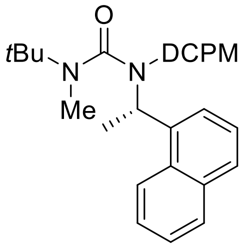

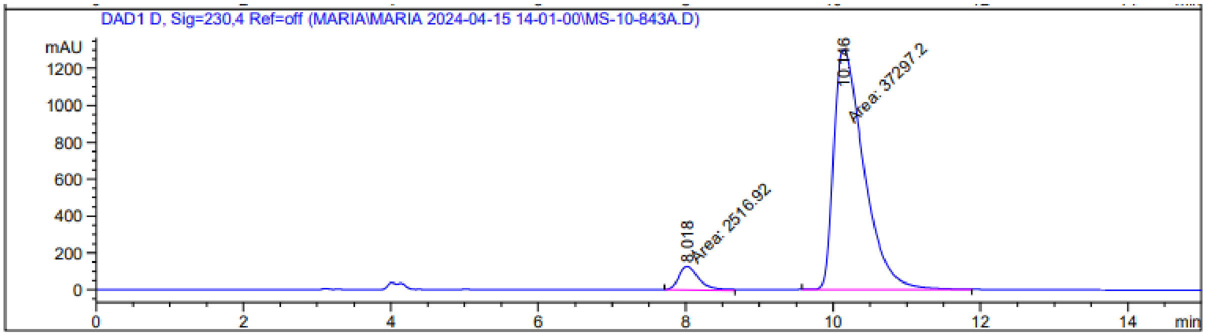

Peak #	RetTime [min]	Type	Width [min]	Area [mAU*s]	Height [mAU]	Area %
1	8.018	MM	0.3268	2516.91943	128.37782	6.3217
2	10.146	MM	0.4774	3.72972e4	1302.12207	93.6783

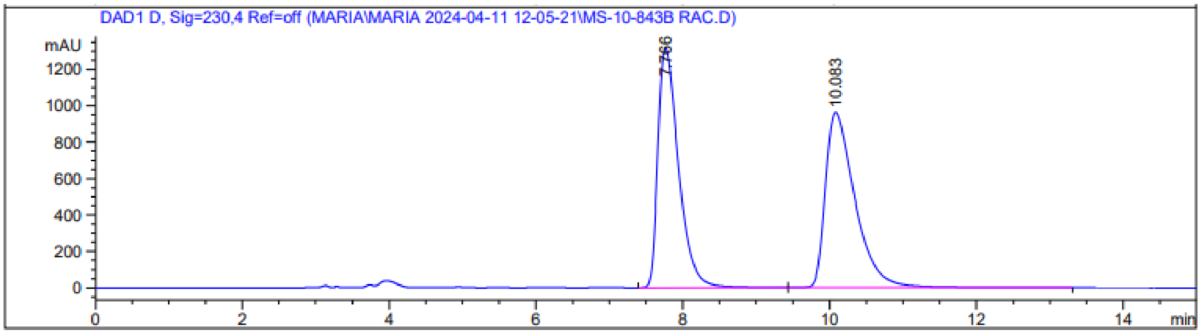

Peak #	RetTime [min]	Type	Width [min]	Area [mAU*s]	Height [mAU]	Area %
1	7.766	BB	0.2963	2.55495e4	1316.93347	48.6847
2	10.083	BB	0.4219	2.69300e4	964.08679	51.3153

(*S*)-1-(*tert*-Butyl)-3-(dicyclopropylmethyl)-1-methyl-3-(phenyl(trimethylsilyl)methyl)urea **11**

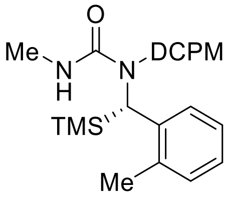

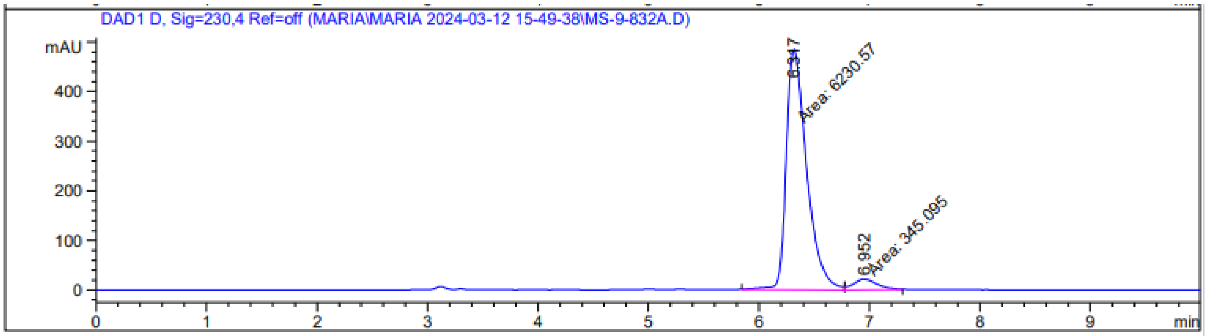

Peak #	RetTime [min]	Type	Width [min]	Area [mAU*s]	Height [mAU]	Area %
1	6.317	MM	0.2141	6230.56543	485.03497	94.7519
2	6.952	MM	0.2582	345.09546	22.27428	5.2481

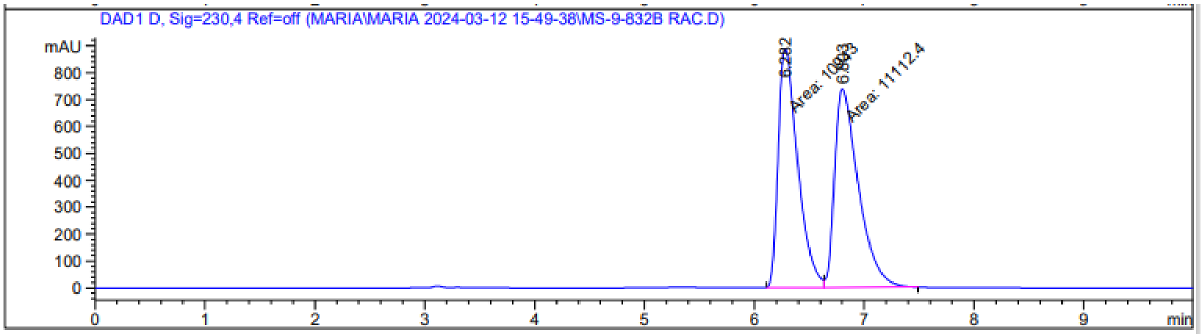

Peak #	RetTime [min]	Type	Width [min]	Area [mAU*s]	Height [mAU]	Area %
1	6.282	MM	0.2059	1.09430e4	885.69489	49.6159
2	6.803	MM	0.2511	1.11124e4	737.47424	50.3841

(*S*)-1-(1-(2-Chlorophenyl)ethyl)-1-isopropyl-3-methylurea **SI-3**

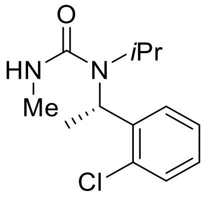

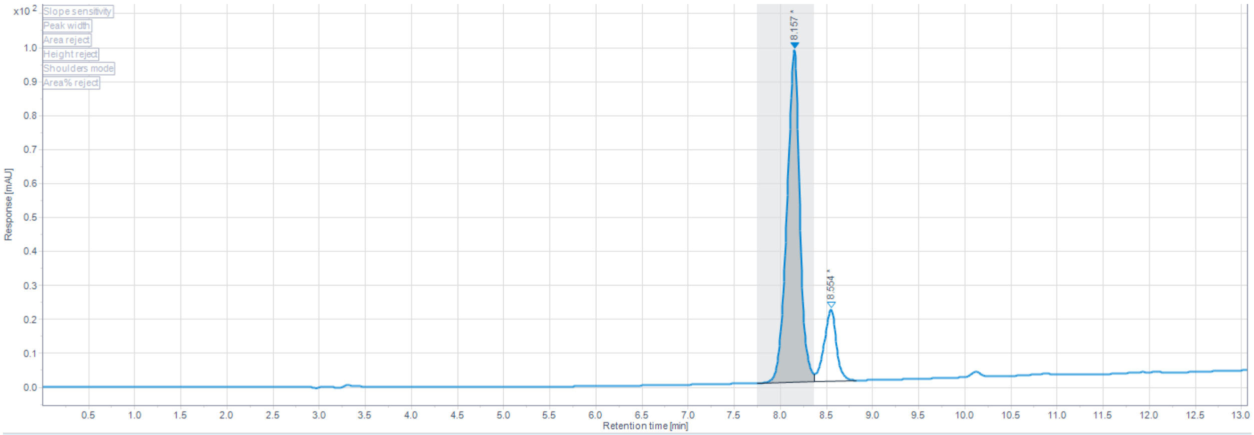

Injection Results

Peaks Summary

#	Name	Signal description	RT (min)	Area (mAU·s)	Area%	Height (mAU)	Height%	Amount	Concentration	Start time (min)	End time (min)
1		DAD1A,Sig=254,4 Ref=off	8.157	954.982	82.976	97.855	82.26			7.751	8.375
2		DAD1A,Sig=254,4 Ref=off	8.554	195.933	17.024	21.103	17.74			8.375	8.825

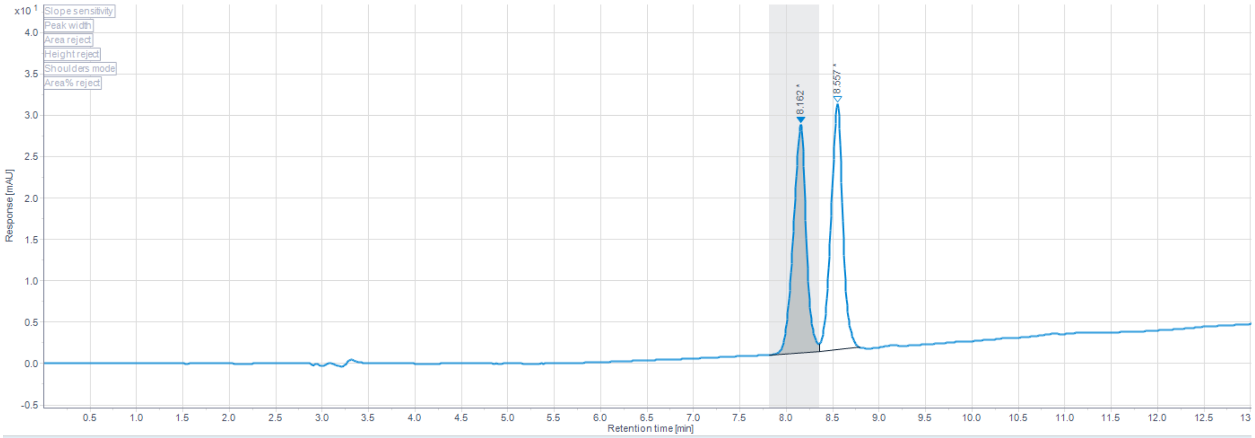

Injection Results

Peaks Summary

#	Name	Signal description	RT (min)	Area (mAU·s)	Area%	Height (mAU)	Height%	Amount	Concentration	Start time (min)	End time (min)
1		DAD1A,Sig=254,4 Ref=off	8.162	266.897	50.014	27.584	48.09			7.827	8.361
2		DAD1A,Sig=254,4 Ref=off	8.557	266.746	49.986	29.779	51.91			8.361	8.797

(*S*)-*N*-(1-(2-Chlorophenyl)ethyl)propan-2-amine **12**

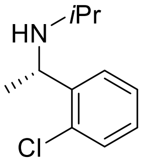

Chiral HPLC analysis of urea derivative (*S*)-1-(1-(2-chlorophenyl)ethyl)-1,3-diisopropylurea

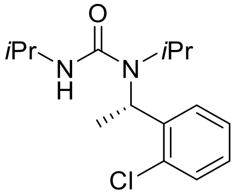

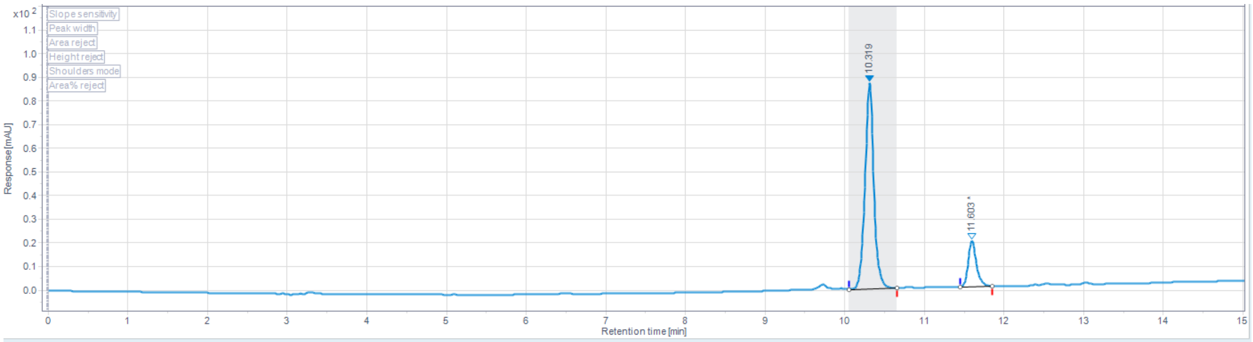

Injection Results

Peaks | Summary

#	Name	Signal description	RT (min)	Area (mAU·s)	Area%	Height (mAU)	Height%	Amount	Concentration	Start time (min)	End time (min)
1		DAD1A,Sig=254,4 Ref=off	10.319	672.594	82.992	87.159	81.72			10.057	10.660
2		DAD1A,Sig=254,4 Ref=off	11.603	137.837	17.008	19.497	18.28			11.451	11.861

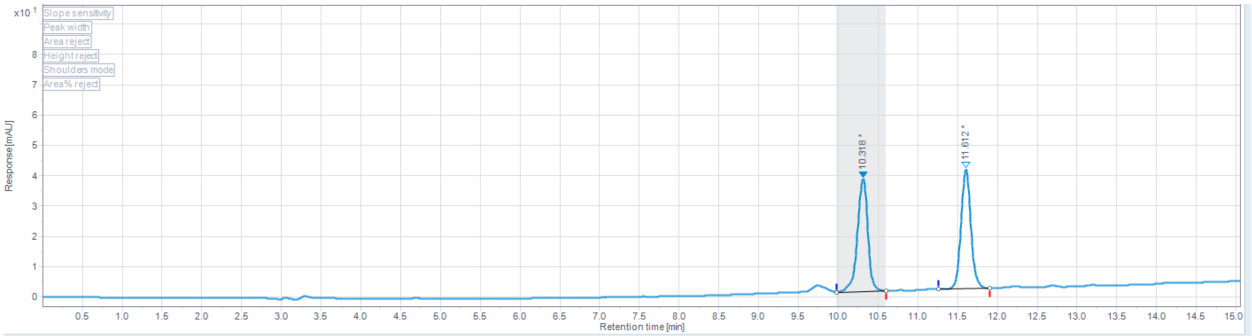

Injection Results

Peaks | Summary

#	Name	Signal description	RT (min)	Area (mAU·s)	Area%	Height (mAU)	Height%	Amount	Concentration	Start time (min)	End time (min)
1		DAD1A,Sig=254,4 Ref=off	10.318	343.735	50.198	37.274	48.68			9.988	10.608
2		DAD1A,Sig=254,4 Ref=off	11.612	341.027	49.802	39.303	51.32			11.269	11.911

(*S*)-1-(*tert*-Butyl)-1-methyl-3-(1-(naphthalen-1-yl)ethyl)urea **13**

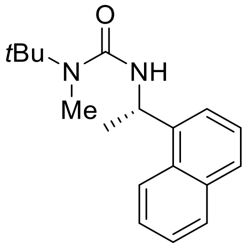

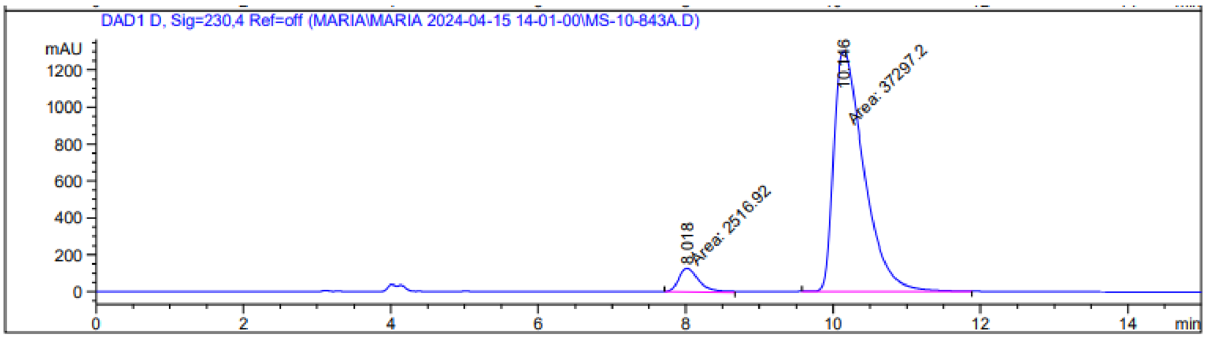

Peak #	RetTime [min]	Type	Width [min]	Area [mAU*s]	Height [mAU]	Area %
1	8.018	MM	0.3268	2516.91943	128.37782	6.3217
2	10.146	MM	0.4774	3.72972e4	1302.12207	93.6783

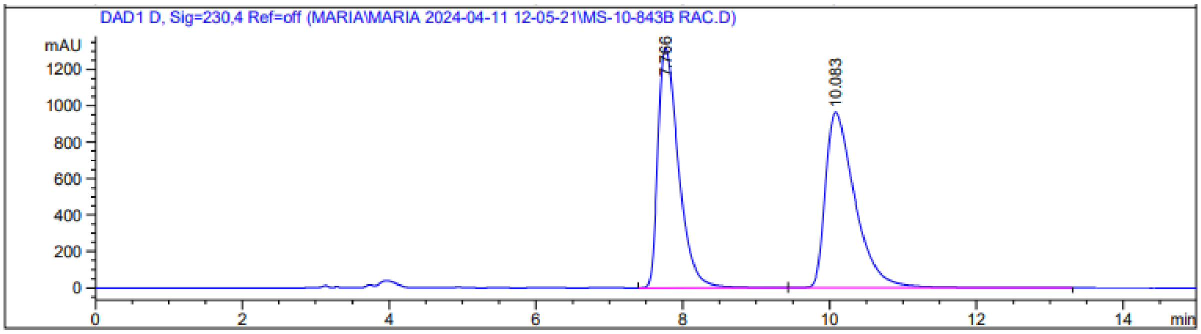

Peak #	RetTime [min]	Type	Width [min]	Area [mAU*s]	Height [mAU]	Area %
1	7.766	BB	0.2963	2.55495e4	1316.93347	48.6847
2	10.083	BB	0.4219	2.69300e4	964.08679	51.3153

tert-Butyl (*S*)-(1-(naphthalen-1-yl)ethyl)carbamate **14**

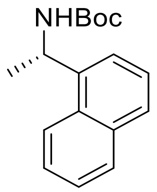

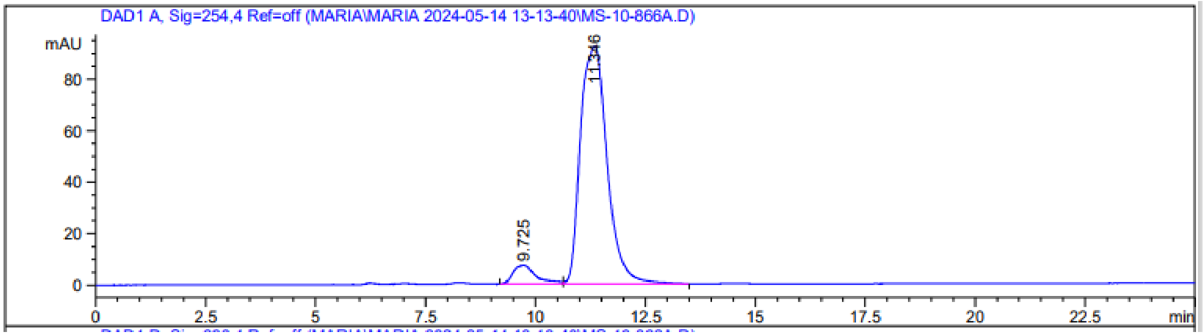

Peak #	RetTime [min]	Type	Width [min]	Area [mAU*s]	Height [mAU]	Area %
1	9.725	BV	0.4745	264.62268	7.23941	6.3751
2	11.346	VB	0.6672	3886.25220	92.31031	93.6249

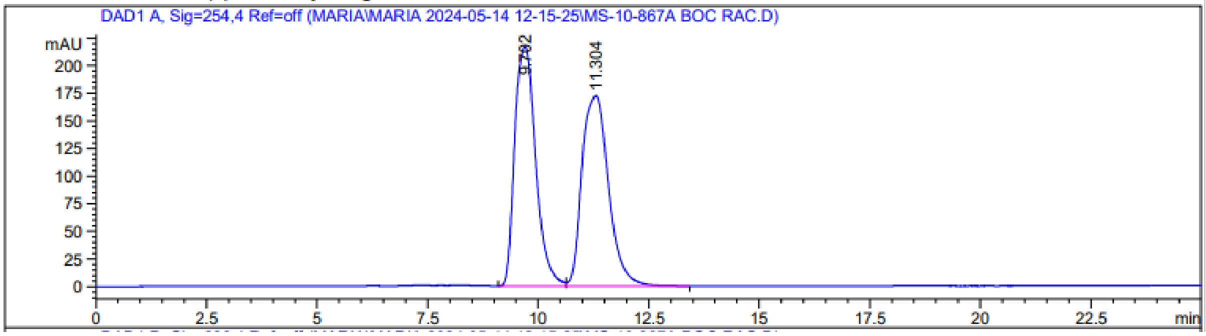

Peak #	RetTime [min]	Type	Width [min]	Area [mAU*s]	Height [mAU]	Area %
1	9.702	BV	0.5205	7147.98828	216.90039	49.8388
2	11.304	VB	0.6612	7194.22217	172.26805	50.1612

Benzyl (*S*)-(1-(naphthalen-1-yl)ethyl)carbamate **15**

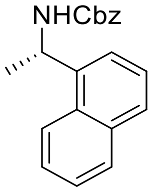

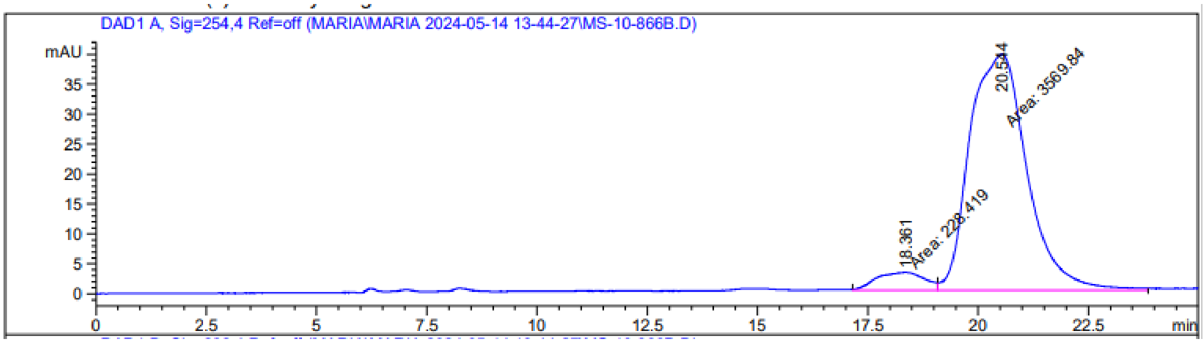

Peak #	RetTime [min]	Type	Width [min]	Area [mAU*s]	Height [mAU]	Area %
1	18.361	MM	1.2644	228.41934	3.01092	6.0138
2	20.544	MM	1.5081	3569.83545	39.45310	93.9862

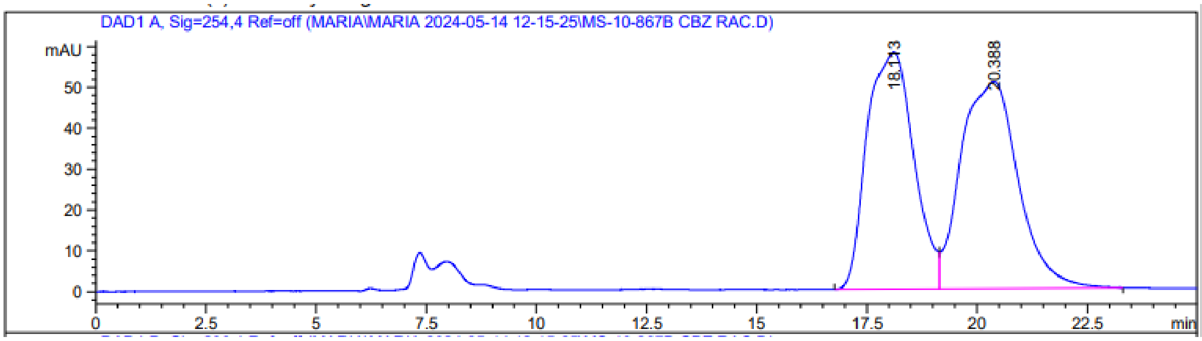

Peak #	RetTime [min]	Type	Width [min]	Area [mAU*s]	Height [mAU]	Area %
1	18.113	BV	0.9975	4275.58301	57.90025	48.2812
2	20.388	VB	1.1999	4579.99512	50.60590	51.7188

3 References

(1) Saunthwal, R. K.; Schwarz, M.; Mallick, R. K.; Terry-Wright, W.; Clayden, J. Enantioselective Intramolecular alpha-Arylation of Benzylamine Derivatives: Synthesis of a Precursor to Levocetirizine. *Angew. Chem. Int. Ed.* **2023**, *62* (14), e202216758. DOI: 10.1002/anie.202216758.
(2) Mallick, R. K.; Žabka, M.; Clayden, J. Benzo-fused Nitrogen Heterocycles by Asymmetric Ring Expansion and Stereochemically Retentive Re-contraction of Cyclic Ureas. *Angew. Chem. Int. Ed.* **2024**, *63* (12), e202318417. DOI: 10.1002/anie.202318417.
(3) Clayden, J.; Dufour, J.; Grainger, D. M.; Helliwell, M. Substituted Diarylmethylamines by Stereospecific Intramolecular Electrophilic Arylation of Lithiated Ureas. *J. Am. Chem. Soc.* **2007**, *129* (24), 7488-7489. DOI: doi.org/10.1021/ja071523a.
(4) Nechab, M.; El Blidi, L.; Vanthuyne, N.; Gastaldi, S.; Bertrand, M. P.; Gil, G. N-acyl glycinates as acyl donors in serine protease-catalyzed kinetic resolution of amines. Improvement of selectivity and reaction rate. *Org. Biomol. Chem.* **2008**, *6* (21), 3917-3920. DOI: 10.1039/b812089g.
(5) Liu, S.; Achou, R.; Boulanger, C.; Pawar, G.; Kumar, N.; Lusseau, J.; Robert, F.; Landais, Y. Copper-catalyzed oxidative benzylic C(sp(3))-H amination: direct synthesis of benzylic carbamates. *Chem. Commun.* **2020**, *56* (85), 13013-13016. DOI: 10.1039/d0cc05226d.
